# Genome Mining of *Pseudomonas* Species: Diversity and Evolution of Metabolic and Biosynthetic Potential

**DOI:** 10.3390/molecules26247524

**Published:** 2021-12-12

**Authors:** Khorshed Alam, Md. Mahmudul Islam, Caiyun Li, Sharmin Sultana, Lin Zhong, Qiyao Shen, Guangle Yu, Jinfang Hao, Youming Zhang, Ruijuan Li, Aiying Li

**Affiliations:** 1 Helmholtz International Lab for Anti-Infectives, Shandong University-Helmholtz Institute of Biotechnology, State Key Laboratory of Microbial Technology, Shandong University, Qingdao 266237, China; microkhorshed@mail.sdu.edu.cn (K.A.); 201712255@mail.sdu.edu.cn (C.L.); 201511767@mail.sdu.edu.cn (L.Z.); shenqiyao@mail.sdu.edu.cn (Q.S.); yuguangle@mail.sdu.edu.cn (G.Y.); 202012593@mail.sdu.edu.cn (J.H.); zhangyouming@sdu.edu.cn (Y.Z.); 2 Department of Microbiology, Rajshahi Institute of Biosciences (RIB), University of Rajshahi, Rajshahi 6212, Bangladesh; mislamru@gmail.com; 3 Department of Microbiology, University of Chittagong, Chittagong 4331, Bangladesh; sharminmbio@gmail.com

**Keywords:** *Pseudomonas*, genome mining, natural products, biosynthetic pathway, gene cluster, genome comparison

## Abstract

Microbial genome sequencing has uncovered a myriad of natural products (NPs) that have yet to be explored. Bacteria in the genus *Pseudomonas* serve as pathogens, plant growth promoters, and therapeutically, industrially, and environmentally important microorganisms. Though most species of *Pseudomonas* have a large number of NP biosynthetic gene clusters (BGCs) in their genomes, it is difficult to link many of these BGCs with products under current laboratory conditions. In order to gain new insights into the diversity, distribution, and evolution of these BGCs in *Pseudomonas* for the discovery of unexplored NPs, we applied several bioinformatic programming approaches to characterize BGCs from *Pseudomonas* reference genome sequences available in public databases along with phylogenetic and genomic comparison. Our research revealed that most BGCs in the genomes of *Pseudomonas* species have a high diversity for NPs at the species and subspecies levels and built the correlation of species with BGC taxonomic ranges. These data will pave the way for the algorithmic detection of species- and subspecies-specific pathways for NP development.

## 1. Introduction

Microorganisms can produce a wide range of secondary metabolite or natural products (NPs), such as non-ribosomal peptides (NRPs) [1,2,3], polyketides (PKs) [4,5], ribosomally synthesized and post-translationally modified peptides (RiPPs) [6,7,8,9], saccharides [10,11], alkaloids [12,13,14] and terpenoids [15,16,17], which offer diverse applications in the pharmaceutical and agricultural industries [18,19]. More than 50% of Food and Drug Administration (FDA) approved drugs and 65% of current clinical drugs are inspired from NPs. The biosynthesis of microbial NPs is controlled by a group of genes clustered together on the microbial chromosomes to form the biosynthetic gene clusters (BGCs) [20] allowing for the co-expression of the biosynthetic enzymes, regulators, and transporters responsible for NP production and secretion.

To combat the emerging worldwide challenge of antibiotic resistance, new antimicrobial agents are desperately needed. Antimicrobial resistance takes the lives of at least 700,000 people every year and it is expected that this number will reach 10 million by 2050 if the problem is not addressed [21,22]. Indeed, less than 25% of clinical drugs represent limited novel classes or act via novel mechanisms. Drugs active against Gram-negative *Enterococcus faecium*, *Staphylococcus aureus*, *Klebsiella pneumoniae*, *Acinetobacter baumannii*, *Pseudomonas aeruginosa*, and *Enterobacter* spp. (ESKAPE) or World Health Organization (WHO) critical threat pathogens are still far from being available.

Bioactivity-guided traditional screening used to directly extract these chemicals from microorganisms is no longer sufficient to meet the continual demand for new chemical entities due to the low production, prolonged duration, high expense, and high rediscovery rates in finding, isolating, and characterizing compounds [23]. In fact, tens of thousands of NPs known so far constitute only a small part of NPs’ potential chemical space, which has yet to be discovered [24,25,26]. These limitations could be solved by looking for BGCs in the genomes which show cryptic metabolic potential. Identification of a varied spectrum of formerly undiscovered new NPs has been made possible by the advent of powerful data mining technologies, as well as genetic and analytical instruments [27].

The availability of sophisticated computational methods and genome sequence data due to fast and low-cost next generation sequencing technologies opens up previously unexplored avenues for studying NP biosynthesis, and expands our understanding of the diversity in the producers, activities, and structures of NPs [24,27,28,29].

The molecular genetics of NP production has advanced significantly in recent years. Microbial BGCs have a lot of genetic variation, which leads to a lot of chemical variability in coding NPs [20,28]. Genome sequence analysis shows that the metabolic capacity of bacteria is substantially greater than what can be demonstrated in the lab, due to the severe silence of biosynthetic genes or poor synthesis yields, which prevent the substances from being detected by analytical methods.

New insights into the diversity and distributions of NP BGCs and evolutionary mechanisms to generate these BGCs can be gained with the access to genome sequence data. The considerable structural variety in NPs is likely caused by the more rapid evolution of BGCs in comparison to other genetic components [28]. While the selective factors to drive NP diversification are still unknown, vast numbers of genome sequences have allowed scientists to begin to disclose the evolutionary mechanisms that govern structural novelty during the biosynthesis of NPs [29]. However, more than 50% of discovered BGCs are not expressed under current laboratory circumstances, and thus are characterized as “silent”, “cryptic”, or “orphan” gene clusters.

*Pseudomonas* represents one of the most widespread and metabolically diverse bacterial genera. It includes more than 200 species [30] used for biotechnology, medicine, and environmental protection. Some members of this genus can act as opportunistic pathogens of humans, animals, and plants or show intrinsic antimicrobial resistance while some species are featured with successful colonization in many different environments, metabolic versatility, and genetic plasticity [31,32]. In addition, many members are biocontrol agents to promote plant growth and improve phytoremediation potential [33].

Genome mining has been used for the discovery and characterization of many new NPs from *Pseudomonas*, such as gacamide A, a lipodepsipeptide in *Pseudomonas fluorescens* Pf01, which has a moderate antibiotic activity and promotes bacterial surface motility [34]. Three structurally diverse lipopeptides (thanapeptin and thanamycin as well as cyclocarbamate brabantamide A–C) were isolated from *Pseudomonas* sp. SH-C52—closely linked to *P. fluorescens* DSM 11579—and showed a different antimicrobial activity spectrum [35]. Thanafactin A, a linear, proline-containing octalipopeptide, was characterized from *Pseudomonas* sp. SH-C52 [36]. Chimeric natural products pyonitrins A–D were produced by *P. protegens* [36]. Many genes involved in the biocontrol process discovered using genome mining from *P. fluorescens* BRZ63 encoded transporters, siderophores, and other active secondary metabolites [37]. Recently, *P. putida* has been widely used as a heterologous host for the biosynthesis of various NPs [38,39]. All these examples demonstrate the potential of genome mining in the discovery of NPs.

NCBI datasets have a large collection of *Pseudomonas* genome sequences, as an important source for the study of these bacteria’s biosynthetic potentialities. We utilized various bioinformatics tools to scan all publicly available complete reference genome sequences of species in the *Pseudomonas* genus and the subspecies of *P. fluorescence* to elucidate the phylogenetic diversity, distributions of known and uncharacterized BGCs, and the NP-coding potential of these genomes.

## 2. Results

### 2.1. Distribution and Diversity of Biosynthetic Potential in Pseudomonas at Species Level

A total of 50 annotated reference genomic sequences of different *Pseudomonas* species and 31 subspecies of P. fluorescence are available in NCBI genome datasets. Among them, we analyzed 37 complete genomes of *Pseudomonas* species and 23 *P. fluorescence* subspecies genomes for their biosynthetic potential with different genome mining tools. The rest of the genomes were avoided due to the lack of the *rpoB* gene and were not included in the phylogenetic tree (Supplementary Files 1 and 2).

#### 2.1.1. Putative BGC Prediction by antiSMASH in *Pseudomonas* Species Genomes

**Figure 1:**
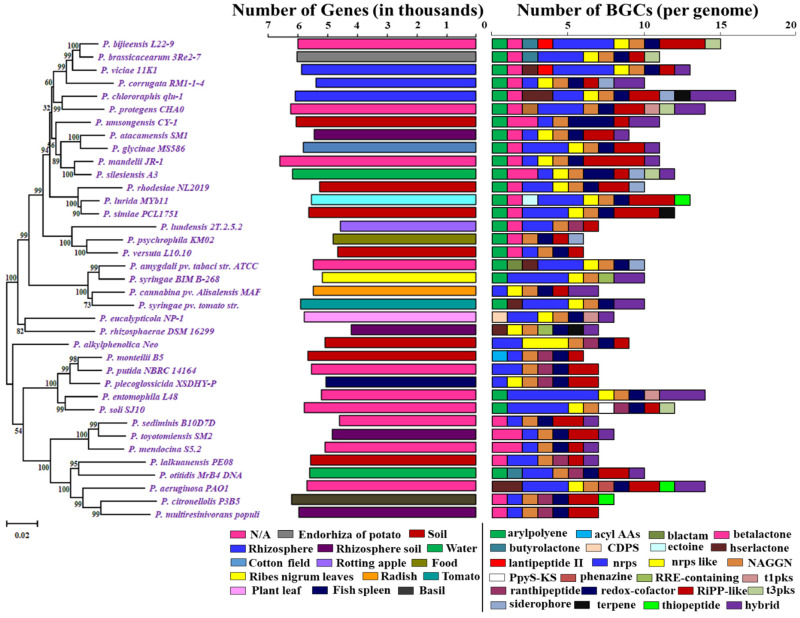
Phylogenetic tree of *Pseduomonas* along with the gene numbers, isolation sources, and NP BGCs number determined by antiSMASH. The phylogenetic tree is built using *rpoB* sequences extracted from the genomes based on the maximum likelihood method. Two bar-plots show genome size in thousands of genes on the left, colored by habitats, and the number of BGCs on the right. Species in these two bar-plots keep the same order as the phylogenetic tree. Hybrid clusters are shown separately. The colors matching to habitat types and 24 major NP classes are displayed below the bar-plots. N/A: Not Available.

All *Pseudomonas* reference genomes were scanned with antiSMASH for the exploration of known and putative secondary metabolite biosynthetic potential in their genome sequences. The diversity of these species influences the phylogenetic diversity and heterogeneity (Figure 1).

In total, the scanning of the 37 *Pseudomonas* species references genome data revealed 363 BGCs coding for small molecule classes, including nonribosomal peptides (NRPs), polyketides (PKs), ribosomally synthesized and post-translationally modified peptides (RiPPs), terpene, saccharides, and PKS/NRPS hybrids. We identified a total of 24 major BGC classes, such as aryl polyene, acyl_amino_acids, beta-lactam, beta-lactone, butyrolactone, tRNA-dependent cyclodipeptide synthases (CDPS), ectoine, hserlactone, lantipeptide class II, nonribosomal peptides (NRPs), NRPS-like, N-acetylglutamine amide (NAGGN), PpyS-KS, phenazine, RRE-containing, ranthipeptide, redox-cofactor, RiPP-like, siderophore, t1pks, t3pks, terpene, thiopeptide, and hybrid BGCs (Figure 1) (Supplementary File 3).

The most typical BGCs in *Pseduomonas* were detected to encode the multidomain enzyme nonribosomal peptide synthetase (NRPS) and polyketide synthases (PKS). Their products, nonribosomal peptides (NRPs) and polyketides (PKs), are two varied groups of secondary metabolites that have been identified as toxins, medicines, siderophores, and pigmentation agents. The analysis of the *Pseudomonas* species’ genomic sequences demonstrated their potential to produce a variety of NRPs through biosynthesis. The NRPS modules encoded in typical modular NRPS gene clusters had at least adjacent condensation (C) and adenylation (A) domains. We included NRPS-like clusters lacking the C domain in the NRPS clusters because they could actively produce secondary metabolite even without a proper C domain. A PKS type had at least a ketosynthase (KS) domain. The hybrid kind was made up of NRPS and PKS modules together. So far, three kinds of PKS have been identified in bacterium species. The polyketide chain elongation and synthesis are catalyzed non-iteratively by most type I PKS. The biosynthetic domains of type II PKSs encode iteratively active aromatic polyketides. The acyl carrier protein (ACP) is used by type I and II PKS to trigger acyl CoA precursors for the development of polyketide molecules. Enzymes iteratively active for aromatic polyketide biosynthesis independent of ACP are also found in type III PKSs.

RiPPs’ post-translational modifications increase the structural diversity of short peptides which are generally stabilized as a result of these changes, making them more resistant to heat and proteases.

Hybrid BGCs encode more than one type of scaffold-synthesizing enzymes for different types of secondary metabolites which were joined in a variety of combinations [25,40], including hybrid hserlactone–NRPS, NRPS-like–T1PKS, other–NRPS, NRPS–ranthipeptide, NRPS-like–LAP, NRPS–terpene, siderophore–NRPS, NRPS-like–NRPS–T1PKS, T1PKS–NRPS, hserlactone–NRPS, NRPS–NRPS-like, aryl polyene–resorcinol, resorcinol–aryl polyene, redox-cofactor–RiPP-like, T3PKS–CDPS, NRPS-like–beta-lactone, hserlactone–NRPS–NRPS-like, NRPS–resorcinol–ranthipeptide, NRPS-like–NRPS, NRPS–ranthipeptide, NAPAA–redox-cofactor, siderophore–NRPS–terpene, T1PKS–NRPS–NRPS-like, beta-lactone–ranthipeptide, phenazine–NRPS, hserlactone, phenazine, and RiPP-like–NRPS-like–NRPS. The origins and precise roles of these hybrid BGCs are unknown, but they facilitate significant structural and chemical alterations in the main classes of BGCs, as well as the potential to develop medically useful derivatives of a molecule [41,42]. We found a total of 32 hybrid clusters among the 37 *Pseudomonas* species. Sixteen species do not have any hybrid cluster. *P. chlororaphis* qlu-1 and *P. entomophila* L48 have the largest number of three hybrid clusters. Nine *Pseudomonas* species contain double hybrid clusters. We acquired a total of 15 unique BGCs when we separated the 25 BGCs into their hybrid forms (Supplementary File 3).

**Figure 2:**
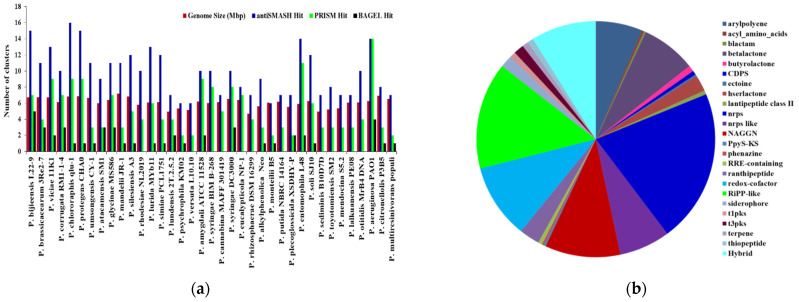
The correlation between genome size and quantity of BGCs on each genome and distributions of major classes of BGCs in *Pseudomonas* species. (**a**) The number of BGCs per genome mined by different genome mining tools is compared to the size of the genome. (**b**) Distribution of antiSMASH hits of major classes of BGCs.

In total, *Pseudomonas* bacteria carry between 6–16 BGCs per genome (mean = 9.81, s.d. = 2.85). Among the 37 genomes, the smallest genome size was found to be 4.689 Mbp in *P. rhizosphaerae* DSM 16299, which has 7 BGCs. The largest genome size, 7.189 mb, found in *P. mandelii* JR-1 had 11 BGCs, whereas the most BGCs (16) were found in *P. chlororaphis* qlu-1, whose genome size is 6.828 Mbp. Three genomes (*P. monteilii* B5, *P. versuta* L10.10 and *P. psychrophila* KM02) contain at least 6 BGCs. *P. bijieensis* L22-9 and *P. protegens* CHA0 have the second most BGCs (15) (Figure 2a). The most prevalent classes of BGCs were those encoding NRPSs, RiPPS, redox-cofactor, and NAGGN (Table 1, Figure 2b). The number of BGCs per genome has a moderate but statistically significant positive connection with genome size and total genes (R^2^ = 0.3556, *p*-value = 0.0).

The most common BGCs were for NAGGN (present in 37 genomes), non-ribosomal peptide synthetases (NRPS; 35 genomes), redox-cofactor (34 genomes), RiPP-like (31 genomes), aryl polyene, beta-lactone and NRPS-like (23 genomes), and ranthipeptide (10 genomes) (Supplementary File 3). These seven types of BGCs accounted for more than two-thirds of all the BGCs detected in a genome.

According to our findings, a BGC class can be found in numerous copies in a strain. Taking NRPS clusters as an example, 21 *Pseudomonas* species contain multiple NRPS BGCs, including *P. entomophila* L48 with the most NRPS clusters (6); *P. soli* SJ10, *P. syringae* BIM B-268, *P. viciae* 11K1; and *P. bijieensis* L22-9 with the second most clusters (4); *P. aeruginosa* PAO1, *P. syringae* pv. tomato str. DC3000, *P. amygdali* pv. tabaci str. ATCC 11528, *P. simiae* PCL1751, *P. lurida* MYb11, *P. glycinae* MS586, *P. protegens* CHA0, and *P. brassicacearum* 3Re2-7 with 3 NRPS clusters; and *P. otitidis* MrB4 DNA, *P. lalkuanensis* PE08, *P. putida* NBRC 14164, *P. alkylphenolica* Neo, *P. eucalypticola* NP-1, *P. lundensis* 2T.2.5.2, *P. rhodesiae* NL2019, and *P. chlororaphis* qlu-1 with 2 NRPS clusters. Additionally, *P. mendocina* S5.2, *P. toyotomiensis* SM2, *P. silesiensis* A3, and *P. umsongensis* CY-1 each have double beta-lactone BGCs. Seventeen *Pseudomonas* sp. have multiple RIPPS-like cluster. *P. mandelii* JR-1 has the highest number of RIPPS-like clusters, i.e., 4 (Supplementary File 3).

A few BGCs were rare, appearing in only a few genomes. They include BGCs predicated for acyl_amino_acids, beta-lactam, ectoine, PpyS-KS, phenazine (1 genome), CDPS, lantipeptide class II, RRE-containing (2 genomes), butyrolactone, t1pks, terpene, thiopeptide (3 genomes), t3pks (5 genomes), and siderophore (6 genomes) (Figure 1).

Based on our analysis in silico for the categorization of potential compounds by the BGCs in *Pseudomonas* genomes, most NRPS BGCs encrypted compounds predicted structurally to be new NRPs similar to cichopeptin, pyoverdine, TP-1161, pyochelin, putisolvin, entolysin, lokisin, rimosamide, coelibactin, ambactin, tolaasin I/F, anikasin, rimosamide, lokisin, caryoynencin, crochelin A, viscosin, and syringomycin. Some BGCs are predicted to form NRP-like compounds similar to fragin, chejuenolide A/B, ambactin, fragin, coronatine, and L-2 amino-4 methoxy-trans 3-butonoic acid.

Most of the hybrid BGCs in the genomes of *Pseudomonas* encrypted compounds predicted to have similar structures to yersiniabactin, syringomycin, pyoverdine, thuggacin, pseudomonine, pseudopyronine A/B, endophenazine A/B, pyocyanine, pseudomonic A, 1-nonadecene, rimosamide, methanobactin, and banamide 1/2/3.

#### 2.1.2. Putative BGC Prediction by PRISM in *Pseudomonas* Species Genomes

The PRISM 4 analyses for the *Pseudomonas* genome datasets revealed a total of 191 different types of BGCs (Supplementary File 3). We found a total of 97 NRPS and 41 PKS BGCs. Some hybrids clusters were also seen for melanin, NRPS-independent siderophore, ectoine, isonitrile, tabtoxin, cyclodipeptide (XYP family), acyl homoserine lactone, pantocin, aminoglycoside, class II/III confident bacteriocin, resorcinol, and class II lantipeptide, infrequently found in different genomes of *Pseudomonas*.

#### 2.1.3. Putative BGC Prediction by BAGEL in *Pseudomonas* Species Genomes

From the BAGEL4 data analysis, we identified 49 bacteriocins coding clusters for the whole genome datasets of *Pseudomonas* species (Supplementary File 3). Bacteriocins are categorized into four subgroups based on their chemical structures and modes of action. Class I bacteriocins are post-translationally modified peptides having antibacterial action. Bacteriocins of class II are antimicrobial peptides that have not undergone post-translational modification and are split into four subclasses. Bacteriocins of class III, commonly known as bacteriolysins, are heat-labile proteins having a molecular weight of >10 kDa. The C-terminal domains of these bacteriocins demonstrate similarity to endopeptidases and selectivity for target cells. Bacteriocins of class IV are cyclic bacteriocins that have undergone post-translational modification.

Most of the bacteriocins found here are annotated as class III bacteriocins with molecular weight > 10 kDa showing similarity with colicin_E6, carocin_D, colicin_E9, putidacin_L1, colicin, lin_M18, pyocin_S2, and colicin-10. Some *Pseudomonas* species are shown to produce class II bacteriocins exhibiting a similar hit to microcin, Pep5, bottromycin, class II lanthipeptide, and class III bacteriocins.

#### 2.1.4. KS and C Domain Determination in the *Pseudomonas* Genus Using NaPDoS

KS and C domains represent, respectively, the presence of BGCs for PKs and NRPs. We found a total 274 KS domains and 810 C domains from the 37 *Pseudomonas* reference genomic sequences (Supplementary File 3). The most KS domains (11) were found in *P. glycinae* MS586 and the least KS domains (2) were seen in the *P. plecoglossicida* XSDHY-P genome while the average number of KS domains was found to be 7.406. On the other hand, the highest number of C domains (70) were found in the *P. syringae* BIM B-268, and no C domain existed in the *P. psychrophila* KM02. The average C domain number was 42.63.

### 2.2. Whole-Genome Comparisons in Pseudomonas Species

**Figure 3:**
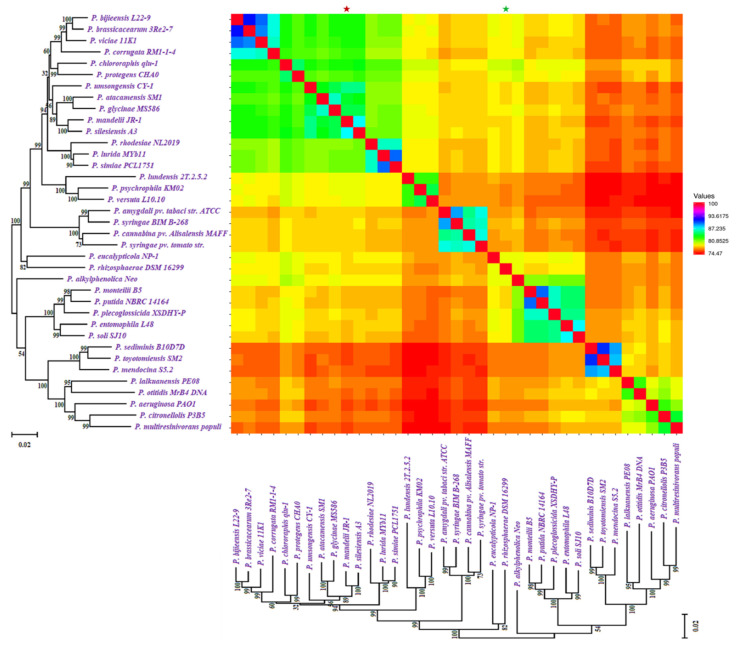
Similarity across the whole genomes of *Pseudomonas* species. Comparison follows the same sequence as the phylogenetic tree in Figure 1. All comparisons between a genome and itself take place on a line that runs from the top left to the bottom right corners of the genome. The numerator for each comparison is the number of comparable genes between two genomes, whereas the denominator is the genome represented by each column. The smallest genome is marked with a * (green), and the biggest genome is marked with a * (red).

Based on ANI (average nucleotide identity) analyses and the 95 percent threshold for species delimitation, the majority of input strain clusters were grouped into six core species identification groupings. ANI is computed using different algorithms: ANIb (ANI algorithm using BLAST), ANIm (ANI using MUMmer), OrthoANIb (OrthoANI using BLAST), and OrthoANIu (OrthoANI using USEARCH). The distribution of the six clades found in previous phylogenetic analyses is the same as in this one. Figure 3 showed the similarity across the whole genomes of our studied *Pseudomonas* species. Two strains were considered co-specific when they shared more than 95% nucleotide identity on at least 70% of their whole genome sequence.

### 2.3. Distribution and Evolution of Secondary Metabolites in Pseudomonas fluorescence at Subspecies Level

**Figure 4:**
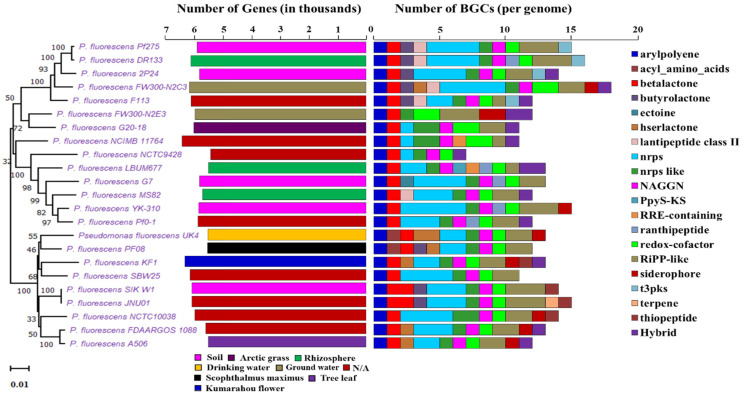
Phylogenetic tree of *P. fluorescence* sub-species along with the gene number, isolation source, and NP BGCs number determined by antiSMASH. The phylogenetic tree is built using *rpoB* sequences extracted from the genome based on the maximum likelihood method. Two bar-plots show genome size in thousands of genes on the left, colored by habitats, and the number of BGCs on the right. Species in these two bar-plots keep the same order as the phylogenetic tree. Hybrid clusters are shown separately. The colors matching to habitat types and 20 major NP classes are displayed below the bar-plots. N/A: Not available.

In order to understand the metabolic and biosynthetic potential in subspecies level of *Pseudomonas,* we chose the *P. fluorescence* reference genomes for our study. We found obvious variation in the genome size, genes number, G+C content, and biosynthetic capability among strains of *P. fluorescence* (Supplementary File 4). Figure 4 exhibits the phylogenetic relationship with the diversity of biosynthetic potential among the *P. fluorescence* subspecies with their gene numbers and habitats.

**Figure 5:**
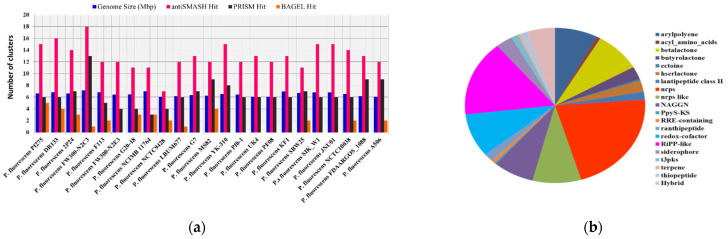
The correlation between genome size and the number of BGCs. (**a**) Comparative study of different genome mining hits with genome size (Mbp) in *P. fluorescence* subspecies and (**b**) distribution of major classes of BGCs in different *P. fluorescence* genomes.

Though strains of *P. fluorescence* share similarly sized genomes, due to belonging to a common species, the BGC number shows obvious differences between different strains. *P. fluorescens* FW300-N2C3 has the largest genome size (7.119 Mbp) with the most BGCs (18) and *P. fluorescens* NCTC9428 has least 7 BGCs with a size of 6.034 Mbp. *P. fluorescens* A506 has the smallest genome size with 12 BGCs (Table 2). The antiSMASH tool detected a total of 298 different BGCs in *P. fluorescence* reference genomes (Supplementary File 4, Figure 5b). We found a total of 20 different types of major classes of BGCs in *P. fluorescence*, predicted to be similar to arylpolyene-23, acyl_amino_acids-2, betalactone-25, butyrolactone-8, ectoine-1, hserlactone-7, lantipeptide class II-5, NRPS-64, NRPS-like-26, NAGGN-22, PpyS-KS-1, RRE-containing-2, ranthipeptide-5, redox-cofactor-27, RiPP-like-47, siderophore-9, t3pks-4, terpene-1, thiopeptide-4, and hybrid-15 (Supplementary File 4, Figure 5b).

**Figure 6:**
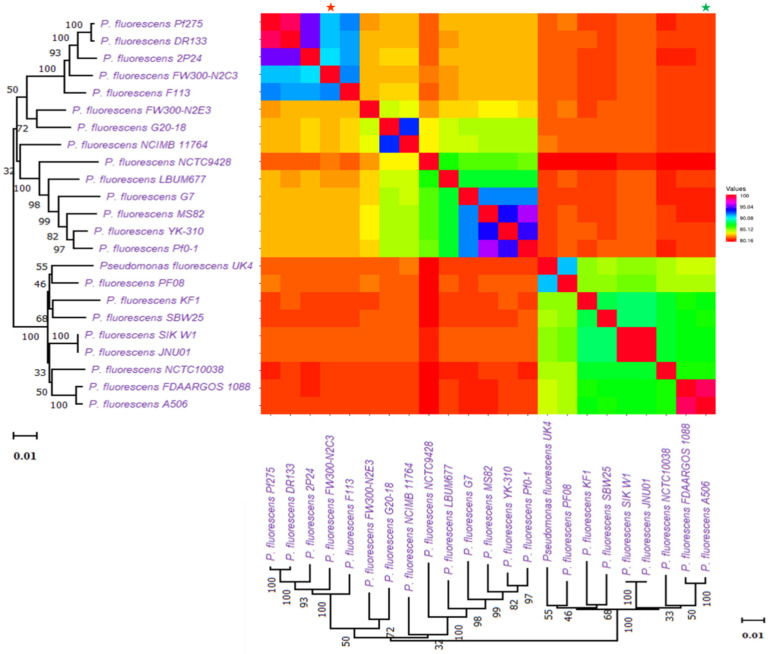
Whole genome similarity across the genomes of *P. fluorescence* sub-species. Comparison follows the same sequences as the phylogenetic tree in Figure 4. The smallest genome is marked with a * (green), and the biggest genome is marked with a * (red).

We found a total of 149 BGCs cluster detected by PRISM 4. Among them, there were 76 clusters for NRPS and 23 for PKS (Supplementary File 4). We found a total of 34 bacteriocins detected by BAGEL4 (Supplementary File 4). Most of them are colicin bacteriocins (type I). A few microcin, PaeM, putidacin, and class II lanthipeptide were also seen. On the contrary, antiSMASH hit a total of clusters for 90 RiPPs, including 47 RiPP-like compounds, 27 redox-cofactors, 5 class II lantipeptides and ranthipeptides, 4 thiopeptides, and 2 RRE-containing compounds. Whole genome similarity across genomes of *P. fluorescence* subspecies was also investigated (Figure 6). The comparison followed the same sequences as the phylogenetic tree in Figure 4.

## 3. Discussion

Projects to sequence the genomes of microorganisms at the early stages of their development discovered dozens of cryptic biosynthetic areas inside the industrially important, well-studied bacterial genomes and sparked hopes that genome mining would lead to a new “golden era” of novel NPs.

The main goal of this study was to identify probable drug-like metabolites using publicly available data for *Pseudomonas* species and *P. fluorescens* sub-species reference genomes from NCBI. Despite earlier thorough research, our findings demonstrated that both *Pseudomonas* species and *P. fluorescence* sub-species have a large and distinct natural product metabolic potential with high diversity, indicating that they are still a good source of novel metabolites.

Comparative genomic analysis is an effective approach for revealing microorganisms’ capacity for the production of novel specialized compounds. Comparative genomics investigations in NP fields have revealed that there is a plethora of new compounds embedded in both culturable and non-culturable microorganism genomes waiting to be revealed. The findings that follow add to our knowledge of their genetics and behaviors.

The research presented here is the first step in establishing a comprehensive methodology for analyzing natural compounds from the *Pseudomonas* genus. The BGC patterns indicated that certain species and sub-species of *Pseudomonas* and *P. fluorescence* had a higher incidence of metabolic potentials in NPs than others. We grouped every gene cluster in each genus well-represented by whole genomes using different comparisons. Such gene cluster families are necessary for cluster determination.

Comparative genomics revealed the similarity and difference between the species despite their differences in geography, morphology, and secondary metabolite profiles. Gene cluster networking highlights that this genus is distinctive in the number of secondary metabolite pathways, distinct from all other bacterial gene clusters to date. These findings portend that future genome-guided secondary metabolite discovery and isolation efforts should be highly productive.

Besides most of the BGC NRPSs common in *Pseudomonas* predicted for new NRPs, *Pseudomonas* genomes carry some BGCs for arylpolyene type compounds, similar to APE Vf, syringolin A, beta-lactone type compounds, similar to fengycin, burkholderic acid, tetarimycin A/B, redox-cofactor type compounds, similar to lankacidin C, ranthipeptide type compounds, similar to pyoverdine, NAGGN type compounds, similar to O-antigen, hserolactone type compounds, similar to toxoflavin/frevenulin, cepacin A, resorcinol type compounds, similar to pyoverdine, T3 PKS type compounds, similar to Fischer indole, siderophore type compounds, similar to xanthoferrin, vibrioferrin, terpene type compounds, similar to sodorifen, bacillomycin D, carotenoid, 2-methylisoborneol, thiopeptide type compounds, and similar to lipopolysaccharide. There are also some unspecified BGCs found for LAP, beta-lactone, RiPP-like, NAGGN, hserolactone, acyl_amino_acids, NAGGN, butyrolactone, T3 pks, siderophore, aryl polyene, and RRE-containing compounds.

Hence, the data here will help us in future BGC prioritization. For example, we found that all the *Pseudomonas* species and *P. fluorescence* subspecies contain the pyoverdine gene cluster, where most of them encoded more than one pyoverdine BGC. All the redox-cofactor BGC type encoded lankacidin C, which showed a considerable antitumor activity [43]. Interestingly, all the redox-factor encoded lankacidin BGC showed only a 13% similarity with most known BGCs of lankacidin C, implying a high possibility to isolate lankacidin-analogues with new structures.

However, beta-lactam, CDPS, phenazine, and terpene BGCs are not seen in *P. fluorescence* reference genomes. The findings show that the genus has a high level of route diversity, with the majority having been gained very recently in its history. The patterns and phylogenetic trajectories of these routes reveal the processes that create novel compound variety, as well as the tactics bacteria adopt to enhance their population-level ability to manufacture various molecules.

The high diversity of NP BGCs at the subspecies level demonstrated that the secondary metabolite production pathways are among the fastest-evolving genomic elements yet found [44]. Gene duplication, loss, HGT, NRPS, and PKS genes alteration, domain reorganization, and module redundancy [44,45,46] probably contribute to the emergence of novel small-molecule diversity.

The phylogenetic trajectories of individual PKS and NRPS domains have been noted, especially as pertains to the use of the KS and C domains to reveal information on enzyme design and function [47,48]. These studies have also contributed to the understanding of how widespread HGT is among biosynthetic genes for NP production [49,50], and the variation among PKS and NRPS gene phylogenies [51]. Although establishing the evolutionary histories of complete pathways is more difficult than resolving the evolutionary histories of individual genes or domains, comparative investigations of BGCs have been beneficial in identifying route boundaries [52].

In all, *Pseudomonas* species have demonstrated significant variation within the genus, and among species, and even strains within the same species, according to comparative genomics studies. Many of these BGCs were strain-specific, supporting the theory that they perform specialized metabolic tasks unique to certain ecological niches.

## 4. Materials and Methods

### 4.1. Collection of Genome Sequences

We used the NCBI Datasets’ genome browser (NCBI: https://www.ncbi.nlm.nih.gov/datasets/genomes/, accessed on 31 August 2021) to search for and collect the *Pseudomonas* complete genome sequences. We found a total of 27,125 different types of *Pseudomonas* genomes, including contigs, scaffold, chromosome, and complete genome. We filtered, as reference genome, an annotated and complete assembly level to obtain *Pseudomonas* genome sequences and retrieved 50 complete reference genome sequences in FASTA format of different *Pseudomonas* species and 31 complete reference genome sequences of *Pseudomonas fluorescens* from NCBI datasets on 31 August 2021. We discarded the 13 *Pseudomonas* and 7 *P. fluorescence* reference genomes from our study due to the lack of *rpoB* gene in these sequences (Supplementary Files 1 and 2). Supplementary Files 3 and 4 show genome assembly, accession numbers, and genome information (genome size, genes number, and genes of protein coding).

### 4.2. Phylogeny and Whole Genome Comparisons

The *rpoB* sequences were extracted from the genomic assemblies and aligned using MEGA X. [53]. The phylogenetic tree was constructed using *rpoB* sequences in these genomes (Supplementary Files 1 and File 2). Some genome sequences lacked *rpoB* genes, and others were in poor conditions; therefore they were removed from the phylogenetic tree. Using the program MEGA X [53] and a general time reversible (GTR) nucleotide substitution model [54], four gamma categories for rate heterogeneity, and 100 bootstrap replicates, the *rpoB* sequences were utilized to construct a maximum likelihood phylogeny (Supplementary Files 1 and 2).

Comparative genomics analyses were obtained using the pairwise average nucleotide identity (ANI) with an improved ANI algorithm, called OrthoANI [55] to check the genetic diversity among genomes, or clear species boundaries (Supplementary Files 3 and 4). Typically, the ANI values between genomes of the same species are above 95%.

### 4.3. Computational Approaches for the Identification of Gene Clusters Potentially Encoding Secondary Metabolites

We calculated the number of BGCs for each genome based on the three methodologies. The genome mining prediction platforms, namely, antiSMASH 6 [56], PRISM 4 [57] and BAGEL4 [58], using a combination of computational programs with default settings were implemented for the possible discovery of BGCs involved in the production of secondary metabolites. The antiSMASH tool makes it easy to find, annotate, and research secondary metabolite biosynthesis gene clusters all throughout the genome. Similarly, BAGEL4 is meant to comprehensively mine RiPPs and bacteriocin [58], whereas PRISM 4 is developed to analyze secondary metabolite structure and biological activity in a complete manner [57]. These sophisticated computer model services give accurate predictions of the encoding potential of microbial secondary metabolites [59]. These programs use several database systems for BGC annotation from genomic sequences, such as the principles of the hidden Markov model (HMM) [60], BLAST algorithm [61], PFAM [62], GenBank [63], UniProtKB [64], BACTIBASE [65] CAMPR3 [66], and the MIBig data repository [67]. Furthermore, we used NaPDoS [68] to detect KS and C domains in these genomic sequences.

#### 4.3.1. antiSMASH 6.0

The antiSMASH 6.0 tool is an advanced and rigorous bioinformatics platform that uses a predictive method to identify and annotate existing and suspected undiscovered BGCs. The public version of antiSMASH 6.0 can be found online (antiSMASH: https://antismash.secondarymetabolites.org/#!/start, accessed on 31 August 2021) while R&D versions can be found online (R&D versions: https://bitbucket.org/antismash/, accessed on 31 August 2021) [56]. Profile hidden Markov models (pHMMs), as published by Medema et al., and the tool HMMER were used to find signature enzymes for the main categories of bioactive molecules [69]. The antiSMASH tool can create a database of presently existing BGCs across the tree of life “Minimum Information about a Biosynthetic Gene cluster” (MIBiG) community project (MIBiG: http://mibig.secondarymetabolites.org, accessed on 31 August 2021). The current antiSMASH version, which includes the ClusterFinder and ClusterBlast packages, may now detect potential unexplored forms of BGCs based on comparisons to existing BGCs and final chemical product information [56].

#### 4.3.2. PRISM 4

PRISM 4 analyzes open reading frames with a library of hundreds of hidden Markov models and curated BLAST databases to annotate bacterial genomes for BGCs, and allows for genome-guided chemical structure prediction for every class of bacterial natural antibiotics now in use in clinical trials. Furthermore, PRISM 4 dramatically improves the coverage of enzymatic tailoring processes encoded inside conventional thiotemplated pathways. In order to predict the chemical structures of 16 different classes of secondary metabolites, PRISM 4 includes 1772 hidden Markov models (HMMs) and 618 in silico tailoring reactions. PRISM 4 as a freely accessible web server is available at (PRISM: https://prism.adapsyn.com/, accessed on 31 August 2021).

#### 4.3.3. BAGEL4

BAGEL4, a user-friendly web server, allows researchers to mine bacterial (meta-) genomic DNA for ribosomally synthesized and post-translationally modified peptides (RiPPs) and (unmodified) bacteriocin. BAGEL4 is the most recent edition of the BAGEL package. Due to the need for new antibiotics and their crucial function in preserving food, microbial ecology, and plant biocontrol, demand in these families of compounds is growing. BAGEL4 is available for free online (BAGEL4: http://bagel4.molgenrug.nl, accessed on 31 August 2021). It also includes directories as well as a BLAST against the core peptide databases. The mining databases have been updated and expanded to include literature references as well as connections to UniProt and NCBI. It also contains an automatic promoter and terminator prediction, as well as the ability to submit RNA expression data to be presented alongside the clusters found. Additional enhancements include the annotation of context genes, which is now based on a quick blast against the UniRef 90 database’s prokaryote component, and the enhanced web-BLAST function, which dynamically imports structural data from UniProt such as internal cross-linking.

#### 4.3.4. NaPDoS-Analysis of C and KS Domains from NRPS and PKS Clusters

NaPDoS [68], which is accessible online ( NaPDoS: https://npdomainseeker.sdsc.edu/, accessed on 31 August 2021) as a fast way to extract and categorize ketosynthase (KS) and condensation (C) domains from PCR products, genomes, and metagenomic datasets. Condensation (C) domains are functionally active protein sequences found in NRPS clusters that catalyze the creation of amide bonds, a key step in peptide elongation [70]. Likewise, in PKS clusters, ketosynthase (KS) domains catalyze the condensation process. These domains are good candidates for genomic study since they are highly conserved and may be utilized to differentiate between distinct NRPS/PKS natural product pathways. To uncover probable natural product pathways from NRPS and PKS gene clusters, the NaPDoS pipeline was utilized to compare C and KS domain sequences to a domain library of previously found natural products. Close database matches may be used to anticipate secondary metabolite generalized structures, whereas unique phylogenetic lineages can be utilized to discover new enzyme designs or secondary metabolite assembly processes. The findings provide a rapid method for analyzing secondary metabolite biosynthesis gene diversity and abundance in species or habitats, as well as a method for identifying genes associated with unknown biochemistry. The output from antiSMASH was used to extract the C and KS domains from NRPS and PKS found in the 37 *Pseudomonas* species and *P. fluorescence* genomes, which were then examined using the NaPDoS web server with default parameters.

## 5. Conclusions

Less than 10 percent of microorganisms’ biosynthetic capabilities are utilized in searching for bioactive NPs. Genome mining has tremendously benefited natural product developments. Currently, the genome sequences’ availability of diverse species of *Pseudomonas* and sub-species of *P. fluorescence* provides an excellent opportunity for comprehensive comparisons of their biosynthetic potential.

Here, by combining different computational tools, the species and sub-species genomic sequences of *Pseudomonas* were analyzed in silico and revealed a wide range of biosynthetic capabilities to produce diverse sets of secondary metabolites. These putative secondary metabolite coding clusters (BGCs) are promising targets for further research to uncover additional resources.

Large amounts of genomic data are now public, and significant progress has been made in data mining, chemical monitoring, single-cell techniques, and genetic approaches to pathway activation, making the cryptic metabolome accessible. New culturing methods, effective genome editing, and appropriate expression systems will eventually overcome key impediments to obtain hidden chemical diversity.

It is notable that additional methodologies are required to decipher these biosynthetic genome motifs into corresponding compounds to open a new era in the discovery of secondary metabolism. Specific triggers or stimuli are required to activate quiet or downregulated gene clusters and enhance compound production rates, allowing access to these cryptic compounds [71].

## Figures and Tables

**Table 1:** Reference genomes of *Pseudomonas* species studied with different hits from different genome mining tools for BGCS.

Species	Isolation Source	Size (mb)	Genes	antiSMASH	PRISM	BAGEL	KS Domains	C Domain
				Hit	Hit	Hit		
*P. bijieensis* L22-9	N/A	6.730	5984	15	7	5	8	37
*P. brassicacearum* 3Re2-7	Endorhiza of potato	6.739	6014	11	4	3	8	27
*P. viciae* 11K1	Rhizosphere	6.705	5868	13	9	2	7	66
*P. corrugata* RM1-1-4	Rhizosphere	6.124	5394	10	7	3	8	45
*P. chlororaphis* qlu-1	Rhizosphere	6.828	6093	16	9	1	11	26
*P. protegens* CHA0	N/A	6.868	6252	15	9	1	10	28
*P. umsongensis* CY-1	Soil	6.690	6060	11	3	1	8	12
*P. atacamensis* SM1	Rhizospheric soil	5.991	5436	9	3	3	7	13
*P. glycinae* MS586	Cotton field	6.397	5818	11	7	3	11	26
*P. mandelii* JR-1	N/A	7.189	6604	11	3	1	10	12
*P. silesiensis* A3	Wastewater	6.824	6166	12	5	1	9	12
*P. rhodesiae* NL2019	Soil	5.779	5262	10	4	0	7	17
*P. lurida* MYb11	Rotting apple	6.101	5549	13	6	1	7	24
*P. simiae* PCL1751	Soil	6.144	5643	12	4	1	7	16
*P. lundensis* 2T.2.5.2	Meltwater pond	4.934	4563	7	4	2	7	12
*P. psychrophila* KM02	Food	5.314	4813	6	2	1	7	0
*P. versuta* L10.10	Soil	5.15	4671	6	2	0	6	10
*P. amygdali* pv. tabaci str. ATCC 11528	N/A	6.202	5489	10	9	2	6	26
*P. syringae* BIM B-268	Ribes nigrum leaves	6.019	5165	10	8	0	6	70
*P. cannabina* pv. alisalensis MAFF 301419	Radish	6.145	5486	7	5	0	6	27
*P. syringae* pv. tomato str. DC3000	Tomato	6.538	5891	10	8	3	9	32
*P. eucalypticola* NP-1	Plant leaf	6.402	5782	8	7	0	4	29
*P. rhizosphaerae* DSM 16299	Rhizospheric soil	4.689	4214	7	4	0	6	3
*P. alkylphenolica* Neo	Soil	5.612	5092	9	3	1	4	23
*P. monteilii* B5	Soil	6.079	5661	6	2	1	5	17
*P. putida* NBRC 14164	N/A	6.157	5539	7	3	0	5	17
*P. plecoglossicida* XSDHY-P	Fish spleen	5.526	5067	7	2	2	2	11
*P. entomophila* L48	N/A	5.889	5199	14	11	2	10	44
*P. soli* SJ10	N/A	6.248	5798	12	6	1	7	32
*P. sediminis* B10D7D	N/A	4.934	4612	7	3	0	9	9
*P. toyotomiensis* SM2	Rhizospheric soil	5.235	4857	8	3	0	8	11
*P. mendocina* S5.2	N/A	5.372	5081	7	3	0	10	9
*P. lalkuanensis* PE08	Soil	6.057	5558	7	3	0	7	12
*P. otitidis* MrB4 DNA	Water	6.089	5615	10	4	0	9	13
*P. aeruginosa* PAO1	N/A	6.264	5697	14	14	4	6	21
*P. citronellolis* P3B5	Basil	6.951	6219	8	3	1	8	14
*P. multiresinivorans populi*	Rhizosphere soil	6.518	5974	7	2	1	9	7

N/A: Not Available.

**Table 2:** List of different *P. fluorescence* reference genomes with different hits.

Species Name	Source	Size	Genes	AntiSMASH	BAGEL	PRISM	KS Domain	C Domain
		(Mbp)		Hit	Hit	Hit		
*P. fluorescens* Pf275	Soil	6.61	5884	15	5	6	8	37
*P. fluorescens* DR133	Rhizosphere	6.848	6102	16	4	6	8	33
*P. fluorescens* 2P24	Soil	6.611	5803	14	3	7	19	34
*P. fluorescens* FW300-N2C3	Ground water	7.119	6149	18	1	13	10	80
*P. fluorescens* F113	N/A	6.846	6093	12	2	5	13	17
*P. fluorescens* FW300-N2E3	Ground water	6.392	5951	12	0	4	10	2
*P. fluorescens* G20-18	Arctic grass	6.481	6001	11	3	4	8	13
*P. fluorescens* NCIMB 11764	N/A	6.998	6404	11	3	3	8	13
*P. fluorescens* NCTC9428	N/A	6.034	5413	7	2	4	6	16
*P. fluorescens* LBUM677	Rhizosphere	6.14	5487	12	1	6	6	25
*P. fluorescens* G7	Soil	6.336	5804	13	0	7	8	26
*P. fluorescens* MS82	Rhizosphere	6.208	5690	12	4	9	11	26
*P. fluorescens* YK-310	Soil	6.499	5825	15	0	8	9	41
*P. fluorescens* Pf0-1	N/A	6.438	5852	12	0	6	9	33
*P. fluorescens* UK4	Drinking water	6.064	5513	13	0	6	6	19
*P. fluorescens* PF08	Scophthalmus maximus	6.031	5518	12	0	6	7	0
*P. fluorescens* KF1	Kumarahou flower	6.957	6306	13	0	6	8	15
*P. fluorescens* SBW25	N/A	6.723	6123	11	2	7	8	33
*P. fluorescens* SIK_W1	Soil	6.791	6058	15	0	6	7	24
*P. fluorescens* JNU01	N/A	6.79	6058	15	0	6	7	24
*P. fluorescens* NCTC10038	N/A	6.515	5965	14	2	6	6	22
*P. fluorescens* FDAARGOS_1088	N/A	6.135	5585	13	0	9	7	16
*P. fluorescens* A506	Tree leaf	6.02	5493	12	2	9	7	15

N/A: Not Available.

## Data Availability

Not applicable.

## Supplementary Materials

The following are available online. Supplementary File 1: *rpoB* sequences from genomes of *Pseudomonas* species; Supplementary File 2: *rpoB* sequences from genomes of *P. fluorescence* subspecies; Supplementary File 3 & 4: different genome mining data from *Pseudomonas* species and *P. fluorescence* subspecies.
