# Supplementary material for: Genome Mining of Pseudomonas Species: Diversity and Evolution of Metabolic and Biosynthetic Potential
Source: Molecules. 2021 Dec 12;26(24):7524. doi: 10.3390/molecules26247524 (PMC8704066; doi:10.3390/molecules26247524)
Supplement: Supplementary file 1 [file molecules-26-07524-s001.zip › Supplementary file 1.pdf]

P. aeruginosa PAO1_ GCF_000006765.1_ AE004091.2

ATGGCTTACTCATACACTGAGAAAAAACGTATCCGCAAGGACTTTAGCAAGTTGCCGGACGTCATGGATGTGCCGTATTTGCTGG
CCATCCAGCTGGATTCCTATCGCGAATTCCTGCAGGCTGGCGCAACCAAGGAGCAGTTCCGCGATGTCGGTCTGCACGCGGCCTT
CAAGTCCGTTTTCCCGATTATCAGCTATTCCGGCAATGCTGCCCTGGAATACGTCGGCTACCGTCTGGGTGAGCCGGCATTCGATG
TCAAGGAGTGCGTGCTGCGCGGCGTGACCTTCGCCGTACCGCTGCGCGTGAAAGTTCGCCTGATCATCTTCGACCGCGAGTCGTC
GAACAAGGCGATCAAGGACATCAAGGAACAAGAAGTCTACATGGGGGAAATCCCCCTGATGACCGAGAACGGTACCTTCATCAT
CAACGGTACCGAGCGTGTCATCGTCTCCCAGCTGCACCGTTCCCCGGGCGTGTTCTTCGACCACGACCGTGGCAAGACCCACAGC
TCCGGCAAGCTGCTGTACTCCGCGCGGATCATTCCTTACCGCGGTTCCTGGCTGGACTTCGAGTTCGATCCGAAGGACTGCGTGTT
CGTCCGTATCGACCGTCGCCGCAAGCTGCCGGCCTCGGTACTGCTGCGCGCGCTCGGCTACAGCACGGAAGAGATCCTCAACGCC
TTCTACGCGACCAACGTCTTCCACATCAAGGGCGAGACCCTGAACCTGGAACTGGTCCCGCAGCGCCTGCGCGGTGAAGTCGCGA
GCATCGACATCAAGGATGGCAGCGGCAAGGTGATCGTGGAGCAGGGGCGTCGTATCACTGCCCGCCACATCAACCAGCTGGAAA
AGGCTGGCGTGAGCCAGCTGGAAGTGCCGTTCGACTACCTGATCGGCCGTACCATCGCCAAGGCGATCGTGCATCCGGCTACCGG
CGAGATCATCGCCGAGTGCAACACCGAGCTGACCCTCGACCTCCTGGCCAAGGTGGCCAAGGCCCAGGTCGTGCGCATCGAGACC
CTGTACACCAACGACATCGACTGCGGTCCGTTCATCTCCGACACCCTGAAGATCGACAACACCAGCAACCAGCTGGAAGCCCTGG
TCGAGATCTACCGGATGATGCGTCCGGGCGAGCCGCCGACCAAGGAAGCTGCCGAGACCCTGTTCGGCAACCTGTTCTTCAGCGC
CGAGCGTTACGACCTGTCGGCCGTAGGCCGGATGAAGTTCAACCGCCGTATCGGTCGTACCGAGATCGAAGGTCCGGGCGTCCTG
AGCAAGGAAGACATCATCGATGTGCTCAAGACCCTCGTCGACATCCGTAACGGCAAGGGCATCGTCGATGACATCGACCACCTGG
GCAACCGTCGTGTCCGTTGCGTCGGCGAAATGGCCGAGAACCAGTTCCGCGTGGGCCTGGTGCGTGTCGAGCGCGCGGTCAAGGA
ACGCCTGTCCATGGCCGAAAGCGAAGGCCTGATGCCGCAAGACCTGATCAACGCCAAGCCGGTGGCTGCCGCGATCAAGGAGTT
CTTCGGTTCGAGCCAGCTGTCGCAGTTCATGGACCAGAACAACCCGCTTTCCGAGATCACCCACAAGCGCCGCGTCTCCGCGCTC
GGCCCGGGCGGTCTGACCCGTGAGCGTGCGGGCTTCGAGGTTCGTGACGTACACCCGACCCACTACGGCCGCGTGTGCCCGATCG
AAACCCCTGAAGGTCCGAACATCGGTCTGATCAACTCCCTGGCGACCTACGCCCGCACCAACAAGTACGGCTTCCTCGAGAGCCC
GTACCGCGTGGTCAAGGACAGCCTGGTAACCGACGAGATCGTGTTCCTGTCGGCGATCGAAGAAGCCGACCACGTCATCGCCCAG
GCTTCGGCGACCCTCAACGAGAAGGGTCAACTGGTGGACGAGCTGGTGGCCGTGCGTCACCTGAACGAATTCACCGTGAAGGCG
CCGGAAGACGTGACCCTGATGGACGTGTCGCCGAAGCAGGTCGTTTCCGTCGCTGCCTCGCTGATTCCGTTCCTCGAGCACGATG
ACGCCAACCGCGCACTCATGGGCTCGAACATGCAGCGTCAGGCCGTGCCGACCCTGCGTGCCGACAAGCCGCTGGTGGGTACCGG
CATGGAGCGCAACGTGGCGCGCGACTCCGGCGTCTGCGTCGTGGCTCGCCGTGGCGGTGTGATCGACTCGGTCGATGCCAGCCGT
GTCGTGGTTCGCGTGGCGGATGACGAAGTCGAGACCGGCGAAGCGGGTGTCGACATCTACAACCTGACCAAGTACACTCGTTCCA
ACCAGAACACCTGCATCAACCAGCGTCCGCTGGTGAGCAAGGGTGACGTGGTCGCGCGCGGCGACATCCTGGCCGACGGTCCGTC
CACCGACATGGGCGAACTGGCCCTGGGCCAGAACATGCGCGTAGCGTTCATGCCCTGGAACGGCTTCAACTTCGAAGACTCCATC
TGCCTGTCCGAGCGCGTGGTCCAGGAAGATCGTTTCACCACGATCCACATCCAGGAACTGACCTGCGTCGCTCGTGACACCAAGC
TCGGCCCAGAGGAAATCACCGCGGACATCCCGAACGTGGGCGAGGCCGCGCTGAACAAGCTGGACGAAGCCGGTATCGTCTACG
TCGGCGCCGAAGTGCAGGCCGGCGACATCCTGGTCGGCAAGGTCACTCCGAAAGGCGAGACCCAGCTGACTCCGGAAGAGAAGC
TGCTGCGCGCGATCTTCGGTGAGAAGGCGTCCGACGTGAAGGACACCTCCCTGCGTGTGCCGACCGGCACCAAGGGTACCGTCAT
CGACGTTCAGGTCTTCACCCGCGACGGCGTCGAGCGCGATTCCCGCGCGCTGTCCATCGAGAAGATGCAACTCGACCAGATCCGC
AAGGACCTGAACGAAGAGTTCCGCATCGTCGAAGGCGCGACCTTCGAGCGTCTGCGTGCCGCCCTGGTCGGTGCCAAGGCTGAAG
GTGGCCCGGCGCTGAAGAAGGGCACGGAGATCACCGACGACTACCTCGACGGTCTCGAGCGCGGCCAGTGGTTCAAGCTGCGCA
TGGCCGACGACGCCCTGAACGAACAGCTGGAGAAGGCCCAGGCCTACATCAGCGATCGTCGCCAGCTCCTGGACGACAAGTTCG
AGGACAAGAAGCGCAAGCTGCAGCAGGGCGACGACCTGGCTCCGGGCGTGCTGAAGATCGTCAAGGTCTACCTGGCGATCAAGC
GTCGCATCCAGCCGGGCGACAAGATGGCCGGCCGTCACGGTAACAAGGGTGTGGTCTCGGTGATCATGCCGGTGGAAGACATGC
CGCACGATGCCAACGGCACCCCGGTCGACATCGTCCTCAACCCGCTGGGCGTACCGTCGCGTATGAACGTCGGTCAGATCCTCGA
AACCCACCTGGGCCTCGCGGCCAAGGGGCTGGGCGAGAAGATCAACCGCATGCTCGAAGAGCAGCGCAAGGTCGCCGAACTGCG
TAAGTTCCTGCACGAGATCTACAACGAGATCGGCGGTCGCGAGGAAAACCTCGACGAGCTGGGCGACAACGAGATCCTCGCGCT
GGCCAAGAACCTGCGCGGTGGCGTACCGATGGCGACCCCGGTGTTCGATGGCGCCAAGGAACGCGAGATCAAGGCCATGCTGAA
GCTGGCCGACCTGCCGGAAAGCGGCCAGATGCGTCTGTTCGACGGCCGTACCGGCAACCAGTTCGAGCGTCCGACCACCGTCGGC
TACATGTACATGCTCAAGCTGAACCACCTGGTGGACGACAAGATGCACGCCCGTTCCACCGGCTCGTACAGCCTGGTTACCCAGC
AGCCGCTGGGTGGTAAGGCACAGTTCGGTGGTCAGCGCTTCGGTGAGATGGAGGTGTGGGCGCTGGAAGCCTATGGCGCGGCGT
ACACCCTGCAGGAAATGCTGACGGTCAAGTCGGACGACGTGAACGGCCGGACCAAGATGTACAAGAACATCGTGGACGGCGATC
ACCGCATGGAGGCCGGCATGCCCGAGTCCTTCAACGTTCTGATCAAAGAGATCCGTTCGCTCGGCATCGACATCGAACTGGAAAC
CGAATAA

P. alkylphenolica Neo_GCF_009755645.1_CP046621:

ATGGCTTACTCATACACTGAGAAAAAACGTATCCGCAAGGACTTTAGCAAGTTGCCGGACGTCATGGATGTGCCTTACCTCCTGG
CCATCCAGCTGGATTCGTATCGTGAATTCTTGCAAGCGGGAGCGACCAAAGATCAGTTCCGCGACGTGGGCCTGCATGCGGCCTT
CAAATCGGTTTTCCCGATCATCAGCTACTCCGGCAATGCTGCCCTGGAGTACGTTGGCTATCGTCTGGGCGAACCGGCTTTTGATG
TCAAAGAATGCGTACTGCGCGGCGTGACCTACGCGGTACCGCTGCGGGTAAAAGTCCGCCTGATCATTTTCGACAAAGAATCGTC
GAACAAAGCGATCAAGGACATCAAAGAGCAAGAAGTCTACATGGGTGAAATCCCCCTGATGACTGAAAACGGTACCTTCGTAAT
CAACGGTACCGAGCGTGTTATCGTTTCCCAGCTGCACCGTTCGCCGGGCGTGTTCTTCGATCACGACCGCGGCAAAACGCACAGC
TCTGGCAAACTGCTGTACTCGGCGCGGATCATTCCGTACCGCGGCTCGTGGCTGGACTTCGAGTTCGATCCGAAAGACTGTGTATT
CGTCCGTATCGACCGTCGTCGCAAGCTGCCTGCGTCGGTATTGCTGCGCGCGCTGGGCTACAGCACTGAAGAAGTGCTCAACGCT
TTCTACACCACCAACGTATTCCACGTGTCGGGCGAGAGCCTGAGCCTGGAGCTGGTGCCTCAGCGCCTGCGTGGTGAAGTTGCGG
TCATGGACATCCATGATGAAACCGGCAAGGTGATTGTCGAGCAAGGTCGTCGTATTACCGCTCGCCACATCAACCAGCTGGAAAA
GGCCGGCGTGAAGCAGCTCGACGTTCCTATGGAATACGTCCTGGGCCGCACCACCGCCAAGGCCATCGTGCACCCGGCTACCGGC

GAAATCCTGGCCGAGTGCAACACCGAGCTGAACACCGAGCTGCTGATCAAGATCGCCAAGGCTCAGGTTGTCCGCATCGAGACCC
TGTACACCAACGACATCGACTGCGGTCCGTTCATCTCCGATACCCTGAAGATCGACTCCACCAGCAATCAGCTGGAAGCGCTGGT
CGAAATCTACCGGATGATGCGTCCTGGCGAGCCGCCAACCAAGGATGCTGCCGAGACCCTGTTCAACAACCTGTTCTTCAGCGCC
GAGCGTTACGACCTGTCGGCCGTTGGTCGCATGAAGTTCAACCGTCGTATCGGTCGCACCGAGATCGAAGGTTCGGGCGTGCTGA
GCAAGGAAGACATCGTCGAGGTCCTCAAGACCCTGGTCGATATCCGTAACGGCAAAGGCATCGTCGACGACATCGACCACCTCG
GTAACCGTCGCGTTCGTTGCGTCGGCGAGATGGCCGAGAACCAGTTCCGCGTTGGCCTGGTGCGTGTTGAGCGTGCGGTCAAAGA
GCGTCTGTCGATGGCAGAAAGCGAAGGCCTGATGCCTCAGGACCTGATCAACGCCAAGCCGGTTGCGGCGGCGGTGAAAGAGTT
CTTCGGCTCCAGCCAGCTCTCGCAGTTCATGGACCAGAACAACCCGCTCTCCGAGATCACCCACAAGCGTCGTGTCTCCGCACTCG
GCCCTGGTGGTCTGACCCGTGAGCGTGCAGGCTTCGAAGTTCGTGACGTACACCCGACCCACTACGGCCGTGTGTGCCCGATCGA
GACTCCTGAAGGTCCGAACATCGGTCTGATCAACTCCCTGGCGGCCTATGCCCGCACCAACCAGTACGGCTTCCTGGAAAGCCCG
TACCGTGTGGTTAAAGAGGGTGTTGTCACCGACGACATCGTGTTCCTGTCGGCGATCGAAGAAGCTGATCACGTCATCGCCCAGG
CTTCGGCCGCGATGAACGAGAAGAAGCAACTGATCGACGAGCTGGTAGCCGTTCGTCACCTGAACGAATTCACCGTCAAGGCGCC
GGAAGACGTCACCCTGATGGATGTTTCGCCGAAGCAGGTAGTTTCGGTTGCAGCGTCGCTGATTCCGTTCCTCGAGCACGACGAC
GCCAACCGTGCGTTGATGGGTTCGAACATGCAGCGTCAGGCTGTACCAACCCTGCGTGCCGACAAGCCGCTGGTCGGTACCGGCA
TGGAGCGTAACGTTGCCCGTGACTCCGGCGTTTGCGTCGTGGCTCGTCGTGGCGGCGTGATCGATTCCGTCGATGCCAGCCGTATC
GTTGTTCGCGTTGCTGATGACGAAGTTGAAACCGGTGAAGCCGGTGTCGACATCTACAACCTGACCAAGTACACCCGTTCGAACC
AGAACACCTGCATCAACCAGCGTCCGCTGGTGAGCAAAGGTGATGTGGTTCAGCGTAGCGACATCATGGCCGACGGCCCGTCCAC
CGACATGGGTGAACTGGCACTGGGTCAGAACATGCGTATCGCGTTCATGGCGTGGAACGGCTTCAACTTCGAAGACTCCATCTGC
CTGTCCGAGCGTGTGGTTCAGGAAGACCGCTTCACCACGATCCACATCCAGGAACTGACCTGTGTGGCCCGTGACACCAAGCTTG
GCCCAGAAGAGATCACCGCGGACATTCCGAACGTCGGTGAAGCTGCGCTGAACAAGCTGGACGAAGCCGGTATTGTTTACGTGG
GTGCTGAAGTCGGCGCTGGCGACATTCTGGTCGGCAAGGTCACGCCAAAAGGCGAAACCCAGCTGACTCCAGAAGAGAAGCTGC
TGCGCGCGATCTTCGGTGAGAAGGCCAGCGACGTTAAAGACACCTCCCTGCGCGTGCCTACCGGCACCAAAGGTACTGTCATCGA
CGTACAGGTCTTCACCCGTGACGGCGTTGAGCGCGACAGCCGTGCGCTGTCCATCGAGAAGATGCAGCTCGACGAGATCCGCAAG
GACCTGAACGAAGAGTTCCGTATCGTTGAAGGCGCAACCTTCGAACGTCTGCGTTCTGCCCTGAACGGCCAGGTCGTCGATGGTG
GTGCAGGCCTGAAGAAAGGCACTGTGATCACCAACGAAGTGCTGGACGGTCTGGAGCACGGCCAGTGGTTCAAACTGCGCATGG
CCGAAGACGCGCTGAACGAGCAACTCGAGAAGGCCCAGGCCTACATCGTTGATCGTCGCCGTCTGCTGGACGACAAGTTCGAAG
ACAAGAAGCGCAAGCTGCAGCAGGGCGATGACCTGGCACCGGGCGTACTGAAGATCGTCAAGGTCTACCTGGCAATCCGTCGAC
GCATCCAGCCGGGCGACAAGATGGCCGGTCGTCACGGTAACAAGGGTGTGGTCTCGGTGATCATGCCGGTCGAAGACATGCCGC
ACGACGCCAACGGTACTCCGGTTGACGTGGTACTCAACCCACTGGGTGTACCATCGCGTATGAACGTTGGTCAGATCCTCGAAAC
CCACCTGGGCCTGGCGGCCAAAGGTCTGGGTGAGAAGATCAACCGCATGCTCGAAGAGCAGCGCAAGGTCATTGAACTGCGCAA
GTTCCTCACCGAGATCTACAACGAGATCGGTGGTCGTCAGGAAAGCCTGGAAGACTTCTCCGACCAGGAAATCCTGGATCTGGCG
AAGAACCTCAAAGGCGGTGTGCCAATGGCCACTCCAGTCTTCGACGGCGCCAAGGAAAGCGAAATCAAGGCCATGCTGAAACTG
GCAGACATGCCAGAAAGCGGTCAGATGCAGCTGTTCGACGGCCGTACCGGTAACAAGTTCGAGCGTCCAGTGACCGTTGGTTACA
TGTACATGCTCAAGCTGAACCACTTGGTGGACGACAAGATGCACGCGCGTTCCACTGGTTCTTACAGCCTGGTTACCCAGCAGCC
GCTGGGTGGTAAGGCGCAGTTCGGTGGTCAGCGTTTCGGGGAGATGGAAGTGTGGGCGCTGGAAGCATACGGCGCGGCATACAC
CCTGCAAGAAATGCTCACAGTGAAGTCGGACGACGTGAACGGTCGGACCAAGATGTACAAAAACATCGTGGACGGCGATCACCG
TATGGAGCCGGGCATGCCCGAGTCCTTCAACGTGTTGATCAAAGAGATCCGTTCGCTCGGTATCGATATCGATCTGGAAACCGAA
TAA

P. amygdali pv. tabaci str. ATCC 11528_GCF_000145945.2_CP042804.1

ATGGCTTACTCATATACTGAGAAAAAACGTATCCGCAAGGACTTTAGCAAGTTGCCGGACGTAATGGATGTGCCGTATCTCTTGG
CCATCCAGCTGGATTCGTATCGCGAATTCCTGCAGGCGGGAGCGACCAAAGATCAGTTCCGCGACGTCGGTCTGCATGCAGCCTT
CAAATCCGTTTTCCCGATCATCAGCTACTCCGGCAATGCTGCGCTGGAGTATGTAGGTTATCGCTTGGGCGAACCGGCATTTGATG
TCAAGGAATGCGTGCTGCGCGGTGTGACTTACGCAGTACCTCTGCGGGTCAAGGTCCGTCTGATCATTTTCGACAAAGAATCGTCG
AACAAAGCGATCAAGGACATCAAAGAGCAAGAAGTCTACATGGGTGAAATCCCCCTGATGACTGAAAACGGTACCTTCGTAATC
AATGGCACCGAGCGCGTTATCGTGTCTCAGCTTCACCGCTCGCCTGGCGTATTCTTCGACCACGACCGTGGCAAGACGCACAGCTC
CGGTAAGCTGCTTTACTCCGCTCGTATCATTCCTTACCGCGGTTCGTGGCTGGACTTCGAGTTCGATCCGAAAGACTGCGTATTCGT
CCGTATCGACCGTCGTCGCAAGCTGCCTGCATCCGTACTTCTGCGCGCTCTGGGTTATACCACCGAGCAAGTGCTCGATGCTTTCT
ACACCACCAACGTATTCCATGTGCGCGGCGAAAACCTGAGCCTGGAACTGGTGCCTCAGCGCCTGCGTGGTGAAATTGCCGTTCT
GGATATTCTGGACGACAAGGGCAAGGTCATTGTCGAGCAGGGTCGCCGTATTACCGCTCGTCACATCAACCAGCTGGAAAAAGCC
GGGATCAAAGAGCTGGAAGTACCTCTGGACTACGTCCTGGGTCGTACTACTGCCAAGGTCATCGTGCATCCGGCAACCGGTGAGA
TCATTGCCGAGTGCAACACCGAGCTGAACACTGAAATCCTGGCCAAGATCGCCAAGGCTCAGGTCGTTCGCATCGAAACGTTGTA
CACCAACGACATCGACTGCGGTCCGTTCGTCTCTGACACGCTGAAGATCGACTCCACCAGCAACCAACTGGAAGCGCTGGTCGAG
ATCTATCGCATGATGCGTCCTGGCGAGCCGCCAACCAAGGATGCCGCTGAAACACTGTTCAACAACCTGTTCTTCAGTCCTGAGC
GTTACGACCTGTCTGCTGTAGGCCGGATGAAGTTCAACCGTCGTATCGGTCGTACCGAAATCGAAGGCTCGGGCGTGCTGTGCAA
GGAAGACATCGTAGCGGTCCTCAAGACCCTCGTTGACATCCGTAACGGCAAAGGCATCGTCGATGACATCGACCACCTCGGTAAC
CGTCGCGTTCGTTGCGTTGGTGAGATGGCCGAGAACCAGTTCCGTGTAGGTCTGGTCCGTGTCGAGCGCGCTGTCAAAGAGCGCC
TGTCCATGGCAGAAAGCGAAGGCCTGATGCCTCAGGACCTGATCAACGCCAAGCCTGTTGCGGCGGCGGTCAAGGAATTCTTCGG
TTCCAGCCAGCTTTCCCAGTTCATGGGCCAGAACAACCCGCTGTCCGAGATCACTCACAAGCGCCGTGTTTCTGCACTCGGCCCTG
GCGGTCTGACGCGTGAGCGTGCTGGCTTTGAAGTTCGAGACGTTCACCCGACTCACTACGGCCGTGTTTGCCCGATCGAGACGCCT
GAAGGTCCGAACATCGGTCTGATCAACTCCTTGGCGGCTTATGCTCGCACCAACCAGTACGGCTTCCTTGAGAGCCCGTACCGTGT
GGTCAAGGAAGGTCTGGTAACCGAAGAAATCGTTTTCCTTTCGGCGATCGAAGAAGCGGACCACGTCATTGCTCAGGCTTCGGCT
GCAATGAACGACAAGCAAGAGCTGATCGACGAGCTGGTTGCTGTGCGTCACTTGAACGAATTCACCGTCAAGGCACCAGCCGAT
GTCACCCTGATGGACGTTTCGCCCAAGCAGGTTGTGTCGGTAGCCGCATCGCTGATCCCGTTCCTCGAGCACGATGACGCCAACC

GCGCATTGATGGGTTCGAACATGCAGCGTCAGGCTGTACCGACCCTGCGTGCCGACAAGCCGCTGGTAGGTACTGGCATGGAGCG
CAACGTTGCTCGCGACTCCGGCGTTTGCGTCGTGGCTCGTCGTGGCGGCGTGATCGACTCTGTCGACGCCAGCCGTATCGTTGTTC
GCGTTGCCGATGACGAAGTAGAAACAGGTGAGGCAGGTGTAGACATCTACAACCTGACCAAATACACCCGTTCGAACCAGAACA
CCTGCATCAACCAGCGTCCGCTGGTCAGCAAGGGTGACCGTGTTCAGCGTAGCGACATCATGGCCGACGGTCCGTCCACCGATAT
GGGTGAGCTGGCTCTGGGTCAGAACATGCGCATCGCGTTCATGGCCTGGAACGGTTTCAACTTCGAAGACTCCATCTGCCTCTCCG
AGCGTGTAGTTCAAGAAGATCGCTTCACAACGATCCACATTCAGGAACTGACCTGTGTGGCTCGTGACACCAAGCTTGGGCCTGA
AGAGATCACCGCCGACATCCCGAACGTGGGTGAAGCGGCTCTGAACAAGCTGGACGAAGCCGGTATCGTTTATGTAGGTGCCGA
GGTTGGCGCCGGCGACATCCTGGTGGGCAAGGTCACTCCGAAAGGCGAGACCCAGCTGACTCCGGAAGAAAAACTGTTGCGCGC
GATTTTCGGTGAGAAAGCCAGCGACGTTAAAGATACTTCCCTGCGCGTGCCGACCGGCACCAAGGGTACTGTCATTGACGTTCAG
GTCTTCACCCGTGATGGCGTCGAGCGTGATGCGCGTGCCCTGTCCATCGAGAAGTCGCAACTCGACGAGATCCGTAAGGATCTGA
ACGAAGAGTTCCGTATTGTCGAAGGCGCTACTTTCGAACGTCTGCGCTCTGCGCTGGTGGGTCGTGTAGCCGAAGGCGGTGCTGG
CCTCAAGAAAGGTCAGGAAATCACCAACGAAGTGCTTGATGGTCTTGAGCATGGTCAGTGGTTCAAACTGCGCATGGCTGAAGAT
GCTCTGAATGAGCAGCTTGAGAAAGCTCAGGCCTACATCGTTGATCGTCGCCGTCTTCTGGATGACAAGTTCGAAGACAAGAAAC
GCAAACTGCAGCAAGGCGATGACCTGGCTCCAGGCGTTCTGAAAATCGTCAAGGTCTACCTGGCAATCCGTCGTCGCATCCAGCC
GGGCGACAAGATGGCCGGTCGTCACGGTAACAAGGGTGTGGTCTCCGTGATCATGCCGGTTGAAGACATGCCGCACGATGCCAAT
GGCACGCCGGTCGATATCGTCCTCAACCCGCTGGGCGTACCTTCGCGTATGAACGTTGGTCAGATTCTCGAAACTCACCTGGGCCT
CGCGGCCAAAGGTCTGGGCGAGAAGATCAACCGCATGCTTGAAGAGCAGCGTAAAGTTGCTGAGCTGCGTAAGTTCCTCAACGA
GATCTATAACGAAATCGGCGGTCGTCAGGAGTCTCTGGAAGACCTCACCGACAACGAGATTCTGGACCTCGCGAAGAACCTGCGT
AACGGCGTACCGATGGCTACCCCGGTTTTCGACGGTGCCAAGGAAAGCGAAATCAAGGCAATGCTCAAGCTGGCAGATATGCCG
GAAAGCGGCCAGATGCAGCTGTTCGACGGTCGTACCGGCAACAAGTTTGAACGTGCTGTAACGGTTGGCTACATGTACATGCTGA
AGCTGAACCACTTGGTGGACGACAAGATGCACGCGCGTTCCACTGGTTCGTACAGCCTGGTTACCCAGCAGCCGCTGGGTGGTAA
GGCACAGTTCGGTGGTCAGCGTTTCGGGGAGATGGAAGTGTGGGCGCTGGAAGCATACGGCGCGGCGTACACTCTGCAGGAAAT
GCTCACAGTGAAGTCGGACGATGTGAACGGTCGTACCAAGATGTACAAAAACATCGTGGACGGCGATCACCGTATGGAGCCGGG
CATGCCCGAGTCTTTCAACGTGTTGATCAAAGAAATCCGTTCGCTCGGTATCGATATCGATCTGGAAACCGAATAA

P. atacamensis SM1_GCF_017167965.1_CP070503.1

ATGGCTTACTCATATACTGAGAAAAAACGTATCCGCAAGGACTTTAGCAAGTTGCCGGACGTCATGGATGTGCCTTACCTCCTGGC
CATCCAGCTGGATTCGTATCGTGAATTCTTGCAAGCGGGAGCGACTAAAGATCAGTTCCGCGACGTGGGCCTGCATGCGGCCTTC
AAATCCGTTTTCCCGATCATCAGCTACTCCGGCAATGCTGCGCTGGAGTACGTCGGTTATCGCCTGGGCGAACCGGCATTTGATGT
CAAAGAATGCGTATTGCGCGGTGTGACTTTCGCCGTACCTTTGCGGGTAAAAGTGCGCCTGATCATTTTCGACAAAGAATCGTCGA
ACAAAGCGATCAAGGACATCAAAGAGCAAGAAGTCTACATGGGTGAAATCCCCCTGATGACTGAGAACGGTACCTTCGTAATCA
ACGGTACCGAGCGTGTTATCGTTTCCCAGCTGCACCGTTCCCCGGGCGTGTTCTTCGACCACGACCGCGGCAAGACGCACAGCTCC
GGCAAACTGCTGTACTCGGCTCGCATCATTCCTTACCGCGGTTCGTGGCTGGACTTCGAATTCGACCCGAAAGACTGCGTCTTCGT
GCGTATCGACCGTCGTCGCAAACTGCCGGCTTCGGTACTGCTGCGCGCACTCGGTTACACCACCGAAGAAGTGCTGGACGCGTTC
TACACCACCAACGTATTCCACCTGAGCGGCGAAACCCTCAGCCTGGAACTGGTGCCATCGCGCCTGCGTGGTGAAGTTGCGGTTC
TGGACATCCAGGACGAGAAGGGCAAGGTCATCGTTGAGCAAGGCCGCCGTATTACCGCGCGCCACATCAACCAGATCGAAAAAG
CCGGTATCAAGACGCTGGACGTGCCACTCGACTACGTCCTTGGCCGCACCACCGCCAAGGCTATCGTGCACCCGGCTACCGGAGA
AATCCTGGCCGAATGCAACACCGAGCTGAACACTGAAGTCCTGGCAAAAATCGCCAAGGCTCAGGTCGTTCGCATCGAGACCCTG
TACACCAACGACATCGACTGCGGTCCGTTCGTCTCCGACACGCTGAAGATCGACTCCACCAGCAACCAATTGGAAGCGCTGGTCG
AGATCTATCGCATGATGCGTCCAGGCGAGCCGCCAACCAAAGACGCTGCCGAAACCCTGTTCAACAACCTGTTCTTCAGCCCTGA
GCGCTATGACCTGTCTGCGGTCGGCCGGATGAAGTTCAACCGTCGTATCGGTCGTACCGAGATCGAAGGTTCGGGCGTGCTGTGC
AAGGAAGACATCGTCGCGGTCCTGAAGACTCTGGTCGACATCCGCAACGGTAAAGGCATCGTCGATGACATCGACCACCTGGGTA
ACCGTCGTGTTCGCTGCGTAGGCGAAATGGCCGAGAACCAGTTCCGCGTTGGCCTGGTACGTGTTGAGCGTGCGGTCAAAGAGCG
TCTGTCGATGGCAGAAAGCGAAGGCCTGATGCCGCAAGACCTGATCAACGCCAAGCCAGTGGCTGCGGCGGTGAAAGAGTTCTT
CGGTTCCAGCCAGCTGTCGCAGTTCATGGACCAGAACAACCCGCTGTCCGAGATCACCCACAAGCGTCGTGTCTCTGCACTCGGC
CCTGGCGGTCTGACTCGTGAGCGCGCAGGCTTCGAAGTCCGTGACGTACACCCGACTCACTACGGTCGTGTCTGCCCGATTGAAA
CGCCGGAAGGTCCGAACATCGGCCTGATCAACTCCCTGGCTGCGTACGCTCGCACCAACCAGTACGGCTTCCTGGAAAGCCCGTA
CCGCGTGGTGAAAGAGGGTGTAGTCACCGACGAGATCGTGTTCCTGTCCGCTATTGAAGAAGCCGATCACGTGATCGCGCAGGCT
TCGGCGACCATGAACGAGCAGAAAGTCCTGATCGACGAACTGGTTGCCGTACGTCACCTGAACGAATTCACCGTCAAGGCGCCGG
AAGACGTCACCCTGATGGACGTTTCGCCGAAGCAGGTTGTGTCGGTTGCAGCGTCGCTGATTCCGTTCCTCGAGCACGACGACGC
CAACCGTGCGTTGATGGGTTCGAACATGCAGCGTCAGGCTGTACCGACTCTGCGCGCTGACAAGCCGCTGGTAGGTACCGGCATG
GAGCGTAACGTAGCCCGTGACTCCGGCGTTTGCGTCGTGGCTCGTCGTGGTGGCGTGATCGATTCCGTCGATGCCAGCCGTATCGT
GGTTCGTGTTGCCGATGACGAAGTTGAAACCGGTGAAGCCGGTGTCGACATCTACAACCTGACCAAGTACACCCGCTCGAACCAG
AACACCTGCATCAACCAGCGTCCGCTGGTGCGTAAAGGTGATCGCGTTCAGCGTAGCGACATCATGGCCGACGGCCCGTCCACCG
ACATGGGTGAGCTGGCGCTGGGTCAGAACATGCGCATCGCGTTCATGGCATGGAACGGCTTCAACTTCGAAGACTCCATCTGCCT
GTCCGAGCGTGTGGTTCAGGAAGACCGTTTCACCACGATCCACATTCAGGAACTGACCTGTGTGGCCCGTGACACCAAGCTTGGC
CCAGAGGAAATCACTGCGGACATCCCGAACGTGGGTGAAGCTGCACTGAACAAGCTGGACGAAGCCGGTATCGTTTACGTAGGT
GCTGAAGTAGGCGCAGGCGACATCCTGGTTGGCAAGGTCACTCCCAAAGGCGAGACCCAACTGACTCCGGAAGAAAAACTGCTG
CGTGCAATCTTCGGTGAAAAAGCCAGCGACGTTAAAGACACATCCCTGCGCGTGCCAACCGGCACCAAAGGTACTGTCATCGACG
TACAGGTCTTCACCCGTGACGGCGTTGAGCGTGATGCTCGTGCACTGTCGATCGAGAAGTCCCAGCTGGACGAGATCCGCAAGGA
TCTGAACGAAGAGTTCCGCATCGTTGAAGGCGCGACTTTCGAGCGTCTGCGTTCCGCACTGGTAGGCCACAAGGCCGAAGGCGGC
GCCGGCCTGAAGAAGGGGCAGGACATCACCGACGAAGTACTCGACGGTCTTGAGCACGGCCAGTGGTTCAAACTGCGCATGGCT
GAAGATGCGCTGAACGAGCAGCTCGAGAAGGCTCAGGCCTACATCGTTGATCGTCGCCGTCTGCTGGACGACAAGTTCGAAGAC
AAGAAGCGCAAACTGCAGCAGGGCGATGACCTGGCTCCAGGCGTGCTGAAAATCGTCAAGGTTTACCTGGCAATCCGTCGCCGC

ATCCAGCCGGGCGACAAGATGGCCGGTCGTCACGGTAACAAGGGTGTGGTCTCCGTGATCATGCCGGTTGAAGACATGCCGCACG
ATGCCAATGGCACCCCGGTCGACGTGGTCCTCAACCCGCTGGGCGTACCTTCGCGTATGAACGTTGGTCAGATCCTCGAAACCCA
CCTGGGCCTCGCGGCCAAAGGTCTGGGCGAGAAGATCAACCGGATGGTCGAAGAGCAGCGTAAAGTCGCTGAACTGCGCACCTT
CCTGGACGAGATCTACAACCAGATCGGCGGTCGTAACGAAGATCTGGACAGCTTCTCCGATCAGGAAATCCTCGATCTGGCGAAC
AACCTGCGTGGCGGCGTTCCAATGGCCACTCCAGTGTTCGACGGCGCCAAGGAAAGCGAAATCAAGGCCATGCTGAAACTGGCA
GACCTGCCGGAAAGCGGCCAGATGCAGCTGACCGACGGCCGTACCGGCAACAAGTTCGAGCGTCCAGTTACCGTTGGCTACATGT
ACATGCTGAAGCTGAACCACTTGGTAGACGACAAGATGCACGCGCGTTCTACCGGTTCTTACAGCCTGGTTACCCAGCAGCCGCT
GGGTGGTAAGGCGCAGTTCGGTGGTCAGCGTTTCGGGGAGATGGAGGTCTGGGCACTGGAAGCATACGGTGCTGCTTACACTCTG
CAAGAAATGCTCACAGTGAAGTCGGACGATGTGAACGGCCGTACCAAGATGTACAAGAACATCGTGGATGGCGATCACCGTATG
GAGCCGGGCATGCCCGAGTCCTTCAACGTGTTGATCAAGGAAATTCGTTCCCTCGGCATCGATATCGATCTGGAAACCGAATAA

P. bijieensis L22-9_GCF_013347965.1_CP048810.1

ATGGCTTACTCATATACTGAGAAAAAACGTATCCGCAAGGACTTTAGCAAGTTGCCGGACGTCATGGATGTGCCGTACCTCCTGG
CCATCCAGCTGGATTCGTATCGTGAATTCTTGCAAGCGGGAGCGACTAAAGATCAGTTCCGCGACGTGGGCCTGCATGCGGCCTT
CAAATCCGTTTTCCCGATCATCAGCTACTCCGGCAATGCTGCGCTGGAGTACGTCGGTTATCGCCTGGGCGAACCGGCATTTGATG
TCAAAGAATGCGTATTGCGCGGTGTTACTTACGCCGTACCTTTGCGGGTAAAAGTGCGCCTGATCATTTTCGACAAAGAATCGTCG
AACAAAGCGATCAAGGACATCAAAGAGCAAGAAGTCTACATGGGTGAAATCCCCCTGATGACTGAGAACGGTACCTTCGTAATC
AACGGTACCGAGCGTGTAATCGTTTCCCAGCTGCACCGTTCTCCAGGCGTGTTCTTCGACCACGACCGTGGCAAGACGCACAGCT
CCGGCAAACTGCTGTACTCCGCGCATCATTCCTTACCGCGGTTCGTGGCTGGACTTCGAGTTCGACCCGAAAGACTGCGTATTC
GTGCGTATCGACCGTCGTCGCAAGCTGCCTGCATCGGTACTGCTGCGCGCGCTCGGCTATACCACCGAAGAAGTGCTGGACGCGT
TCTACACCACCAACGTCTTCCACGTGCAAGGTGAAAACCTCAGCCTGGAACTGGTGCCTCAGCGCCTGCGCGGTGAAATCGCTGT
CCTCGATATCCAGGATGACAAAGGCAAGGTTATTGTCGAGCAGGGTCGCCGTATCACCGCTCGCCACATCAACCAGCTGGAAAAA
GCCGGGATCAAAGAGCTGCAGGTGCCGATCGACTACGTCCTGGGTCGCACCACGGCCAAGGTCATCGTGCATCCGGCCACTGGCG
AAATCCTGGCAGAGTGCAATACCGAGCTGACCACCGAGATCCTGGCGAAAATCGCCAAGGCCCAGGTCGTTCGTATCGAAACGTT
GTACACCAACGACATCGACTGCGGTCCGTTCATCTCCGACACGCTGAAGATCGACTCCACCGGCAACCAGCTGGAAGCCCTGGTC
GAAATCTATCGCATGATGCGTCCTGGCGAGCCGCCAACCAAGGATGCAGCCGAGACCCTTTTCAACAACCTGTTCTTCAGCCCTG
AGCGCTATGACCTGTCTGCGGTCGGCCGGATGAAGTTCAACCGTCGTATCGGTCGTACCGAGATCGAAGGTTCGGGTGTTCTGAA
CAAGGACGACATCGTTGCGGTCCTCAAGACCCTGGTCGACATCCGTAACGGCAAAGGCATCGTCGATGACATCGACCACCTGGGT
AACCGTCGTGTTCGCTGTGTAGGCGAAATGGCCGAGAACCAGTTCCGTGTTGGCCTGGTGCGCGTAGAGCGTGCGGTCAAGGAAC
GCCTGTCGATGGCTGAAAGCGAAGGCCTGATGCCGCAAGACCTGATCAACGCCAAGCCTGTGGCTGCGGCGGTGAAAGAGTTCTT
CGGTTCGAGCCAGCTGTCCCAGTTCATGGACCAGAACAACCCGCTGTCCGAGATCACCCACAAGCGTCGTGTTTCCGCACTGGGC
CCAGGCGGTCTGACCCGTGAGCGTGCAGGCTTCGAAGTTCGTGACGTGCACCCGACGCACTACGGTCGTGTATGCCCGATCGAAA
CGCCGGAAGGTCCGAACATCGGTCTGATCAACTCCCTGGCTGCCTATGCGCGCACCAACCAGTACGGTTTCCTCGAGAGCCCGTA
TCGTGTGGTGAAAGACGCCCTGGTCACCGACGAGATCGTGTTCCTGTCCGCCATCGAAGAAGCCGATCACGTGATCGCCCAGGCT
TCGGCCACGATGAACGACAAGAAAGTCCTGACCGATGAATTGGTAGCTGTTCGTCACCTGAACGAGTTCACCGTCAAGGCGCCGG
AAGACGTCACCTTGATGGACGTATCGCCGAAGCAGGTAGTTTCGGTTGCAGCGTCGCTGATCCCGTTCCTCGAGCACGACGACGC
CAACCGTGCGTTGATGGGTTCGAACATGCAGCGTCAAGCTGTACCGACCCTGCGTGCCGACAAGCCGCTGGTAGGTACCGGCATG
GAGCGTAACGTTGCCCGTGACTCCGGCGTTTGCGTCGTGGCTCGTCGTGGTGGCGTGATCGACTCCGTCGACGCCAGCCGTATCGT
GGTTCGTGTTGCCGATGACGAAGTTGAAACTGGCGAAGCCGGTGTCGACATCTACAACCTGACCAAATACACCCGCTCCAACCAG
AACACCTGCATCAACCAGCGTCCGCTGGTAAGCAAGGGTGATCGGGTTCAGCGTAGCGACATCATGGCCGACGGTCCGTCCACCG
ATATGGGTGAACTGGCGTTGGGTCAGAACATGCGCATCGCGTTCATGGCATGGAACGGCTTCAACTTCGAAGACTCCATCTGCCT
GTCCGAGCGTGTGGTCCAGGAAGACCGTTTCACCACGATCCACATCCAGGAACTGACCTGTGTGGCCCGTGACACCAAGCTTGGC
CCAGAGGAAATCACTGCGGACATCCCGAACGTGGGTGAAGCTGCACTGAACAAACTGGACGAAGCCGGTATCGTTTACGTAGGT
GCTGAAGTAGGCGCAGGCGACATCCTGGTGGGCAAGGTCACTCCGAAAGGCGAGACCCAGCTGACTCCGGAAGAAAAACTGCTG
CGTGCCATTTTCGGTGAAAAAGCCAGCGACGTTAAAGACACCTCCCTGCGCGTGCCTACTGGCACCAAGGGTACCGTCATCGACG
TACAGGTCTTCACCCGTGACGGCGTCGAGCGTGATGCTCGTGCACTGTCGATCGAGAAGACTCAACTCGACGAGATCCGCAAGGA
CCTGAACGAAGAGTTCCGTATCGTCGAAGGCGCAACTTTCGAGCGTCTGCGTTCCGCCCTGGTCGGCCACAAAGCCGAAGGCGGC
GCCGGCCTGAAGAAAGGTCAGGAAATCACCGACGAAGTCCTCGACGGTCTTGAGCATGGCCAGTGGTTCAAACTGCGCATGGCT
GAAGATGCTCTGAACGAGCAGCTCGAGAAGGCCCAGGCCTATATCGTTGATCGCCGCCGTCTGCTGGACGACAAGTTCGAAGACA
AGAAGCGCAAACTGCAGCAGGGCGATGACCTGGCTCCAGGCGTGCTGAAAATCGTCAAGGTTTACCTGGCAATCCGTCGTCGCAT
CCAGCCGGGCGACAAGATGGCCGGTCGTCACGGTAACAAGGGTGTGGTCTCTGTGATCATGCCGGTTGAAGACATGCCGCACGAT
GCCAATGGCACCCCGGTCGACGTGGTCCTCAACCCATTGGGCGTACCTTCGCGTATGAACGTCGGTCAGATCCTTGAAACCCACCT
GGGCCTCGCGGCCAAGGGCTTGGGCGAGAAGATCAACCGTATGATCGAAGAGCAGCGCAAGGTTGCTGACCTGCGTAAGTTCCT
GCACGAGATCTACAACGAGATCGGCGGCCGCAACGAAGAGCTGGACACCTTCTCCGACCAGGAAATCCTGGATCTGGCGAAGAA
CCTGCGCGGTGGCGTTCCAATGGCTACTCCGGTGTTCGACGGTGCCAAGGAAAGCGAAATCAAGGCCATGCTGAAACTGGCAGAC
CTGCCGGAAAGCGGCCAGATGCAGCTGTTCGACGGCCGTACCGGCAACAAGTTCGAGCGCCCGGTTACCGTTGGCTACATGTACA
TGCTGAAGCTGAACCACTTGGTGGACGACAAGATGCACGCTCGTTCTACCGGTTCTTACAGCCTGGTTACCCAGCAACCGCTGGG
TGGTAAGGCGCAGTTCGGTGGTCAGCGTTTCGGGGAGATGGAGGTCTGGGCACTGGAAGCGTACGGTGCTGCATACACTCTGCAA
GAAATGCTCACAGTGAAGTCGGACGATGTGAACGGCCGGACCAAGATGTACAAAAACATCGTGGACGGCGATCACCGTATGGAG
CCGGGCATGCCCGAGTCTTTCAACGTGTTGATCAAGGAAATTCGTTCCCTCGGCATCGATATCGATCTGGAAACCGAATAA

P. brassicacearum 3Re2-7_GCF_008370715.1_CP034725.1

ATGGCTTACTCATATACTGAGAAAAAACGTATCCGCAAGGACTTTAGCAAGTTGCCGGACGTCATGGATGTGCCGTACCTCCTGG
CCATCCAGCTGGATTCGTATCGTGAATTCTTGCAAGCGGGAGCGACTAAAGATCAGTTCCGCGACGTGGGCCTGCATGCGGCCTT

CAAATCCGTTTTCCCGATCATCAGCTACTCCGGCAATGCTGCGCTGGAGTACGTCGGTTATCGCCTGGGCGAACCGGCATTTGATG
TCAAAGAATGCGTATTGCGCGGTGTTACTTACGCCGTACCTTTGCGGGTAAAAGTGCGCCTGATCATTTTCGACAAAGAATCGTCG
AACAAAGCGATCAAGGACATCAAAGAGCAAGAAGTCTACATGGGTGAAATCCCCCTGATGACTGAGAACGGTACCTTCGTAATC
AACGGTACCGAGCGTGTAATCGTTTCCCAGCTGCACCGTTCTCCGGGCGTGTTCTTCGACCACGACCGTGGCAAGACGCACAGCT
CCGGCAAACTGCTTTACTCCGCGCGCATCATTCCTTACCGCGGTTCGTGGTTGGACTTCGAGTTCGACCCGAAAGACTGCGTATTC
GTGCGTATCGACCGTCGTCGCAAGCTGCCTGCATCGGTACTGCTGCGCGCGCTCGGCTATACCACCGAAGAAGTGCTGGACGCGT
TCTACACCACCAACGTTTTCCATGTGCAGGGTGAAAACCTCAGCCTGGAACTGGTGCCTCAGCGCCTGCGCGGTGAAATCGCTGT
CCTCGATATCCAGGATGACAAAGGCAAGGTTATTGTCGAGCAGGGTCGCCGTATCACCGCTCGCCACATCAACCAGCTGGAAAAA
GCCGGGATCAAAGAGCTGCAGGTGCCTCTGGACTACGTCCTGGGTCGCACCACGGCCAAGGTCATCGTGCATCCGGCCACCGGCG
AAATCCTGGCAGAGTGCAACACCGAGCTGAACACCGAGATCCTGGCAAAAATCGCCAAGGCCCAGGTCGTTCGCATCGAGACGC
TGTACACCAACGACATCGACTGCGGTCCGTTCATCTCCGACACGCTGAAGATCGACTCCACCGGCAACCAACTGGAAGCCCTGGT
CGAGATCTATCGCATGATGCGTCCTGGCGAGCCGCCAACCAAGGATGCCGCCGAAACGCTGTTCAACAACCTGTTCTTCAGCCCT
GAGCGCTATGACCTGTCTGCGGTCGGCCGGATGAAGTTCAACCGTCGTATCGGTCGTACCGAAATCGAAGGTTCGGGTGTATTGA
ACAAGGACGATATCGTTGCCGTCCTCAAGACCTTGGTCGACATCCGTAACGGCAAGGGCATCGTCGATGACATCGACCACCTGGG
TAACCGTCGTGTTCGCTGTGTAGGCGAAATGGCCGAGAACCAGTTCCGTGTTGGCCTGGTGCGCGTAGAGCGTGCGGTCAAGGAA
CGTCTGTCGATGGCTGAAAGCGAAGGCCTGATGCCGCAAGACCTGATCAACGCCAAGCCTGTGGCTGCGGCGGTGAAGGAGTTCT
TCGGTTCGAGCCAGCTGTCCCAGTTCATGGACCAGAACAACCCGCTGTCCGAGATCACCCACAAGCGTCGTGTCTCCGCACTCGG
CCCGGGCGGTCTGACCCGTGAGCGTGCGGGCTTCGAAGTTCGTGACGTACACCCGACTCACTACGGTCGTGTATGCCCGATCGAA
ACGCCGGAAGGTCCGAACATCGGTCTGATCAACTCCCTGGCTGCCTATGCGCGCACCAACCAGTACGGCTTCCTCGAGAGCCCGT
ACCGTGTGGTGAAAGACGCCCTGGTCACCGACGAGATCGTGTTCCTGTCCGCCATCGAAGAAGCTGATCATGTGATCGCCCAGGC
TTCGGCCACGATGAACGACAAGAAGGTCCTGGTCGACGAGCTGGTAGCTGTTCGTCACTTGAACGAGTTCACCGTCAAGGCGCCG
GAAGACGTCACCTTGATGGACGTATCGCCGAAGCAGGTAGTTTCGGTTGCAGCGTCGCTGATCCCGTTCCTCGAGCACGACGACG
CCAACCGTGCGTTGATGGGTTCGAACATGCAGCGCCAAGCTGTACCCACCCTGCGTGCCGACAAGCCGCTGGTAGGTACCGGCAT
GGAGCGCAACGTTGCCCGTGACTCCGGCGTTTGCGTCGTGGCTCGTCGTGGTGGCGTGATCGACTCCGTCGATGCCAGCCGTATCG
TGGTTCGTGTTGCCGATGACGAAGTTGAAACTGGCGAAGCCGGTGTCGACATCTACAACCTGACCAAATACACCCGCTCCAACCA
GAACACCTGCATCAACCAGCGTCCGCTGGTGAGCAAGGGTGATCGGGTTCAGCGCAGCGACATCATGGCCGACGGTCCGTCCACT
GATATGGGTGAACTGGCGTTGGGTCAGAACATGCGCATCGCGTTTATGGCATGGAACGGCTTCAACTTCGAAGACTCCATCTGCC
TGTCCGAGCGTGTGGTTCAGGAAGACCGCTTCACCACGATCCACATCCAGGAACTGACCTGTGTGGCCCGTGACACCAAGCTTGG
CCCAGAGGAAATCACTGCGGACATCCCGAACGTGGGTGAAGCTGCACTGAACAAGCTGGACGAAGCCGGTATCGTTTACGTAGG
TGCTGAAGTAGGCGCAGGCGACATCCTGGTGGGCAAGGTCACTCCGAAAGGCGAGACCCAGCTGACTCCGGAAGAAAAACTGCT
GCGTGCCATCTTCGGTGAAAAAGCCAGCGACGTTAAAGACACCTCCCTGCGCGTGCCTACCGGCACCAAGGGTACCGTCATCGAC
GTACAAGTCTTCACTCGCGACGGCGTTGAGCGTGATGCTCGTGCACTGTCGATCGAGAAGACTCAACTCGACGAGATCCGCAAGG
ACCTGAACGAAGAGTTCCGTATCGTCGAAGGCGCAACTTTCGAGCGTCTGCGCTCCGCTCTGGTCGGCCACAAAGCCGAAGGCGG
CGCCGGCCTGAAGAAAGGTCAGGAAATCACCGACGAAGTTCTCGACGGTCTTGAGCATGGCCAGTGGTTCAAACTGCGCATGGCT
GAAGATGCTCTGAACGAGCAGCTCGAGAAGGCCCAGGCCTATATCGTTGATCGCCGCCGTCTGCTGGACGACAAGTTCGAAGACA
AGAAGCGCAAACTGCAGCAGGGCGATGACCTGGCTCCAGGCGTGCTGAAAATCGTCAAGGTTTACCTGGCAATCCGTCGTCGCAT
CCAACCGGGCGACAAGATGGCCGGTCGTCACGGTAACAAAGGTGTGGTCTCCGTGATCATGCCGGTTGAAGACATGCCGCACGAT
GCCAATGGCACCCCGGTCGATGTGGTCCTCAACCCGTTGGGCGTACCTTCGCGTATGAACGTCGGTCAGATCCTTGAAACCCACCT
GGGCCTCGCGGCCAAGGGCTTGGGCGAGAAGATCAACCGTATGATCGAAGAGCAGCGCAAGGTCGCTGACCTGCGTAAGTTCCT
GCACGAGATCTACAACGAGATCGGCGGTCGCAACGAAGAGCTGGACACCTTCTCCGATCAGGAAATCCTGGATCTGGCGAAGAA
CCTGCGCGGCGGCGTTCCAATGGCTACTCCGGTGTTCGACGGTGCCAAGGAAAGCGAAATCAAGGCCATGCTGAAACTGGCAGAT
CTGCCAGAAAGCGGCCAGATGCAGCTGTTCGACGGCCGTACCGGCAACAAGTTCGAGCGCCCGGTTACCGTTGGCTACATGTACA
TGCTGAAGCTGAACCACTTGGTAGACGACAAGATGCACGCTCGTTCTACCGGTTCTTACAGCCTGGTTACCCAGCAGCCGCTGGG
TGGTAAGGCGCAGTTCGGTGGTCAGCGTTTCGGGGAGATGGAGGTCTGGGCACTGGAAGCGTACGGTGCTGCATACACTCTGCAA
GAAATGCTCACAGTGAAGTCGGACGATGTGAACGGCCGGACCAAGATGTACAAAAACATCGTGGACGGCGATCACCGTATGGAG
CCGGGCATGCCCGAGTCTTTCAACGTGTTGATCAAGGAAATTCGTTCCCTCGGCATCGATATCGATCTGGAAACCGAATAA

P. cannabina pv. Alisalensis MAFF 301419_GCF_016599635.1_CP067022.1

ATGGCTTACTCATATACTGAGAAAAAACGTATCCGCAAGGACTTTAGCAAGTTGCCGGACGTAATGGATGTGCCGTATCTCTTGG
CCATTCAGCTGGATTCGTATCGCGAATTCCTGCAGGCGGGAGCGACCAAAGATCAGTTCCGCGACGTCGGTCTGCATGCAGCCTT
CAAATCCGTTTTCCCGATCATCAGCTACTCCGGCAATGCTGCGCTGGAGTATGTAGGTTATCGCTTGGGCGAACCGGCATTTGATG
TCAAGGAATGCGTGCTGCGCGGTGTGACTTACGCAGTACCTCTGCGGGTCAAGGTCCGTCTGATCATTTTCGACAAAGAATCGTCG
AACAAAGCGATCAAGGACATCAAAGAGCAAGAAGTCTACATGGGTGAAATCCCCCTGATGACTGAAAACGGTACCTTTGTAATC
AATGGCACCGAGCGCGTTATCGTGTCTCAGCTGCACCGTTCGCCAGGCGTATTCTTCGACCACGACCGTGGCAAGACGCACAGCT
CCGGTAAGCTGCTTTACTCCGCTCGTATCATTCCTTACCGCGGTTCGTGGCTGGACTTCGAGTTCGATCCGAAAGACTGCGTATTC
GTCCGTATCGACCGTCGTCGCAAGCTGCCTGCGTCTGTACTTCTGCGCGCGCTGGGTTACACCACCGAGCAAGTGCTCGATGCTTT
CTACACCACCAACGTCTTCCATGTGCGCGGCGAAAACCTGAGCCTGGAACTGGTGCCTCAGCGCCTGCGTGGTGAAATTGCCGTT
CTGGACATCCTGGACGACAAGGGCAAGGTCATTGTCGAGCAAGGTCGCCGTATCACTGCCCGTCACATCAACCAGCTGGAAAAG
GCCGGGATCAAAGAGCTGGAAGTACCTCTGGACTACGTCCTGGGCCGTACGACTGCCAAGGTCATCGTGCATCCGGCAACCGGCG
AGATCATTGCCGAGTGCAACACCGAGCTGAACACTGAAATCCTCGGCAAGATCGCCAAGGCTCAGGTTGTTCGCATCGAAACGCT
GTACACCAACGACATCGATTGCGGCCCGTTCGTTTCCGATACGTTGAAGATCGACTCCACCAGCAACCAACTGGAAGCGCTGGTC
GAGATATATCGCATGATGCGTCCTGGCGAGCCACCGACCAAGGATGCTGCTGAAACCCTGTTCAACAACCTGTTCTTCAGCCCAG
AGCGCTATGACCTGTCTGCTGTAGGCCGGATGAAGTTCAACCGTCGTATCGGTCGTACCGAAATCGAAGGTTCGGGCGTGTTGTG
CAAGGAAGATATCGTCGCGGTTCTCAAGACCCTCGTTGATATCCGTAACGGCAAAGGCATCGTCGATGACATCGACCACCTCGGT

AACCGTCGCGTTCGCTGCGTAGGCGAGATGGCCGAGAACCAGTTCCGTGTAGGTCTGGTCCGCGTCGAGCGTGCTGTCAAAGAAC
GTCTGTCCATGGCGGAAAGCGAAGGCCTGATGCCTCAGGACCTGATCAATGCCAAGCCTGTTGCGGCAGCGGTCAAAGAGTTCTT
CGGTTCCAGCCAGCTTTCCCAGTTCATGGACCAGAACAACCCGCTTTCCGAGATTACCCACAAGCGTCGTGTTTCTGCACTCGGCC
CTGGCGGTCTGACTCGTGAGCGTGCCGGCTTTGAAGTTCGCGACGTTCACCCGACTCACTATGGTCGTGTGTGCCCGATCGAAACG
CCGGAAGGTCCGAACATCGGTCTGATCAACTCCCTGGCGGCCTATGCCCGCACCAACCAGTACGGTTTCCTCGAGAGCCCGTACC
GTGTGGTGAAGGAAGGTCTGGTCACCGAAGAAATCGTGTTCCTTTCGGCGATCGAAGAAGCTGACCACGTCATTGCCCAGGCTTC
GGCTGCAATGAACGACAAGCAAGAGCTGATCGACGAGCTGGTTGCTGTGCGTCACTTGAACGAATTCACCGTCAAGGCGCCAGCC
GACGTCACCCTGATGGACGTTTCGCCCAAGCAGGTTGTCTCGGTAGCGGCTTCGCTGATCCCGTTCCTCGAGCACGATGACGCCAA
CCGTGCGTTGATGGGTTCGAACATGCAGCGTCAGGCTGTACCAACCTTGCGTGCCGACAAGCCGCTCGTGGGTACCGGCATGGAG
CGCAACGTTGCCCGCGATTCCGGCGTTTGCGTCGTGGCCCGTCGTGGTGGCGTGATCGATTCGGTTGATGCCAGCCGTATCGTTGT
TCGCGTCGCCGATGACGAAGTTGAAACAGGTGAGGCGGGTGTAGACATCTACAACCTGACCAAATACACCCGTTCGAACCAGAA
CACCTGCATCAACCAGCGTCCGCTGGTCAGCAAGGGTGATCGTGTTCAGCGCAGCGACATCATGGCCGACGGCCCGTCCACCGAT
ATGGGTGAGCTGGCACTGGGTCAGAACATGCGCATCGCGTTCATGGCCTGGAACGGTTTCAACTTCGAAGACTCCATCTGTCTTTC
CGAGCGCGTGGTTCAGGAAGATCGCTTCACCACAATCCACATTCAGGAACTGACCTGTGTGGCGCGTGACACCAAGCTTGGGCCT
GAGGAAATCACAGCTGACATCCCGAACGTGGGTGAAGCTGCTCTGAACAAGCTGGACGAAGCCGGTATCGTTTATGTAGGTGCTG
AAGTTGGTGCTGGCGACATCCTGGTGGGCAAGGTCACTCCGAAAGGCGAGACCCAGCTGACGCCGGAAGAAAAACTGTTGCGCG
CGATCTTCGGTGAGAAGGCCAGCGACGTTAAAGATACCTCCCTGCGCGTGCCAACCGGCACCAAAGGCACTGTCATTGACGTTCA
GGTCTTCACCCGTGATGGCGTGGAGCGTGATGCGCGTGCCCTGTCCATCGAGAAGTCGCAACTCGACGAGATCCGCAAGGACCTG
AACGAAGAGTTCCGTATTGTCGAAGGCGCTACCTTTGAACGTCTGCGCTCTGCGCTGGTGGGTCGTGTAGCAGAAGGCGGTGCCG
GCCTGAAGAAAGGCCAGGAAATCACCAATGAAGTGCTCGACGGTCTTGAGCATGGTCAGTGGTTCAAGCTGCGTATGGCTGAAG
ATGCTCTGAACGAGCAGCTTGAGAAAGCGCAGGCTTACATCGTTGATCGTCGTCGTCTTCTGGATGACAAGTTCGAAGACAAAAA
GCGCAAGCTGCAGCAGGGCGATGACCTGGCTCCAGGCGTGCTGAAAATCGTCAAGGTTTACCTGGCAATCCGTCGTCGCATTCAG
CCGGGCGACAAGATGGCCGGTCGTCACGGTAACAAGGGTGTGGTCTCCGTGATCATGCCGGTTGAAGACATGCCGCACGATGCCA
ATGGCACACCGGTAGATATCGTCCTCAACCCGTTGGGCGTACCTTCGCGTATGAACGTGGGCCAGATTCTCGAAACTCACCTTGGC
CTCGCCGCCAAAGGTCTGGGCGAGAAGATCAACCGCATGCTCGAAGAGCAGCGTAAAGTGGTTGAGCTGCGCAAGTTCCTGAAC
GAGATCTACAACGAGATCGGTGGTCGCCAGGAAAGTCTGGAAGACCTCTCCGACAAGGAGATCCTCGATCTTGCGAAGAACCTTC
GCAACGGTGTGCCAATGGCTACACCGGTGTTCGACGGTGCTAAAGAAAGCGAAATCAAGGCAATGCTCAAGCTGGCAGATATGC
CGGAAAGCGGCCAGATGCAGCTGTTTGACGGTCGTACTGGCAACAAGTTTGAGCGCCCGGTAACGGTCGGCTACATGTACATGCT
GAAGCTGAACCACTTGGTGGACGACAAGATGCACGCGCGTTCCACTGGTTCGTACAGCCTGGTTACCCAGCAGCCGCTGGGTGGT
AAGGCCCAGTTCGGTGGTCAGCGTTTCGGGGAGATGGAAGTGTGGGCGCTGGAAGCATACGGCGCGGCGTACACTCTGCAAGAA
ATGCTCACAGTGAAGTCGGACGATGTGAACGGTCGTACCAAGATGTACAAAAACATCGTGGACGGCGATCACCGTATGGAGCCG
GGCATGCCCGAGTCTTTCAACGTGTTGATCAAAGAAATCCGTTCGCTCGGTATCGATATCGATCTGGAAACCGAATAA

P. chlororaphis qlu-1_GCF_014524625.1_CP061079.1

ATGGCTTACTCATATACTGAGAAAAAACGTATCCGCAAGGACTTTAGCAAGTTGCCGGACGTCATGGATGTGCCGTACCTCCTGG
CCATCCAGCTGGATTCGTATCGTGAATTCTTGCAAGCGGGAGCGACTAAAGATCAGTTCCGCGACGTGGGCCTGCATGCGGCCTT
CAAATCCGTTTTCCCGATCATCAGCTACTCCGGCAATGCTGCGTTGGAGTATGTCGGTTATCGCCTGGGCGAACCGGCATTTGATG
TCAAAGAATGCGTGCTGCGCGGTGTAACTTACGCCGTACCTTTGCGGGTAAAAGTTCGTCTGATCATTTTCGACAAAGAATCGTCG
AACAAAGCGATCAAGGACATCAAAGAGCAAGAAGTCTACATGGGTGAAATCCCCCTGATGACTGAGAACGGTACCTTCGTAATC
AACGGTACCGAGCGTGTAATCGTTTCCCAACTGCACCGTTCCCCTGGCGTATTCTTCGACCACGACCGTGGCAAGACGCACAGCTC
CGGTAAGCTGCTGTACTCCGCACGTATCATTCCTTACCGCGGTTCCTGGTTGGACTTCGAGTTCGACCCGAAAGACTGCGTATTCG
TGCGTATCGACCGTCGTCGCAAGCTGCCTGCATCGGTACTGCTGCGTGCGTTGGGCTACACCACTGAAGAAGTGCTGGACGCTTTC
TACACCACCAACGTATTCCACGTGCGCGGCGAAAGCCTGAGCCTGGAGTTGGTGCCTCAGCGCCTGCGTGGTGAAATTGCGGTCC
TCGATATTCAGGATGACAAAGGCAAGGTTATTGTCGAGCAGGGTCGTCGTATTACTGCTCGCCACATCAACCAGCTGGAAAAAGC
CGGGATCAAAGAGCTGGAAGTGCCTCTGGACTACGTCCTGGGTCGTACTACCGCCAAGGTCATCGTGCATCCGGCCACCGGCGAA
ATCCTGGCAGAGTGCAACACCGAGCTGAACACCGAGATCCTGGCGAAGATCGCCAAGGCTCAGGTTGTGCGCATCGAAACGCTG
TACACCAACGATATCGATTGCGGTCCGTTCGTTTCCGACACTCTAAAGATCGACTCCACCACCAACCAACTGGAAGCGTTGGTCG
AGATCTATCGCATGATGCGTCCTGGCGAGCCTCCAACCAAGGATGCGGCCGAGACCCTGTTCAACAACCTGTTCTTCAGCCCTGA
GCGTTATGACCTGTCTGCGGTCGGCCGGATGAAGTTCAACCGTCGTATCGGTCGTACCGAGATCGAAGGTTCCGGCGTGTTGTGCA
AGGAAGACATCGTTGCGGTTCTGAAGACTCTGGTCGACATCCGTAACGGCAAAGGCATCGTCGATGACATCGACCACCTGGGTAA
CCGTCGTGTTCGCTGCGTAGGCGAGATGGCCGAGAACCAGTTCCGCGTTGGCCTGGTGCGTGTAGAGCGCGCGGTCAAAGAGCGT
CTGTCGATGGCTGAAAGCGAAGGCCTGATGCCGCAAGACCTGATCAACGCCAAGCCAGTGGCGGCGGCGGTGAAGGAGTTCTTC
GGTTCCAGCCAGCTGTCCCAGTTCATGGACCAGAACAACCCGCTGTCCGAGATCACCCACAAGCGCCGTGTCTCTGCACTCGGTC
CAGGCGGTCTGACTCGTGAGCGTGCAGGCTTCGAAGTTCGTGACGTACACCCGACTCACTATGGTCGTGTATGCCCGATCGAGAC
GCCGGAAGGTCCGAACATCGGTCTGATCAACTCCCTGGCCGCCTATGCTCGCACCAACCAGTACGGCTTCCTCGAGAGCCCGTAC
CGTGTGGTGAAAGAAGGTCTGGTGACCGACGAAATCGTGTTCCTGTCCGCTATCGAAGAGGCCGATCACGTGATCGCCCAGGCTT
CGGCGACCATGAACGACAAAGGTCAGCTGATCGACGAGCTGGTAGCTGTTCGTCACCTGAACGAATTCACCGTCAAGGCGCCAG
AAGACGTCACCTTGATGGACGTTTCGCCGAAGCAGGTAGTTTCGGTTGCGGCGTCGCTGATTCCGTTCCTCGAGCACGACGACGC
CAACCGTGCGTTGATGGGTTCGAACATGCAGCGTCAGGCTGTACCAACCCTGCGTGCCGACAAGCCGCTGGTAGGTACCGGCATG
GAGCGTAACGTTGCCCGTGACTCCGGCGTCTGCGTCGTGGCTCGTCGTGGCGGCGTGATCGACTCGGTTGATGCCAGCCGTATCGT
GGTTCGTGTTGCAGATGACGAAGTTGAGCCAGGCGAAGCCGGTGTCGACATCTACAACCTGACCAAATACACCCGCTCCAACCAG
AACACCTGCATCAACCAGCGTCCGCTGGTGAGCAAGGGTGATCGGGTTCAGCGCAGCGACATCATGGCCGACGGTCCGTCCACCG
ACATGGGTGAACTGGCTCTGGGTCAGAACATGCGCATCGCGTTCATGGCATGGAACGGCTTCAACTTCGAAGACTCCATCTGCCT
GTCCGAGCGTGTGGTCCAGGAAGACCGCTTCACCACGATCCACATCCAGGAACTGACCTGTGTGGCCCGTGACACCAAGCTTGGC

CCAGAGGAAATCACTGCGGACATCCCGAACGTGGGTGAAGCTGCACTGAACAAGCTGGACGAAGCCGGTATCGTTTATGTCGGTG
CTGAAGTAGGCGCAGGCGACATCCTGGTCGGTAAGGTCACTCCGAAAGGCGAGACCCAGCTGACTCCGGAAGAAAAACTGCTGC
GTGCAATCTTCGGTGAAAAAGCCAGCGACGTTAAAGACACCTCCCTGCGCGTACCTACCGGTACCAAGGGTACTGTCATCGACGT
ACAGGTCTTCACTCGTGACGGTGTAGAGCGTGACGCTCGTGCCCTGTCGATCGAGAAGAGCCAGCTCGACGAGATCCGCAAGGAT
CTGAACGAAGAGTTCCGTATCGTCGAAGGCGCCACTTTCGAACGTCTGCGTTCCGCTCTGGTCGGCCACAAAGCCGAAGGCGGCG
CCGGCCTGAAGAAAGGTCAGGAAATCACCGACGAAGTACTCGACGGTCTTGAGCATGGCCAGTGGTTCAAACTGCGCATGGCTG
AAGATGCTCTGAACGAGCAGCTCGAGAAGGCCCAGGCCTACATCGTTGATCGCCGCCGTCTGCTGGACGACAAGTTCGAAGACA
AGAAGCGCAAGCTGCAGCAGGGCGATGACCTGGCTCCAGGCGTGCTGAAAATCGTCAAGGTTTACCTGGCAATCCGTCGTCGCAT
CCAGCCGGGCGACAAGATGGCCGGTCGTCACGGTAACAAGGGTGTGGTCTCGGTGATCATGCCGGTTGAAGACATGCCGCACGA
TGCCAACGGCACCCCGGTCGACGTCGTCCTCAACCCGTTGGGCGTACCTTCGCGTATGAACGTTGGTCAGATTCTCGAAACTCACC
TGGGCCTCGCGGCCAAAGGTCTGGGCGAGAAGATCAACCGCATGGTCGAAGAGCAGCGCAAGGTCGCGGAACTGCGTAAGTTCC
TGGACGAGATCTACAACCAGATCGGTGGTCGTAACGAAGATCTGGATAGCTTCTCCGATCAGGAAATCCTGGATCTGGCGAAAAA
CCTGCGTGGCGGCGTGCCGATGGCCACTCCGGTGTTCGACGGTGCCAAGGAAAGCGAAATCAAGGCCATGCTGAAACTGGCGGA
CCTGCCAGAAAGCGGCCAGATGCAACTGACCGACGGCCGTACCGGCAACAAGTTCGAGCGCCCGGTTACTGTTGGCTACATGTAC
ATGCTGAAGCTGAACCACTTGGTAGACGACAAGATGCACGCTCGTTCTACCGGTTCGTACAGCCTGGTTACCCAGCAGCCGCTGG
GTGGTAAGGCGCAGTTCGGTGGTCAGCGTTTCGGGGAGATGGAGGTCTGGGCACTGGAAGCATACGGTGCTGCATACACTCTGCA
AGAAATGCTCACAGTGAAGTCGGACGATGTGAACGGCCGGACCAAGATGTACAAAAACATCGTGGACGGCGATCACCGTATGGA
GCCGGGCATGCCCGAGTCCTTCAACGTGTTGATCAAAGAAATTCGTTCCCTCGGCATCGATATCGATCTGGAAACCGAATAA

P. citronellolis P3B5_GCF_001586155.1_CP014158.1

ATGGCTTACTCATACACTGAGAAAAAACGTATCCGCAAGGACTTTAGCAAGTTGCCGGACGTCATGGATGTGCCGTATTTGCTGG
CCATCCAGCTGGATTCCTATCGCGAATTCCTGCAGGCAGGTGCAAGCAAGGATCAGATCCGCGACATCGGTCTGCATGCGGCCTT
CAAGTCCGTTTTCCCGATTATCAGCTATTCCGGCAACGCTGCCCTGGAATACGTCGGTTATCGCCTGGGTGAGCCGGCCTTCGACG
TCAAGGAGTGCGTGCTGCGCGGCGTGACCTTCGCCGTCCCGCTGCGGGTGAAAGTGCGCCTGATCATCTTCGACAAAGAGTCGTC
GAGCAAAGCGATCAAGGACATCAAGGAACAGGAAGTCTACATGGGGGAAATCCCCCTGATGACCGAGAACGGTACCTTCATCAT
CAACGGTACCGAGCGCGTCATCGTTTCCCAGCTGCACCGTTCCCCGGGTGTGTTCTTCGATCACGACCGTGGCAAGACCCACAGCT
CCGGCAAGCTGCTGTACTCCGCGCGCATCATTCCTTACCGCGGTTCCTGGCTGGACTTCGAATTCGATCCGAAGGACTGCGTGTTC
GTCCGTATCGACCGTCGCCGCAAGCTGCCGGCGTCCGTCCTGCTGCGCGCGCTGGGCTATAGCACCGAGGAGATCCTCGATGCCT
TCTACGCGACCAACGTCTACCACATCAAGGGCGAAGGTCTGCGTCTGGAGCTGGTGCCGCACCGTCTGCGCGGCGAGATCGCCAG
CTTCGACATCAAGGATGCCGCCGGCAAGGTCATCGTCGAAGCGGGCCGCCGTATCACCGCGCGCCACATCAACCAGCTGGAAAA
GGCTGGCATCACCGAGCTGGAAGTGCCCTTCGACTACCTCATTGGCCGTACCGTGGCCAAGGCTGTCGTGCACCCGGCTACCGGC
GAGATCATCGCCGAGTGCAACACCGAGCTGACCGTCGACGTACTGGCGAAGATCGCCAAGGCCCAGGTCGTGCGCCTGGAAACC
CTGTACACCAACGACATCGACTGCGGTCCGTTCATCTCCGACACGCTGAAGATCGACTCCACCGGCAACCAGTTGGAAGCCCTGG
TCGAGATCTACCGCATGATGCGTCCCGGCGAGCCGCCGACCAAGGAAGCCGCAGAGACCCTGTTCGGCAACCTGTTCTTCAGCGC
CGAACGCTACGACCTGTCCGCCGTCGGCCGCATGAAGTTCAACCGCCGCATCGGCCGCAGCGAGATCGAAGGTCCGGGCGTGCTG
AGCAAGGAAGACATCGTCGACGTACTGCGCACCCTGGTCGACATCCGTAACGGCAAGGGCATCGTCGACGACATCGACCACCTG
GGCAACCGTCGCGTCCGCTGCGTCGGCGAGATGGCCGAGAACCAGTTCCGCGTTGGCCTGGTGCGCGTCGAGCGCGCGGTCAAGG
AACGTCTGTCCATGGCCGAAAGCGAAGGCCTGATGCCGCAGGACCTGATCAACGCCAAGCCGGTGGCCGCCGCGATCAAGGAGT
TCTTCGGTTCCAGCCAGCTGTCGCAGTTCATGGACCAGAACAACCCGCTCTCCGAGATCACCCACAAGCGCCGTGTCTCCGCACTC
GGCCCGGGCGGTCTGACCCGTGAGCGCGCGGGCTTCGAGGTCCGTGACGTACACCCGACCCACTACGGCCGCGTGTGCCCGATCG
AAACCCCCGAAGGTCCGAACATCGGTCTGATCAACTCCCTGGCGACCTACGCCCGCACCAACAAGTACGGCTTCCTGGAAAGCCC
GTACCGCGTGGTGAAGGAAGGTCTGGTCAGCGACGAGATCGTGTTCCTCTCCGCCATCGAAGAGGCCGACCACGTGATCGCCCAG
GCCTCCGCGACCCTGGACGCCAAGGGCCGCCTGATCGACGAGCTGGTCGCCGTACGTCACCTGAACGAATTCACCGTGAAGGCGC
CGGAAGACGTCACCCTCATGGACGTATCGCCGAAGCAGGTCGTTTCCGTCGCTGCCTCGCTGATTCCGTTCCTCGAGCACGACGAC
GCCAACCGTGCGCTCATGGGTTCGAACATGCAGCGTCAGGCCGTTCCGACCCTGCGCGCCGACAAGCCGCTGGTGGGCACCGGCA
TGGAGCGCAACGTCGCCCGTGACTCCGGCGTCTGCGTGGTGGCTCGCCGCGGCGGTGTGATCGACTCGGTCGACGCCAGCCGTAT
CGTGGTGCGCGTGGCGGATGACGAGGTCGAGACCGGTGAAGCCGGCGTCGACATCTACAACCTGACCAAGTACACCCGTTCCAA
CCAGAACACCTGCATCAACCAGCGTCCGCTGGTCAGCAAGGGTGACGTGATCGCCCGCGGCGACATCCTCGCCGACGGTCCGTCC
ACCGACATGGGTGAACTGGCCCTGGGCCAGAACATGCGCGTCGCGTTCATGCCCTGGAACGGCTTCAACTTCGAAGACTCCATCT
GCCTGTCCGAGCGCGTGGTCCAGGAGGATCGTTTCACCACGATCCACATCCAGGAGCTGACCTGCGTCGCCCGTGACACCAAGCT
CGGCCCAGAAGAAATCACCGCGGACATCCCGAACGTGGGTGAGGCCGCGCTGAACAAGCTGGACGAAGCTGGCATCGTCTACGT
CGGCGCCGAAGTGCAGGCCGGCGACATCCTGGTCGGCAAGGTCACCCCGAAAGGCGAGACCCAGCTGACTCCGGAAGAGAAGCT
GCTGCGCGCGATCTTCGGTGAGAAGGCGTCCGACGTTAAGGACACCTCCCTGCGCGTGCCGACCGGCACCAAGGGCACCGTGATC
GACGTCCAGGTCTTCACCCGCGACGGCGTGGAGCGCGACAGCCGCGCCCTGTCCATCGAGAAGATGCAGCTCGACGAGATCCGC
AAGGACCTCAACGAGGAGTTCCGCATCGTCGAAGGCGCCACCTTCGAGCGTCTGCGTTCGGCCCTGGTCGGCGCCGTCGCCGATG
GCGGCCCGGCGCTGAAGAAAGGCACCGAGATCACCGACGAGTACCTCGACGGTCTCGAGCGCGGCCAGTGGTTCAAGCTGCGCA
TGGCCGAAGACGCCCTGAACGAGCAGTTGGAGAAGGCCCAGGCCTACCTCAGCGACCGCCGTCAGATGCTCGACGACAAGTTCG
AGGACAAGAAGCGCAAGCTGCAGCAGGGCGACGACCTGGCTCCGGGCGTGCTGAAGATCGTCAAGGTCTACCTCGCCATCAAGC
GCCGCATCCAGCCGGGCGACAAGATGGCCGGCCGCCACGGTAACAAGGGCGTGGTCTCGGTGATCATGCCGGTCGAAGACATGC
CGCACGACGCCAACGGCACGCCGGTGGACATCGTTCTGAACCCGCTGGGCGTACCCTCGCGTATGAACGTCGGTCAGATCCTCGA
AACCCACCTGGGCCTCGCGGCCAAGGGCCTGGGCGAGAAGATCAACCGCATGCTCGAAGAGCAGCGCAAGATCGCCGAGCTGCG
CCAGTTCCTGAACGAGATCTACAACGAGATCGGTGGCCGCCAGGAGAACCTGGAAGAGCTGTCCGATAACGAGATCGTCGCTCTG

GCCAACAACCTCAAGGGCGGCGTGCCCATGGCTACCCCGGTGTTCGACGGTGCCAAGGAGCGCGAGATCAAGGCCATGCTGAAG
CTGGCCGACCTGCCGGAGAGCGGCCAGATGCGCCTGTTCGACGGCCGCACCGGCAACCAGTTCGAGCGTCCGACCACCGTCGGCT
ACATGTACATGCTCAAACTGAACCACCTGGTCGATGACAAGATGCACGCACGTTCCACCGGCTCCTACAGCCTGGTCACCCAGCA
GCCGCTGGGTGGCAAGGCGCAGTTCGGTGGCCAGCGTTTCGGGGAGATGGAGGTCTGGGCGCTGGAAGCCTACGGCGCCGCCTA
CACCCTGCAGGAAATGCTGACCGTGAAGTCGGACGACGTGAACGGCCGTACCAAGATGTACAAGAACATCGTGGACGGGGATCA
CCGCATGGAGGCCGGCATGCCCGAGTCCTTCAACGTGCTGATCAAAGAGATCCGCTCGCTCGGCATCGATATCGAACTGGAAACC
GAATAA

P. corrugata RM1-1-4_GCF_001708425.1_CP014262.1

ATGGCTTACTCATATACTGAGAAAAAACGTATCCGCAAGGACTTTAGCAAGTTGCCGGACGTCATGGATGTGCCGTACCTCCTGG
CCATCCAGCTGGATTCGTATCGTGAATTCTTGCAAGCGGGAGCGACTAAAGATCAGTTCCGCGACGTGGGCCTGCATGCGGCCTT
CAAATCCGTTTTCCCGATCATCAGCTACTCCGGCAATGCTGCGCTGGAGTACGTCGGTTATCGCCTGGGCGAACCGGCATTTGATG
TCAAAGAATGCGTATTGCGCGGTGTAACTTACGCCGTACCTTTGCGGGTGAAAGTGCGCCTGATCATTTTCGACAAAGAATCGTCG
AACAAAGCGATCAAGGACATCAAAGAGCAAGAAGTCTACATGGGTGAAATCCCCCTGATGACTGAGAACGGTACCTTCGTAATC
AACGGTACCGAGCGTGTAATCGTTTCCCAGCTGCACCGTTCTCCGGGCGTGTTCTTCGACCACGACCGTGGCAAGACGCACAGCT
CCGGCAAACTGCTCTACTCCGCGCGCATCATTCCTTACCGCGGTTCCTGGTTGGACTTCGAGTTCGACCCGAAAGACTGCGTATTC
GTGCGTATCGACCGTCGTCGCAAACTGCCAGCATCGGTATTGTTGCGTGCGCTCGGCTACACCACGGAAGAAGTTCTGGACGCGT
TTTACACCACCAACGTCTTCCATGTGCAGGGTGAAAACCTCAGCCTGGAACTGGTGCCTCAGCGCCTGCGCGGTGAAATCGCGGT
CCTCGATATCCAGGACGACAAGGGCAAGGTTATTGTCGAGCAGGGTCGTCGTATCACCGCTCGCCACATCAACCAGCTGGAAAAG
GCCGGGATCAAAGAGCTGCAGGTGCCTATGGACTACGTCCTCGGTCGCACCACGGCCAAGGTCATCGTGCACCCGGCTACCGGCG
AAATCCTCGCAGAGTGCAACACCGAACTGAGCACCGAGATCCTGGCGAAAATCGTCAAGGCTCAGGTCGTTCGTATCGAAACTCT
GTACACCAACGATATCGACTGCGGTCCGTTCATCTCCGATACGTTGAAGATCGACTCCACCGGCAACCAACTGGAAGCGCTGGTC
GAGATCTATCGCATGATGCGTCCTGGCGAGCCACCAACCAAGGATGCCGCGGAAACGCTGTTCAACAACCTGTTCTTCAGTCCTG
AGCGCTATGACCTGTCTGCGGTCGGCCGGATGAAGTTCAACCGTCGTATCGGTCGTACCGAGATCGAAGGTTCGGGCGTGCTGAA
CAAGGATGACATCGTTGCAGTACTCAAGACCCTGGTCGACATCCGTAACGGCAAAGGCATCGTCGATGACATCGACCACTTGGGT
AACCGTCGCGTTCGCTGCGTAGGCGAAATGGCCGAGAACCAGTTCCGTGTTGGCCTGGTGCGTGTAGAGCGCGCGGTCAAGGAAC
GTCTGTCGATGGCTGAAAGCGAAGGCCTGATGCCGCAAGACCTGATCAACGCCAAGCCGGTAGCGGCGGCGGTGAAGGAGTTCT
TCGGTTCCAGCCAGCTGTCCCAGTTCATGGACCAGAACAACCCGCTGTCCGAGATCACCCACAAGCGTCGTGTCTCCGCACTCGG
CCCAGGTGGTCTGACCCGTGAGCGCGCAGGCTTCGAAGTTCGTGACGTACACCCGACCCACTACGGTCGCGTCTGCCCGATCGAA
ACGCCGGAAGGTCCGAACATCGGTCTGATCAACTCCCTGGCCGCCTATGCGCGCACCAACCAGTACGGCTTCCTCGAGAGCCCGT
ACCGTGTGGTGAAAGACGCTCTGGTCACCGACGAGATCGTGTTCCTGTCCGCCATCGAAGAGGCCGATCACGTTATCGCTCAGGC
CTCGGCCACGATGAACGACAAGAAGGTGCTGATCGACGAGCTGGTAGCTGTTCGTCACCTGAACGAGTTCACCGTCAAGGCGCCG
GAAGACGTCACCCTGATGGACGTGTCGCCCAAGCAGGTTGTTTCGGTCGCAGCGTCGCTGATCCCGTTCCTTGAGCACGACGACG
CCAACCGTGCGTTGATGGGTTCGAACATGCAGCGTCAGGCTGTACCGACCCTGCGTGCCGACAAGCCGCTGGTAGGTACCGGCAT
GGAGCGTAACGTTGCCCGTGACTCCGGCGTTTGCGTCGTGGCTCGTCGTGGCGGCGTGATCGATTCCGTCGATGCCAGTCGTATCG
TGGTTCGTGTTGCCGATGACGAAGTGGAGCCGGGCGAAGCCGGTGTCGACATCTACAACCTGACCAAATACACCCGCTCGAACCA
GAACACCTGCATCAACCAGCGTCCGCTGGTGAGCAAGGGTGATCGGGTCCAGCGCAGCGACATCATGGCTGACGGCCCGTCCACC
GACATGGGTGAGCTGGCGCTGGGTCAGAACATGCGCATCGCGTTCATGGCGTGGAACGGCTTCAACTTCGAAGACTCCATCTGCC
TGTCCGAGCGTGTGGTTCAGGAAGACCGCTTCACCACGATCCACATCCAGGAACTGACCTGTGTGGCCCGTGACACCAAGCTTGG
CCCAGAGGAAATCACTGCGGACATCCCGAACGTGGGTGAAGCTGCGCTGAACAAGCTGGACGAAGCCGGTATCGTTTATGTAGGT
GCCGAAGTAGGCGCTGGCGACATCCTGGTCGGCAAGGTCACACCGAAAGGCGAGACCCAGCTGACTCCGGAAGAAAAGCTGCTG
CGTGCCATCTTCGGTGAGAAGGCCAGCGACGTTAAGGACACCTCCCTGCGCGTGCCTACTGGCACCAAGGGTACCGTCATCGACG
TACAAGTCTTCACTCGCGACGGCGTTGAGCGTGATGCACGTGCACTGTCCATCGAGAAGACTCAGCTCGACGAAATCCGCAAGGA
CCTGAACGAAGAGTTCCGTATCGTTGAGGGTGCAACTTTCGAACGTCTGCGTGCAGCGCTGGTTGGCCACAAGGCCGAAGGCGGC
GCCGGCCTGAAGAAAGGTCAGGAAATCACCGACGAAGTCCTCGACGGTCTTGAGCATGGCCAGTGGTTCAAACTGCGCATGGCT
GAGGACGCGTTGAACGAGCAGCTCGAGAAGGCTCAGGCTTACATCGTTGATCGCCGTCGCCTGTTGGACGACAAGTTCGAAGACA
AGAAGCGCAAGCTGCAGCAGGGCGATGACCTGGCTCCGGGCGTACTGAAGATCGTCAAGGTCTACCTGGCAATCCGTCGTCGCAT
CCAGCCGGGTGACAAGATGGCTGGTCGTCACGGTAACAAGGGTGTGGTCTCCGTGATCATGCCGGTTGAAGACATGCCGCACGAT
GCCAACGGCACTCCGGTCGACGTCGTCCTCAACCCGCTGGGCGTACCTTCGCGTATGAACGTCGGTCAGATCCTTGAAACCCACCT
GGGCCTCGCGGCCAAGGGTCTGGGCGAGAAGATCAACCGCATGCTCGAAGAGCAGCGCAAGGTCGCTGACCTGCGCAAGTTCCT
GCACGAGATCTACAACGAGATCGGCGGTCGTAACGAAGAGCTCGACAGCTTCTCCGACCAGGAAATCCTGGATCTGGCGAAGAA
CCTCAAGGGCGGCGTTCCAATGGCCACTCCGGTGTTCGACGGTGCCAAGGAAGTTGAAATCAAGGCCATGCTGAAACTGGCAGAT
CTGCCAGAAAGCGGCCAGATGCAGCTGTTCGATGGTCGTACCGGCAACAAGTTCGAGCGTCCGGTTACCGTTGGCTACATGTACA
TGCTGAAGCTGAACCACTTGGTGGACGACAAGATGCACGCTCGTTCTACCGGTTCTTACAGCCTGGTTACCCAGCAGCCGCTGGG
TGGTAAGGCGCAGTTCGGTGGTCAGCGTTTCGGGGAGATGGAGGTCTGGGCACTGGAAGCATACGGTGCTGCATACACTCTGCAA
GAAATGCTCACAGTGAAGTCGGACGATGTGAACGGTCGGACCAAGATGTACAAGAACATCGTGGACGGCGATCACCGTATGGAG
CCGGGCATGCCCGAGTCCTTCAACGTGTTGATCAAGGAAATTCGTTCCCTCGGCATCGATATCGATCTGGAAACCGAATAA

P. entomophila L48_GCF_000026105.1_CT573326.1

ATGGCTTACTCATACACTGAGAAAAAACGTATCCGCAAGGACTTTAGCAAGTTGCCGGACGTCATGGATGTGCCTTACCTCCTGG
CCATCCAGCTGGATTCGTATCGCGAATTCCTGCAGGCGGGAGCATCCAAGGATCAGTTCCGCGACGTCGGTCTGCATGCGGCCTT
CAAATCGGTATTCCCGATCATCAGCTACTCCGGCAATGCTGCCCTGGAGTACGTCGGCTATCGCCTGGGCGAGCCGGCCTTCGATG
TGAAGGAATGTGTCCTGCGCGGTGTGACCTTTGCGGTCCCGCTGCGGGTGAAGGTGCGCCTGATCATCTTCGACAAGGAATCGTC
GAACAAAGCGATCAAGGACATCAAAGAGCAAGAAGTCTACATGGGTGAAATTCCCCTGATGACTGAAAACGGTACCTTCGTTATC

AACGGTACCGAGCGTGTAATCGTATCTCAGCTGCACCGTTCGCCGGGTGTGTTCTTCGACCACGACCGTGGCAAGACCCACAGCT
CCGGCAAGCTGCTGTACTCCGCGCGCATCATCCCTTACCGCGGTTCCTGGCTGGACTTCGAGTTCGACCCGAAGGACTGCGTGTTC
GTGCGTATCGACCGTCGCCGCAAGCTGCCGGCCTCGGTACTGCTGCGCGCGCTGGGCTACAGCACCGAAGAAGTACTCAACACCT
TCTACACCACCAACGTGTTCCACCTGTCCGGCGAGAAACTCAGCCTGGAACTGGTGCCACAGCGCCTGCGTGGTGAAGTCGCGGT
CATGGACATCCATGACGGCAGCGGCAAGGTCATCGTCGAGCAGGGCCGCCGTATCACCGCGCGCCACATCAACCAGCTGGAAAA
GGCTGGCGTGAAAGAGCTGGACGTTCCGCTGGAGTACGTACTGGGCCGCACCACCGCCAAGGCGATCGTGCACCCGGCTACCGG
CGAGATCCTGGCCGAATGCAACACCGAGCTGACCACCGATCTGCTGGTCAAGATCGCCAAGGCCCAGGTCGTTCGCATCGAGACG
CTGTACACCAACGACATCGACTGCGGTCCGTTCATTTCGGACACCCTGAAGATCGACACCACCAGCAACCAACTGGAAGCGCTGG
TCGAGATCTATCGCATGATGCGTCCTGGCGAGCCGCCAACCAAAGACGCCGCCGAAACCCTGTTCAACAACCTGTTCTTCAGCGC
CGAGCGTTACGACCTGTCTGCCGTCGGCCGCATGAAGTTCAACCGTCGTATCGGTCGTACCGAGATCGAAGGTTCGGGCGTGCTG
AGCAAGGAAGACATCGTCGAGGTCCTGAAGACCCTGGTCGATATCCGTAACGGCAAAGGCATCGTCGACGACATCGACCACCTG
GGTAACCGTCGCGTCCGTTGTGTCGGCGAGATGGCCGAAAACCAGTTCCGCGTTGGCCTGGTGCGTGTAGAGCGCGCGGTCAAAG
AGCGTCTGTCGATGGCCGAAAGCGAAGGCCTGATGCCTCAGGACCTGATCAACGCCAAGCCGGTAGCGGCGGCGGTGAAAGAGT
TCTTCGGTTCCAGCCAGCTCTCGCAGTTCATGGGCCAGAACAACCCGCTCTCCGAGATCACCCACAAGCGCCGTGTCTCTGCACTC
GGCCCTGGCGGTCTGACCCGTGAGCGTGCAGGCTTCGAAGTCCGTGACGTACACCCGACCCACTATGGCCGTGTGTGCCCGATCG
AAACGCCGGAAGGTCCGAACATCGGTCTGATCAACTCCCTGGCCGCTTATGCCCGCACCAACCAGTACGGCTTCCTGGAAAGCCC
GTACCGCGTGGTGAAAGAGGGTGTGGTCAGCGACGACATCGTGTTCCTGTCGGCGATCGAAGAAGCGGATCACGTCATTGCACAG
GCTTCGGCCGCAATGAACGAGAAGAAGCAGCTGATCGATGAGCTGGTGGCCGTTCGTCACCTGAACGAATTCACCGTCAAGGCGC
CGGAAGACGTCACCTTGATGGACGTTTCGCCGAAGCAGGTAGTTTCGGTCGCAGCGTCGCTGATTCCGTTCCTCGAGCACGACGA
CGCCAACCGTGCGTTGATGGGTTCGAACATGCAGCGTCAGGCTGTACCAACCCTGCGCGCCGACAAGCCGCTGGTCGGTACCGGC
ATGGAGCGCAACGTTGCCCGTGACTCCGGTGTCTGCGTGGTTGCTCGCCGCGGTGGTGTGATCGACTCGGTCGACGCCAGCCGTA
TCGTTGTTCGCGTGGCTGACGACGAAGTCGAGACTGGTGAAGCCGGTGTGGATATCTACAACCTCACCAAGTACACCCGTTCGAA
CCAGAACACCTGCATCAACCAGCGTCCGCTGGTGAGCAAGGGTGATGTGGTCGCGCGTGGCGACATCATGGCTGACGGTCCGTCC
ACCGACATGGGTGAACTGGCACTGGGCCAGAACATGCGCATTGCGTTCATGGCGTGGAACGGCTTCAACTTCGAAGACTCCATCT
GCCTGTCCGAGCGTGTGGTCCAGGAAGACCGCTTCACCACGATCCACATCCAGGAACTGACCTGTGTGGCCCGTGACACCAAGCT
TGGCCCAGAGGAAATCACCGCGGACATCCCGAACGTGGGTGAAGCTGCGCTGAACAAGCTGGACGAAGCCGGTATCGTCTACGT
GGGTGCCGAAGTCGGCGCTGGCGACATCCTGGTCGGCAAGGTCACTCCGAAAGGCGAAACCCAGCTGACTCCGGAAGAAAAACT
GCTGCGTGCGATCTTCGGTGAGAAGGCCAGCGACGTCAAAGACACCTCCCTGCGCGTACCGACCGGTACCAAGGGCACCGTCATC
GACGTACAGGTCTTCACCCGTGATGGCGTTGAGCGCGACAGCCGCGCCCTGGCCATCGAGAAGATGCAACTGGACGAGATCCGC
AAGGACCTCAACGAAGAGTTCCGCATCGTCGAAGGCGCAACCTTCGAGCGTCTGCGTGCAGCCCTGAACGGCCAGGTGGTCGAC
GGTGGCGCGGGCCTGAAGAAAGGCACCGTGATCACTGACGACGTGCTGAACGGTCTGGAGCACGGCCAGTGGTTCAAACTGCGC
ATGGCAGAAGATGCACTGAACGAGCAGCTGGAAAAGGCTCAGCAGTACATCGTCGATCGCCGTCGCCTGCTGGACGACAAGTTC
GAAGACAAGAAGCGCAAGCTGCAGCAGGGCGATGACCTGGCACCGGGCGTACTGAAGATCGTCAAGGTCTACCTGGCCATCCGC
CGTCGCATCCAGCCGGGTGACAAGATGGCCGGTCGTCACGGTAACAAGGGTGTCGTCTCGGTGATCATGCCGGTCGAAGACATGC
CGCACGACGCCAACGGTACTCCGGTCGACGTCGTACTGAACCCGCTGGGCGTACCTTCGCGTATGAACGTCGGTCAGATCCTCGA
AACCCACCTGGGCCTCGCGGCCAAGGGCCTGGGCGAGAAGATCGACCGCATGATCGAAGAGCAGCGCAAGGCCGCTGAACTGCG
TACCTTCCTCACCGAGATCTACAACGAGATCGGTGGTCGTCAGGAGAACCTGGAAGAGTTCACCGACGAAGAGATCATGGCTCTG
GCAAACAACCTGAAGAAAGGCGTGCCTATGGCCACTCCAGTCTTCGACGGTGCCAAGGAGCGTGAGATCAAGGCCATGCTGAAG
CTGGCAGACCTGCCGGAAAGCGGCCAGATGCAGCTGTTCGACGGCCGCACCGGCAACAAGTTCGAGCGTTCTGTGACCGTTGGTT
ACATGTACATGCTCAAGCTGAACCACTTGGTGGACGACAAGATGCACGCGCGTTCCACTGGTTCCTACAGCCTGGTTACCCAGCA
GCCGCTGGGTGGTAAGGCGCAGTTCGGTGGTCAGCGTTTCGGGGAGATGGAAGTGTGGGCGCTGGAAGCATACGGCGCGGCATA
CACCCTGCAAGAAATGCTCACAGTGAAGTCGGACGACGTGAACGGCCGTACCAAGATGTACAAGAACATCGTGGATGGCGATCA
CCGTATGGAGCCGGGCATGCCCGAGTCCTTCAACGTGTTGATCAAAGAGATCCGTTCGCTCGGTATCGATATCGATCTGGAAACC
GAATAA

P. eucalypticola NP-1_GCF_013374995.1_CP056030.1

ATGGCTTACTCATACACTGAGAAAAAACGTATCCGCAAGGACTTTAGCAAGTTGCCGGACGTCATGGACGTACCGTACCTCTTGG
CTATCCAGCTGGATTCGTATCGCGAATTCTTGCAGGCGGGCGCGACCAAGGACCAGTTCCGTGACGTCGGTCTGCATGCGGCCTTC
AAATCCGTTTTCCCGATCATCAGCTACTCCGGCAACGCTGCTCTGGAGTACGTTGGTTATCGCCTGGGCGAACCGGCCTTTGATGT
CAAAGAATGCGTATTGCGCGGCGTAACGTACGCCGTACCTTTGCGGGTAAAAGTACGCCTGATCATTTTCGACAAAGAATCGTCG
AACAAAGCGATCAAGGACATCAAGGAGCAGGAAGTCTACATGGGGGAAATCCCCCTGATGACCGAGAACGGTACCTTCGTAATC
AACGGTACCGAGCGCGTCATCGTCTCCCAGTTGCACCGTTCGCCAGGTGTGTTCTTCGACCACGACCGTGGCAAGACCCACAGCT
CCGGCAAACTGTTGTACTCGGCCCGTATCATTCCTTACCGCGGCTCGTGGCTGGACTTCGAATTCGACCCGAAAGACTGTGTATTC
GTCCGGATCGACCGTCGTCGCAAGCTGCCTGCGTCGGTACTGCTGCGCGCACTGGGCTATAGCACCGAGGAAGTCCTCGATGCGT
TCTACACCACCAACGTATTCCACGTGCAGGGCGAAAAGCTCAGCCTGGAACTGGTGCCTCAGCGCCTGCGTGGTGAAGTTGCCGT
GCTGGACATCCTCGACAACACCGGCAAGGTGATCGTCGAGCAGGGTCGCCGCATCACTGCCCGCCACATCAACCAGTTGGAAAA
AGCCGGCATCAAAGAGCTGGAAGTTCCGCTGGACTACGTGCTGGGTCGTACCACTGCCAAGGCCATCGTTCACCCGGCCACTGGT
GAAATCCTGTGCGAATGCAACACCGAACTGAGCACCGAGCTGCTGGTGAAGATCGCCAAGGCCCAGGTCGTCCGTATCGAAACG
TTGTACACCAACGACATCGACTGCGGTCCGTTCATCTCCGACACGCTGAAGATCGACTCCACCGCCAACCAACTGGAAGCGCTGG
TCGAGATCTACCGCATGATGCGTCCTGGCGAGCCGCCAACCAAGGACGCCGCCGAGACCCTGTTCAACAACCTGTTCTTCAGCCC
TGAGCGCTACGATCTGTCCGCTGTAGGCCGCATGAAGTTCAACCGTCGTATCGGTCGTACCGAGATCGAAGGTTCGGGCGTGCTG
AGCAAGGAAGACATCGTTGCCGTTCTGAAGACCCTGGTCGACATCCGTAACGGCAAAGGCATCGTCGACGACATCGACCACCTGG
GTAACCGTCGTGTTCGCTGCGTAGGTGAAATGGCCGAGAACCAGTTCCGTGTTGGCCTGGTGCGCGTTGAGCGTGCGGTCAAGGA
ACGCCTGTCGATGGCTGAAAGCGAAGGCCTGATGCCTCAGGACCTGATCAACGCCAAGCCGGTAGCGGCCGCGGTGAAAGAGTT

CTTCGGTTCCAGCCAACTGTCCCAGTTCATGGACCAGAACAACCCGCTCTCGGAAATTACCCACAAGCGTCGTGTCTCCGCACTCG
GCCCAGGTGGTCTGACCCGCGAGCGCGCAGGCTTCGAAGTTCGTGACGTACACCCGACTCACTACGGTCGTGTATGCCCGATCGA
GACCCCTGAAGGTCCGAACATCGGTCTGATCAACTCCCTGGCCGCCTACGCGCGTACCAACCAGTACGGCTTCCTGGAAAGCCCG
TACCGCGTTGTTAAAGAGGGCGTTGTCACCGACGAGATCGTGTTCCTGTCCGCCATCGAAGAAGCCGATCACGTGATCGCGCAGG
CTTCGGCGACCATGAACGAGAACAAGGTCCTGATCGACGAGCTGGTAGCCGTACGTCACCTGAACGAATTCACCGTGAAGGCGCC
TGAAGACGTAACCCTGATGGACGTTTCGCCCAAGCAGGTAGTCTCGGTCGCAGCCTCGCTGATTCCGTTCCTGGAGCACGACGAC
GCCAACCGTGCGTTGATGGGTTCCAACATGCAGCGTCAGGCTGTACCGACCCTGCGCGCCGACAAGCCGCTGGTAGGTACCGGCA
TGGAGCGCAACGTTGCCCGTGACTCCGGCGTTTGCGTCGTGGCCCGTCGTGGCGGCGTGATCGACTCCGTCGATGCCAGCCGTATC
GTTGTTCGCGTGGCGGACGATGAAGTCGAGCCAGGCGAAGCCGGTGTAGATATCTACAACCTGACCAAGTACACCCGTTCGAACC
AGAACACCTGCATCAACCAGCGTCCGCTGGTGAGCAAGGGTGACCGCGTTCAGCGCAGCGACATCATGGCCGACGGCCCGTCCA
CCGACATGGGTGAGCTGGCACTGGGTCAGAACATGCGCATCGCGTTCATGGCCTGGAACGGTTTCAACTTCGAAGACTCCATCTG
TCTGTCGGAGCGTGTGGTTCAGGAAGATCGTTTCACCACGATCCACATCCAGGAACTGACCTGTGTGGCCCGTGACACCAAGCTT
GGCCCAGAGGAAATCACCGCTGACATCCCGAACGTGGGTGAAGCAGCGCTGAACAAGCTGGACGAAGCCGGTATCGTTTACGTA
GGTGCCGAAGTTGGCGCCGGCGACATCCTGGTCGGCAAGGTCACCCCGAAAGGCGAGACCCAGCTGACTCCGGAAGAAAAACTG
CTGCGCGCCATCTTCGGTGAAAAAGCCAGCGACGTTAAGGACACCTCCCTGCGCGTGCCTACCGGCACCAAAGGTACTGTCATCG
ACGTCCAGGTCTTCACCCGTGACGGCGTGGAGCGTGACGCTCGTGCACTGTCGATCGAGAAGAGCCAGCTGGACGAGATCCGCAA
GGATCTGAACGAAGAGTTCCGTATCGTCGAAGGCGCGACCTTCGAACGTCTGCGTCAGGCCCTGGTCGGCCAGATCGTCGACGGT
GGTGCCGGCCTGAAAAAAGGTCTGGAAATCACCAACGAAGTCCTGGACGGTCTGGAACACGGTCAGTGGTTCAAGCTGCGCATG
GCCGAAGACGCGCTCAACGAACAGCTGGAAAAAGCCCAGCAGTACATCGTTGACCGTCGTCGCCTGCTGGACGACAAGTTCGAA
GACAAGAAGCGCAAACTGCAGCAAGGCGATGACCTGGCTCCAGGCGTGCTGAAAATCGTCAAGGTTTACCTGGCAATCCGTCGC
CGCATCCAGCCGGGCGACAAGATGGCCGGTCGTCACGGTAACAAGGGTGTGGTCTCCGTGATCATGCCGGTTGAAGACATGCCAC
ACGATGCCAACGGCACCCCTGTGGACGTGGTACTCAACCCGCTGGGCGTACCTTCGCGTATGAACGTCGGTCAGATTCTGGAAAC
CCACCTGGGCCTCGCGGCCAAGGGCTTGGGCGAAAAGATCAACCGTATGCTCGAAGAGCAGCGTAAAGTCATCGAACTGCGCAA
GTTCCTCACCGAGATCTACAACGAGATCGGTGGCCGCCAAGAGAATCTGGAAAGCTTCAGCGACCAGGAAATCCTGGACCTGGC
GAAGAACCTGCGTGCTGGCGTTCCAATGGCTACCCCGGTATTCGACGGTGCCAAGGAAAGCGAAATCAAGGCCATGCTGAAACT
GGCAGACCTGCCGGAAAGCGGCCAGATGCAGCTGTTCGACGGCCGGACTGGCAACAAGTTCGAGCGTCACGTTACTGTTGGCTAC
ATGTACATGCTGAAACTGAACCACTTGGTGGACGACAAGATGCACGCGCGTTCCACTGGTTCCTACAGCCTGGTTACCCAGCAGC
CGCTGGGTGGTAAGGCTCAGTTCGGTGGTCAGCGTTTCGGGGAGATGGAGGTCTGGGCACTGGAAGCTTATGGTGCCGCCTACAC
CCTGCAAGAAATGCTCACAGTGAAGTCGGACGATGTGAACGGTCGTACCAAGATGTACAAGAACATCGTGGACGGCGATCACCG
TATGGAGCCGGGCATGCCCGAGTCCTTCAACGTGTTGATCAAAGAGATTCGTTCCCTCGGTATCGATATCGATCTGGAAACCGAAT
AA

P. glycinae MS586_GCF_001594225.2_CP014205.2

ATGGCTTACTCATATACTGAGAAAAAACGTATCCGCAAGGACTTTAGCAAGTTGCCGGACGTCATGGATGTGCCTTACCTCCTGGC
CATCCAGCTGGATTCGTATCGTGAATTCTTGCAAGCGGGAGCGACTAAAGATCAGTTCCGCGACGTGGGCCTGCATGCGGCCTTC
AAATCCGTTTTCCCGATCATCAGCTACTCCGGCAATGCTGCGCTGGAGTACGTCGGTTATCGCCTGGGCGAACCGGCATTTGATGT
CAAAGAATGCGTATTGCGCGGTGTAACTTTCGCCGTACCTTTGCGGGTAAAAGTGCGCCTGATCATTTTCGACAAAGAATCGTCGA
ACAAAGCGATCAAGGACATCAAAGAGCAAGAAGTCTACATGGGTGAAATCCCATTGATGACTGAGAACGGTACCTTCGTAATCA
ACGGTACCGAGCGTGTGATCGTTTCCCAGCTGCACCGTTCCCCGGGCGTGTTCTTCGACCACGACCGTGGCAAGACGCACAGCTC
CGGCAAACTGCTGTACTCCGCGCGCATCATTCCTTACCGCGGTTCGTGGCTGGACTTCGAGTTCGACCCGAAAGACTGCGTCTTCG
TGCGTATCGACCGTCGTCGCAAGCTGCCGGCCTCGGTTTTGCTGCGTGCACTCGGTTACACCACCGAAGAAGTGCTGGACGCGTTC
TACACCACCAACGTATTCCACCTGAGCGGCGAAACCCTCAGCCTGGAACTGGTGCCATCGCGCCTGCGTGGTGAAGTTGCGGTTC
TGGACATCCAGGATGAGAAGGGCAAGGTCATCGTTGAGCAAGGCCGCCGTATTACCGCGCGCCACATCAACCAGATCGAAAAAG
CCGGAATCAAGACGCTGGATGTGCCGCTGGACTACGTCCTCGGTCGTACGACCGCGAAGGCCATCGTGCACCCGGCTACCGGTGA
GATCCTGGCAGAGTGCAACACCGAGCTGTCGACCGAGATCCTGGCGAAAATCGCCAAGGCCGGCGTCGTACGCATCGAGACTCT
GTACACCAACGACATCGACTGCGGTCCGTTCGTCTCCGACACGCTGAAGATCGACTCCACCAGCAACCAATTGGAAGCGCTGGTC
GAGATTTATCGCATGATGCGTCCAGGCGAGCCGCCAACCAAGGACGCTGCCGAGACCCTGTTCAACAACCTGTTCTTCAGCCCTG
AGCGTTACGATCTCTCTGCGGTCGGCCGGATGAAGTTCAACCGTCGTATCGGTCGTACCGAGATCGAAGGTTCGGGTGTGCTGTGC
AAGGAAGACATCGTCGCGGTACTGAAGACTCTGGTCGACATCCGTAACGGTAAAGGCATCGTCGATGACATCGACCACCTGGGTA
ACCGTCGTGTTCGCTGCGTAGGCGAAATGGCCGAGAACCAGTTCCGCGTTGGCCTGGTACGTGTTGAGCGTGCGGTCAAAGAGCG
CCTGTCGATGGCTGAAAGCGAAGGCCTGATGCCGCAAGATCTGATCAACGCCAAGCCAGTGGCTGCGGCGGTGAAAGAGTTCTTC
GGTTCCAGCCAGCTGTCGCAGTTCATGGACCAGAACAACCCGCTGTCCGAGATCACCCACAAGCGTCGTGTCTCTGCACTCGGCC
CTGGCGGTCTGACTCGTGAGCGCGCGGGCTTCGAAGTCCGTGACGTACACCCGACTCACTACGGTCGTGTCTGCCCGATTGAAAC
GCCGGAAGGTCCGAACATCGGTCTGATCAACTCCCTGGCTGCTTACGCTCGCACCAACCAGTACGGCTTCCTCGAGAGCCCGTAC
CGCGTGGTGAAAGAGGGTGTGGTCACCGACGACATCGTGTTCCTGTCTGCCATTGAAGAAGCCGATCACGTGATCGCGCAGGCTT
CGGCGACCATGAACGAGCAGAAAGTCCTGGTCGACGAACTGGTGGCCGTACGTCACCTGAACGAATTCACCGTCAAGGCGCCGG
AAGACGTCACCTTGATGGACGTTTCGCCGAAGCAGGTAGTTTCGGTTGCAGCGTCGCTGATTCCGTTCCTCGAGCACGACGACGC
CAACCGTGCATTGATGGGTTCGAACATGCAGCGTCAAGCTGTACCAACCCTGCGCGCTGACAAGCCGCTGGTCGGTACCGGCATG
GAGCGTAACGTGGCTCGCGACTCCGGCGTTTGCGTCGTGGCTCGTCGCGGCGGCGTGATCGACTCCGTTGATGCCAGCCGTATCGT
GGTTCGCGTTGCTGATGACGAAGTTGAAACCGGCGAAGCGGGTGTCGACATCTACAACCTGACCAAATACACCCGCTCGAACCAG
AACACCTGCATCAACCAGCGTCCGTTGGTGAGCAAGGGTGATCGTGTTCAGCGTGGCGACATCATGGCCGACGGTCCGTCCACCG
ACATGGGTGAACTGGCTCTGGGTCAGAACATGCGCATCGCGTTCATGGCATGGAACGGCTTCAACTTCGAAGACTCCATCTGCCT
GTCCGAGCGTGTGGTTCAGGAAGACCGTTTCACCACGATCCACATTCAGGAACTGACCTGTGTGGCACGTGACACCAAGCTTGGC
CCAGAGGAAATCACTGCGGACATCCCTAACGTGGGTGAAGCTGCGCTGAACAAGCTGGACGAAGCCGGTATCGTTTACGTAGGTG

CTGAAGTTGGCGCAGGCGACATCCTGGTAGGCAAGGTCACTCCGAAAGGCGAGACCCAACTGACTCCGGAAGAAAAACTGCTGC
GCGCAATCTTCGGTGAAAAAGCCAGCGACGTTAAGGACACTTCCCTGCGCGTGCCAACGGGCACCAAAGGTACTGTCATCGACGT
ACAGGTCTTCACGCGTGACGGCGTTGAGCGTGATGCTCGTGCGCTGTCGATCGAGAAGTCCCAGCTGGACGAGATCCGCAAGGAT
CTGAACGAAGAGTTCCGCATCGTTGAAGGCGCGACCTTCGAGCGTCTGCGTTCCGCTCTGGTAGGCCACAAGGCTGAAGGCGGCG
CGGGCCTGAAGAAAGGTCAGGACATCACCGACGAAGTACTCGACGGTCTTGAGCATGGTCAGTGGTTCAAACTGCGCATGGCTGA
AGATGCTCTGAACGAGCAGCTCGAGAAGGCTCAGGCCTACATCGTTGATCGTCGCCGTCTGCTGGACGACAAGTTCGAAGACAAG
AAGCGCAAACTGCAGCAGGGCGATGACCTGGCTCCAGGCGTGCTGAAGATCGTCAAGGTTTACCTGGCAATCCGTCGTCGCATCC
AGCCGGGCGACAAGATGGCCGGTCGTCACGGTAACAAAGGTGTGGTCTCCGTGATCATGCCGGTTGAAGACATGCCGCACGATG
CCAATGGCACCCCGGTCGACGTGGTCCTCAACCCGCTGGGCGTACCTTCGCGTATGAACGTTGGTCAGATCCTTGAAACCCACCTG
GGCCTCGCGGCCAAAGGTCTGGGCGAGAAGATCAACCGGATGGTCGAAGAGCAGCGTAAAGTCGCTGAGCTGCGTACCTTCCTG
GACGAGATCTACAACCAGATCGGCGGTCGTAACGAAGATCTGGACAGCTTCTCCGATCAGGAAATCCTGGATCTGGCGAAGAAC
CTGCGTGGCGGCGTTCCAATGGCCACTCCAGTGTTCGACGGTGCCAAGGAAAGCGAAATCAAGGCCATGCTGAAACTGGCAGAC
CTGCCAGAAAGCGGCCAGATGCAGCTGACCGACGGCCGTACCGGCAACAAGTTCGAGCGTCCAGTTACCGTTGGCTACATGTACA
TGCTGAAGCTGAACCACTTGGTAGACGACAAGATGCACGCGCGTTCTACCGGTTCGTACAGCCTGGTTACCCAGCAGCCGCTGGG
TGGTAAGGCGCAGTTCGGTGGTCAGCGTTTCGGGGAGATGGAGGTCTGGGCACTGGAAGCATACGGTGCTGCTTACACTCTGCAA
GAAATGCTCACAGTGAAGTCGGACGATGTGAACGGCCGGACCAAGATGTACAAAAACATCGTGGACGGCGATCACCGTATGGAG
CCGGGCATGCCCGAGTCCTTCAACGTGTTGATCAAGGAAATTCGTTCCCTCGGCATCGATATCGATCTGGAAACCGAATAA

P. lalkuanensis PE08_GCF_008807375.1_CP043311.1

ATGGCTTACTCATACACTGAGAAAAAACGTATCCGCAAAGACTTTAGCAAGTTGCCGGACGTCATGGATGTACCCTACCTCCTGG
CCATCCAGCTGGATTCGTATCGCGAATTCCTGCAGGCGGGAGTCAGCAAAGAGCAATTCCGTGACATCGGCCTGCACGCGGCCTT
CAAGTCTGTTTTCCCGATCATCAGCTATTCCGGCAACGCCGCTCTGGAATATGTCGGCTATCGCCTGGGTGAGCCGGCGTTCGATG
TCAAGGAATGCGTGCTGCGTGGCGTGACCTTCGCCGTGCCGCTGCGGGTAAAAGTCCGTCTGATCATTTTCGACAAAGAGTCGTC
GAACAAAGCGATCAAGGACATCAAGGAACAAGAAGTCTACATGGGGGAAATCCCCCTGATGACCGAGAACGGCACCTTCATCAT
CAACGGTACCGAGCGCGTCATCGTTTCCCAGTTGCACCGTTCTCCGGGTGTGTTCTTCGACCACGACCGTGGCAAGACCCACAGCT
CGGGCAAGCTGCTGTACTCCGCTCGAATCATCCCCTACCGCGGTTCCTGGCTGGACTTCGAGTTCGATCCGAAGGATTGCGTGTTC
GTCCGTATCGACCGTCGCCGCAAACTGCCGGCTACCGTCCTGCTGCGCGCGCTGAACTACAGCACTGAAGAAGTGCTGAACGCCT
TCTATGCCACCAACGTATTCCACGTGAAGGGCGAAGGCCTGCACCTGGAACTGGTTCCGCAGCGTCTGCGTGGCGAGATCGCCGT
GTTCGACATCAAGGACCAGAGTGGCAAGGTGATTGTTGAGCAGGGCCGTCGTATCACCGCCCGCCACATCAACCAGCTGGAAAA
GGCTGGCATCAAGGAGCTGGAAGTCCCGATGGACTACGTGCTTGGCCGTACCAGCGCCAAGGCTATCGTGCATCCGGCTACCGGC
GAGATCATTGCCGAGTGCAATACCGAGCTGACCACCGACCTGCTGGTGAAGATCGCCAAGGCCCAGGTAGTCCGCATCGAGACCC
TGTACACCAACGACATCGACTGCGGTCCGTTCATTTCGGACACCCTGAAGATCGACTCCACCGGCAACCAGCTGGAAGCCCTGGT
CGAGATCTACCGCATGATGCGTCCTGGCGAGCCGCCGACCAAGGAAGCCGCCGAAACCCTGTTCAACAACCTGTTCTTCAGCGCC
GAGCGTTACGACCTGTCCGCCGTCGGTCGCATGAAGTTCAATCGCCGCATCGGTCGTACCGAGATCGAAGGCTCCGGCGTGCTGA
GCAAGGAAGACATCGTTGACGTACTGAAGACCCTGGTCGACATCCGTAACGGCAAGGGCATCGTCGACGATATCGACCACCTGG
GTAACCGTCGCGTACGTTGCGTTGGCGAAATGGCCGAGAACCAGTTCCGTGTCGGCCTCGTGCGTGTAGAGCGCGCGGTCAAGGA
ACGTCTGTCCATGGCCGAAAGCGAAGGCCTGATGCCTCAGGACCTGATCAACGCCAAGCCGGTGGCTGCTGCGATCAAGGAGTTC
TTCGGCTCCAGCCAGCTGTCCCAGTTCATGGACCAGAACAACCCGCTCTCCGAGATCACCCACAAGCGCCGTGTTTCCGCACTCGG
CCCGGGCGGTCTGACTCGTGAACGTGCAGGCTTCGAAGTCCGCGACGTACACCCGACCCACTACGGCCGCGTGTGCCCGATCGAA
ACCCCTGAAGGTCCGAACATCGGTCTGATCAACTCCCTGGCGACCTACGCCCGCACCAACCAGTACGGCTTCCTGGAAAGCCCGT
ACCGCGTGGTCAAGGAAGGTACGGTCACCGACGACATCGTCTTCCTCTCCGCTATCGAAGAGGCTGACCACGTCATCGCCCAGGC
TTCCGCCACCCTGAACGAACAGGGTCAACTGGTGGACGAGCTGGTAGCCGTACGTCACCTGAACGAGTTCACCGTCAAGGCGCCG
GAAGACGTGACCCTGATGGACGTGTCGCCGAAGCAGGTCGTTTCCGTCGCTGCCTCGCTGATTCCGTTCCTCGAGCACGACGACG
CCAACCGTGCACTCATGGGCTCGAACATGCAGCGTCAGGCCGTGCCGACCCTGCGTGCCGACAAGCCTCTGGTAGGTACCGGCAT
GGAGCGCAACGTCGCCCGTGACTCCGGTGTCTGCGTCGTGGCCCGTCGTGGCGGCGTGATCGACTCCGTCGACGCCAGCCGTATC
GTGGTTCGCGTCAACGACGACGAAGTCGAGACCGGCGAAGCAGGTGTGGATATCTACAACCTGACCAAATACACCCGTTCCAACC
AGAACACCTGCATCAACCAGCGTCCGCTGGTGCAGAAGGGTGATGTGGTCGCTCGTGCCGACATCCTCGCCGATGGTCCGTCCAC
CGACATGGGTGAACTCGCTCTGGGTCAGAACATGCGCGTAGCGTTCATGCCCTGGAACGGTTTCAACTTCGAGGACTCCATCTGC
CTGTCCGAGCGTGTGGTGCAGGAAGATCGCTTCACCACCATCCACATCCAGGAACTGACCTGTGTGGCGCGTGACACCAAGCTCG
GCCCAGAGGAAATCACCGCGGACATCCCGAACGTGGGTGAGGCTGCGCTGAACAAGCTGGACGAGGCCGGTATCGTTTACGTCG
GTGCCGAAGTACAGGCTGGCGACATCCTGGTCGGCAAGGTCACCCCGAAAGGCGAAACCCAGCTGACTCCGGAAGAGAAGCTGC
TGCGTGCGATCTTCGGCGAGAAGGCGTCCGACGTGAAGGACACTTCCCTGCGCGTGCCGACCGGCACCAAGGGCACCGTCATCGA
CGTGCAGGTCTTCACCCGCGATGGCGTCGAACGCGACTCGCGCGCCCTGTCCATCGAGAAGATGCAGCTGGATGAGATCCGCAAG
GACCTGAACGAGGAGTTCCGTATCGTCGAAGGCGCCACCTTCGAGCGTCTGCGTTCCGCTCTTGTCGGCCAGGTTGCCGAAGGCG
GCGCGGGCCTGAAGAAGGGCACCGAAATCACCGACGAGTACCTCGATGGTCTCGAGCGCGGCCAGTGGTTCAAGCTGCGCATGT
CGGAAGACGCTCTTAACGAGCAGCTGGAGAAGGCACAGGCCTACCTGTCCGACCGTCGCCAGATGCTGGACGACAAGTTCGAAG
ACAAGAAGCGCAAGCTGCAGCAGGGCGACGACCTGGCTCCGGGCGTGCTGAAGATCGTCAAGGTCTACCTGGCCATCAAGCGTC
GCATCCAGCCGGGCGACAAGATGGCGGGCCGTCACGGTAACAAGGGTGTGGTCTCCGTGATCATGCCGGTCGAAGACATGCCGC
ACGACGCCAATGGCACTCCGGTGGATATCGTTCTGAACCCGCTGGGCGTACCGTCTCGTATGAACGTCGGTCAGATCCTCGAAAC
TCACCTGGGCCTCGCAGCCAAGGGCCTGGGCGAGAAGATCAACCGCATGATGGAAGAGCAGCGCAAGATTGCTGAACTGCGTGA
GTTCCTCCACGAGATCTACAACGAGATCGGTGGCCGTCAGGAAAACCTGAACGAGCTGAGTGACCAGGAAATCCTGGACCTGGC
GAAGAACCTGAAGGGCGGCGTACCCATGGCCACCCCGGTGTTCGACGGCGCCAAGGAAAGCGAGATCAAGGCCATGCTGAAGCT
CGCTGACTTGCCGGAGAGCGGTCAGATGCGCCTGTTCGACGGTCGTACCGGCAATCAGTTCGAGCGTCCGACCACCGTCGGCTAC
ATGTACATGCTCAAGCTGAACCACCTGGTCGACGACAAGATGCACGCACGTTCCACCGGTTCCTACAGCCTGGTTACCCAGCAGC

CGCTGGGTGGTAAGGCGCAGTTCGGTGGCCAGCGTTTCGGGGAGATGGAGGTCTGGGCACTGGAAGCCTACGGCGCCGCCTACA
CCCTGCAGGAAATGCTGACCGTGAAGTCGGACGACGTGAACGGCCGGACCAAGATGTACAAGAACATCGTGGACGGCGATCACC
GCATGGAGGCCGGCATGCCCGAGTCCTTCAACGTGCTGATCAAAGAGATCCGCTCGCTCGGCATCGACATCGAACTGGAAACCGA
ATAA

P. lundensis 2T.2.5.2_GCF_015377145.2_CP062158.2

ATGGCTTACTCATATACTGAGAAAAAACGTATCCGCAAGGACTTTAGCAAGTTGCCGGACGTCATGGATGTGCCGTATCTCTTGGC
AATCCAGCTGGATTCGTATCGTGAATTCTTGCAGGCGGGAGCGACTAAAGATCAGTTCCGCGACGTGGGCCTGCATGCGGCCTTC
AAATCCGTTTTCCCGATCATCAGCTACTCCGGCAATGCTGCGCTGGAGTATGTGGGTTATCGTTTGGGTGAACCGGCATTTGATGT
CAAAGAATGCGTGTTGCGCGGTGTAACTTACGCCGTACCTTTGCGGGTTAAAGTTCGCTTGATCATTTTCGACAAAGAATCGTCGA
ACAAAGCGATCAAGGACATCAAAGAGCAAGAAGTCTACATGGGCGAAATCCCATTGATGACTGAAAACGGTACCTTCGTTATTA
ACGGTACCGAGCGTGTAATTGTTTCCCAGCTGCACCGTTCCCCGGGCGTGTTCTTCGACCACGACCGCGGCAAGACGCACAGCTC
CGGTAAGCTGCTGTATTCCGCGCGTATCATTCCTTACCGTGGTTCGTGGTTGGATTTCGAGTTCGACCCGAAAGACTGCGTATTCG
TACGTATCGACCGTCGTCGCAAATTGCCTGCGTCCGTGCTTCTGCGCGCATTGGGTTACACCACTGAACAGGTGCTTGAAGCGTTC
TACACCACGAACGTATTCCACGTTCAAGGTGAGAGCATCAGCCTGGAGCTGGTACCACAGCGCCTGCGCGGTGAAATCGCGGTCA
TCGATATTATGGATGACAAGGGCAAAGTGATTGTCGAGCAAGGCCGTCGTATTACTGCTCGCCATATCAACCAGCTGGAAAAAGC
CGGTGTCAAAGAGCTCGTAATGCCTCTTGACTATGTCCTGGGTCGCACAACGGCCAAGGCAATCGTGCATCCGGCTACCGGCGAA
ATCATTGCTGAGTGCAACACAGAGCTGACCACTGAAATTCTGGCAAAAGTTGCCAAGAGTCAGGTTGTCCGTATCGAAACGTTGT
ACACCAACGACATCGACTGTGGTCCGTTCGTTTCTGACACGCTGAAAATTGATTCCACCAGCAACCAATTGGAAGCGCTGGTCGA
AATCTATCGCATGATGCGTCCAGGCGAGCCGCCAACCAAAGACGCTGCCGAGACCCTGTTCAACAACCTGTTCTTCAGCCCTGAG
CGTTATGACCTGTCTGCGGTCGGCCGAATGAAGTTCAACCGTCGTATCGGTCGTACCGAAATCGAAGGTTCGGGTGTGCTGTGCA
AGGAAGACATCGTTGCAGTGTTGAAGACTCTGGTCGACATCCGTAACGGTAAAGGCATCGTCGATGACATCGACCACTTGGGTAA
CCGTCGTGTTCGCTGCGTGGGCGAGATGGCTGAAAACCAGTTCCGTGTTGGCCTGGTGCGTGTTGAGCGTGCAGTTAAAGAGCGT
CTGTCGATGGCAGAAAGCGAAGGCCTGATGCCGCAAGACCTCATCAACGCCAAGCCAGTTGCTGCGGCGGTGAAAGAGTTCTTTG
GCTCCAGCCAGCTTTCGCAGTTCATGGACCAAAACAACCCGCTTTCCGAAATCACCCACAAGCGCCGTGTCTCTGCACTGGGCCC
GGGTGGTTTGACACGTGAGCGTGCTGGCTTTGAAGTTCGTGACGTACACCCGACTCACTACGGTCGTGTATGCCCGATTGAAACAC
CGGAAGGTCCGAACATCGGTCTGATCAACTCATTGGCTGCTTACGCCCGCACCAACCAGTATGGTTTCCTTGAGAGCCCGTACCGT
GTTGTGAAAGAAGGCGTAGTAACCGACGAGATCGTGTTCCTGTCTGCCATTGAAGAAGCAGATCACGTTATTGCTCAGGCTTCGG
CCACCATGAACGATCAGAAAGTCCTGATCGATGAGCTGGTAGCTGTTCGTCACTTGAACGAATTCACCGTTAAGGCGCCGGAAGA
CGTCACCTTGATGGACGTTTCGCCGAAGCAGGTAGTTTCGGTTGCTGCGTCGCTGATTCCGTTCCTCGAGCATGACGATGCCAACC
GTGCATTGATGGGTTCGAACATGCAGCGTCAAGCTGTACCGACACTGCGTGCTGACAAGCCGCTGGTGGGTACGGGTATGGAGCG
CAACGTAGCGCGAGACTCGGGGGTTTGTGTTGTAGCCCGTCGTGGTGGCGTTATTGACTCCGTGGATGCAAGTCGTATCGTTGTTC
GCGTTGCAGATGATGAAGTTGAAACCGGTGAAGCCGGTGTCGACATCTACAACCTGACCAAGTACACGCGCTCCAACCAGAACA
CCTGCATCAACCAGCGTCCGCTGGTTCGCAAAGGTGATCGGGTCCAGCGTAGTGACATCATGGCCGACGGTCCGTCCACCGACAT
GGGTGAACTGGCTCTGGGTCAGAACATGCGTATTGCCTTCATGGCATGGAACGGCTTCAACTTCGAAGACTCCATCTGCCTGTCGG
AACGTGTCGTTCAGGAAGACCGTTTCACCACGATCCACATTCAGGAACTGACCTGTGTGGCTCGTGACACCAAGCTTGGTCCAGA
GGAAATCACTGCAGACATCCCGAACGTCGGTGAAGCTGCTCTGAATAAACTGGATGAGGCCGGTATCGTTTATGTGGGTGCTGAA
GTCGGCCCGGGCGACATTCTGGTTGGTAAAGTGACACCGAAAGGTGAGACGCAGCTAACGCCAGAAGAGAAGCTGTTGCGCGCA
ATCTTCGGTGAAAAAGCCAGTGACGTAAAAGACACTTCTCTGCGTGTGCCGACCGGCACCAAAGGTACTGTCATTGACGTTCAGG
TTTTCACCCGTGACGGCGTTGAGCGTGACGCTCGTGCACTCTCGATCGAAAAAACCCAGCTGGACGAAATCCGCAAGGATCTGAA
CGAAGAGTTCCGTATCGTTGAAGGTGCAACTTTCGAACGTCTGCGCTCTGCATTGGTGGGTAACATTGCCGAAGGCGGTGCTGGTC
TCAAGAAAGGCCAGGAAATCACCAATGAGGTCCTGGATGGTCTTGAGCATGGTCAGTGGTTCAAGCTGCGCATGGCTGAAGATGC
TCTGAACGAGCAACTTGAAAAGGCTCAGGCTTACATCATCGACCGTCGTCGTCTGCTGGATGACAAGTTCGAAGATAAGAAACGC
AAGCTGCAGCAAGGCGATGACCTGGCTCCAGGCGTGTTGAAAATCGTCAAGGTTTACCTGGCAATTCGCCGTCGCATCCAGCCGG
GTGACAAGATGGCTGGTCGTCACGGTAACAAAGGTGTGGTCTCCGTGATCATGCCGGTTGAAGACATGCCGTACGATGCCAATGG
CACCCCGGTTGATGTTGTCCTCAACCCGTTGGGCGTACCATCGCGTATGAACGTTGGTCAGATTCTTGAAACTCACCTGGGCCTCG
CAGCCAAAGGGTTGGGCGAGAAGATCAACCTCATGATCGAGGAGCAGCGCAAGGTTGCAGACCTGCGTAAGTTCCTGCATGAGA
TCTACAACGAAATTGGCGGTCGTCAAGAAAGCCTGGATGACTTCTCTGATCAGGAAATTCTTGATCTGGCGAAGAACCTTCGCGG
CGGTGTTCCTATGGCTACCCCGGTATTTGACGGTGCCAAGGAAAGCGAAATCAAGGCCATGCTGCGCTTGGCAGATCTGCCCGAC
AGCGGCCAGATGCAGCTGACTGACGGTCGTACCGGCAACAAGTTTGAGCGTCCGGTTACCGTTGGCTACATGTACATGCTGAAAC
TGAACCACTTGGTAGACGACAAGATGCATGCTCGTTCTACCGGTTCTTACAGCCTGGTTACTCAACAGCCGTTGGGTGGTAAGGCG
CAGTTCGGTGGTCAGCGTTTCGGGGAGATGGAGGTCTGGGCGCTGGAAGCCTATGGCGCGGCATACACTCTGCAAGAAATGCTCA
CAGTGAAGTCGGACGATGTGAACGGTCGTACCAAGATGTACAAAAACATCGTGGACGGCGATCACCGTATGGAGCCGGGCATGC
CCGAGTCCTTCAACGTGTTGATCAAAGAAATTCGTTCCCTCGGCATCGATATCGATCTGGAAACCGAATAA

P. lurida MYb11_GCF_002966835.1_CP023272.1

ATGGCTTACTCATATACTGAGAAAAAACGTATCCGCAAGGACTTTAGCAAGTTGCCGGACGTCATGGATGTCCCGTACCTTCTGGC
TATCCAGCTGGATTCGTATCGTGAATTCTTGCAGGCGGGAGCGACCAAAGATCAGTTCCGCGACGTGGGCCTGCATGCGGCCTTC
AAATCCGTTTTCCCGATCATCAGCTACTCCGGCAATGCTGCGCTGGAGTACGTGGGTTATCGCCTGGGCGAACCGGCATTTGATGT
CAAAGAATGCGTGTTGCGCGGTGTTACGTACGCCGTACCTTTGCGGGTAAAAGTCCGCCTGATCATTTTCGACAAAGAATCGTCG
AACAAAGCGATCAAGGACATCAAAGAGCAAGAAGTCTACATGGGCGAAATCCCATTGATGACTGAGAACGGTACCTTCGTTATC
AACGGTACCGAGCGTGTGATCGTTTCCCAGCTGCACCGTTCCCCGGGCGTGTTCTTCGACCACGACCGCGGCAAGACGCACAGCT
CCGGCAAGCTCCTGTACTCCGCGCGGATCATTCCGTACCGTGGTTCGTGGTTGGACTTCGAGTTCGACCCGAAAGACTGCGTGTTC
GTGCGTATCGACCGTCGTCGCAAGCTGCCGGCCTCGGTACTGCTGCGCGCGCTCGGCTATACCACTGAGCAAGTGCTGGACGCCT

TCTACACCACCAACGTATTCAGCCTGAAGGATGAAACCCTCAAGCTGGAGCTGATCGCTTCGCGTCTGCGTGGTGAAATTGCCGT
CCTGGACATCCAGGATGAAAAAGGCAAGGTCATTGTTGAAGCTGGCCGTCGTATCACTGCGCGCCACATCAACCAGATCGAAAA
AGCCGGTATCAAGGAACTGGAAGTGCCTCTGGACTACGTCCTGGGTCGCACTACCGCCAAGGTCATCGTTCACCCGGCTACAGGC
GAAATCCTGGCTGAGTGCAACACCGAGCTGAACACCGAGATCCTGGCCAAAATCGCCAAGGCCCAGGTTGTTCGTATCGAGACCC
TGTACACCAACGACATCGACTGCGGTCCGTTCATCTCCGACACACTGAAGATCGACTCCACCAGCAACCAATTGGAAGCGCTGGT
CGAGATCTATCGCATGATGCGTCCTGGCGAGCCACCGACCAAAGACGCTGCCGAAACCCTGTTCAACAACCTGTTCTTCAGCCCT
GAGCGCTATGACCTGTCTGCGGTCGGCCGGATGAAGTTCAACCGTCGTATCGGTCGTACCGAGATCGAAGGTTCGGGCGTGTTGT
GCAAGGAAGATATCGTTGCGGTCCTGAAGACCCTGGTCGACATCCGTAACGGCAAAGGCATCGTCGATGACATCGACCACCTGGG
TAACCGTCGTGTTCGCTGCGTAGGCGAAATGGCCGAGAACCAGTTCCGCGTTGGCCTGGTACGTGTTGAGCGTGCGGTCAAAGAG
CGTCTGTCGATGGCTGAAAGCGAAGGCCTGATGCCGCAAGACCTGATCAACGCCAAGCCAGTGGCTGCGGCGGTGAAAGAGTTC
TTTGGTTCCAGCCAGCTTTCCCAGTTCATGGACCAGAACAACCCGCTCTCCGAGATCACCCACAAGCGCCGTGTATCCGCACTGGG
CCCGGGCGGTCTGACCCGTGAGCGTGCTGGCTTTGAAGTTCGTGACGTACACCCGACGCACTATGGTCGTGTTTGCCCGATCGAA
ACGCCGGAAGGTCCGAACATCGGTCTGATCAACTCCCTGGCCGCTTATGCGCGCACCAACCAGTACGGCTTCCTCGAGAGCCCGT
ACCGCGTGGTGAAAGACGCTCTGGTCACCGACGAGATCGTATTCCTGTCCGCCATCGAAGAAGCAGATCACGTGATCGCTCAGGC
TTCGGCCACGATGAACGACAAGAAAGTCCTGATCGACGAACTGGTAGCTGTTCGTCACCTGAACGAGTTCACCGTCAAGGCGCCG
GAAGACGTCACCTTGATGGACGTTTCGCCGAAGCAGGTAGTTTCGGTTGCAGCGTCGCTGATCCCGTTCCTGGAGCACGATGACG
CCAACCGTGCGTTGATGGGTTCCAACATGCAGCGTCAAGCTGTACCCACCCTGCGCGCTGACAAGCCGCTGGTAGGTACCGGCAT
GGAGCGTAACGTAGCTCGTGACTCCGGCGTTTGCGTCGTGGCTCGTCGTGGCGGCGTGATCGATTCTGTGGATGCTAGCCGTATCG
TGGTTCGTGTTGCCGATGACGAAGTAGAAACTGGCGAAGCCGGTGTCGACATCTACAACCTGACCAAATACACCCGCTCGAACCA
GAACACCTGCATTAACCAGCGTCCGCTGGTGAGCAAGGGTGATCGCGTTCAGCGCAGCGACATCATGGCTGACGGCCCGTCCACC
GACATGGGTGAACTGGCGCTGGGTCAGAACATGCGCATCGCGTTCATGGCGTGGAACGGCTTCAACTTCGAAGACTCCATCTGCC
TGTCCGAGCGTGTTGTTCAGGAAGACCGTTTCACCACGATCCACATTCAGGAACTGACCTGTGTGGCCCGTGACACCAAGCTTGG
GCCAGAGGAAATCACTGCAGACATCCCGAACGTGGGTGAAGCTGCACTGAACAAGCTGGACGAAGCCGGTATCGTTTACGTAGG
TGCTGAAGTAGGCGCGGGCGACATCCTGGTTGGTAAGGTCACTCCGAAAGGCGAGACCCAGCTGACTCCGGAAGAGAAGCTGTT
GCGCGCGATCTTCGGTGAAAAAGCCAGCGACGTTAAAGACACCTCCCTGCGCGTACCTACCGGTACCAAAGGTACTGTCATCGAC
GTACAGGTCTTCACCCGTGACGGCGTTGAGCGTGATGCTCGTGCACTGTCCATCGAGAAGACCCAGCTCGACGAGATCCGCAAGG
ACCTCAACGAAGAGTTCCGTATCGTTGAAGGTGCGACCTTCGAACGTCTGCGTTCCGCCCTGGTAGGCCATAAGGCCGAAGGTGG
CGCAGGCCTGAAGAAAGGTCAGGACATCACCGACGAAGTCCTCGACGGTCTTGAGCACGGCCAGTGGTTCAAACTGCGCATGGC
TGAAGATGCTCTGAACGAGCAGCTCGAGAAGGCCCAGGCCTACATCGTTGATCGTCGCCGTCTGCTGGACGACAAGTTCGAAGAC
AAGAAGCGCAAACTGCAGCAGGGCGATGACCTGGCTCCAGGCGTGCTGAAAATCGTCAAGGTTTACCTGGCAATCCGTCGCCGC
ATCCAGCCGGGCGACAAGATGGCCGGTCGTCACGGTAACAAAGGTGTGGTCTCTGTGATCATGCCGGTTGAAGACATGCCGCACG
ATGCCAATGGCACCCCGGTCGACGTCGTCCTCAACCCGTTGGGCGTACCTTCGCGTATGAACGTTGGTCAGATCCTTGAAACCCAC
CTGGGCCTCGCGGCCAAAGGTCTGGGCGAGAAGATCAACCGTATGATCGAAGAGCAGCGCAAGGTCGCAGACCTGCGTAAGTTC
CTGCACGAGATCTACAACGAGATCGGCGGTCGCAACGAAGAGCTGGACACCTTCTCCGACCAGGAAATCCTGGACCTGGCGAAG
AACCTGCGCGGCGGCGTTCCGATGGCTACCCCGGTGTTCGACGGTGCCAAGGAAAGCGAAATCAAGGCCATGCTGAAACTGGCA
GACCTGCCAGAAAGCGGCCAGATGCAGCTGTTCGACGGACGTACCGGCAACAAGTTTGAGCGCCCGGTTACTGTTGGCTACATGT
ACATGCTGAAGCTGAACCACTTGGTAGACGACAAGATGCACGCTCGTTCTACCGGTTCGTACAGCCTGGTTACCCAGCAGCCGCT
GGGTGGTAAGGCTCAGTTCGGTGGTCAGCGTTTCGGGGAGATGGAGGTCTGGGCACTGGAAGCATACGGTGCTGCTTACACTCTG
CAAGAAATGCTCACAGTGAAGTCGGACGATGTGAACGGTCGGACCAAGATGTACAAAAACATCGTGGACGGCGATCACCGTATG
GAGCCGGGCATGCCCGAGTCTTTCAACGTGTTGATCAAAGAAATTCGTTCCCTCGGCATCGATATCGATCTGGAAACCGAATAA

P. mandelii JR-1_GCF_000257545.3_CP005960.1

ATGGCTTACTCATATACTGAGAAAAAACGTATCCGCAAGGACTTTAGCAAGTTGCCGGACGTCATGGATGTGCCGTACCTCCTGG
CCATCCAGCTGGATTCGTATCGTGAATTCTTGCAAGCGGGAGCGACTAAAGATCAGTTCCGCGACGTGGGCCTGCATGCGGCCTT
CAAATCCGTTTTCCCGATCATCAGCTACTCCGGCAATGCTGCGCTGGAGTACGTCGGTTATCGCCTGGGCGAACCGGCATTTGATG
TCAAAGAATGCGTATTGCGCGGTGTGACTTACGCCGTACCTTTGCGGGTAAAAGTGCGCCTGATCATTTTCGACAAAGAATCGTCG
AACAAAGCGATCAAGGACATTAAAGAGCAAGAAGTCTACATGGGTGAGATCCCCCTGATGACTGAGAACGGTACCTTCGTAATC
AACGGTACCGAGCGTGTAATCGTTTCCCAGCTGCACCGTTCCCCGGGCGTGTTCTTCGACCACGACCGTGGCAAGACGCACAGCT
CCGGCAAACTGCTGTACTCCGCGCGCATCATTCCTTACCGCGGTTCGTGGCTGGACTTCGAGTTCGACCCGAAAGACTGCGTGTTC
GTGCGTATCGACCGTCGTCGCAAGCTGCCTGCATCGGTACTGCTGCGCGCGCTCGGCTATACCACTGAAGAAGTGCTCGACGCGT
TCTACACCACCAACGTTTTCCACCTGAGCGGCGAAACCCTCAGTCTGGAACTGATTGCTTCGCGTCTGCGTGGTGAAATCGCTGTT
CTTGATATTCAGGATGAGAAGGGCAAGGTCATCGTTGAGGCTGGTCGCCGTATTACTGCGCGCCACATCAACCAGATCGAAAAAG
CCGGCCTCAAGACCCTGGAAGTGCCTCTGGACTACGTCCTGGGTCGTACTACCGCCAAGGCCATCGTGCATCCGGCAACCGGCGA
AATCCTGGCAGAGTGCAACACCGAGCTGAACACCGAGATCCTGGCAAAAATTGCCAAGGCCCAGGTTGTTCGCATCGAAACTCTG
TACACCAACGATATCGACTGCGGTCCGTTTGTCTCCGACACTCTGAAGATCGACTCCACCAGCAACCAATTGGAAGCGCTGGTCG
AGATCTATCGCATGATGCGTCCAGGCGAGCCGCCAACCAAAGACGCTGCCGAGACTCTGTTCAACAACCTGTTCTTCAGCCCTGA
GCGCTATGACCTGTCTGCGGTCGGCCGGATGAAGTTCAACCGTCGTATCGGTCGTACCGAGATCGAAGGTTCGGGCGTGTTGTGC
AAAGAAGACATCGTCGCGGTACTGAAGACTCTGGTCGACATCCGTAACGGTAAAGGCATCGTCGATGACATCGACCACCTGGGTA
ACCGTCGTGTTCGCTGCGTAGGCGAAATGGCCGAGAACCAGTTCCGCGTTGGCTTGGTACGTGTTGAGCGTGCGGTCAAAGAGCG
TCTGTCGATGGCTGAAAGTGAAGGCCTGATGCCGCAAGACCTGATCAACGCCAAGCCAGTGGCTGCGGCGGTGAAAGAGTTCTTC
GGTTCCAGCCAGCTTTCCCAGTTCATGGACCAGAACAACCCGCTGTCCGAGATCACCCACAAGCGTCGTGTGTCTGCACTCGGCCC
TGGCGGTTTGACTCGTGAGCGTGCTGGCTTTGAAGTTCGTGACGTACACCCGACTCACTATGGTCGTGTATGCCCGATTGAAACGC
CGGAAGGTCCGAACATCGGTCTGATCAACTCCCTGGCAGCCTATGCGCGCACCAACCAGTACGGCTTCCTCGAGAGCCCGTACCG
TGTGGTGAAAGACGCTCTGGTCACCGACGAGATCGTGTTCCTGTCCGCCATCGAAGAAGCTGATCACGTGATCGCTCAGGCTTCG

GCCACGATGAACGACAAGAAAGTCCTGATCGACGAGCTGGTAGCTGTTCGTCACTTGAACGAATTCACCGTCAAGGCGCCTGAAG
ACGTCACCTTGATGGACGTATCGCCGAAGCAGGTAGTCTCGGTTGCTGCGTCGCTGATCCCGTTCCTCGAGCACGACGACGCCAA
CCGTGCGTTGATGGGTTCGAACATGCAGCGTCAAGCTGTACCAACTCTGCGCGCTGACAAGCCGCTGGTCGGTACCGGCATGGAG
CGTAACGTAGCCCGTGACTCCGGCGTTTGCGTCGTGGCTCGTCGTGGTGGCGTTATCGATTCCGTCGACGCCAGCCGTATCGTGGT
TCGAGTTGCTGATGACGAAGTTGAAACCGGCGAAGCTGGTGTCGACATCTACAACCTGACCAAGTACACCCGCTCCAACCAGAAC
ACCTGCATCAACCAGCGTCCGCTGGTGCGTAAAGGTGATCGGGTTCAGCGTAGCGACATCATGGCCGACGGTCCGTCCACCGATA
TGGGTGAGCTGGCTCTGGGTCAGAACATGCGCATCGCGTTCATGGCATGGAACGGCTTCAACTTCGAAGACTCCATCTGCCTGTCC
GAGCGTGTGGTTCAGGAAGATCGCTTCACCACGATCCACATTCAGGAACTGACCTGTGTGGCGCGTGACACCAAGCTTGGGCCAG
AGGAAATCACTGCAGACATCCCGAACGTGGGTGAAGCTGCACTGAACAAGCTGGACGAAGCCGGTATCGTTTACGTAGGTGCTG
AAGTAGGCGCAGGCGACATCCTGGTTGGTAAGGTCACTCCGAAAGGCGAGACCCAGCTGACTCCGGAAGAAAAACTGCTGCGTG
CCATCTTCGGTGAAAAAGCCAGCGACGTTAAAGACACCTCCCTGCGCGTGCCTACCGGCACCAAGGGTACCGTCATCGACGTACA
GGTCTTCACCCGCGACGGCGTTGAGCGTGATGCTCGTGCACTGTCGATCGAGAAGACTCAACTCGACGAGATCCGCAAGGACCTG
AACGAAGAGTTCCGTATCGTTGAAGGCGCCACTTTCGAACGTCTGCGTTCCGCTCTGGTCGGCCACAAAGCCGAAGGCGGCGCCG
GCCTGAAGAAAGGTCAGGACATCACCGACGAAGTTCTCGACGGTCTTGAGCATGGTCAGTGGTTCAAACTGCGCATGGCTGAAGA
TGCTCTGAACGAGCAGCTTGAGAAGGCTCAGGCCTACATCGTTGATCGCCGCCGTCTGCTGGACGACAAGTTCGAAGACAAGAAG
CGCAAACTGCAGCAGGGCGATGACCTGGCTCCAGGCGTGCTGAAAATCGTCAAGGTTTACCTGGCAATCCGTCGCCGCATCCAGC
CGGGCGACAAGATGGCCGGTCGTCACGGTAACAAGGGTGTGGTCTCCGTGATCATGCCGGTTGAAGACATGCCGCACGATGCCA
ATGGCACTCCGGTCGACGTCGTCCTCAACCCGCTGGGCGTACCTTCGCGTATGAACGTTGGTCAGATCCTTGAAACCCACCTGGGC
CTCGCGGCCAAAGGTCTGGGCGAGAAGATCAACCGGATGATCGAAGAGCAGCGCAAAGTCGCTGAGCTTCGTAAATTCCTCGAC
GAGATCTACAACCAGATCGGCGGTCGTAACGAAGATCTGGATAGCTTCTCCGACCAGGAAATCCTGGATCTGGCGAAGAACCTGC
GTGGCGGCGTTCCAATGGCCACTCCAGTGTTCGACGGCGCCAAGGAAAGCGAAATCAAGGCCATGCTGAAACTGGCAGACCTGC
CAGAAAGCGGCCAGATGCAGCTGACCGACGGCCGTACCGGCAACAAGTTCGAGCGCCCGGTTACTGTTGGCTACATGTACATGCT
GAAGCTGAACCACTTGGTAGACGACAAGATGCACGCTCGTTCTACCGGTTCGTACAGCCTGGTTACCCAGCAGCCGCTGGGTGGT
AAGGCGCAGTTCGGTGGTCAGCGTTTCGGGGAGATGGAGGTCTGGGCACTGGAAGCATACGGTGCTGCTTACACTCTGCAAGAAA
TGCTCACAGTGAAGTCGGACGATGTGAACGGCCGTACCAAGATGTACAAAAACATCGTGGATGGCGATCACCGTATGGAGCCGG
GCATGCCCGAGTCTTTCAACGTGTTGATCAAGGAAATTCGTTCCCTCGGCATCGATATCGATCTGGAAACCGAATAA

P. marincola YSy11_GCF_900682675.2_LR215729.2

ATGGCTTACTCATATACTGAGAAAAAACGTATCCGCAAGGACTTTAGCAAGTTGCCGGATGTCATGGATGTGCCTTATCTCCTGGC
CATCCAGCTGGATTCGTATCGCGAATTCCTGCAGGCGGGAGCGACCAAAGATCAGTTTCGCGATATTGGCTTGCATGCAGCCTTCA
AATCCGTTTTCCCGATTATCAGCTATTCCGGCAATGCTGCTCTGGAATATGTCGGTTATCGCCTGGGCGAACCGGCCTTTGACGTC
AAAGAGTGCGTACTTCGTGGCGTAACCTTTGCCGTACCGCTGCGGGTAAAAGTCCGTCTGATCATTTTCGACAAAGAATCGTCGA
ACAAAGCGATCAAGGACATTAAAGAGCAAGAAGTTTACATGGGGGAAATCCCCCTGATGACTGAGAACGGTACCTTCGTAATCA
ACGGTACCGAGCGCGTCATCGTCTCCCAGTTGCACCGTTCGCCGGGCGTGTTCTTCGATCACGACCGTGGCAAGACGCACAGCTCT
GGCAAACTGCTGTATTCAGCTCGCGTAATTCCTTACCGTGGCTCGTGGCTCGACTTCGAATTCGACCCGAAAGACGCCGTGTTCGT
ACGTATCGACCGTCGCCGCAAATTGCCGGCTTCCGTGCTGCTGCGTGCACTGGGTTACACCACTGAAGAAGTGCTGGACGCTTTCT
ATACCACCAACGTTTTCCACGTCACCGGCGAAAGCCTGCGTCTGGAACTGGTGCCAGAGCGTCTGCGCGGTGAAATTGCTGTACT
GGATATTGCTGACGACAAGGGCAAAGTCATTGTCGAGCAAGGCCGCCGTATTACTGCGCGCCACATCAACCAGTTGAACAAAGCC
GGTATCACCGAGCTCAACGTGCCTATCGATTACCTGCTTGGCCGTACTTCTGCCAAGGCAATCGTGCACCCGGCAACTGGCGAGA
TCCTGGTTGAATGCAACACTGAGCTGAGCGTTGAAGACCTGGCCAAGGTCGTCAAGGCGCAGGTTGTGCGCATTGAAACCCTGTA
CACCAACGATATCGATTGCGGTCCGTTCATCTCCGATACCCTGAAGATCGACTCGACCACCAATCAGCTCGAAGCGCTGGTTGAA
ATCTATCGCATGATGCGTCCAGGCGAGCCGCCAACCAAGGATGCTGCCGAGACTCTGTTCAACAACCTGTTCTTCAGCCCAGAGC
GTTATGATCTGTCGGCTGTTGGCCGGATGAAGTTCAACCGTCGTATCGGTCGCACCGAGATCGAAGGTTCTGGCGTACTCAACAA
AGAAGACATCGTCGAGGTTCTGAAGACCCTCGTGGATATCCGCAACGGCAAAGGCATCGTCGATGACATCGACCACCTGGGTAAC
CGTCGCGTGCGTTGTGTCGGCGAAATGGCCGAGAACCAGTTCCGTGTAGGTCTGGTACGTGTTGAGCGTGCGGTTAAAGAACGTC
TTTCGATGGCTGAAAGCGAAGGCTTGATGCCGCAGGACCTGATCAACGCCAAGCCAGTGGCTGCTGCGGTCAAAGAGTTCTTCGG
TTCCAGCCAGCTATCGCAGTTCATGGACCAGAACAACCCGCTGTCCGAGATCACCCACAAGCGCCGCGTTTCGGCTCTTGGCCCG
GGCGGTCTGACACGTGAGCGTGCAGGCTTCGAAGTCCGTGACGTACACCCGACTCACTACGGTCGTGTATGCCCGATTGAAACGC
CGGAAGGTCCGAACATTGGTCTGATCAACTCCCTGGCGGCCTATGCGCGTACCAACCAGTACGGCTTCCTTGAGAGCCCGTACCG
TGTGGTTAAAGACACATTGGTTACTGATGAGATCGTGTTCCTGTCTGCGATCGAAGAAGCAGACCACGTGATCGCTCAGGCATCG
GCAACGATGAACGACAAAGGTCAGCTGGTTGATGAGTTGGTGGCGGTACGTCACCTCAACGAATTCACCGTAAAAGCGCCTGAA
GACGTGACCCTGATGGACGTTTCGCCGAAGCAGGTAGTTTCGGTTGCAGCCTCCCTCATCCCGTTCCTCGAGCACGACGATGCTAA
CCGGGCCCTGATGGGTTCGAACATGCAGCGTCAGGCTGTTCCGACCCTGCGTTCGGACAAGCCGCTGGTAGGTACTGGTATGGAG
CGCAACGTAGCGCGCGACTCCGGTGTTTGCGTTGTTGCACGTCGTGGCGGTGTGATCGACTCGGTCGACGCCAGCCGTATCGTTGT
TCGTGTTAACGATGACGAAGTTGAAACTGGCGAAGCCGGTGTCGACATTTACAACCTGACCAAATACACCCGTTCTAACCAGAAC
ACCTGCATCAACCAGCGTCCGCTGGTGAGCAAAGGTGATCAGGTAGCACGCAGCGACATTCTCGCTGACGGCCCGTCGACCGACA
TGGGTGAACTGGCTTTGGGCCAGAACATGCGCGTCGCGTTCATGCCGTGGAACGGTTACAACTTCGAAGACTCCATCCTCCTTTCC
GAGCGTGTAGTACAAGAAGACCGCTTCACCACGATCCACATCCAGGAACTGACCTGTGTGGCCCGTGACACCAAGCTTGGGCCTG
AGGAAATCTCCTCGGACATCCCGAACGTGGGTGAAGCTGCACTGAACAAGCTGGACGAAGCAGGCATCGTGTATGTCGGCGCTG
AAGTTGGCCCGGGCGACATTCTGGTTGGTAAGGTAACGCCGAAAGGTGAGACCCAGCTGACGCCGGAAGAGAAGCTGTTGCGTG
CGATCTTCGGTGAGAAGGCATCTGACGTTAAAGATACTTCCCTGCGTGTGCCGACTGGCACCAAAGGTACAGTCATCGACGTACA
GGTCTTCACCCGTGATGGTGTAGAGCGCGATTCGCGTGCCCTGGCTATCGAGAAGATGCAACTGGACGAGATCCGTAAGGACCTT
AACGAAGAGTTCCGCATCGTTGAAGGCGCAACCTTCGAACGTCTGCGTTCTGCTCTCGTGGGTAAAGTTGTTGAAGGCGGTGCTG
GCCTGAAGAAAGGCGCCGAAATCACTGACGAATTCCTCGACGGTCTTGAGCGTGGCCAGTGGTTCAAGCTGCGCATGGCTGACGA

TGCACTGAACGAGCAGTTGGAAAAGGCTCAGGCCTACATCTCTGATCGCCGTCAGTTGCTCGACGACAAGTTCGAAGACAAGAAG
CGCAAGCTGCAGCAGGGCGATGACCTGGCTCCGGGCGTTCTGAAGATCGTTAAGGTTTACCTGGCAATCCGCCGTCGCATCCAGC
CGGGTGACAAGATGGCCGGTCGTCACGGTAACAAGGGTGTTGTCTCGGTGATCATGCCGGTTGAAGACATGCCTCACGATATTCA
CGGTACGCCGGTAGACATCGTGTTGAACCCGCTGGGTGTACCTTCGCGTATGAACGTGGGTCAGATCCTCGAAACTCACCTGGGC
CTCGCGGCTAAAGGTTTGGGCGAGAAGATCAACCTGATGCTCGAAGAGCAGCGCAAAGTCGCTGAACTGCGTCAATTCATGCAGC
AGATCTACAACGAGATCGGCGGCCGTCAGGAAAGTCTGGATGATCTTAGCGACACGGAAATCCTCAACCTGGCGAAGAACTTGC
GCGGCGGCGTGCCAATGGCTACTCCGGTGTTCGACGGTGCTAAAGAAGTTGAAATCAAGGCCATGCTGAAACTGGCAGACCTGCC
AGAAAGCGGCCAGATGCGACTGATCGATGGCCGTACCGGTAATCAGTTCGAGCGTCCAACTACCGTTGGCTACATGTACATGCTC
AAGCTGAACCACTTGGTTGACGACAAGATGCACGCGCGTTCTACCGGTTCTTACAGCCTGGTTACTCAGCAGCCGCTGGGTGGTA
AGGCGCAATTCGGTGGCCAGCGCTTCGGTGAGATGGAGGTGTGGGCACTCGAGTCGTATGGTGCTGCTTACACGCTGCAGGAAAT
GCTCACTGTGAAGTCGGACGATGTGAACGGCCGTACCAAGATGTACAAAAACATCGTGGACGGTGATCACCGTATGGAGCCAGG
CATGCCTGAGTCCTTCAACGTACTGATCAAAGAGATCCGTTCGCTCGGTATCGATATCGATCTGGAAACCGAATAA

P. mendocina S5.2_GCF_000733715.2_CP013124.1

ATGGCTTACTCATACACTGAGAAAAAACGTATCCGCAAGGACTTTAGCAAGTTGCCGGATGTCATGGATGTGCCTTACCTCCTGGC
CATCCAGCTGGATTCGTACCGCGAATTCCTGCAGCAAGGGGTGAGCAAGGAACAGTTCCGTGACATCGGCCTGCATGCGGCCTTC
AAATCCGTATTCCCGATCATCAGCTACTCCGGCAACGCCGCCCTGGAGTACGTCGGCTATCGCCTGGGCGAGCCGGCGTTCGACG
TCAAGGAGTGCGTCCTGCGTGGCGTGACCTTCGCCGTGCCGCTGCGCGTGAAAGTCCGTCTGATCATTTTCGACAAAGAATCGTCG
AACAAAGCGATCAAGGACATCAAAGAGCAGGAAGTGTACATGGGCGAAATTCCGCTCATGACCGAGAACGGTACCTTCGTCATC
AACGGTACCGAGCGCGTGATCGTGTCCCAGCTGCACCGTTCGCCGGGTGTGTTCTTCGACCACGACCGTGGCAAGACCCACAGCT
CGGGCAAGCTGCTGTACTCCGCGCGCATCATTCCTTACCGCGGTTCCTGGCTGGACTTCGAGTTCGACCCGAAGGACGCGGTCTTC
GTGCGTATCGACCGTCGCCGCAAACTGCCGGCGTCCGTTCTGCTGCGCGCATTGGGCTACAGCACCGAAGAAGTGCTGGATGCCT
TCTACGACACCAACGTCTACCACGTTAAGAACGAGAGCCTGAGCCTGGAGCTGGTGCCTCAGCGTCTGCGTGGTGAAGTCGCGGT
CCTGGACATCAAGGACGCCAGCGGCAAGGTAATCGTCGAGCAGGGCCGTCGTATCACTGCTCGTCACATCAACCAGCTGGACAA
GGCTGGCATCAAAGAGCTGGAAGTTCCGCTCGACTACGTCATTGGCCGTACCACTGCCAAGGCTATCGTGCACCCGGCTACCGGC
GAGATCATCGCCGAATGCAACACCGAGCTGACCGCCGACCTGCTGGTCAAGATGGCCAAGGCGCAGGTGGTTCGCTTCGAGACCC
TGTACACCAACGACATCGATTGTGGCCCGTTCATCAGCGACACGCTGAAGATCGACAGCACCACCAATCAGTTGGAAGCGCTGGT
CGAGATCTATCGCATGATGCGTCCTGGCGAGCCGCCAACCAAGGATGCTGCCGAGACCCTGTTCAACAACCTGTTCTTCAGCGCC
GAGCGTTACGATCTGTCTGCTGTCGGCCGCATGAAGTTCAACCGTCGTATCGGTCGTACCGAGATCGAAGGTTCGGGCGTGCTGA
GCAAGGAAGACATCGTTGCCGTACTGAAGACCCTGGTCGACATCCGTAACGGCAAAGGCATCGTCGACGACATCGACCACTTGG
GTAACCGTCGCGTGCGTTGCGTCGGCGAGATGGCCGAGAACCAGTTCCGCGTTGGCCTGGTGCGTGTAGAGCGTGCGGTCAAGGA
ACGTCTGTCGATGGCCGAAAGCGAAGGCCTGATGCCGCAGGACCTGATCAACGCCAAGCCGGTCGCGGCGGCGGTGAAGGAGTT
CTTCGGTTCCAGCCAGCTCTCGCAGTTCATGGACCAGAACAACCCGCTCTCCGAGATCACCCACAAGCGCCGCGTCTCCGCACTCG
GCCCGGGCGGTCTGACCCGTGAGCGCGCCGGCTTCGAAGTCCGCGACGTACACCCGACCCACTACGGCCGTGTGTGCCCGATCGA
AACGCCGGAAGGTCCGAACATCGGTCTGATCAACTCGCTGGCTGCCTACGCCCGCACCAACCAGTACGGCTTCCTGGAAAGCCCG
TACCGCGTGGTCAAGGAAGGTCAGGTCACCGACGAGATCGTGTTCCTGTCCGCTATCGAAGAAGCCGATCACGTGATCGCTCAGG
CGTCCGCGACCCTGAACGACAAGGGTCAGCTGATCGACGAGCTGGTCGCCGTACGTCACCTGAACGAATTCACCGTCAAGGCGCC
GGAAGACGTCACCCTGATGGACGTTTCGCCGAAGCAGGTAGTGTCGGTTGCCGCCTCGCTGATTCCGTTCCTTGAGCACGACGAC
GCCAACCGTGCACTCATGGGTTCGAACATGCAGCGTCAGGCTGTACCGACCCTGCGCGCTGACAAGCCGCTGGTAGGTACCGGCA
TGGAGCGCAACGTCGCTCGTGACTCTGGCGTCTGCGTCGTGGCTCGTCGTGGTGGAGTGATCGATTCCGTTGATGCCAGCCGCATC
GTGGTTCGTGTCAATGATGACGAAGTCGAAACTGGCGAAGCCGGTGTCGACATCTACAACCTGACCAAGTACACCCGCTCCAACC
AGAACACCTGCATCAACCAGCGCCCGCTGGTGAGCAAGGGTGATCAGGTATCGCGTGGCGACATCATGGCTGACGGCCCGTCCAC
TGACATGGGTGAACTGGCGCTGGGTCAGAACATGCGCGTCGCGTTCATGCCGTGGAACGGCTTCAACTTCGAAGACTCCATCTGC
CTGTCCGAGCGCGTGGTTCAGGAAGACCGCTTCACCACCATCCACATCCAGGAACTGACCTGTGTGGCGCGTGACACCAAGCTCG
GCCCAGAGGAAATCTCCTCTGATATCCCGAACGTCGGTGAAGCTGCTCTGAACAAGCTGGACGAAGCCGGTATCGTCTACGTCGG
TGCCGAAGTCGGCCCGGGCGACATTCTGGTCGGTAAGGTCACCCCGAAAGGCGAGACCCAGCTGACTCCGGAAGAGAAGCTGCT
GCGCGCGATCTTCGGTGAGAAGGCGTCCGACGTGAAGGACACCTCCCTGCGCGTGCCGACCGGCACCAAGGGCACCGTCATCGA
CGTGCAGGTCTTCACCCGTGATGGTGTCGAGCGCGACAGCCGCGCCCTGGCCATCGAGAAGCAGCAACTGGACGAGATCCGCAA
GGACCTGAACGAAGAGTTCCGCATCGTCGAAGGCGCGACCTTCGAGCGTCTGCGTTCGGCTTTGGTTGGCGCGATCGCCGAAGGT
GGCGCTGGCCTGAAGAAAGGCACCGCGATCACCGACGAGTTCCTCGACGGTCTCGAGCGTGGCCAGTGGTTCAAACTGCGCATGG
CCGACGACGCCCTGAACGAGCAGCTGGAGAAGGCCCAGGCCTATATCTCCGACCGCCGTCAGATGCTCGACGACAAGTTCGAAG
ACAAGAAGCGCAAGCTGCAGCAGGGCGATGACCTGGCTCCGGGCGTACTGAAGATCGTCAAGGTCTACCTGGCCATCCGCCGTC
GCATCCAGCCGGGTGACAAGATGGCCGGTCGTCACGGTAACAAGGGTGTGGTCTCGGTGATTATGCCGGTCGAAGACATGCCGCA
CGACGCCAACGGTACGCCGGTGGACATCGTACTGAACCCGCTGGGCGTACCGTCGCGTATGAACGTCGGTCAGATCCTCGAAACC
CACCTGGGCCTGGCGGCCAAGGGCCTGGGCGAGAAGATCAACCGCATGCTCGAAGAGCAGCGCAAGGTTGCCGAACTGCGCAAG
TTCCTCGCCGAGATCTACAACGAGATCGGTGGTCGTCAGGAAAACCTCGACGAGTTCTCCGACAACGAGATCCTCGAGCTGGCGA
AGAACCTCAAAGGCGGTGTACCGATGGCGACTGCCGTGTTCGACGGCGCCAAGGAAACCGAGATCAAGGCCATGCTGAAGCTGG
CCGATCTGCCGGAAAGCGGCCAGATGCGTCTGTTCGACGGTCGTACCGGTAACCAGTTCGAGCGCCCGACCACCGTCGGCTACAT
GTACATGCTGAAACTGAACCACCTGGTGGACGACAAGATGCACGCGCGTTCCACGGGTTCCTACAGCCTGGTTACCCAGCAGCCG
CTGGGTGGTAAGGCGCAGTTCGGTGGTCAGCGCTTCGGGGAGATGGAGGTCTGGGCGCTGGAAGCCTACGGCGCCGCCTACACCC
TGCAGGAAATGCTGACCGTGAAGTCGGACGACGTGAACGGCCGTACCAAGATGTACAAGAACATCGTGGATGGCGATCACCGTA
TGGAGCCGGGCATGCCCGAGTCCTTCAACGTACTGATCAAAGAGATCCGTTCGCTCGGCATCGACATCGATCTGGAAACCGAATA
A

P. monteilii B5_GCF_003671975.1_CP022562.1

ATGGCTTACTCATACACTGAGAAAAAACGTATCCGCAAGGACTTTAGCAAGTTGCCGGACGTCATGGATGTGCCTTACCTCCTGG
CCATCCAGCTGGATTCGTATCGCGAATTCCTGCAAGCGGGAGCATCCAAGGATCAGTTCCGCGACGTCGGCCTGCACGCGGCCTT
CAAATCGGTATTCCCGATCATCAGCTACTCCGGCAATGCTGCCCTGGAGTACGTAGGCTATCGCCTGGGCGAACCGGCCTTCGAT
GTGAAGGAATGTGTCCTGCGTGGCGTGACCTTCGCGGTCCCACTGCGGGTCAAGGTGCGCCTGATCATCTTCGACAAGGAATCGT
CGAACAAAGCGATCAAGGACATCAAAGAGCAAGAAGTCTACATGGGTGAAATCCCCCTGATGACTGAGAACGGTACCTTCGTTA
TCAACGGTACCGAGCGTGTGATCGTTTCCCAGCTGCACCGTTCGCCTGGTGTGTTCTTCGACCACGACCGTGGCAAGACCCACAGC
TCCGGCAAGCTGCTGTACTCCGCTCGCATCATCCCTTACCGCGGCTCCTGGCTGGACTTCGAGTTCGACCCGAAGGACTGCGTGTT
CGTGCGTATCGACCGTCGCCGCAAACTGCCGGCTTCGGTGCTGCTGCGTGCCCTGGGCTACAGCACTGAAGAAGTGCTGAACACC
TTCTACACCACCAACGTGTTCCACATTTCCGGCGAAAAGCTCAGCCTGGAACTGGTGCCTCAGCGTCTGCGTGGTGAAGTTGCAGT
CATGGATATCCATGACGAAACCGGCAAAGTCATCGTCGAGCAAGGCCGCCGTATTACTGCGCGCCACATCAACCAGCTCGAGAA
AGCCGGCGTCAAGCAGCTGGACGTTCCAATGGAATACGTCCTGGGCCGCACTACCGCCAAGGCCATCGTGCATCCGGCTACCGGC
GAGATCCTGGCCGAATGCAACACCGAGATGACCACCGAACTGCTGATCAAGGTCGCCAAGGCACAGGTTGTCCGTATCGAGACC
CTGTACACCAACGACATCGATTGCGGTCCGTTCATCTCCGACACCCTGAAGATCGACACCACCAGCAACCAGCTGGAAGCTCTGG
TCGAGATCTACCGCATGATGCGTCCAGGCGAGCCGCCAACCAAGGATGCAGCCGAGACCCTGTTCAACAACCTGTTCTTCAGCGC
CGAGCGTTACGACCTGTCTGCCGTTGGCCGCATGAAGTTCAACCGTCGTATCGGTCGTACCGAGATCGAAGGTTCGGGCGTGCTG
AGCAAGGAAGACATCGTCGAGGTTCTGAAGACCCTGGTCGATATCCGTAACGGCAAAGGCATCGTCGACGACATCGACCACCTC
GGTAACCGTCGCGTACGTTGCGTTGGTGAAATGGCCGAGAACCAGTTCCGCGTTGGCCTGGTGCGTGTAGAGCGCGCGGTCAAGG
AACGCCTGTCGATGGCGGAAAGCGAAGGCCTGATGCCGCAAGACCTGATCAACGCCAAGCCGGTTGCGGCGGCGGTGAAAGAGT
TCTTCGGTTCCAGTCAGCTGTCCCAGTTCATGGACCAGAACAACCCGCTCTCCGAGATCACCCACAAGCGCCGCGTCTCTGCACTC
GGCCCTGGCGGTCTGACCCGTGAGCGTGCCGGCTTCGAAGTCCGAGACGTACACCCGACCCACTACGGCCGTGTGTGCCCGATCG
AGACCCCTGAAGGTCCGAACATCGGTCTGATCAACTCCCTGGCAGCCTATGCCCGTACCAACCAGTACGGCTTCCTGGAAAGCCC
GTACCGCGTTGTGAAGGAAGGCGTTGTCAGCGACGACATCGTGTTCCTGTCGGCCATCGAAGAAGCCGATCACGTCATCGCCCAG
GCTTCGGCCGCGATGAACGAGAAGAAGCAACTGATCGATGAGCTGGTAGCGGTTCGTCACCTGAACGAATTCACCGTCAAGGCG
CCGGAAGACGTCACCCTGATGGACGTTTCGCCGAAGCAGGTTGTTTCCGTTGCTGCCTCGCTGATTCCGTTCCTCGAGCACGACGA
CGCCAACCGTGCATTGATGGGTTCGAACATGCAGCGTCAGGCTGTACCAACCCTGCGTGCCGACAAGCCGCTGGTAGGTACTGGC
ATGGAGCGCAACGTTGCCCGTGACTCCGGTGTCTGCGTGGTTGCTCGCCGTGGTGGTGTGATCGACTCGGTCGATGCCAGCCGTAT
CGTTGTTCGCGTTGCCGACGACGAAGTGGAAACCGGCGAAGCAGGTGTGGATATCTACAACCTGACCAAGTACACCCGTTCGAAC
CAGAACACCTGCATCAACCAGCGTCCGCTGGTGAGCAAAGGCGACAAGGTTCAGCGTGGTGACATCATGGCCGACGGCCCGTCC
ACCGACATGGGTGAGCTGGCACTGGGTCAGAACATGCGCATCGCGTTCATGGCGTGGAACGGCTTCAACTTCGAAGACTCCATCT
GCCTGTCCGAGCGTGTGGTTCAGGAAGACCGCTTCACCACCATCCACATCCAGGAACTGACCTGTGTGGCGCGTGACACCAAGCT
TGGCCCAGAGGAAATCACTGCGGACATCCCGAACGTGGGTGAAGCTGCACTGAACAAACTGGACGAAGCCGGTATCGTCTACGT
GGGTGCTGAAGTTGGCGCTGGTGACATCCTGGTTGGCAAGGTCACGCCAAAAGGCGAAACCCAGCTGACTCCGGAAGAAAAACT
GCTGCGTGCAATCTTCGGTGAGAAGGCCAGCGACGTTAAAGACACCTCCCTGCGCGTGCCAACCGGCACCAAGGGTACCGTCATC
GACGTACAGGTCTTCACCCGTGATGGCGTAGAGCGCGACAGCCGCGCCCTGGCCATCGAGAAGATGCAGCTGGACGAGATCCGC
AAGGACCTCAACGAAGAGTTCCGCATCGTCGAAGGCGCAACCTTCGAACGTCTGCGTTCTGCCCTGAACGGCCAGGTGGTCGACG
GTGGCGCGGGCCTGAAGAAAGGCACCGTGATCACTGACGAAGTCCTGGACGGCCTGGAGCACGGCCAGTGGTTCAAACTGCGCA
TGGCTGAAGATGCACTGAACGAGCAGCTGGAAAAGGCTCAGCAGTACATCGTCGACCGTCGCCGTCTGCTGGACGACAAGTTCG
AAGACAAGAAGCGCAAGCTGCAGCAGGGCGATGACCTGGCACCGGGCGTACTGAAGATCGTCAAGGTCTACCTGGCAATCCGCC
GTCGCATCCAGCCGGGTGACAAGATGGCCGGTCGTCACGGTAACAAGGGTGTTGTCTCGGTCATCATGCCGGTCGAAGACATGCC
GCACGATGCCAACGGTACTCCGGTCGACGTCGTACTGAACCCGCTGGGCGTACCTTCGCGTATGAACGTTGGTCAGATCCTTGAA
ACCCACCTGGGCCTGGCGGCCAAGGGCCTGGGCGAGAAGATCGACCGCATGCTCGAAGAGCAGCGCAAAGCCGCTGAACTGCGC
GTGTTCCTGACCGAGGTCTACAACGAGATCGGCGGTCGTCAGGAAAACCTCGAAGAGTTCACCGACGCAGAGATCCTGGCTCTGG
CCCACAACCTGAAGAAAGGCGTGCCGATGGCGACCCCGGTCTTCGATGGTGCCAAAGAGCGCGAGATCAAGGCCATGCTGAAAC
TGGCTGACCTGCCAGAAAGCGGCCAGATGGTGCTGTTCGATGGCCGTACCGGCAACAAGTTCGAGCGTCCTGTGACCGTTGGTTA
CATGTACATGCTCAAGCTGAACCACTTGGTGGACGACAAGATGCACGCGCGTTCCACTGGTTCCTACAGCCTGGTTACCCAGCAG
CCGCTGGGTGGTAAGGCGCAGTTCGGTGGTCAGCGTTTCGGGGAGATGGAAGTGTGGGCGCTGGAAGCATACGGCGCGGCATAC
ACCCTGCAAGAAATGCTCACAGTGAAGTCGGACGACGTGAACGGCCGTACCAAGATGTACAAAAACATCGTGGATGGCGATCAC
CGTATGGAGCCGGGCATGCCCGAGTCCTTCAACGTGTTGATCAAAGAGATCCGTTCGCTCGGTATCGATATCGATCTGGAAACCG
AATAA

P. multiresinivorans populi_GCF_012971725.1_CP048833.1

ATGGCTTACTCATACACTGAGAAAAAACGTATCCGCAAGGACTTTAGCAAGTTGCCGGACGTCATGGATGTGCCGTATTTGCTGG
CCATCCAGCTGGATTCCTATCGCGAATTCCTGCAGGCGGGTGCAAGCAAGGACCAGGTCCGAGACATCGGCCTGCATGCGGCCTT
CAAGTCCGTCTTCCCGATTATCAGCTATTCCGGCAATGCTGCCCTGGAATACGTCGGCTACCGTCTGGGTGAGCCGGCCTTCGATG
TCAAGGAATGCGTGCTGCGCGGTGTGACCTTTGCGGTCCCGCTGCGTGTGAAAGTGCGCCTGATCATTTTCGACAAGGAATCGTCG
AACAAAGCGATCAAGGACATCAAGGAACAGGAAGTCTACATGGGGGAAATCCCCCTGATGACCGAGAACGGTACCTTCATCATC
AACGGTACCGAGCGCGTCATCGTTTCCCAGTTGCACCGCTCCCCGGGTGTGTTCTTCGACCACGACCGTGGCAAGACCCACAGCTC
CGGCAAGCTGCTGTACTCCGCTCGGATCATTCCTTACCGCGGTTCCTGGCTCGACTTCGAGTTCGATCCGAAGGACTGCGTGTTCG
TTCGTATCGACCGTCGCCGCAAGCTGCCGGCTTCGGTACTGCTGCGCGCGCTGAACTACAGCACCGAGGAAATCCTCAACGCGTT

CTATGACACCAACGTCTACGAGATCAAGGGTGAGACCCTGAATCTGGAGCTGGTGCCGTCGCGTCTGCGTGGCGAGATCGCCAGC
TTCGATATCAAGGATGCTGCCGGCAAGGTCATCGTTGAAGCGGGTCGCCGTATTACCGCACGACACATCAACCAGCTCGAGAAGG
CCGGCATCAGCCAGCTGGAAGTGCCGTTCGACTACCTGATTGGCCGTACCGTGGCCAAGGCTGTCGTGCACCCGGCTACCGGTGA
GATCATCGCCGAGTGCAACACCGAGCTGACCGTCGATGCCCTGGCCAAGATCGCCAAGGCCCAGGTTGCGCGTCTCGAAACGCTG
TACACCAACGACATCGACTGCGGACCGTTCATCTCCGACACGCTGAAGATCGACTCCACCAGCAACCAACTGGAAGCGCTGGTCG
AGATCTATCGCATGATGCGTCCCGGCGAGCCGCCGACCAAGGAAGCCGCCGAGACCCTGTTCGGCAACCTGTTCTTCAGCGCCGA
GCGTTACGACCTGTCTGCCGTTGGCCGCATGAAGTTCAACCGTCGTATCGGTCGTACCGAGATCGAAGGCGCCGGCGTCCTGAGC
AAGGAAGACATCGTCGAGGTCCTCAAGACTCTGGTTGCCATCCGTAACGGTAAAGGCATCGTCGACGACATCGACCACCTGGGGA
ACCGTCGCGTTCGTTGCGTCGGTGAGATGGCCGAGAACCAGTTCCGCGTTGGCCTGGTGCGTGTAGAGCGCGCGGTCAAGGAACG
TCTGTCGATGGCCGAAAGCGAAGGCCTGATGCCGCAGGACCTGATCAACGCCAAGCCGGTGGCTGCTGCGATCAAGGAGTTCTTC
GGTTCCAGCCAGCTCTCGCAGTTCATGGACCAGAACAACCCGCTCTCCGAGATCACCCACAAGCGCCGTGTCTCTGCACTCGGCC
CAGGTGGTCTGACCCGTGAGCGCGCGGGCTTCGAGGTTCGTGACGTACACCCGACCCACTACGGCCGTGTGTGCCCGATCGAAAC
CCCTGAAGGTCCGAACATCGGTCTGATCAACTCCCTGGCCACCTACGCCCGCACCAACAAGTACGGCTTCCTGGAAAGCCCGTAC
CGCGTGGTGAAGGAAGGTCTGGTCAGCGACGACATCGTCTTCCTGTCCGCGATCGAAGAAGCTGACCACGTCATCGCTCAGGCGT
CTGCGACCCTGAACGAAAAAGGCCAGCTGATCGACGAACTGGTAGCCGTCCGTCACCTGAACGAATTCACCGTGAAGGCGCCGG
AAGACGTCACCCTCATGGACGTATCGCCGAAGCAGGTCGTTTCCGTCGCTGCCTCGCTGATTCCGTTCCTCGAGCACGACGACGCC
AACCGTGCACTCATGGGTTCGAACATGCAGCGTCAGGCTGTACCGACCCTGCGCGCCGACAAGCCGCTGGTAGGTACCGGCATGG
AGCGCAACGTCGCCCGTGACTCCGGCGTCTGCGTGGTTGCCCGTCGTGGCGGCGTGATCGACTCGGTCGACGCCAGCCGTATCGT
TGTGCGTGTGAACGACGACGAAGTCGAGACTGGCGAAGCCGGTGTCGACATCTACAACCTGACCAAGTACACCCGTTCCAACCAG
AACACCTGCATCAACCAGCGTCCGCTGGTGCAGAAGGGTGACCAGGTTTCGCGCAGCGACATCCTGGCCGACGGTCCGTCCACCG
ACATGGGTGAGCTGGCTCTGGGTCAGAACATGCGCGTCGCGTTCATGCCCTGGAACGGCTTCAACTTCGAAGACTCCATCTGCCT
GTCCGAGCGCGTGGTCCAGGAAGATCGTTTCACCACGATCCACATCCAGGAACTGACCTGCGTTGCCCGTGACACCAAGCTCGGC
CCAGAAGAAATCACCGCGGACATCCCGAACGTGGGTGAAGCTGCGCTGAACAAGCTGGACGAAGCTGGCATCGTCTACGTCGGC
GCCGAAGTACAGGCCGGCGACATCCTGGTCGGCAAGGTCACCCCGAAAGGCGAGACCCAGCTGACTCCGGAAGAGAAGCTGCTG
CGCGCGATCTTCGGTGAGAAGGCGTCCGACGTGAAGGACACCTCCCTGCGTGTGCCGACCGGCACCAAGGGCACCGTCATCGACG
TACAGGTCTTCACTCGCGATGGCGTGGAGCGCGATAGCCGTGCCCTGTCCATCGAGAAGATGCAGCTGGACGAGATCCGCAAGGA
CCTGAACGAAGAGTTCCGCATCGTCGAAGGCGCGACCTTCGAGCGTCTGCGTTCCGCTCTGGTCGGCTCCAAGGCCGACGGCGGT
CCTGCCCTGAAGAAAGGCGCAGAGATTACCGACGAGTACCTGGACGGTCTCGAGCGCGGCCAGTGGTTCAAGCTGCGCATGGCG
GAAGATGCTCTGAACGAGCAGTTGGAGAAGGCTCAGGCCTACATCAGCGATCGCCGCCAGATGCTGGACGACAAGTTCGAAGAC
AAGAAGCGCAAGCTGCAGCAGGGCGACGACCTGGCTCCGGGCGTACTGAAGATCGTCAAGGTCTACCTCGCTATCAAGCGCCGC
ATCCAGCCGGGCGACAAGATGGCAGGCCGCCACGGTAACAAGGGTGTGGTCTCGGTGATCATGCCGGTCGAAGACATGCCGCAC
GACGTGCATGGCACTCCGGTCGACATCGTTCTGAACCCGCTGGGCGTACCTTCGCGCATGAACGTCGGTCAGATCCTCGAAACCC
ACCTGGGCCTCGCGGCAAAGGGTCTGGGCGAGAAGATCAACCGCATGCTCGAAGAGCAGCGCAAGATCGCTGAGCTGCGTGTGT
TCCTGAACGAGATCTACAACGAGATCGGCGGCCGCCAGGAGAGCCTGGACGAGCTGAACGATAACGAGATCCTCGCTCTGGCCA
ACAACCTCAAGGGCGGCGTGCCCATGGCTACCCCGGTGTTCGACGGTGCCAAGGAACGCGAGATCAAGGCCATGCTGAAGCTGG
CCGATCTGCCGGAGAGCGGTCAGATGCGTCTGTACGACGGCCGCACCGGCAACCAGTTCGAGCGTACGACCACTGTTGGTTACAT
GTACATGCTCAAGCTGAACCACCTGGTCGATGACAAGATGCACGCACGTTCCACCGGCTCCTATAGCCTGGTTACCCAGCAGCCG
CTGGGTGGTAAGGCGCAGTTCGGTGGCCAGCGTTTCGGGGAGATGGAGGTCTGGGCGCTGGAAGCCTACGGTGCCGCCTACACCC
TGCAGGAAATGCTGACCGTGAAGTCGGACGACGTGAACGGCCGTACCAAGATGTACAAGAACATCGTGGACGGGGATCACCGCA
TGGAGGCCGGCATGCCCGAGTCCTTCAACGTGTTGATCAAAGAGATCCGCTCGCTCGGCATCGATATCGAACTGGAAACCGAATA
A

P. otitidis MrB4 DNA_GCF_011397855.1_AP022642.1

ATGGCTTACTCATACACTGAGAAAAAACGTATCCGCAAAGACTTTAGCAAGTTGCCGGACGTCATGGATGTACCCTACCTCCTGG
CCATCCAGCTGGATTCGTACCGCGAATTCCTGCAGGCGGGCGCGAGCAAGGAGCGGATCCGTGATATCGGCCTGCACGCGGCCTT
CAAGTCTGTATTCCCGATCATCAGCTATTCCGGCAACGCCGCTCTGGAATACGTTGGCTATCGCCTTGGCGAGCCGGCCTTCGACG
TCAAGGAATGCGTGCTGCGCGGCGTGACCTTCGCCGTTCCGCTGCGCGTCAAGGTGCGTCTGATCATTTTCGACAAGGAGTCGTCG
AACAAGGCCATCAAGGACATCAAGGAACAAGAAGTCTACATGGGCGAAATCCCGCTCATGACCGAGAACGGTACCTTCATCATC
AACGGTACCGAGCGCGTGATCGTCTCCCAGCTGCACCGCTCCCCGGGCGTGTTCTTCGACCATGACCGTGGCAAGACCCACAGCT
CCGGCAAGCTGCTGTACTCCGCGCGGATCATTCCCTACCGTGGTTCCTGGCTGGACTTCGAGTTCGATCCGAAGGACTGCGTATTC
GTTCGTATCGACCGTCGCCGCAAGCTGCCGGCCTCCGTCCTGCTGCGCGCGCTCGGCTACAGCACCGAGGAAGTGCTCAACGCCT
TCTATGCCACCAACGTCTTCCATGTGAAGGGCGAAGGCCTGCACCTGGAACTGGTGCCTCAGCGCCTGCGCGGTGAAGTCGCCGT
ATTCGACATCAAGGATCCGTCCGGCAAGGTGATCGTGGAGCAGGGCCGTCGTATCACCGCCCGCCACATCAACCAGCTGGAGAA
GGCTGGCATCAGCGAGCTGGAAGTTCCGCTGGACTACGTCCTCGGTCGCACCACCGCCAAGGCCATCGTGCATCCGGCCACCGGC
GAGATCATCGTCGAGTGCAATACCGAGCTGACCACCGACCTGCTGGTCAAGATCGCCAAGGCACAGGTCGTCCGCATCGAGACCC
TGTACACCAACGACATCGACTGCGGTCCGTTCATCTCCGACACCCTGAAGATCGACTCCACCAGCAACCAGCTGGAAGCCCTGGT
GGAAATCTATCGCATGATGCGTCCTGGTGAGCCGCCGACCAAGGAGGCTGCCGAGACCCTGTTCAACAACCTGTTCTTCAGCGCC
GAGCGTTACGACCTGTCCGCTGTCGGCCGCATGAAGTTCAACCGCCGGATCGGTCGCACCGAGATCGAGGGGCCTGGTGTCCTCA
GTCGCGAAGATATCGTCGAGGTCCTCAAGACCCTGGTCGACATCCGCAACGGCAAGGGCATCGTCGACGACATCGACCACCTGGG
TAACCGTCGTGTCCGTTGCGTTGGCGAAATGGCCGAAAACCAGTTCCGTGTTGGCCTGGTGCGCGTCGAGCGCGCGGTCAAGGAA
CGCCTGTCCATGGCGGAGAGCGAAGGCCTGATGCCTCAGGACCTGATCAACGCCAAGCCCGTGGCTGCTGCGATCAAGGAGTTCT
TCGGCTCCAGCCAGCTCTCCCAGTTCATGGACCAGAACAACCCGCTGTCCGAGATCACCCACAAGCGCCGCGTTTCCGCACTCGG
CCCAGGTGGTCTGACTCGCGAGCGCGCGGGCTTCGAAGTCCGCGACGTACACCCGACCCACTACGGCCGTGTGTGCCCGATCGAG
ACGCCCGAAGGTCCGAACATCGGTCTGATCAACTCCCTGGCTACCTACGCCCGCACCAACCAGTACGGTTTCCTCGAGAGCCCCT

ACCGTGTTGTAAAAGGCACCCAAGTGACCGATGAGATCGTCTTCCTCTCCGCCATCGAAGAGGCCGACCACGTCATCGCTCAGGC
GTCTGCCACGCTCAACGAGCAGGGCCAACTGATCGACGAACTGGTGGCTGTGCGTCACCTGAACGAGTTCACCGTGAAGGCGCCG
GAAGACGTCACCCTCATGGACGTATCGCCGAAGCAGGTCGTTTCCGTCGCTGCCTCGCTGATCCCGTTCCTCGAGCACGACGACG
CCAACCGCGCACTCATGGGCTCGAACATGCAGCGCCAGGCTGTGCCGACCCTGCGTGCCGACAAGCCTCTGGTCGGTACCGGCAT
GGAGCGCAACGTTGCCCGTGACTCCGGTGTCTGCGTCGTGGCCCGTCGTGGTGGCGTGATCGACTCTGTCGATGCCAGCCGCATC
GTCGTCCGTGTGAACGACGACGAAGTCGAGACTGGCGAAGCCGGTGTCGACATCTACAACCTGACCAAGTACACCCGTTCCAACC
AGAACACCTGCATCAACCAGCGTCCGCTGGTGCAGAAGGGTGACAAGGTTTCGCGCAGCGACATCCTGGCCGATGGTCCGTCCAC
CGACATGGGTGAACTGGCCCTGGGTCAGAACATGCGCGTCGCGTTCATGCCCTGGAACGGCTTCAACTTCGAAGACTCCATCTGC
CTCTCCGAGCGCGTGGTCCAGGAGGATCGTTTCACCACGATCCACATCCAGGAACTGACCTGCGTCGCCCGTGACACCAAGCTAG
GCCCAGAAGAAATCACCGCGGACATCCCGAACGTGGGTGAGGCTGCGCTGAACAAGCTGGATGAAGCCGGCATCGTCTACGTCG
GCGCCGAAGTACAGGCTGGCGACATCCTGGTCGGCAAGGTCACCCCGAAAGGCGAGACCCAGCTGACTCCGGAAGAGAAGCTGC
TGCGCGCGATTTTCGGTGAGAAGGCATCCGACGTTAAGGACACCTCCCTGCGTGTGCCGACCGGTACCAAGGGCACCGTCATCGA
CGTCCAGGTCTTCACCCGTGATGGCGTGGAGCGCGACTCCCGTGCTCTGTCCATCGAGAAGATGCAGCTCGACGAGATCCGTAAG
GACCTGAACGAAGAGTTCCGCATCGTCGAAGGTGCAACCTTCGAGCGCCTGCGTTCCGCCCTGGTCGGCCAGGTTGCAGAAGGTG
GCGCCGGCCTCAAGAAAGGCACCGAGATCACCGACGAGTACCTCGACGGCCTCGAGCGCGGCCAGTGGTTCAAGCTGCGCATGG
CGGACGACGCTCTGAACGAGCAGCTTGAGAAGGCCCAGGCCTACCTCTCCGACCGCCGCCAGCTGCTGGACGACAAGTTCGAAG
ACAAGAAGCGCAAGCTTCAGCAAGGCGACGACCTGGCGCCGGGCGTCCTGAAGATCGTCAAGGTGTACCTCGCCATCAAGCGCC
GCATCCAGCCGGGTGACAAGATGGCGGGCCGCCACGGTAACAAGGGTGTGGTCTCCGTGATCATGCCGGTCGAAGACATGCCCC
ACGATGCCAATGGCACGCCGGTGGACATCGTCCTCAACCCGCTGGGTGTACCGTCGCGTATGAACGTCGGCCAGATTCTCGAAAC
CCACCTGGGCCTTGCGGCCAAGGGCCTGGGCGAGAAGATCAACCGCATGCTCGAAGAGCAGCGCAAGATCGCCGAGCTGCGCAA
GTTCCTGCACGAGATCTACAACGAGATCGGTGGGCGTCAGGAGAGCCTGGATGAACTGAGCGATCAGGAAATCCTCGATCTGGCG
AAGAACCTGCGCGGCGGCGTGCCGATGGCTACCCCGGTGTTCGACGGTGCCAAGGAGCGCGAGATCAAGGCGATGCTGAAGCTC
GCCGATCTGCCCGAGAGCGGCCAGATGCGCCTGTTCGACGGTCGTACCGGCAACATGTTCGAGCGTCCGACCACCGTTGGCTACA
TGTACATGCTGAAGCTGAACCACCTGGTCGACGACAAGATGCACGCACGTTCCACCGGCTCTTACAGCCTGGTTACCCAGCAGCC
GCTGGGTGGTAAGGCGCAGTTCGGTGGCCAGCGTTTCGGGGAGATGGAGGTCTGGGCGCTGGAAGCCTACGGCGCCGCCTACAC
CCTGCAGGAGATGCTGACCGTGAAGTCCGACGACGTGAACGGCCGTACCAAGATGTACAAGAACATCGTGGATGGCGATCACCG
CATGGAGGCCGGCATGCCCGAGTCCTTCAACGTGTTGATCAAAGAGATCCGTTCGCTCGGCATCGACATCGAACTGGAAACCGAA
TAA

P. plecoglossicida XSDHY-P_GCF_003391255.1_CP031146.1

ATGGCTTACTCATACACTGAGAAAAAACGTATCCGCAAGGACTTTAGCAAGTTGCCGGACGTCATGGATGTGCCTTACCTCCTGG
CCATCCAGCTGGATTCGTATCGCGAATTCCTGCAAGCGGGAGCATCCAAGGATCAGTTCCGCGACGTCGGCCTGCACGCGGCCTT
CAAGTCGGTATTCCCGATCATCAGCTACTCCGGCAATGCTGCCCTGGAGTACGTAGGCTATCGCCTGGGCGAACCAGCCTTCGAT
GTGAAGGAATGTGTCCTGCGTGGCGTGACCTTCGCGGTCCCACTGCGGGTCAAGGTGCGCCTGATCATCTTCGACAAGGAATCGT
CGAACAAAGCGATCAAGGACATCAAAGAGCAAGAAGTCTACATGGGTGAAATCCCCCTGATGACTGAGAACGGTACCTTCGTTA
TCAACGGTACCGAGCGTGTAATCGTTTCCCAGCTGCACCGTTCGCCTGGTGTGTTCTTCGACCACGACCGTGGCAAGACTCACAGC
TCGGGCAAGCTGCTGTACTCCGCGCGCATCATTCCGTACCGCGGTTCGTGGCTGGACTTCGAGTTCGATCCGAAGGACTGCGTGTT
CGTGCGTATCGACCGTCGCCGCAAACTGCCGGCTTCGGTACTGCTGCGCGCGCTGGGCTACAGCACCGAAGAAGTACTGAACACC
TTCTACACCACCAACGTGTTCCACATTTCCGGCGAAAAACTGAGCCTGGAACTGGTACCTCAGCGTCTGCGTGGTGAAGTTGCGGT
CATGGACATCCATGACGGCAGCGGCAAGGTCATCGTCGAGCAAGGCCGCCGTATTACCGCGCGCCACGTCAACCAGCTCGAGAA
GGCCGGTGTCAACCAGCTGGACGTTCCAATGGAATACGTCCTGGGTCGCACCACTGCCAAGGCCATCGTGCATCCGGCTACCGGC
GAGATCCTGGCCGAGTGCAACACCGAGCTGACCACCGAGCTGCTGATCAAGATCGCCAAGGCTCAGGTCGTTCGCATCGAGACCC
TGTACACCAACGACATCGACTGCGGTCCGTTCATCTCGGATACCCTGAAGATCGACACCACCAGCAACCAACTGGAAGCGCTGGT
CGAGATCTACCGCATGATGCGTCCAGGCGAGCCGCCAACCAAGGACGCCGCCGAGACCCTGTTCAACAACCTGTTCTTCAGCGCC
GAGCGTTACGACCTGTCCGCCGTTGGCCGCATGAAGTTCAACCGTCGTATCGGTCGTACCGAGATCGAAGGTTCGGGCGTGCTGA
GCAAGGAAGATATCGTCGAGGTCCTCAAGACTCTGGTCGACATCCGTAACGGCAAAGGCATCGTCGACGACATCGACCATCTGGG
CAACCGTCGCGTACGTTGCGTCGGCGAGATGGCCGAGAACCAGTTCCGCGTTGGCCTGGTGCGTGTCGAGCGCGCGGTCAAAGAG
CGCCTGTCGATGGCAGAAAGCGAAGGCCTGATGCCGCAAGACCTGATCAACGCCAAGCCGGTTGCGGCGGCGGTGAAAGAGTTC
TTCGGCTCCAGCCAGCTCTCGCAGTTCATGGACCAGAACAACCCGCTCTCCGAGATCACCCACAAGCGCCGCGTCTCCGCACTCG
GCCCGGGCGGTCTGACCCGTGAGCGTGCAGGCTTCGAGGTTCGTGACGTACACCCGACCCACTACGGCCGCGTGTGCCCGATCGA
GACCCCTGAAGGTCCGAACATCGGTCTGATCAACTCCCTGGCAGCCTATGCCCGCACCAACCAGTACGGCTTCCTGGAAAGCCCG
TACCGCGTGGTGAAGGAAGGCGTGGTCAGCGACGACATCGTGTTCCTGTCGGCAATCGAAGAAGCCGATCACGTCATCGCCCAGG
CTTCTGCCGCGATGAACGAGAAGAAGCAGCTGATCGATGAGCTGGTAGCGGTCCGTCACCTGAACGAATTCACCGTCAAGGCGCC
GGAAGACGTCACCCTGATGGACGTTTCGCCGAAGCAGGTTGTTTCCGTTGCTGCCTCGCTGATCCCGTTCCTCGAGCACGACGACG
CCAACCGTGCATTGATGGGTTCGAACATGCAGCGTCAGGCTGTACCGACCCTGCGCGCGGACAAGCCGCTGGTAGGTACCGGCAT
GGAGCGCAACGTTGCCCGTGACTCCGGTGTCTGCGTGGTTGCTCGCCGCGGTGGTGTGATCGACTCGGTCGACGCCAGCCGTATC
GTTGTTCGTGTCAACGACGACGAAGTGGAAACCGGCGAAGCAGGTGTGGATATCTACAACCTGACCAAGTACACCCGTTCGAACC
AGAACACCTGCATCAACCAGCGTCCGCTGGTGAGCAAAGGTGACAAGGTTCAGCGTAGCGACATCATGGCCGACGGCCCGTCCA
CCGACATGGGTGAGCTGGCACTGGGCCAGAACATGCGCATCGCGTTCATGGCGTGGAACGGCTTCAACTTCGAAGACTCCATCTG
CCTGTCCGAGCGTGTGGTTCAGGAAGACCGCTTCACCACCATCCACATCCAGGAACTGACCTGTGTGGCGCGTGACACCAAGCTT
GGCCCAGAGGAAATCACTGCGGACATCCCGAACGTGGGTGAAGCTGCACTGAACAAGCTGGACGAAGCCGGTATCGTCTACGTA
GGTGCTGAAGTCGGCGCTGGCGACATTCTGGTCGGCAAGGTCACGCCAAAAGGCGAAACCCAGCTGACTCCGGAAGAAAAACTG
CTGCGCGCAATCTTCGGTGAGAAGGCCAGCGACGTTAAGGACACCTCCCTGCGCGTGCCAACCGGCACCAAGGGCACCGTCATCG
ACGTACAGGTCTTCACCCGTGATGGCGTCGAGCGCGACAGCCGCGCCCTGGCCATCGAGAAGATGCAGCTGGACGAGATCCGCA

AGGACCTCAACGAAGAGTTCCGCATCGTCGAAGGTGCAACCTTCGAGCGTCTGCGTGCTGCCCTGAACGGCCAGGTGGTCGACGG
TGGCGCGGGCCTGAAGAAAGGCACCGTGATCACTGACGAAGTGCTGGACGGCCTGGAGCACGGCCAGTGGTTCAAACTGCGCAT
GGCCGAAGATGCACTGAACGAGCAGCTGGAAAAGGCTCAGCAGTACATCGTCGATCGTCGCCGTCTGCTGGACGACAAGTTCGA
AGACAAGAAGCGCAAGCTGCAGCAGGGCGATGACCTGGCTCCGGGCGTACTGAAGATCGTCAAGGTCTACCTGGCAATCCGCCG
TCGCATCCAGCCGGGTGACAAGATGGCCGGTCGTCACGGTAACAAGGGTGTCGTCTCGGTGATCATGCCGGTGGAAGACATGCCG
CACGACGCCAACGGTACTCCGGTCGACGTCGTACTGAACCCGCTGGGCGTACCTTCGCGTATGAACGTCGGTCAGATTCTCGAAA
CCCACCTGGGCCTCGCGGCCAAGGGCCTGGGCGAGAAGATCGACCGCATGATCGAAGAACAGCGCAAAGCCGCTGAGCTGCGTG
GCTTCCTCACCGAGATCTACAACGAGATCGGTGGTCGTCAGGAGAACCTGGAAGAGTTCACCGACGAAGAGATCCTCGCCCTGGC
CAACAACCTGAAGAAAGGCGTGCCAATGGCAACTCCGGTCTTCGACGGTGCCAAGGAGCGTGAGATCAAGGCCATGCTGAAGCT
GGCAGACCTGCCAGAAAGCGGCCAGATGGTGCTGTTCGACGGCCGTACCGGCAACAAGTTCGAGCGTCCTGTGACCGTTGGTTAC
ATGTACATGCTCAAGCTGAACCACTTGGTGGACGACAAGATGCACGCGCGTTCCACTGGTTCCTACAGCCTGGTTACCCAGCAGC
CGCTGGGTGGTAAGGCGCAGTTCGGTGGTCAGCGTTTCGGGGAGATGGAAGTGTGGGCGCTGGAAGCATACGGCGCGGCATACA
CCCTGCAAGAAATGCTCACAGTGAAGTCGGACGACGTGAACGGCCGTACCAAGATGTACAAGAACATCGTGGATGGCGATCACC
GTATGGAGCCGGGCATGCCCGAGTCCTTCAACGTGTTGATCAAAGAGATCCGTTCGCTCGGTATCGATATCGATCTGGAAACCGA
ATAA

P. protegens CHA0_GCF_900560965.1_LS999205.1

ATGGCTTACTCATATACTGAGAAAAAACGTATCCGCAAGGACTTTAGCAAGTTGCCGGACGTCATGGATGTGCCGTACCTCCTGG
CCATCCAGCTGGATTCGTATCGTGAATTCTTGCAAGCGGGAGCGACTAAAGATCAGTTCCGCGACGTGGGCCTGCATGCGGCCTT
CAAATCCGTTTTCCCGATCATCAGCTACTCCGGCAATGCTGCGTTGGAGTATGTCGGTTATCGCCTGGGCGAACCGGCGTTTGATG
TTAAAGAATGCGTGTTGCGTGGTGTAACTTACGCCGTACCTTTGCGGGTAAAAGTCCGTCTGATCATTTTCGACAAAGAATCGTCG
AACAAAGCGATCAAGGACATCAAAGAGCAAGAAGTCTACATGGGTGAAATTCCCCTGATGACTGAGAACGGTACCTTCGTAATC
AACGGTACCGAGCGTGTAATCGTTTCCCAGCTGCACCGCTCCCCTGGCGTATTCTTCGACCACGACCGTGGCAAGACGCACAGCT
CCGGTAAGCTGCTGTATTCCGCGCGGATCATTCCTTACCGCGGTTCGTGGCTGGACTTCGAGTTCGACCCGAAAGACTGCGTGTTC
GTCCGTATCGACCGTCGTCGCAAGCTGCCGGCATCGGTTCTGCTGCGCGCGCTGGGCTATACCACTGAAGAAGTGCTGGACGCTTT
CTACACCACCAACGTATTCCACGTGAAAGGCGAGAGCCTGAGCCTGGAGCTGGTGCCTCAGCGCCTGCGTGGTGAGATTGCCGTT
CTGGACATCCAGGATGACAAGGGCAAGGTTATTGTCGAGCAGGGTCGTCGTATCACCGCTCGTCACATCAACCAGCTGGAAAAAG
CCGGTATCAAAGAGCTGGATGTACCTCTGGACTACGTCCTGGGTCGTACCACCGCCAAGGCTATCGTGCACCCGGCTACCGGTGA
AATCCTGGCAGAGTGCAACACCGAGCTGAACACCGAGATCCTGGCAAAAATCGCCAAGGCTCAGGTCGTTCGCATCGAGACTCTG
TACACCAACGACATCGACTGCGGTCCGTTCGTCTCTGACACTCTGAAGATCGACTCCACCAGCAACCAATTGGAAGCCCTGGTCG
AGATCTATCGCATGATGCGTCCTGGCGAGCCACCGACCAAAGATGCTGCCGAGACTTTGTTCAACAACCTGTTCTTCAGCCCTGAG
CGGTATGATCTGTCCGCCGTTGGCCGGATGAAGTTCAACCGTCGTATCGGTCGTACCGAGATCGAAGGTTCCGGCGTTCTGTGCAA
AGAAGACATCGTTGCCGTACTGAAGACTCTGGTCGACATCCGTAACGGTAAAGGCATTGTCGACGACATCGACCACCTGGGTAAC
CGTCGTGTTCGCTGTGTTGGCGAAATGGCCGAGAACCAGTTCCGCGTTGGCCTGGTGCGTGTAGAGCGTGCGGTCAAAGAGCGCC
TGTCGATGGCTGAAAGCGAAGGCCTGATGCCGCAAGACCTGATCAATGCCAAGCCAGTGGCTGCGGCAGTGAAAGAGTTCTTCGG
TTCCAGCCAGCTGTCCCAGTTCATGGACCAGAACAACCCGCTGTCCGAGATCACCCACAAGCGCCGTGTTTCTGCACTTGGCCCAG
GTGGTCTGACCCGTGAGCGTGCAGGCTTCGAAGTCCGTGACGTACACCCGACCCACTACGGTCGTGTATGCCCGATTGAAACGCC
GGAAGGTCCGAACATCGGTCTGATCAACTCCCTGGCGGCCTATGCGCGCACCAACCAGTACGGCTTCCTCGAGAGCCCGTACCGT
GTGGTGAAAGACGCTCTGGTAACCGACGAGATCGTGTTCCTGTCCGCTATCGAAGAGGCCGATCACGTTATCGCCCAGGCTTCGG
CGACGATGAACGACAAAGGTCAGTTGGTCGATGAGCTGGTAGCTGTTCGTCACTTGAACGAATTCACCGTCAAGGCGCCGGAAGA
CGTCACCTTGATGGACGTTTCGCCGAAGCAGGTAGTTTCGGTTGCAGCGTCGCTGATTCCGTTCCTCGAGCACGACGACGCCAACC
GTGCATTGATGGGTTCCAACATGCAGCGTCAGGCTGTACCGACCCTGCGTGCTGACAAGCCGCTGGTAGGTACCGGCATGGAGCG
CAACGTTGCCCGTGACTCCGGCGTTTGCGTCGTGGCTCGTCGTGGCGGTGTGATCGACTCGGTCGATGCCAGCCGTATTGTGGTTC
GTGTTGCTGATGACGAAGTTGAAACCGGTGAAGCCGGTGTCGACATCTACAACCTGACCAAGTACACCCGCTCCAACCAGAACAC
CTGCATCAACCAGCGTCCGCTGGTGAGCAAGGGTGATCGGGTTCAGCGCAGCGACATCATGGCCGATGGTCCGTCCACCGACATG
GGTGAGCTGGCACTGGGTCAGAACATGCGTATCGCGTTCATGGCGTGGAACGGCTTCAACTTCGAAGACTCCATCTGCCTGTCCG
AGCGTGTGGTTCAGGAAGACCGTTTCACCACGATCCACATCCAGGAACTGACCTGTGTGGCTCGTGACACCAAGCTTGGCCCAGA
GGAAATCACTGCAGACATCCCGAACGTGGGTGAGGCTGCACTGAACAAGCTGGACGAAGCCGGTATCGTTTATGTAGGTGCTGAA
GTTGGCGCAGGCGACATCCTGGTGGGCAAGGTCACTCCGAAAGGCGAGACCCAGCTGACTCCGGAAGAGAAACTGCTGCGTGCG
ATCTTCGGTGAAAAAGCCAGCGACGTTAAAGACACTTCCCTGCGTGTGCCTACTGGTACCAAGGGCACTGTTATCGACGTACAGG
TCTTCACTCGCGACGGCGTTGAGCGTGATGCTCGTGCACTGTCGATCGAGAAGAGCCAGCTGGACGAGATCCGCAAGGACCTGAA
CGAAGAGTTCCGTATCGTTGAAGGTGCGACCTTCGAACGTCTGCGCTCCGCTCTGGTTGGCCATAAAGCCGAAGGCGGCGCCGGC
CTGAAGAAAGGCCAGGAAATCACCGATGAAGTTCTCGACGGTCTTGAGCATGGCCAGTGGTTCAAACTGCGCATGGCTGAAGATG
CTCTGAACGAGCAGCTCGAGAAGGCCCAGGCCTACATCGTTGATCGTCGCCGTCTGCTGGACGACAAGTTCGAAGACAAGAAGC
GCAAGCTGCAGCAAGGCGATGACCTGGCTCCTGGCGTGCTGAAAATCGTCAAGGTCTACCTGGCAATCCGTCGTCGCATCCAGCC
GGGCGACAAGATGGCCGGTCGTCACGGTAACAAGGGTGTGGTCTCCGTGATCATGCCGGTTGAAGACATGCCGCACGATGCCAAT
GGCACCCCGGTCGACGTAGTCCTCAACCCGCTGGGCGTACCTTCGCGTATGAACGTTGGTCAGATCCTCGAAACTCACCTGGGCCT
CGCGGCCAAAGGTCTGGGCGAGAAGATCAATCGCATGGTCGAAGAGCAGCGCAAGGTCGCAGAACTGCGTACCTTCCTGGACGA
GATCTACAACCAGATCGGCGGTCGCAACGAAGACCTGGACAGCTTCTCCGATCAGGAGATCCTGGATCTGGCGAACAACCTGCGC
GGCGGTGTTCCGATGGCCACTCCGGTGTTCGACGGTGCCAAGGAAAGCGAAATCAAGGCCATGCTGAAACTGGCAGACCTGCCA
GAAAGCGGCCAGATGCAACTGACCGACGGCCGTACCGGCAACAAGTTCGAGCGTCCAGTTACCGTTGGCTACATGTACATGCTGA
AACTGAACCACTTGGTAGACGACAAGATGCACGCTCGTTCTACCGGTTCGTACAGCCTGGTTACCCAGCAGCCGCTGGGTGGTAA
GGCGCAGTTCGGTGGTCAGCGTTTCGGGGAGATGGAGGTCTGGGCACTGGAAGCATACGGTGCTGCATACACTCTGCAAGAAATG

CTCACAGTGAAGTCGGACGATGTGAACGGTCGTACCAAGATGTACAAAAACATCGTGGACGGCGATCACCGTATGGAGCCGGGC
ATGCCCGAGTCCTTCAACGTGTTGATCAAAGAAATTCGTTCCCTCGGCATCGATATCGATCTGGAAACCGAATAA

P. psychrophila KM02_ GCF_011040435.1_ CP049044.1

ATGGCTTACTCATATACTGAGAAAAAACGTATCCGCAAGGACTTTAGCAAGTTGCCGGACGTCATGGATGTGCCGTATCTCTTGGC
AATCCAGCTGGATTCGTATCGTGAATTCTTGCAGGCGGGAGCAACTAAAGATAAGTTCCGCGACGTGGGCCTGCATGCGGCCTTC
AAATCCGTTTTCCCGATCATCAGCTACTCCGGCAATGCTGCGCTGGAGTACGTCGGTTATCGCTTGGGCGAGCCGGCATTTGATGT
CAAAGAATGCGTGTTGCGTGGCGTAACGTACGCCGTACCTTTGCGGGTAAAAGTTCGTTTGATCATTTTCGACAAAGAATCGTCGA
ACAAAGCGATCAAGGACATCAAAGAGCAAGAAGTCTACATGGGTGAAATCCCCCTGATGACTGAAAACGGTACCTTCGTAATCA
ACGGTACCGAGCGTGTAATTGTTTCCCAGCTGCACCGTTCCCCGGGCGTGTTCTTTGACCACGACCGCGGCAAGACGCACAGCTCC
GGTAAGCTGCTTTATTCTGCGCGTATCATTCCTTACCGTGGTTCGTGGTTGGACTTTGAGTTCGATCCGAAAGACTGCGTGTTCGTG
CGTATTGACCGTCGTCGCAAGCTGCCTGCATCGGTATTGCTGCGTGCGCTGGGCTATACCACTGAACAAGTGCTGGACGCGTTCTA
CACCACTAACGTGTTCCACGTTCAGGGCGAAAGCATTAGCCTGGAATTGGTTCCGCACCGTCTGCGCGGTGAAATCGCGGCCATC
GATATTACCGATGACAAAGGCAAGGTGATTGTTGAGCAGGGTCGTCGTATTACTGCTCGCCACATCAACCAGCTGGAAAAAGCCG
GTATCAAAGAGCTCGTTATGCCTCTGGACTATGTCCTGGGTCGCACAACGGCCAAGGCTATCGTGCATCCGGCTACCGGCGAAAT
CATTGCTGAGTGCAACACCGAGCTGACCACTGAAATCCTGGCAAAAATTGCCAAGGGTCAGGTTGTTCGCATCGAGACGTTGTAC
ACCAACGATATCGACTGCGGTCCGTTCGTTTCCGACACTCTGAAGATCGACTCCACCAGCAACCAATTGGAAGCGCTGGTCGAGA
TCTATCGCATGATGCGTCCAGGCGAGCCGCCAACCAAAGACGCTGCTGAGACTCTGTTCAACAACCTGTTCTTCAGCCCCGAGCG
TTATGACCTGTCTGCGGTCGGCCGGATGAAGTTCAACCGTCGTATCGGTCGTACCGAGATCGAAGGTTCGGGCGTGTTGTGCAAA
GAAGACATCGTTGCCGTGCTGAAGACTCTGGTCGACATCCGTAACGGTAAAGGCATCGTCGATGACATCGACCACCTGGGTAACC
GTCGTGTTCGCTGTGTAGGCGAAATGGCCGAGAACCAGTTCCGCGTTGGCCTGGTACGTGTTGAGCGTGCAGTCAAAGAGCGTCT
GTCGATGGCTGAAAGCGAAGGCCTGATGCCGCAAGACTTGATCAACGCCAAGCCAGTGGCTGCGGCGGTGAAAGAGTTCTTCGGT
TCGAGCCAGCTTTCCCAGTTCATGGACCAGAACAACCCGTTGTCCGAGATCACCCACAAGCGCCGTGTTTCTGCACTGGGCCCGG
GCGGTTTGACGCGTGAGCGTGCTGGCTTTGAAGTTCGTGACGTACACCCGACTCACTACGGCCGTGTTTGCCCGATTGAGACGCCG
GAAGGTCCGAACATTGGTCTGATCAACTCCTTGGCTGCTTATGCGCGCACCAACCAGTACGGCTTCCTTGAGAGCCCGTACCGTGT
AGTGAAAGACGCACTGGTAACTGACGAGATCGTTTTCCTGTCCGCCATCGAAGAAGCCGATCACGTGATCGCTCAGGCCTCGGCC
ACGATGAACGACAAGAAAGTCCTGATCGACGAGCTGGTAGCTGTTCGTCACTTGAACGAATTCACCGTTAAGGCGCCGGAAGACG
TCACCTTGATGGACGTCTCTCCGAAGCAGGTTGTTTCGGTTGCAGCGTCGTTGATTCCGTTCCTTGAGCACGATGACGCCAACCGT
GCGTTGATGGGTTCGAACATGCAGCGTCAAGCTGTACCCACTCTGCGCGCTGACAAGCCGCTGGTAGGTACCGGCATGGAGCGTA
ACGTAGCTCGTGACTCCGGCGTTTGCGTCGTGGCTCGTCGTGGCGGCGTGATCGACTCTGTTGATGCCAGCCGTATCGTGGTTCGT
GTTGCTGATGATGAAGTTGAAACTGGCGAAGCCGGTGTCGACATCTACAACCTGACCAAATACACCCGCTCCAACCAGAACACTT
GCATCAACCAGCGTCCGCTGGTTCGCAAGGGTGACCGTGTACAGCGCAGCGACATCATGGCTGACGGCCCGTCCACCGATATGGG
TGAGCTGGCGTTGGGTCAGAACATGCGCATCGCGTTCATGGCCTGGAACGGTTACAACTTCGAAGACTCCATCTGCCTGTCGGAA
CGAGTTGTTCAAGAAGATCGCTTTACCACGATCCACATTCAGGAACTGACCTGTGTGGCACGTGACACCAAGCTTGGGCCTGAAG
AGATCACTGCAGACATCCCGAACGTGGGTGAAGCTGCACTGAACAAACTGGACGAAGCCGGTATTGTTTACGTAGGTGCTGAAGT
TGGCGCTGGCGACATTCTGGTAGGTAAGGTCACTCCGAAAGGCGAGACTCAACTGACCCCAGAAGAGAAGCTGTTGCGCGCTATC
TTCGGTGAAAAAGCCAGCGACGTTAAAGACACTTCCCTGCGTGTACCTACCGGTACCAAAGGTACTGTTATCGACGTACAGGTCT
TCACTCGTGACGGCGTTGAGCGTGATGCTCGTGCCCTGTCGATCGAGAAGACCCAGCTGGACGAGATCCGCAAGGATCTGAACGA
AGAGTTCCGTATCGTTGAAGGCGCCACTTTCGAACGTCTGCGCTCTGCTCTGGTTGGCCGTATAGCCGAGGGCGGTGCCGGTCTGA
AGAAAGGTCAGGAAATCACCAATGAAATCCTGGACGGTCTTGAGCATGGTCAGTGGTTCAAACTGCGCATGGCTGAAGATGCTCT
GAACGAGCAGCTTGAAAAGGCTCAGGCTTACATCATCGATCGCCGTCGTCTGCTGGACGACAAGTTCGAAGACAAGAAGCGCAA
ACTGCAGCAGGGCGATGACCTGGCTCCAGGCGTGCTTAAAATCGTCAAGGTTTACCTGGCAATCCGTCGTCGCATCCAGCCGGGT
GACAAGATGGCTGGTCGTCACGGTAACAAAGGTGTGGTCTCCGTGATCATGCCGGTTGAAGACATGCCGTACGATGCTAATGGCA
CCCCGGTTGATGTGGTTCTCAACCCGTTGGGCGTACCTTCGCGTATGAACGTTGGTCAGATTCTCGAAACTCACCTGGGCCTCGCG
GCTAAAGGTTTGGGCGAGAAGATCAACCTCATGATTGAAGAACAACGCAAGGTCGCTGACCTGCGCAAGTTCCTGCATGAGATCT
ACAACGAAATTGGCGGTCGTCAAGAAAGCCTGGATGACTTCTCGGACCAGGAAATTCTGGATCTGGCGAAGAACCTTCGTGGCGG
TGTGCCGATGGCTACCCCGGTGTTCGACGGTGCCAAAGAAAGCGAAATCAAGGCAATGCTACGTTTGGCTGATCTGCCAGACAGC
GGCCAAATGGAGCTGACTGACGGTCGTACCGGCAACAAGTTCGAGCGTCCGGTAACCGTTGGCTACATGTACATGCTGAAGCTGA
ACCACTTGGTAGACGACAAGATGCATGCTCGTTCTACCGGTTCTTACAGCCTGGTTACCCAGCAGCCGCTGGGTGGTAAGGCACA
GTTCGGTGGTCAGCGTTTCGGGGAGATGGAGGTCTGGGCGCTGGAAGCCTACGGCGCGGCATACACTCTGCAAGAAATGCTCACA
GTGAAGTCGGACGATGTGAACGGTCGTACCAAGATGTACAAAAACATCGTGGACGGCGATCACCGTATGGAGCCGGGCATGCCC
GAGTCCTTCAACGTGTTGATCAAAGAAATTCGTTCCCTCGGCATCGATATCGATCTGGAAACCGAATAA

P. putida NBRC 14164_ GCF_000412675.1_ AP013070.1

ATGGCTTACTCATACACTGAGAAAAAACGTATCCGCAAGGACTTTAGCAAGTTGCCGGACGTCATGGATGTGCCTTACCTCCTGG
CCATCCAGCTGGATTCGTATCGCGAATTCCTGCAAGCGGGAGCATCCAAGGATCAGTTCCGCGACGTCGGCCTGCACGCGGCCTT
CAAATCGGTATTCCCGATCATCAGCTACTCCGGCAATGCTGCCCTGGAGTACGTAGGCTATCGCCTGGGCGAGCCTGCCTTCGATG
TTAAGGAATGTGTCCTGCGTGGCGTGACCTTCGCGGTCCCACTGCGGGTCAAGGTGCGCCTGATCATCTTCGACAAGGAATCGTCG
AACAAAGCGATCAAGGACATCAAAGAGCAAGAAGTCTACATGGGTGAAATCCCCCTGATGACTGAGAACGGTACCTTCGTTATC
AACGGTACCGAGCGTGTGATCGTTTCCCAGCTGCACCGTTCGCCTGGTGTGTTCTTCGACCACGACCGTGGCAAGACTCACAGCTC
CGGCAAGCTGCTGTACTCCGCTCGCATCATCCCTTACCGCGGTTCGTGGCTGGACTTCGAGTTCGACCCGAAAGACTGCGTGTTCG
TGCGTATCGACCGTCGCCGCAAACTGCCGGCGTCGGTGCTGCTGCGCGCCCTGGGTTACAGCACTGAAGAAGTGCTCAACACCTT

CTACACCACCAACGTGTTCCACATTTCCGGCGAAAAACTCAGCCTGGAACTGGTACCGCAGCGTCTGCGTGGTGAAGTTGCAGTC
ATGGACATCCATGACGAAACCGGCAAAGTCATCGTTGAGCAGGGCCGCCGTATTACTGCGCGCCACATCAATCAGCTCGAGAAG
GCCGGCGTCAAGCAGCTGGACGTTCCAATGGAATACGTCCTGGGTCGCACCACTGCCAAGGCCATCGTGCACCCGGCCACTGGCG
AGATCCTGGCCGAGTGCAATACCGAGCTGACCACCGATCTGCTGATCAAAGTCGCCAAGGCACAGGTCGTCCGTATCGAGACCCT
GTACACCAACGACATCGATTGCGGTCCGTTCATCTCCGACACCCTGAAGATCGACACCACCAGCAACCAGCTGGAAGCTCTGGTC
GAGATCTATCGCATGATGCGTCCAGGCGAGCCGCCGACCAAGGATGCTGCCGAGACCCTGTTCAACAACCTGTTCTTCAGCGCCG
AGCGTTACGACCTGTCCGCAGTTGGCCGCATGAAGTTCAACCGTCGTATCGGTCGTACCGAGATCGAAGGTTCGGGCGTGCTGAG
CAAGGAAGACATCGTCGAGGTCCTGAAGACCCTGGTCGATATCCGTAACGGCAAAGGCATCGTCGACGACATCGACCACTTGGGT
AACCGTCGCGTTCGTTGCGTCGGCGAGATGGCCGAAAACCAGTTCCGCGTTGGCCTGGTGCGTGTAGAGCGCGCGGTCAAGGAAC
GTCTGTCGATGGCGGAAAGCGAAGGCTTGATGCCGCAAGACCTGATCAACGCCAAGCCGGTTGCGGCGGCGGTGAAAGAGTTCTT
CGGTTCCAGCCAGCTGTCCCAGTTCATGGACCAGAACAACCCGCTCTCCGAGATCACCCACAAGCGCCGCGTCTCTGCACTCGGC
CCGGGCGGTCTGACCCGTGAGCGTGCTGGCTTCGAAGTCCGTGACGTACACCCGACCCACTACGGCCGTGTGTGCCCGATCGAGA
CCCCTGAAGGTCCGAACATCGGTCTGATCAACTCCCTGGCAGCCTATGCCCGCACCAACCAGTACGGCTTCCTGGAAAGCCCGTA
CCGCGTGGTGAAGGAAGGCGTTGTCAGCGACGACATCGTGTTCCTGTCGGCAATCGAAGAAGCGGACCACGTCATCGCCCAGGCT
TCGGCCGCGATGAACGACAAGAAGCAACTGATCGATGAGCTGGTAGCTGTTCGTCACCTGAACGAATTCACCGTCAAGGCGCCGG
AAGACGTCACCCTGATGGACGTTTCGCCGAAGCAGGTTGTTTCTGTTGCTGCGTCGCTGATTCCGTTCCTCGAGCACGACGACGCC
AACCGTGCGTTGATGGGTTCGAACATGCAGCGTCAGGCTGTACCGACACTGCGTGCCGACAAGCCGCTGGTAGGTACCGGCATGG
AGCGCAACGTTGCCCGTGACTCCGGTGTCTGCGTGGTTGCTCGCCGTGGTGGCGTGATCGACTCGGTCGACGCCAGCCGTATCGTT
GTTCGCGTTAATGACGACGAAGTGGAAACCGGCGAAGCAGGTGTAGATATCTACAACCTGACCAAGTACACCCGTTCGAACCAG
AACACCTGCATCAACCAGCGTCCGCTGGTGAGCAAGGGTGACAAGGTTCAGCGTAGCGACATCATGGCCGACGGCCCGTCCACC
GACATGGGTGAACTGGCGCTGGGTCAGAACATGCGCATCGCGTTCATGGCATGGAACGGCTTCAACTTCGAAGACTCCATCTGTC
TGTCCGAGCGTGTGGTTCAGGAAGATCGCTTCACCACCATCCACATTCAGGAACTGACCTGTGTGGCGCGCGACACCAAGCTTGG
CCCAGAGGAAATCACTGCGGACATCCCGAACGTGGGTGAAGCTGCACTGAACAAACTGGACGAAGCCGGTATCGTTTACGTGGG
TGCTGAAGTCGGCGCTGGCGACATCCTGGTTGGCAAGGTCACGCCAAAAGGCGAAACCCAGCTGACTCCGGAAGAAAAACTGCT
GCGTGCCATCTTCGGTGAGAAGGCCAGTGACGTTAAAGACACCTCCCTGCGCGTGCCAACCGGCACCAAGGGTACCGTCATTGAC
GTGCAGGTCTTCACCCGTGATGGCGTCGAGCGCGACAGCCGCGCCCTGGCCATCGAGAAGATGCAGCTGGACGAGATCCGCAAG
GACCTCAACGAAGAGTTCCGCATCGTCGAAGGCGCAACCTTCGAACGTCTGCGTTCTGCTCTGAACGGCCAGGTGGTCGACGGTG
GCGCGGGCCTGAAGAAAGGCACCGTGGTCACCGACGAAGTGCTGGACGGTCTGGAGCACGGCCAGTGGTTCAAACTGCGCATGG
CTGAAGATGCACTGAATGAGCAGCTGGAAAAGGCTCAGCAGTACATCGTCGACCGTCGCCGTCTGCTGGACGACAAGTTTGAAGA
CAAGAAGCGCAAGCTGCAGCAGGGCGATGACCTGGCACCTGGCGTACTGAAGATCGTCAAGGTCTACCTGGCAATCCGCCGTCG
CATCCAGCCGGGTGACAAGATGGCTGGTCGTCACGGTAACAAGGGTGTTGTCTCGGTAATCATGCCGGTTGAAGACATGCCGCAC
GATGCCAACGGTACTCCGGTCGACGTCGTACTGAACCCGCTGGGCGTACCTTCGCGTATGAACGTTGGTCAGATCCTTGAAACCC
ACCTGGGCCTCGCGGCCAAGGGCCTGGGCGAGAAGATCGACCGCATGATCGAAGAGCAACGCAAAGCCGCTGAGCTGCGCGTAT
TCCTGACCGAGGTCTACAACGAGATCGGTGGTCGTCAGGAAAACCTCGCCGAGTTCACCGACGAAGAGATCCTGGCCCTGGCCCA
CAACCTGAAGAAAGGCGTGCCAATGGCTACCCCGGTCTTCGATGGTGCCAAGGAAAGCGAGATCAAGGCCATGCTGAAGCTGGC
CGATCTGCCAGAGAGCGGCCAGATGGTGCTGTTCGATGGCCGTACCGGCAACAAGTTCGAGCGTCCAGTGACCGTTGGTTACATG
TACATGCTCAAGCTGAACCACTTGGTGGACGACAAGATGCACGCGCGTTCCACTGGTTCCTACAGCCTGGTTACCCAGCAGCCGC
TGGGTGGTAAGGCGCAGTTCGGTGGTCAGCGTTTCGGGGAGATGGAAGTGTGGGCGCTGGAAGCATACGGCGCGGCATACACCC
TGCAAGAAATGCTCACAGTGAAGTCGGACGACGTGAACGGCCGTACCAAGATGTACAAGAACATCGTGGATGGCGATCACCGTA
TGGAGCCGGGCATGCCCGAGTCCTTCAACGTGTTGATCAAAGAGATCCGTTCGCTCGGTATCGATATCGATCTGGAAACCGAATA
A

P. rhizosphaerae DSM 16299_ GCF_000761155.1_ CP009533.1

ATGGCTTACTCATATACTGAGAAAAAACGTATCCGCAAGGACTTTAGCAAGTTGCCGGACGTCATGGACGTACCGTACCTCTTGG
CTATCCAGCTGGATTCGTATCGCGAATTCTTGCAGGCGGGCGCGACCAAGGACCAGTTCCGTGATGTCGGTCTGCATGCGGCCTTC
AAATCCGTTTTCCCGATCATCAGCTACTCCGGCAACGCTGCTCTGGAGTACGTTGGTTATCGTCTGGGCGAACCGGCCTTTGACGT
CAAAGAGTGCGTCCTGCGTGGCGTGACCTACGCCGTGCCGTTGCGTGTGAAAGTGCGCCTGATCATTTTCGACAAAGAATCGTCG
AACAAAGCGATCAAGGACATCAAAGAGCAGGAAGTCTACATGGGGGAAATCCCCCTGATGACCGAGAACGGTACCTTCGTAATC
AACGGTACCGAGCGCGTCATCGTTTCCCAGCTGCACCGTTCGCCAGGCGTGTTCTTCGACCACGACCGTGGCAAGACGCACAGCT
CGGGCAAACTGCTCTATTCGGCACGTATCATCCCTTACCGTGGCTCGTGGCTGGACTTCGAGTTCGATCCGAAAGACTGTGTATTC
GTCCGGATCGACCGTCGTCGTAAATTGCCTGCTTCCGTATTGCTGCGCGCCCTGGGCTACAGCACCGAGGAAGTCCTCGATGCGTT
CTACACCACCAACGTATTCCACGTGCAGGGCGAAATGCTCAGCCTGGAGCTGGTGCCCCAGCGCCTGCGCGGTGAAATCGCCGTT
CTCGACATCGTCGACGAGAAGGGCAAGGTCATTGTCGAACAAGGCCGTCGTATTACTGCCCGGCACATCAACCAGATCGAAAAA
GCTGGCATCAAGCAGCTGGAAGTGCCGCTGGACTACGTCCTAGGTCGCACTACCGCCAAGGCTATCGTACATCCGGCCACCGGCG
AGATCCTGTGCGAGTGCAACACTGAACTGACCACCGAGCTGCTGCTCAAGATTGCCAAGGCGCAGGTCGTTCGCATCGAGACGTT
GTACACCAACGACATCGACTGCGGTCCGTTCATCTCCGATACTCTGAAGATCGATTCCACCGGCAATCAGCTCGAAGCCCTGGTG
GAAATCTATCGCATGATGCGTCCCGGCGAGCCGCCGACCAAGGATGCTGCCGAGACCCTGTTCAACAACCTGTTCTTCAGCCCTG
AGCGCTATGACCTGTCTGCGGTCGGCCGGATGAAGTTCAACCGTCGTATCGGTCGTACCGAGATCGAAGGTTCGGGCGTGTTGAG
CAAGGAAGACATCGTTGCCGTACTGAAGACCCTGGTCGACATCCGTAACGGCAAGGGCATCGTCGACGACATCGACCACCTGGGT
AACCGTCGTGTTCGGTGTGTTGGCGAAATGGCCGAAAACCAGTTCCGTGTTGGCCTGGTGCGTGTAGAGCGCGCGGTCAAGGAAC
GTCTGTCGATGGCCGAAAGCGAAGGCCTGATGCCGCAGGACCTGATCAACGCCAAGCCGGTGGCTGCCGCGGTCAAGGAGTTCTT
CGGTTCCAGCCAGCTCTCGCAGTTCATGGACCAGAACAACCCGCTGTCCGAGATCACCCACAAGCGTCGCGTTTCCGCACTCGGC
CCTGGTGGTCTGACCCGTGAGCGTGCAGGCTTCGAAGTTCGTGACGTTCACCCGACGCACTATGGTCGCGTGTGCCCGATCGAAA
CGCCGGAAGGTCCGAACATCGGCCTGATCAACTCCCTGGCAGCCTATGCGCGCACCAACCAGTACGGCTTCCTGGAAAGCCCGTA

CCGTGTGGTAAAAGACGCTCTGGTCACCGACGAGATCGTGTTCCTGTCCGCCATCGAAGAAGCCGATCACGTGATCGCTCAGGCT
TCGGCCACGATGAACGACAAGAAGGTGCTGATCGACGAGCTGGTAGCCGTACGTCACTTGAACGAATTCACCGTCAAGGCGCCG
GAAGACGTCACCCTGATGGACGTTTCGCCCAAGCAGGTCGTGTCGGTAGCCGCATCGCTGATTCCGTTCCTCGAGCACGACGACG
CCAACCGTGCGTTGATGGGTTCGAACATGCAGCGTCAGGCTGTACCTACCCTGCGTGCCGATAAGCCGCTGGTCGGTACCGGCAT
GGAGCGCAACGTTGCTCGTGACTCCGGCGTTTGCGTCGTGGCTCGTCGTGGTGGCGTGATCGACTCTGTCGATGCCAGCCGTATCG
TGGTTCGGGTTGCCGATGACGAAGTCGAGACTGGCGAAGCCGGTGTCGATATCTACAACCTGACCAAGTACACCCGCTCGAACCA
GAACACCTGCATCAACCAGCGTCCGCTGGTGAACAAGGGTGACGTGGTTCAGCGCAGCGACATCATGGCCGACGGTCCGTCCACC
GACATGGGTGAACTGGCGCTGGGTCAGAACATGCGCATCGCGTTCATGGCGTGGAACGGCTTCAACTTCGAAGACTCCATCTGCC
TGTCCGAGCGTGTGGTTCAGGAAGATCGCTTCACCACGATCCACATCCAGGAACTGACCTGTGTGGCCCGTGACACCAAGCTTGG
CCCAGAGGAAATCACTGCCGACATCCCGAACGTGGGTGAAGCGGCGCTGAACAAGCTGGACGAAGCCGGTATCGTTTACGTGGG
TGCCGAAGTCGGCGCTGGCGACATTCTGGTCGGCAAGGTCACGCCTAAAGGCGAGACCCAGCTCACGCCAGAAGAAAAACTGCT
GCGCGCGATCTTCGGCGAGAAGGCCAGCGACGTTAAGGACACCTCCCTGCGCGTGCCTACCGGCACCAAGGGTACCGTCATCGAC
GTACAGGTCTTCACCCGTGACGGCGTCGAGCGTGATGCTCGTGCACTGTCGATCGAGAAGAGCCAGCTGGACGAGATCCGCAAGG
ACCTGAACGAAGAGTTCCGTATCGTTGAAGGCGCGACCTTCGAACGTCTGCGCTCGGCCCTGATGGGTCAGATCATCGAAGGCGG
CGCTGGCCTGAAGAAAGGCACCGAGGTGACCCACGAGGTCCTCGACGGTCTGGAGCATGGTCAGTGGTTCAAGCTGCGCATGGC
CGAAGACGCGCTGAACGAACAGCTCGAGAAGGCTCAGGCCTACATCGTCGATCGTCGCCGTCTGCTGGACGACAAGTTCGAAGA
CAAGAAGCGCAAGCTGCAGCAAGGCGATGACCTGGCTCCGGGCGTGCTGAAGATCGTTAAGGTCTACCTGGCCATCCGCCGTCGC
ATCCAGCCGGGTGACAAGATGGCCGGTCGTCACGGTAACAAGGGTGTGGTCTCCGTGATCATGCCTGTCGAGGACATGCCGCACG
ACGCCAACGGTACGCCGGTGGACGTGGTACTCAACCCGTTGGGTGTACCTTCGCGTATGAACGTCGGTCAGATTCTCGAAACCCA
CCTGGGCCTCGCGGCCAAGGGTCTGGGCGAGAAGATCAACACCATGCTCGAAGAGCAGCGTAAGGTAGCTGATCTGCGCAAGTT
CCTGACCGAGATCTACAACGAGATCGGTGGCCGTCAAGAACAGCTGGAAACCTTCTCCGACCAGGAAATCCTGGATCTGGCGAA
GAACCTCAAGGGCGGCGTTCCAATGGCCACCCCGGTGTTCGACGGTGCCAAGGAAAGCGAAATCAAGGCCATGCTGAAATTGGC
CGACATGCCGGAAAGCGGCCAGATGCAGCTTTTCGACGGTCGTACCGGCAACAAGTTCGAGCGTCCGGTCACCGTTGGCTACATG
TACATGCTGAAGCTGAACCACTTGGTGGACGACAAGATGCACGCGCGTTCCACTGGTTCTTACAGCCTGGTCACCCAGCAGCCGC
TGGGTGGTAAGGCGCAGTTCGGTGGTCAGCGTTTCGGGGAGATGGAGGTGTGGGCGCTGGAAGCATACGGCGCGGCATACACCC
TGCAAGAAATGCTCACAGTGAAGTCGGACGATGTGAACGGTCGTACCAAGATGTACAAGAACATCGTGGACGGCGATCACCGTA
TGGAGCCGGGCATGCCCGAGTCCTTCAACGTGTTGATCAAAGAGATCCGTTCTCTGGGTATCGATATCGATCTGGAAACCGAATA
A

P. rhodesiae NL2019_ GCF_013285305.1_ CP054205.1

ATGGCTTACTCATATACTGAGAAAAAACGTATCCGCAAGGACTTTAGCAAGTTGCCGGACGTCATGGATGTCCCGTACCTTCTGGC
TATCCAGCTGGATTCGTATCGTGAATTCTTGCAGGCGGGAGCGACCAAAGATCAGTTCCGCGACGTGGGCCTGCATGCGGCCTTC
AAATCCGTTTTCCCGATCATCAGCTACTCCGGCAATGCTGCGCTGGAGTACGTGGGTTATCGCCTGGGCGAACCGGCATTTGATGT
CAAAGAATGTGTGTTGCGTGGCGTTACGTACGCCGTACCTTTGCGGGTAAAAGTCCGTCTGATCATTTTCGACAAAGAATCGTCGA
ACAAAGCGATCAAGGACATCAAAGAGCAAGAAGTCTACATGGGCGAAATCCCATTGATGACTGAAAACGGTACCTTCGTTATCA
ACGGTACCGAGCGTGTTATCGTTTCCCAGCTGCACCGTTCGCCGGGCGTGTTCTTCGACCACGACCGCGGCAAGACGCACAGCTC
CGGTAAGCTCTTGTACTCTGCGCGGATCATTCCGTACCGTGGTTCGTGGTTGGACTTCGAATTCGACCCTAAAGACTGCGTGTTCG
TGCGTATCGACCGTCGTCGTAAATTGCCGGCCTCGGTACTGCTGCGCGCGCTTGGCTACACCACTGAGCAAGTGCTTGATGCTTTC
TACACCACCAACGTATTCAGCCTGAAGGATGAAACCCTCAAGCTGGAACTGATCGCTTCGCGTCTGCGTGGTGAAATTGCCGTTCT
GGATATCCAGGATGAAAAAGGCAAGGTTATTGTTGAAGCTGGCCGTCGTATCACTGCGCGCCATATCAATCAAATCGAAAAAGCC
GGTATTACAGAGCTGGAAGTGCCTCTGGACTACGTCCTGGGTCGCACTACCGCCAAGGTTATCGTTCACCCGGCCACAGGCGAAA
TCCTGGCTGAGTGCAACACCGAGCTGAACACCGAGATCCTGGCCAAAATCGCCAAGGCTCAGGTTGTTCGCATCGAAACCCTGTA
TACCAACGACATCGACTGCGGTCCGTTCATCTCCGACACACTGAAGATCGACTCCACCAGCAACCAATTGGAAGCGCTGGTCGAG
ATCTATCGCATGATGCGTCCTGGTGAACCACCAACCAAAGACGCTGCCGAAACCCTGTTCAACAACCTGTTCTTCAGTCCTGAGCG
TTACGACCTGTCTGCGGTCGGCCGGATGAAGTTCAACCGTCGTATCGGTCGTACCGAGATCGAAGGTTCGGGCGTGCTGTGCAAG
GAAGATATCGTCGCGGTACTGAAGACTCTGGTCGACATCCGTAACGGCAAAGGCATCGTCGATGACATCGACCACCTGGGTAACC
GTCGTGTTCGCTGCGTAGGCGAGATGGCCGAGAACCAGTTCCGCGTTGGCCTGGTACGTGTTGAGCGTGCGGTCAAAGAGCGTCT
GTCGATGGCTGAAAGCGAAGGCCTGATGCCGCAAGACCTGATCAACGCTAAGCCAGTGGCCGCAGCGGTGAAAGAGTTCTTCGG
CTCCAGCCAGCTTTCGCAGTTCATGGACCAGAACAACCCGCTCTCCGAGATTACCCACAAGCGCCGTGTTTCGGCACTGGGCCCG
GGCGGTCTGACTCGTGAGCGTGCTGGCTTCGAAGTTCGTGACGTACACCCTACGCACTACGGTCGTGTTTGCCCGATCGAAACGCC
GGAAGGTCCGAACATCGGTCTGATCAACTCCTTGGCAGCCTATGCGCGCACCAACCAATATGGTTTCCTCGAGAGCCCATACCGT
GTGGTGAAAGACGCTCTGGTCACCGACGAGATCGTGTTCCTGTCCGCAATCGAAGAAGCCGATCACGTGATCGCTCAGGCTTCGG
CCACGATGAACGACAAGAAAGTTTTGATCGACGAGCTGGTAGCTGTTCGTCACTTGAACGAATTCACCGTTAAGGCGCCGGAAGA
CGTCACCTTGATGGACGTTTCGCCGAAGCAGGTAGTTTCGGTTGCAGCGTCTCTGATTCCGTTCCTGGAGCACGATGACGCTAACC
GCGCGTTGATGGGTTCCAACATGCAGCGTCAGGCTGTACCAACCCTGCGCGCTGACAAGCCGCTGGTAGGTACCGGCATGGAGCG
CAACGTAGCGCGTGACTCCGGCGTTTGCGTCGTGGCTCGTCGTGGCGGTGTAATCGATTCCGTTGATGCTAGCCGTATCGTGGTTC
GTGTTGCCGATGACGAAGTTGAAACTGGCGAAGCCGGTGTCGACATCTACAACCTGACCAAATATACCCGTTCGAACCAGAACAC
CTGCATCAACCAGCGTCCGCTGGTGAGCAAGGGTGATCGCGTTCAGCGTAGCGACATCATGGCTGACGGCCCGTCCACCGACATG
GGTGAGCTGGCTCTGGGTCAGAACATGCGCATCGCCTTCATGGCGTGGAACGGCTTCAACTTCGAAGACTCCATCTGCCTGTCCG
AGCGTGTTGTTCAAGAAGATCGCTTCACCACGATCCACATTCAGGAACTGACCTGTGTGGCGCGTGACACAAAGCTTGGGCCAGA
GGAAATCACTGCAGACATTCCGAACGTGGGTGAGGCTGCACTGAACAAGCTGGACGAAGCCGGTATCGTTTATGTAGGTGCTGAA
GTTGGCGCAGGCGACATTCTGGTTGGTAAGGTTACTCCGAAAGGCGAGACCCAACTGACGCCAGAAGAGAAACTGCTGCGTGCC
ATCTTCGGTGAAAAAGCCAGCGACGTTAAAGACACCTCCCTGCGCGTACCTACCGGTACCAAGGGTACTGTCATCGACGTACAGG
TCTTCACCCGTGACGGCGTTGAGCGTGATGCTCGTGCTCTGTCCATCGAGAAGACCCAGCTCGACGAGATCCGTAAGGATCTGAA

CGAAGAGTTCCGTATCGTTGAAGGCGCGACCTTCGAACGTCTGCGCTCCGCTTTGGTAGGCCACAAGGCTGAAGGCGGTGCAGGT
CTGAAGAAAGGTCAAGACATCACTGATGAAGTCCTCGACGGTCTTGAGCACGGCCAGTGGTTCAAACTGCGCATGGCTGAAGATG
CTTTGAACGAGCAGCTCGAGAAGGCCCAGGCCTACATCGTTGATCGCCGCCGTCTGCTGGATGACAAGTTCGAAGATAAGAAGCG
CAAACTGCAGCAGGGCGATGACCTGGCTCCAGGCGTGCTGAAAATCGTCAAGGTTTACCTGGCAATCCGCCGCCGCATCCAGCCG
GGCGACAAGATGGCCGGTCGTCACGGTAACAAAGGTGTGGTCTCCGTGATCATGCCGGTTGAAGACATGCCGCACGATGCCAATG
GCACCCCGGTCGATGTGGTCCTCAACCCGCTGGGCGTACCTTCGCGTATGAACGTTGGTCAGATCCTCGAAACTCACCTGGGCCTC
GCGGCTAAAGGTCTGGGCGAGAAGATCAACCGTATGATCGAAGAGCAGCGCAAGGTTGCTGACCTGCGTAAGTTCTTGCACGAG
ATCTACAACGAGATCGGCGGTCGCAACGAAGAGCTGGACACCTTCTCCGACCAGGAAATCCTGGATCTGGCGAAGAACCTGCGC
GGCGGCGTGCCGATGGCCACACCTGTGTTCGACGGTGCCAAGGAAAGCGAAATCAAGGCCATGCTGAAACTGGCTGACCTGCCG
GAAAGTGGCCAGATGCAGCTGTTCGACGGCCGTACCGGCAACAAATTCGAGCGCCCGGTTACAGTCGGCTACATGTACATGCTGA
AGCTGAACCACTTGGTAGACGACAAGATGCACGCCCGTTCTACCGGATCGTACAGCCTCGTTACCCAGCAGCCGCTGGGTGGTAA
GGCTCAGTTCGGTGGTCAGCGTTTCGGGGAGATGGAGGTCTGGGCACTGGAAGCATACGGTGCTGCTTACACTCTGCAAGAAATG
CTCACAGTGAAGTCGGACGATGTGAACGGTCGGACCAAGATGTACAAAAACATCGTGGACGGCGATCACCGTATGGAGCCGGGC
ATGCCCGAGTCCTTCAACGTGTTGATCAAAGAAATTCGTTCCCTCGGCATCGATATCGATCTGGAAACCGAATAA

P. sediminis B10D7D_ GCF_013409125.2_ CP060009.1

ATGGCTTACTCATACACTGAGAAAAAACGTATCCGCAAGGACTTTAGCAAGTTGCCGGATGTCATGGATGTGCCTTACCTCCTGGC
CATCCAGCTGGATTCGTACCGCGAATTCCTGCAGCAAGGGGTGAGCAAGGAACAGTTCCGCGACATCGGCCTGCATGCGGCCTTC
AAATCGGTATTCCCGATCATCAGCTACTCCGGCAACGCTGCCCTGGAGTACGTCGGCTATCGCCTGGGCGAGCCGGCGTTCGACG
TCAAGGAGTGCGTGCTGCGTGGCGTGACCTTCGCCGTGCCGCTGCGCGTGAAAGTGCGCTTGATCATTTTCGACAAAGAATCGTC
GAACAAAGCGATCAAGGACATCAAAGAGCAAGAAGTGTACATGGGCGAGATTCCGCTCATGACCGAGAACGGTACCTTCGTCAT
CAACGGTACCGAGCGCGTCATCGTGTCCCAGCTGCACCGTTCGCCGGGCGTGTTCTTCGACCACGACCGTGGCAAGACCCACAGC
TCGGGCAAGCTGCTGTACTCCGCTCGCATCATTCCTTACCGCGGTTCGTGGTTGGACTTCGAGTTCGACCCGAAGGACGCGGTATT
CGTACGTATCGACCGTCGCCGCAAACTGCCGGCTTCCGTCCTGCTGCGTGCACTGGGCTACAGCACTGAAGAAGTGCTGGATGCC
TTCTATGACACCAACGTCTACCACGTGAAGGGCGAGAGCCTGAACCTGGAGCTGGTTCCGCAGCGTCTGCGCGGCGAAGTCGCCG
TTCTCGACATCAAGGACGGCAGCGGCAAGGTGATCGTCGAGCAGGGTCGTCGTATCACGGCTCGCCACATCAACCAGCTGGACAA
GGCTGGCATCAAAGAGCTGGAAGTTCCGCTCGACTACGTGATTGGCCGTACCACCGCCAAGGCGATCGTGCACCCGGCTACCGGC
GAAATCATCGCCGAGTGCAACACCGAGCTGACCGCCGACCTGCTGGTCAAGATGGCCAAGGCGCAGGTGGTTCGCTTCGAGACCC
TGTACACCAACGACATCGATTGTGGCCCGTTCATCAGCGACACGCTGAAGATCGACAGCACCACCAATCAGTTGGAAGCGCTGGT
CGAGATCTACCGCATGATGCGTCCTGGCGAGCCGCCAACCAAGGATGCTGCCGAGACCCTGTTCAACAACCTGTTCTTCAGCGCC
GAGCGTTACGACCTGTCCGCCGTGGGCCGCATGAAGTTCAACCGTCGTATCGGTCGCACCGAGATCGAAGGTTCGGGCGTGCTGA
GCAAGGAAGACATCGTTGCCGTACTGAAGACCCTGGTCGATATCCGTAACGGCAAGGGCATCGTCGACGACATCGACCACCTGG
GTAACCGCCGCGTACGTTGCGTCGGCGAGATGGCCGAGAACCAGTTCCGCGTTGGCTTGGTGCGCGTAGAGCGTGCGGTCAAAGA
GCGTCTGTCGATGGCTGAAAGTGAAGGCCTGATGCCGCAAGATCTGATCAACGCCAAGCCGGTTGCGGCGGCGGTGAAAGAGTTC
TTCGGTTCCAGCCAGCTCTCGCAGTTCATGGACCAGAACAACCCGCTCTCCGAGATCACCCACAAGCGCCGTGTTTCTGCACTCGG
CCCAGGTGGTCTGACTCGTGAGCGCGCTGGCTTCGAAGTCCGCGACGTACACCCGACCCACTATGGTCGTGTATGTCCGATCGAA
ACCCCTGAAGGTCCGAACATCGGTCTGATCAACTCGCTGGCTGCCTACGCTCGCACCAACCAGTACGGCTTCCTGGAAAGCCCGT
ACCGCGTGGTCAAGGAAGGTCAGGTCACCGACGAGATCGTGTTCCTGTCCGCCATCGAAGAGGCCGATCATGTGATCGCTCAGGC
ATCCGCTACCCTGAACGACAAGGGGCAGCTGGTGGACGAGCTGGTAGCTGTTCGTCACCTGAACGAGTTCACCGTCAAGGCGCCG
GAAGACGTCACCCTGATGGACGTTTCGCCGAAGCAGGTTGTTTCCGTTGCTGCCTCGCTGATTCCGTTCCTCGAGCACGACGACGC
CAACCGTGCACTCATGGGTTCGAACATGCAGCGTCAGGCTGTACCGACTCTGCGTGCCGACAAGCCGCTGGTAGGTACCGGCATG
GAGCGCAACGTCGCCCGTGACTCCGGCGTCTGCGTCGTGGCCCGTCGCGGTGGCGTGATCGACTCGGTCGATGCCAGCCGTATCG
TGGTTCGTGTCAACGATGACGAAGTTGAAACTGGCGAAGCCGGTGTCGACATCTACAACCTGACCAAGTACACCCGCTCCAACCA
GAATACCTGCATCAACCAGCGTCCGTTGGTGAGCAAAGGTGACAAGGTTGCGCGTAGCGACATCATGGCTGACGGTCCGTCCACC
GACATGGGTGAACTGGCGCTGGGTCAGAACATGCGCGTTGCGTTCATGCCGTGGAACGGCTTCAACTTCGAAGACTCCATCTGCC
TGTCCGAGCGCGTGGTTCAGGAAGATCGCTTCACCACGATCCACATCCAGGAACTGACCTGCGTGGCGCGTGACACCAAGCTCGG
CCCAGAGGAAATCTCCTCTGACATCCCGAACGTTGGCGAAGCGGCGCTGAACAAGCTGGACGAAGCCGGTATCGTCTACGTCGGT
GCCGAAGTTGGCCCGGGCGACATCCTGGTTGGTAAGGTCACCCCGAAAGGTGAGACCCAGCTGACTCCGGAAGAGAAACTGCTG
CGTGCGATCTTCGGTGAGAAGGCGTCCGATGTTAAGGACACCTCCCTGCGTGTGCCGACTGGCACCAAAGGTACCGTCATCGACG
TGCAGGTCTTCACCCGTGACGGCGTGGAGCGCGACTCGCGTGCCCTGTCCATCGAGAAGATGCAGCTGGACGAGATCCGCAAAGA
CCTCAACGAAGAGTTCCGCATCGTCGAAGGCGCAACCTTCGAGCGTCTGCGTTCCGCTCTGGTTGGCGCGACTGCCGAAGGCGGC
GCTGGTCTGAAGAAAGGCACTGCGATCACCGACGAGTTCCTCGACGGTCTCGAGCGTGGCCAGTGGTTCAAGCTGCGCATGGCTG
AAGACGCTCTGAACGAGCAGCTGGAGAAGGCTCAGGCCTACATCTCCGACCGCCGTCAGATGCTCGACGACAAGTTCGAAGACA
AGAAGCGCAAGCTGCAGCAGGGCGATGACCTGGCTCCGGGCGTACTGAAGATCGTCAAGGTCTACCTGGCCATCCGCCGTCGCAT
CCAGCCGGGTGACAAGATGGCCGGTCGTCACGGTAACAAGGGTGTCGTCTCGGTGATCATGCCGGTCGAAGACATGCCGCACGA
CGCCAACGGTACTCCGGTCGATATCGTGCTGAACCCGCTGGGTGTTCCGTCGCGTATGAACGTCGGTCAGATTCTCGAAACCCACC
TGGGCCTCGCGGCCAAAGGCCTGGGCGAGAAGATCAACCGCATGCTCGAAGAGCAGCGCAAGATCGCCGAACTGCGCAAGTTCC
TCGCCGAGATCTACAACGAAATCGGTGGTCGTCAGGAAAACCTCGACGAGTTCTCCGATACCGAGATCCTCGAGCTGGCGAAGAA
CCTCAAAGGCGGTGTACCGATGGCGACTGCCGTGTTCGACGGCGCCAAGGAAACCGAGATCAAGGCCATGCTGAAGCTGGCTGA
TCTGCCGGAGAGCGGCCAGATGCGCCTGTTCGACGGTCGTACCGGTAACCAGTTCGAGCGTCCGACCACCGTCGGCTACATGTAC
ATGCTCAAACTGAACCACCTGGTGGACGACAAGATGCACGCCCGTTCCACTGGTTCCTACAGCCTGGTTACCCAGCAGCCGCTGG
GTGGTAAGGCGCAGTTCGGTGGTCAGCGTTTCGGGGAGATGGAGGTCTGGGCGCTGGAAGCCTATGGCGCCGCCTACACCCTGCA
GGAAATGCTGACCGTGAAGTCGGACGACGTGAACGGCCGTACCAAGATGTATAAGAACATCGTGGATGGCGATCACCGTATGGA
GCCGGGCATGCCCGAGTCCTTCAACGTACTGATCAAAGAGATCCGTTCGCTCGGCATCGATATCGATCTGGAAACCGAATAA

P. silesiensis A3_ GCF_001661075.1_ CP014870.1

ATGGCTTACTCATATACTGAGAAAAAACGTATCCGCAAGGACTTTAGCAAGTTGCCGGACGTCATGGATGTGCCGTATCTCCTGG
CAATCCAGCTGGATTCGTATCGTGAATTCTTGCAGGCGGGAGCGACTAAAGATCAGTTCCGCGACGTGGGCCTGCATGCGGCCTT
CAAATCCGTTTTCCCGATCATCAGCTACTCCGGCAATGCTGCGCTGGAGTACGTCGGTTATCGCCTGGGCGAACCGGCATTTGATG
TCAAAGAATGCGTATTGCGCGGTGTAACTTACGCCGTACCTTTGCGGGTAAAAGTGCGCCTGATCATTTTCGACAAAGAATCGTCG
AACAAAGCGATCAAGGACATCAAAGAGCAAGAAGTCTACATGGGTGAAATCCCCCTGATGACTGAAAACGGTACCTTCGTAATC
AACGGTACCGAGCGAGTAATCGTTTCCCAGCTGCACCGTTCCCCGGGCGTGTTCTTCGACCACGACCGCGGCAAGACGCATAGCT
CCGGCAAACTGCTGTACTCCGCGCGCATCATTCCTTACCGCGGTTCGTGGCTGGACTTCGAGTTCGACCCGAAAGACTGCGTATTC
GTGCGTATCGACCGTCGTCGCAAGCTGCCTGCATCGGTACTGCTGCGCGCGCTCGGCTATACCACTGAAGAAGTGCTCGACGCGT
TCTACACCACCAACGTTTTCCACCTGAGCGGCGAAACCCTCAGTCTGGAACTGATTGCTTCGCGTCTGCGTGGTGAAATCGCTGTT
CTTGATATTCAGGACGAGAAGGGCAAGGTCATCGTTGAGGCTGGTCGCCGTATTACTGCGCGCCACATCAACCAGATCGAAAAAG
CCGGTCTCAAGACCCTGGAAGTGCCTCTGGACTACGTCCTGGGTCGCACTACCGCCAAGGCCATCGTGCATCCGGCAACCGGCGA
AATCCTGGCAGAGTGCAACACCGAGCTGAACACCGAGATCCTGGCAAAAATCGCCAAGGCCCAGGTTGTTCGCATCGAGACTCTG
TACACCAACGATATCGACTGCGGTCCGTTCGTCTCCGACACCCTGAAGATCGACTCCACCAGCAACCAATTGGAAGCGCTGGTCG
AGATCTATCGCATGATGCGTCCAGGCGAGCCGCCAACCAAAGACGCTGCCGAGACCCTGTTCAACAACCTGTTCTTCAGCCCTGA
GCGCTATGACCTGTCTGCGGTCGGCCGGATGAAGTTCAACCGTCGTATCGGTCGTACCGAGATCGAAGGTTCGGGCGTGTTGTGC
AAAGAAGACATCGTCGCGGTACTGAAGACTCTGGTCGACATCCGTAACGGTAAAGGCATCGTCGATGACATCGACCACCTGGGTA
ACCGTCGTGTTCGCTGCGTAGGCGAAATGGCCGAGAACCAGTTCCGCGTTGGCCTGGTACGTGTTGAGCGTGCGGTCAAAGAGCG
TCTGTCGATGGCTGAAAGCGAAGGCCTGATGCCGCAAGACCTGATCAACGCCAAGCCAGTGGCTGCGGCGGTGAAAGAGTTCTTC
GGTTCCAGCCAGCTTTCCCAGTTCATGGACCAGAACAACCCGCTGTCCGAGATCACCCACAAGCGTCGTGTGTCTGCACTCGGCCC
TGGCGGTTTGACCCGTGAGCGTGCCGGCTTTGAAGTGCGTGACGTACACCCGACTCACTACGGTCGTGTATGCCCGATCGAAACG
CCGGAAGGTCCGAACATCGGCCTGATCAACTCCCTGGCTGCCTATGCGCGCACCAACCAGTACGGCTTCCTCGAGAGCCCGTACC
GTGTGGTGAAAGACGCTCTGGTCACCGACGAGATCGTGTTCCTGTCCGCCATCGAAGAAGCTGATCACGTGATCGCTCAGGCTTC
GGCCACGATGAACGACAAGAAGGTCCTGATCGACGAACTGGTAGCTGTTCGTCACTTGAACGAGTTCACCGTCAAGGCGCCGGA
AGACGTCACCTTGATGGACGTATCGCCGAAGCAGGTAGTTTCGGTTGCAGCGTCGCTGATCCCGTTCCTCGAGCACGATGACGCC
AACCGTGCGTTGATGGGTTCGAACATGCAGCGTCAAGCTGTACCTACCCTGCGCGCTGACAAGCCGCTGGTCGGTACCGGCATGG
AGCGTAACGTAGCCCGTGACTCCGGCGTTTGCGTCGTGGCTCGTCGTGGCGGCGTGATCGACTCCGTCGACGCCAGCCGTATCGT
GGTTCGTGTTGCCGATGACGAAGTTGAAACCGGTGAAGCCGGTGTCGACATCTACAACCTGACCAAATACACCCGCTCCAACCAG
AACACCTGCATCAACCAGCGTCCGCTGGTGCGTAAAGGTGATCGGGTTCAGCGCAGCGACATCATGGCCGACGGCCCGTCCACCG
ACATGGGTGAACTGGCGCTGGGTCAGAACATGCGCATCGCGTTCATGGCATGGAACGGCTTCAACTTCGAAGACTCCATCTGCCT
GTCCGAGCGTGTGGTTCAGGAAGACCGCTTCACCACGATCCACATTCAGGAACTGACCTGTGTGGCGCGTGACACCAAGCTTGGG
CCAGAGGAAATCACTGCCGACATCCCGAACGTGGGTGAAGCGGCACTGAACAAGCTGGACGAAGCCGGTATCGTTTACGTAGGT
GCCGAAGTAGGCGCAGGCGACATCCTGGTCGGCAAGGTCACTCCGAAAGGCGAGACCCAACTGACTCCGGAAGAAAAACTGCTG
CGTGCCATCTTCGGTGAAAAAGCCAGCGACGTTAAAGACACCTCCCTGCGCGTGCCTACCGGCACCAAGGGTACTGTCATCGACG
TACAGGTCTTCACCCGCGACGGCGTTGAGCGTGATGCTCGTGCACTGTCGATCGAGAAGACTCAACTCGACGAGATCCGCAAGGA
TCTGAACGAAGAGTTCCGTATCGTTGAAGGCGCGACCTTCGAACGTCTGCGTTCCGCTCTGGTCGGCCACAAAGCCGAAGGCGGC
GCCGGCCTGAAGAAAGGTCAGGACATCACCGACGAAGTGCTCGACGGTCTTGAGCATGGCCAGTGGTTCAAACTGCGCATGGCT
GAAGATGCTCTGAACGAGCAGCTCGAGAAGGCTCAGGCCTACATCGTTGATCGCCGCCGTCTGCTGGACGACAAGTTCGAAGACA
AGAAGCGCAAACTGCAGCAGGGCGATGACCTGGCTCCAGGCGTGCTGAAAATCGTCAAGGTTTACCTGGCAATCCGTCGTCGCAT
CCAGCCGGGCGACAAGATGGCCGGTCGTCACGGTAACAAAGGTGTGGTCTCCGTGATCATGCCGGTTGAAGACATGCCGCACGAT
GCCAATGGCACCCCGGTCGACGTCGTCCTCAACCCGTTGGGCGTACCTTCGCGTATGAACGTTGGTCAGATCCTTGAAACCCACTT
GGGCCTCGCGGCCAAAGGCCTGGGCGAGAAGATCAACCGCATGATCGAGGAGCAACGTAAAGTTGCCGATCTGCGCAAGTTCCT
GCACGAGATCTACAACGAGATCGGCGGTCGCAACGAAGATCTGGACAGCTTCTCCGACCAGGAAATCCTGGATCTGGCGAAGAA
CCTGCGTGGCGGCGTTCCAATGGCCACTCCAGTATTCGACGGTGCCAAGGAAAGCGAAATCAAGGCCATGCTGAAACTGGCGGA
CCTGCCAGAAAGCGGCCAGATGCAGCTGACCGACGGCCGTACCGGCAACAAGTTCGAGCGCCCGGTGACTGTTGGCTACATGTAC
ATGCTGAAGCTGAACCACTTGGTAGACGACAAGATGCACGCTCGTTCTACCGGTTCGTACAGCCTGGTTACCCAGCAGCCGCTGG
GTGGTAAGGCGCAGTTCGGTGGTCAGCGTTTCGGGGAGATGGAGGTCTGGGCACTGGAAGCATACGGTGCTGCTTACACTCTGCA
AGAAATGCTCACAGTGAAGTCGGACGATGTGAACGGCCGTACCAAGATGTACAAAAACATCGTGGATGGCGATCACCGTATGGA
GCCGGGCATGCCCGAGTCCTTCAACGTGTTGATCAAGGAAATTCGTTCCCTCGGCATCGATATCGATCTGGAAACCGAATAA

P. simiae PCL1751_ P. simiae PCL1751_ CP010896.1

ATGGCTTACTCATATACTGAGAAAAAACGTATCCGCAAGGACTTTAGCAAGTTGCCGGACGTCATGGATGTCCCGTACCTTCTGGC
TATCCAGCTGGATTCGTATCGTGAATTCTTGCAGGCGGGAGCGACCAAAGATCAGTTCCGCGACGTGGGCCTGCATGCGGCCTTC
AAATCCGTTTTCCCGATCATCAGCTACTCCGGCAATGCTGCGCTGGAGTACGTCGGTTATCGCCTGGGCGAACCGGCATTTGATGT
CAAAGAATGCGTGTTGCGCGGTGTTACGTACGCCGTACCTTTGCGGGTAAAAGTCCGTCTGATCATTTTCGACAAAGAATCGTCGA
ACAAAGCGATCAAGGACATCAAAGAGCAAGAAGTCTACATGGGCGAAATCCCACTGATGACTGAGAACGGTACCTTCGTTATCA
ACGGTACCGAACGTGTGATCGTTTCCCAGCTGCACCGTTCCCCGGGCGTGTTCTTCGACCACGACCGCGGCAAGACGCACAGCTC
TGGTAAGCTCCTGTACTCCGCGCGGATCATTCCTTACCGTGGTTCGTGGTTGGACTTCGAGTTCGATCCGAAAGACTGCGTGTTCG
TGCGTATCGACCGTCGTCGCAAGCTGCCGGCCTCGGTACTGCTGCGCGCGCTCGGCTACACCACTGAGCAAGTGCTGGACGCCTT
CTACACCACCAACGTATTCAGCCTGAAGGATGAAACCCTCAAGCTGGAGCTGATTGCTTCGCGTCTGCGTGGTGAAATTGCTGTCC
TGGACATTCAGGATGAAAAGGGCAAGGTCATTGTTGAAGCTGGCCGTCGTATCACTGCGCGCCACATCAACCAGATCGAAAAAGC
CGGTATCAAAGAGCTGGAAGTGCCTCTGGACTACGTCCTTGGCCGCACGACCGCCAAGGTCATCGTTCACCCAGCTACAGGCGAA

ATCCTGGCTGAGTGCAACACCGAGCTGAACACCGAAATCCTGGCCAAAATCGCCAAGGCTCAGGTTGTTCGCATCGAGACCCTGT
ATACCAACGACATCGACTGCGGTCCGTTCATTTCCGACACGCTGAAGATCGACTCCACCAGCAACCAATTGGAAGCGCTGGTCGA
GATCTATCGCATGATGCGTCCTGGTGAGCCACCAACCAAAGACGCTGCCGAAACCCTGTTCAACAACCTGTTCTTCAGCCCTGAG
CGCTATGACCTGTCTGCGGTCGGCCGGATGAAGTTCAACCGTCGTATCGGTCGTACCGAGATCGAAGGTTCGGGCGTGCTGTGCA
AGGAAGACATCGTCGCGGTACTGAAGACCCTGGTCGACATCCGTAACGGCAAAGGCATCGTCGATGACATCGACCACCTGGGTA
ACCGTCGTGTTCGCTGTGTAGGCGAAATGGCCGAGAACCAGTTCCGCGTTGGCCTGGTACGTGTTGAGCGTGCGGTCAAAGAGCG
TCTGTCGATGGCTGAAAGCGAAGGCCTGATGCCGCAAGACCTGATCAACGCCAAGCCAGTGGCTGCGGCGGTGAAAGAGTTCTTC
GGTTCCAGCCAGCTTTCCCAGTTCATGGACCAGAACAACCCGCTCTCCGAGATCACCCACAAGCGCCGTGTATCTGCACTGGGCC
CGGGCGGTCTGACCCGTGAGCGTGCTGGCTTTGAAGTTCGTGACGTACACCCGACGCACTACGGTCGTGTTTGCCCGATCGAAAC
GCCGGAAGGTCCGAACATCGGTCTGATCAACTCCCTGGCCGCTTATGCGCGCACCAACCAGTACGGCTTCCTCGAGAGCCCGTAC
CGCGTGGTGAAAGACGCTCTGGTCACCGACGAGATCGTGTTCCTGTCCGCCATCGAAGAAGCTGATCACGTGATCGCTCAGGCTT
CGGCCACGATGAACGACAAGAAAGTCCTGGTCGACGAGCTGGTAGCTGTTCGTCACTTGAACGAGTTCACCGTCAAGGCGCCGGA
AGACGTCACCTTGATGGACGTGTCGCCGAAGCAGGTAGTGTCGGTTGCAGCGTCGCTGATCCCGTTCCTGGAGCACGATGACGCC
AACCGTGCGTTGATGGGTTCCAACATGCAGCGTCAAGCTGTACCGACCCTGCGCGCCGACAAGCCGCTGGTAGGTACCGGCATGG
AGCGTAACGTAGCTCGTGACTCCGGCGTTTGCGTCGTGGCTCGTCGTGGCGGCGTGATCGATTCCGTTGATGCCAGCCGTATCGTG
GTTCGTGTTGCCGATGACGAAGTAGAAACTGGCGAAGCCGGTGTCGACATCTACAACCTGACCAAATACACCCGCTCGAACCAGA
ACACCTGCATCAACCAGCGTCCGCTGGTGAGCAAGGGTGATCGCGTTCAGCGCAGCGACATCATGGCTGATGGCCCGTCCACCGA
TATGGGTGAGCTGGCTCTGGGCCAGAACATGCGTATCGCGTTCATGGCATGGAACGGCTTCAACTTCGAAGACTCCATCTGCCTGT
CCGAGCGTGTTGTTCAAGAAGACCGCTTCACCACGATCCACATTCAGGAACTGACCTGTGTGGCACGTGACACCAAGCTTGGGCC
TGAGGAAATCACTGCAGACATCCCGAACGTGGGTGAAGCTGCACTGAACAAGCTGGACGAAGCCGGTATCGTTTACGTAGGTGCT
GAAGTAGGCGCAGGCGACATCCTGGTAGGTAAGGTCACCCCGAAAGGCGAGACTCAACTGACTCCGGAAGAAAAACTGCTGCGT
GCCATCTTCGGTGAAAAAGCCAGCGACGTTAAAGACACCTCCCTGCGTGTGCCTACCGGTACCAAGGGTACTGTCATCGACGTAC
AGGTCTTCACCCGTGACGGCGTTGAGCGTGATGCTCGTGCACTGTCCATCGAGAAGACCCAGCTCGACGAGATCCGCAAGGACCT
GAACGAAGAGTTCCGTATCGTTGAAGGCGCGACCTTCGAACGTCTGCGTTCCGCCCTGGTAGGCCACAAGGCTGAAGGCGGCGCA
GGTCTGAAGAAAGGTCAGGACATCACCGACGAAATCCTCGACGGTCTTGAGCACGGCCAGTGGTTCAAACTGCGCATGGCTGAA
GATGCTCTGAACGAGCAGCTCGAGAAGGCTCAGGCCTACATCGTTGATCGTCGCCGTCTGCTGGACGACAAGTTTGAAGACAAGA
AGCGCAAACTGCAGCAGGGCGATGACCTGGCTCCAGGCGTGCTGAAAATCGTCAAGGTTTACCTGGCAATCCGTCGCCGCATCCA
GCCGGGCGACAAGATGGCTGGTCGTCACGGTAACAAGGGTGTGGTCTCCGTGATCATGCCGGTTGAAGACATGCCGCACGATGCC
AATGGCACCCCGGTCGACGTCGTCCTCAACCCGTTGGGCGTACCTTCGCGTATGAACGTTGGTCAGATCCTTGAAACCCACCTGGG
CCTCGCGGCCAAAGGTCTGGGCGAGAAGATCAACCGTATGATCGAAGAGCAGCGCAAGGTCGCAGACCTGCGTAAGTTCCTGCA
CGAGATCTACAACGAGATCGGCGGTCGCAACGAAGAGCTGGACACCTTCTCCGACCAGGAAATCCTGGATCTGGCGAAGAACCT
GCGCGGCGGCGTTCCAATGGCTACCCCGGTGTTCGACGGTGCCAAGGAAAGCGAAATCAAGGCCATGCTGAAACTGGCAGACCT
GCCAGAAAGCGGCCAGATGCAGCTGTTCGACGGCCGTACCGGCAACAAGTTCGAGCGCCCGGTTACTGTTGGCTACATGTACATG
CTGAAGCTGAACCACTTGGTAGACGACAAGATGCACGCTCGTTCTACCGGTTCGTACAGCCTCGTTACCCAGCAGCCGCTGGGTG
GTAAGGCTCAGTTCGGTGGTCAGCGTTTCGGGGAGATGGAGGTCTGGGCACTGGAAGCATACGGTGCTGCTTACACTCTGCAAGA
AATGCTCACAGTGAAGTCGGACGATGTGAACGGCCGGACCAAGATGTACAAAAACATCGTGGACGGCGATCACCGTATGGAGCC
GGGCATGCCCGAGTCCTTCAACGTGTTGATCAAAGAAATTCGTTCCCTCGGCATCGATATCGATCTGGAAACCGAATAA

P. soli SJ10_ GCF_000498975.2_ CP009365.1

ATGGCTTACTCATACACTGAGAAAAAACGTATCCGCAAGGACTTTAGCAAGTTGCCGGACGTCATGGATGTGCCGTACCTCCTGG
CCATCCAGCTGGATTCGTATCGCGAATTCCTGCAGGCGGGAGCATCCAAGGATCAGTTCCGCGACGTCGGTCTGCATGCGGCCTT
CAAATCGGTATTCCCGATCATCAGCTACTCCGGCAATGCTGCCCTGGAGTACGTCGGCTATCGCCTGGGCGAGCCGGCTTTCGATG
TGAAGGAATGTGTCCTGCGCGGTGTGACCTTCGCGGTCCCGCTGCGGGTGAAGGTGCGCCTGATCATCTTCGACAAGGAATCGTC
GAATAAAGCGATCAAGGACATCAAAGAGCAAGAAGTCTACATGGGTGAAATCCCCCTGATGACTGAAAACGGTACCTTCGTTATC
AACGGTACCGAGCGTGTGATCGTATCCCAGCTGCACCGTTCGCCTGGTGTGTTCTTCGACCACGACCGTGGCAAGACGCACAGCT
CCGGTAAGCTGCTGTACTCCGCGCGCATCATCCCTTACCGCGGTTCGTGGCTGGACTTCGAGTTCGACCCGAAAGACTGCGTGTTC
GTACGTATCGACCGTCGCCGCAAACTGCCGGCCTCGGTACTGCTGCGTGCCCTGGGTTACAGCACCGAAGAGGTTCTGAACACCT
TCTACACCACCAACGTGTTCCACCTTTCCGGCGAGAAGCTCAGCCTGGAGCTGGTGCCTCAGCGTCTGCGGGGTGAAGTTGCGGT
CATGGATATCCATGACGACAGCGGCAAGGTCATCGTCGAGCAAGGCCGCCGTATTACCGCGCGCCACATCAACCAGCTGGAAAA
AGCTGGCGTGAAAGAGCTGGACGTTCCGCTGGAGTACGTACTGGGTCGCACCACCGCCAAGGCGATCGTGCATCCGGCTACCGGC
GAGATCATTGCCGAGTGCAACACCGAGCTGACCACCGACCTGCTGGTGAAGATCGCCAAGGCCCAGGTCGTTCGCGTCGAGACGC
TGTACACCAACGACATCGACTGCGGTCCGTTCATCTCCGACACCCTGAAGATCGACACCACCAGCAACCAACTGGAAGCGCTGGT
CGAGATCTACCGCATGATGCGTCCAGGCGAGCCACCAACCAAGGACGCCGCCGAGACCCTGTTCAACAACCTGTTCTTCAGCGCC
GAGCGTTACGACCTGTCCGCCGTTGGCCGCATGAAGTTCAACCGTCGTATCGGTCGCACCGAGATCGAAGGTTCGGGCGTGCTGA
GCAAGGAAGACATCGTCGAGGTCCTCAAGACCCTGGTCGATATCCGTAACGGCAAAGGCATCGTCGACGACATCGACCACCTGG
GTAACCGTCGCGTCCGTTGCGTCGGCGAGATGGCCGAGAACCAGTTCCGCGTTGGCCTGGTGCGTGTTGAGCGCGCGGTCAAAGA
GCGTCTGTCGATGGCTGAAAGCGAAGGCCTGATGCCGCAGGACCTGATCAATGCCAAGCCGGTTGCGGCGGCGGTGAAAGAGTT
CTTCGGTTCCAGCCAGCTCTCGCAGTTCATGGACCAGAACAACCCGCTTTCCGAGATCACCCACAAGCGCCGCGTTTCCGCACTCG
GCCCTGGTGGTCTGACCCGTGAGCGTGCTGGCTTCGAAGTCCGTGACGTACACCCGACTCACTACGGCCGCGTGTGCCCGATCGA
GACCCCTGAAGGTCCGAACATCGGCCTGATCAACTCCCTGGCGGCCTATGCCCGCACCAACCAGTACGGCTTCCTGGAAAGCCCG
TACCGCGTGGTGAAGGAAGGCGTGGTCAGCGACGACATCGTGTTCCTGTCCGCCATCGAAGAAGCCGATCACGTGATCGCCCAGG
CTTCGGCCGCGATGAACGACAAGAAGCAGCTGATCGATGAGCTGGTAGCTGTTCGTCACCTGAACGAATTCACCGTCAAGGCGCC
GGAAGACGTCACCCTGATGGACGTTTCGCCGAAGCAGGTAGTTTCGGTTGCCGCGTCGCTGATTCCGTTCCTCGAGCACGACGAC
GCCAACCGTGCGTTGATGGGTTCCAACATGCAGCGTCAGGCTGTACCGACCCTGCGCGCCGACAAGCCGCTGGTAGGTACCGGCA

TGGAGCGCAACGTTGCCCGTGACTCCGGTGTCTGCGTGGTTGCTCGTCGCGGTGGTGTGATCGACTCGGTCGACGCCAGCCGTATC
GTCGTGCGCGTTGCCGACGACGAAGTCGAGACCGGTGAAGCAGGTGTGGATATCTACAACCTGACCAAATACACCCGCTCCAACC
AGAACACCTGCATCAACCAGCGTCCGCTGGTGAGCAAGGGTGACAAGGTTGCGCGTGGCGACATCATGGCTGACGGCCCGTCCA
CCGACATGGGTGAACTGGCACTGGGTCAGAACATGCGCATCGCGTTCATGGCGTGGAACGGCTTCAACTTCGAAGACTCCATCTG
CCTGTCCGAGCGTGTGGTTCAGGAAGACCGCTTCACCACGATCCACATCCAGGAACTGACCTGTGTGGCCCGTGACACCAAGCTT
GGCCCAGAGGAAATCACCGCGGACATCCCGAACGTCGGTGAAGCTGCGCTGAACAAGCTGGACGAAGCCGGTATCGTCTACGTG
GGTGCCGAAGTCGGCGCTGGCGACATCCTGGTCGGCAAGGTCACTCCGAAAGGCGAGACCCAGCTGACTCCGGAAGAAAAACTG
CTGCGTGCGATCTTCGGTGAGAAGGCCAGCGACGTCAAAGACACCTCCCTGCGTGTGCCGACCGGCACCAAGGGTACCGTCATCG
ACGTACAGGTCTTCACCCGTGATGGCGTCGAGCGCGACAGTCGCGCCCTGGCCATCGAGAAGATGCAGCTGGACGAGATCCGCA
AGGACCTCAACGAAGAGTTCCGCATCGTCGAAGGCGCGACCTTCGAGCGTCTGCGTTCGGCTCTGAACGGCCAGGTGGTCGACGG
TGGCGCGGGCCTGAAGAAAGGCACCGTGATCAGCGACGACGTGCTGAACGGTCTGGAGCACGGCCAGTGGTTCAAACTGCGCAT
GGCCGAAGATGCACTGAACGAGCAGCTGGAAAAGGCTCAGCAGTACATCGTCGATCGCCGTCGCCTGCTGGACGACAAGTTCGA
AGACAAGAAGCGCAAGCTGCAGCAGGGCGATGACCTGGCTCCGGGCGTACTGAAGATCGTCAAGGTCTACCTGGCAATTCGCCG
TCGCATCCAGCCGGGTGACAAGATGGCCGGTCGTCACGGTAACAAGGGTGTCGTCTCGGTAATCATGCCGGTCGAAGACATGCCG
CACGACGCCAACGGTACTCCGGTCGACGTCGTACTGAACCCGCTGGGCGTACCTTCGCGTATGAACGTCGGTCAGATCCTTGAAA
CCCACCTGGGCCTCGCGGCCAAGGGTCTGGGCGAGAAGATCGACCGCATGATCGAAGAGCAGCGCAAAGCCGCTGAACTGCGCA
CCTTCCTCACCGAGATCTACAACGAGATCGGTGGTCGTCAGGAGAACCTGGAAGAGTTCACCGACGAAGAGATCATCGCCCTGGC
GAACAACCTGAAGAAAGGCGTGCCAATGGCCACTCCAGTCTTCGACGGTGCCAAGGAGCGTGAGATCAAGGCCATGCTGAAACT
GGCTGATCTGCCAGAGAGCGGCCAGATGGTGCTGTTCGATGGCCGTACCGGCAACAAGTTCGAGCGTCCGGTGACCGTTGGTTAC
ATGTACATGCTCAAGCTGAACCACTTGGTGGACGACAAGATGCACGCGCGTTCCACTGGTTCGTACAGCCTGGTTACCCAGCAGC
CGCTGGGTGGTAAGGCGCAGTTCGGTGGTCAGCGTTTCGGGGAGATGGAAGTGTGGGCGCTGGAGGCATATGGCGCGGCATACA
CCCTGCAAGAAATGCTCACAGTGAAGTCGGACGACGTGAACGGCCGTACCAAGATGTACAAGAACATCGTGGATGGCGATCACC
GTATGGAGCCGGGCATGCCCGAGTCCTTCAACGTGTTGATCAAAGAGATCCGTTCCCTCGGTATCGATATCGATCTGGAAACCGA
ATAA

P. syringae BIM B-268_ GCF_016694755.2_ CP068034.2

ATGGCTTACTCATATACTGAGAAAAAACGTATCCGCAAGGACTTTAGCAAGTTGCCGGACGTAATGGATGTGCCGTATCTCTTGG
CCATCCAGCTGGATTCGTATCGCGAATTCCTGCAGGCGGGAGCGACCAAAGATCAGTTCCGCGACGTCGGTCTGCATGCAGCCTT
CAAATCCGTTTTCCCGATCATCAGCTACTCCGGCAATGCTGCGCTGGAGTATGTAGGTTATCGCTTGGGCGAACCGGCATTTGATG
TCAAGGAATGCGTGCTGCGCGGTGTGACTTACGCAGTACCTCTGCGAGTCAAGGTCCGTCTGATCATTTTCGACAAAGAATCGTCG
AACAAAGCGATCAAGGACATCAAAGAGCAAGAAGTCTACATGGGTGAAATCCCCCTGATGACTGAGAACGGTACCTTTGTAATC
AATGGCACCGAGCGCGTTATCGTGTCTCAGCTTCACCGTTCGCCTGGCGTATTCTTCGACCACGACCGTGGCAAGACGCACAGCTC
CGGCAAGCTGCTGTACTCCGCTCGTATCATTCCTTACCGCGGTTCGTGGCTGGACTTCGAGTTCGATCCGAAAGACTGCGTATTCG
TCCGTATCGACCGTCGTCGCAAGCTGCCTGCGTCCGTGCTTCTGCGCGCTCTGGGTTACACCACCGAGCAAGTGCTTGATGCTTTC
TATACCACCAACGTATTCCATGTGCGCGGTGAAAACCTGAGCCTGGAACTGGTGCCTCAGCGCCTGCGTGGTGAAATTGCCGTTCT
GGATATCCTGGACGACAAGGGCAAGGTCATTGTCGAGCAGGGTCGTCGTATCACTGCCCGTCACATCAACCAGCTGGAAAAGGCC
GGGATCAAAGAGCTGGAAGTGCCTCTGGACTACGTCCTGGGTCGTACTACAGCCAAGGTCATCGTGCATCCGGCAACCGGTGAGA
TCATTGCCGAGTGCAACACCGAGCTGAACACCGAAATCCTGGCCAAGATCGCCAAGGCTCAGGTTGTTCGCATCGAAACGTTGTA
CACCAACGACATCGATTGCGGTCCGTTCGTCTCCGACACACTGAAGATTGACTCCACCAGCAACCAACTGGAAGCGCTGGTCGAG
ATCTATCGCATGATGCGTCCTGGCGAGCCGCCAACCAAGGATGCCGCTGAAACACTGTTCAACAACCTGTTCTTCAGCCCTGAGC
GCTACGACCTGTCTGCTGTAGGCCGTATGAAGTTCAACCGTCGTATCGGTCGTACCGAAATCGAAGGCTCGGGCGTGCTGTGCAA
GGAAGACATCGTAGCGGTCCTCAAGACCCTCGTTGATATCCGTAACGGCAAAGGCATCGTCGATGACATCGACCACCTCGGTAAC
CGTCGCGTTCGTTGCGTTGGCGAGATGGCCGAGAACCAGTTCCGTGTTGGTCTGGTCCGTGTCGAGCGCGCTGTTAAAGAACGTCT
GTCGATGGCAGAAAGCGAAGGCCTGATGCCTCAGGACCTGATCAACGCCAAACCTGTTGCGGCGGCGGTCAAAGAGTTCTTCGGT
TCCAGCCAGCTTTCCCAGTTCATGGACCAGAACAACCCGCTGTCCGAGATCACGCACAAGCGTCGTGTTTCCGCACTCGGCCCTGG
CGGTCTGACGCGTGAGCGCGCTGGCTTTGAAGTTCGAGACGTTCACCCGACTCACTACGGCCGTGTGTGCCCGATCGAGACGCCT
GAAGGTCCGAACATCGGTCTGATCAACTCCCTGGCGGCCTATGCCCGCACCAACCAGTACGGCTTCCTCGAGAGCCCGTACCGTG
TGGTCAAGGAAGGTCTGGTCACCGAAGAGATCGTCTTCCTTTCGGCGATCGAAGAGGCGGACCACGTCATTGCCCAGGCTTCGGC
CGCAATGAACGACAAGCAAGAGCTGATCGACGAGCTGGTTGCTGTGCGTCACTTGAACGAATTCACGGTCAAGGCGCCAGCCGA
TGTCACCCTGATGGACGTTTCGCCCAAGCAGGTTGTCTCGGTAGCCGCATCGCTGATTCCGTTCCTCGAGCACGATGACGCCAACC
GTGCGTTGATGGGTTCGAACATGCAGCGTCAGGCTGTTCCTACTCTGCGTGCCGACAAGCCGCTGGTAGGTACTGGCATGGAGCG
CAACGTTGCTCGCGACTCCGGCGTTTGCGTCGTGGCTCGTCGTGGTGGTGTGATCGACTCGGTTGACGCCAGCCGTATCGTTGTTC
GCGTTGCCGATGATGAAGTTGAAACAGGTGAGGCGGGTGTAGACATCTACAACCTGACCAAATACACCCGTTCGAACCAGAACA
CCTGCATCAACCAGCGTCCGCTGGTCAGCAAGGGTGACCGTGTTCAGCGTAGCGACATCATGGCTGACGGCCCGTCCACCGATAT
GGGTGAACTGGCGCTGGGTCAGAACATGCGCATCGCGTTCATGGCCTGGAACGGTTTCAACTTCGAAGACTCCATCTGTCTTTCTG
AGCGTGTCGTTCAGGAAGACCGCTTCACCACGATCCACATTCAGGAACTGACCTGTGTGGCGCGTGACACCAAGCTTGGGCCAGA
GGAAATCACAGCTGACATCCCTAACGTGGGTGAAGCTGCTCTGAACAAGCTGGACGAAGCGGGTATCGTTTATGTAGGTGCCGAA
GTCGGCGCCGGCGACATCCTGGTCGGTAAGGTCACTCCGAAAGGCGAGACCCAGCTGACTCCGGAAGAAAAACTGTTGCGCGCG
ATCTTCGGTGAGAAGGCGAGCGACGTTAAAGACACCTCCCTGCGCGTACCAACCGGTACCAAAGGTACTGTCATTGACGTTCAGG
TCTTCACCCGTGATGGTGTCGAGCGTGATGCGCGTGCCCTGTCCATCGAGAAATCGCAACTCGACGAGATCCGCAAGGATCTGAA
CGAAGAGTTCCGTATTGTCGAAGGCGCTACTTTCGAACGTCTGCGCTCTGCGCTGGTGGGTCGTGTAGCTGAAGGCGGCGCTGGC
CTCAAGAAGGGCCAGGAAATCACCAACGAAGTGCTCGATGGTCTTGAGCATGGTCAGTGGTTCAAGCTGCGCATGGCTGAAGAC
GCTCTCAACGAGCAGCTCGAAAAGGCTCAGGCTTACATCGTCGATCGTCGCCGTCTTCTGGACGACAAGTTCGAAGACAAGAAAC
GCAAACTGCAGCAGGGCGATGACCTGGCTCCAGGCGTTCTGAAAATCGTCAAGGTCTACCTGGCAATCCGTCGCCGCATCCAGCC

GGGCGATAAGATGGCCGGTCGTCACGGTAACAAGGGTGTGGTCTCTGTGATCATGCCGGTTGAAGACATGCCGCACGATGCCAAT
GGCACGCCGGTTGATATCGTCCTCAACCCGCTGGGCGTACCTTCGCGTATGAACGTTGGTCAGATTCTCGAAACCCACCTGGGCCT
CGCGGCCAAAGGTCTGGGCGAGAAGATCAACCGCATGCTCGAAGAGCAGCGTAAAGTTGCTGAGCTGCGCAAGTTCCTCAACGA
GATCTATAACGAAATCGGCGGTCGTCAGGAGTCTCTGGAAGACCTCACCGACAACGAGATCCTGGACCTCGCGAAGAACCTGCGT
AACGGCGTACCGATGGCTACCCCGGTTTTCGACGGTGCCAAGGAAAGCGAAATCAAGGCAATGCTCAAGCTGGCAGATATGCCG
GAAAGCGGCCAGATGCAGCTGTTCGACGGTCGTACCGGCAACAAGTTTGAACGTGCTGTAACGGTTGGCTACATGTACATGCTGA
AGCTGAACCACTTGGTGGACGACAAGATGCACGCGCGTTCCACTGGTTCGTACAGCCTGGTTACCCAGCAGCCGCTGGGTGGTAA
GGCACAGTTCGGTGGTCAGCGTTTCGGGGAGATGGAAGTGTGGGCGCTGGAAGCATACGGCGCGGCGTACACTCTGCAGGAAAT
GCTCACAGTGAAGTCGGACGATGTGAACGGTCGTACCAAGATGTACAAAAACATCGTGGACGGCGATCACCGTATGGAGCCGGG
CATGCCCGAGTCTTTCAACGTGTTGATCAAAGAAATCCGTTCGCTCGGTATCGATATCGATCTGGAAACCGAATAA

P. syringae pv. tomato str. DC3000_ GCF_000007805.1_ AE016853.1

ATGGCTTACTCATATACTGAGAAAAAACGTATCCGCAAGGACTTTAGCAAGTTGCCGGACGTAATGGATGTGCCGTATCTCTTGG
CCATCCAGCTGGATTCGTATCGCGAATTCCTGCAGGCGGGAGCGACCAAAGATCAGTTCCGCGACGTCGGTCTGCATGCAGCCTT
CAAATCCGTTTTCCCGATCATCAGCTACTCCGGCAATGCTGCGCTGGAGTATGTAGGTTATCGCTTGGGCGAACCGGCATTTGATG
TCAAGGAATGCGTGCTGCGCGGTGTGACTTACGCAGTACCTCTGCGAGTCAAGGTCCGTCTGATCATTTTCGACAAAGAATCGTCG
AACAAAGCGATCAAGGACATCAAAGAGCAAGAAGTCTACATGGGTGAAATCCCCCTGATGACTGAAAACGGTACCTTTGTAATC
AACGGCACCGAGCGCGTTATCGTGTCTCAGCTTCACCGCTCGCCAGGCGTATTCTTCGACCACGACCGTGGCAAGACGCACAGCT
CCGGTAAGCTGCTTTATTCCGCGCGTATCATTCCTTACCGTGGTTCGTGGCTGGACTTCGAGTTCGATCCGAAAGACTGCGTATTC
GTCCGTATCGACCGTCGTCGCAAGCTGCCTGCGTCCGTGCTTCTGCGCGCGCTGGGCTACACCACCGAGCAAGTGCTCGATGCTTT
CTATACCACTAACGTATTCCATGTTCGCGGCGAAAACCTGAACCTGGAGCTGGTGCCTCAGCGCCTGCGTGGTGAAATTGCCGTTC
TGGATATCCTGGACGACAAAGGCAAGGTCATTGTCGAGCAGGGTCGTCGTATCACTGCCCGTCACATCAACCAGCTGGAAAAAGC
CGGGATCAAAGAGCTGGAAGTACCTCTGGACTACGTCCTGGGTCGTACGACTGCCAAGGTCATCGTGCATCCGGCAACCGGTGAG
ATCATTGCCGAGTGCAACACCGAGCTGAACACCGAAATCCTCGGCAAGATTGCCAAGGCTCAGGTTGTTCGCATCGAGACGTTGT
ACACCAACGACATCGATTGCGGCCCGTTCGTTTCGGACACGCTGAAAATCGACTCCACCAGCAATCAGCTGGAAGCGTTGGTCGA
GATCTACCGCATGATGCGTCCTGGTGAGCCACCGACCAAGGATGCAGCAGAAACGCTGTTCAACAACCTGTTCTTCAGTCCTGAG
CGCTATGACCTGTCTGCTGTAGGTCGTATGAAGTTCAACCGTCGTATCGGTCGGACCGAAATCGAAGGTTCGGGCGTGCTGTGCA
AGGAAGACATCGTAGCGGTCCTCAAGACCCTCGTTGATATCCGTAACGGCAAAGGCATCGTCGATGACATCGACCACCTCGGTAA
CCGTCGTGTTCGTTGCGTAGGCGAGATGGCCGAGAACCAGTTCCGTGTAGGTCTGGTCCGTGTTGAGCGTGCAGTCAAAGAACGC
CTTTCCATGGCAGAAAGTGAAGGCCTGATGCCTCAGGACCTGATCAACGCCAAGCCTGTTGCGGCAGCGGTCAAGGAATTCTTCG
GTTCCAGCCAGCTTTCCCAGTTCATGGACCAGAACAACCCGCTGTCCGAGATTACTTGCAAGCGTCGTGTTTCTGCACTCGGCCCT
GGTGGTTTGACTCGTGAGCGTGCCGGCTTTGAAGTTCGAGACGTTCACCCGACTCACTACGGTCGCGTGTGCCCTATCGAAACGCC
GGAAGGTCCGAACATCGGCCTGATCAACTCCCTCGCGGCCTATGCCCGCACCAACCAGTACGGTTTCCTCGAGAGCCCGTACCGT
GTTGTTAAGGAAGGTCTGGTCACCGACGAAATCGTGTTCCTTTCAGCGATCGAAGAAGCTGACCACGTCATTGCACAGGCTTCGG
CCGCAATGAACGACAAGCAAGAGCTGATCGACGAGCTGGTTGCTGTGCGTCACTTGAACGAATTCACCGTCAAGGCGCCAGCCG
ATGTCACCCTGATGGACGTTTCGCCCAAGCAGGTTGTTTCGGTAGCCGCTTCGTTGATTCCGTTCCTCGAGCACGATGACGCCAAC
CGTGCGTTGATGGGTTCGAACATGCAGCGTCAGGCTGTACCAACCTTGCGTGCCGACAAGCCGCTGGTGGGTACCGGCATGGAGC
GCAACGTTGCTCGCGACTCCGGCGTTTGCGTCGTGGCCCGTCGTGGTGGCGTGATCGACTCGGTTGATGCCAGCCGTATCGTTGTT
CGCGTTGCCGATGATGAAGTTGAAACAGGTGAGGCCGGTGTAGACATCTACAACCTGACCAAATACACCCGTTCGAACCAGAAC
ACCTGCATCAACCAGCGTCCGCTGGTAAGCAAGGGTGATCGTGTTCAGCGTAGCGATATCATGGCCGACGGCCCGTCCACCGATA
TGGGTGAGCTGGCGCTGGGTCAGAACATGCGTATCGCGTTCATGGCCTGGAACGGTTTCAACTTCGAAGACTCCATCTGTCTCTCT
GAGCGTGTAGTTCAGGAAGACCGCTTCACCACGATCCACATTCAGGAACTGACCTGTGTGGCGCGTGACACCAAGCTTGGGCCTG
AGGAAATCACAGCTGACATCCCGAACGTGGGTGAAGCTGCTCTGAACAAGCTGGACGAAGCCGGTATCGTTTACGTAGGTGCCGA
AGTCGGCGCTGGCGATATCCTGGTGGGCAAGGTCACCCCGAAAGGCGAGACCCAGCTGACGCCGGAAGAAAAACTGTTGCGCGC
TATCTTCGGTGAGAAGGCCAGCGACGTTAAGGACACTTCCCTGCGTGTGCCTACCGGCACCAAAGGTACTGTCATCGACGTTCAG
GTCTTCACCCGTGATGGTGTCGAGCGTGATGCACGTGCTCTGTCCATTGAGAAGTCGCAGCTCGACGAGATCCGCAAGGATCTGA
ACGAAGAGTTCCGTATTGTCGAAGGCGCTACTTTCGAACGTCTGCGCTCTGCGCTGGTCGGTCGTGTAGCAGAAGGCGGTGCTGG
CCTCAAGAAAGGTCAGGAAATCACCAACGAAGTGCTCGACGGCCTTGAGCATGGTCAGTGGTTCAAGCTGCGCATGGCTGAAGA
CGCTCTGAACGAGCAGCTTGAGAAAGCACAGGCTTACATCGTTGATCGTCGTCGCCTTCTGGATGACAAGTTTGAAGACAAGAAG
CGCAAACTGCAGCAGGGCGATGACCTGGCTCCAGGCGTTCTGAAAATCGTCAAGGTTTACCTGGCAATCCGTCGCCGCATTCAGC
CGGGCGACAAGATGGCCGGTCGTCACGGTAACAAGGGTGTGGTCTCTGTGATCATGCCGGTTGAAGACATGCCGCACGATGCCAA
TGGCACGCCGGTTGATATCGTCCTCAACCCGCTGGGCGTACCTTCGCGTATGAACGTTGGTCAGATTCTCGAAACTCACCTGGGCC
TTGCAGCCAAAGGTCTGGGCGAGAAGATCAACCGCATGCTTGAAGAGCAGCGTAAAGTTATCGAGTTGCGCAAGTTCCTCAACGA
GATCTACAACGAAATCGGTGGTCGCCAGGAAGCCCTGGAAGACCTTACCGACAACGAAATTCTGGACCTCGCCAAGAACCTGCGT
AACGGCGTACCAATGGCTACCCCGGTTTTTGACGGTGCCAAGGAAAGCGAAATCAAGGCAATGCTCAAACTGGCAGATATGCCG
GAAAGCGGCCAGATGCAGCTGTTTGACGGTCGTACCGGCAACAAGTTTGAGCGCCCGGTTACAGTCGGCTACATGTACATGCTGA
AGCTGAACCACTTGGTGGACGACAAGATGCACGCGCGTTCCACTGGTTCGTACAGCCTGGTTACCCAGCAGCCGCTGGGTGGTAA
GGCACAGTTCGGTGGTCAGCGTTTCGGGGAGATGGAAGTGTGGGCGCTGGAAGCATACGGCGCGGCGTACACTCTGCAAGAAAT
GCTCACAGTGAAGTCGGACGATGTGAACGGCCGTACCAAGATGTACAAAAACATCGTGGATGGCGATCACCGTATGGAGCCGGG
CATGCCCGAGTCTTTCAACGTGTTGATCAAAGAAATTCGTTCGCTCGGTATCGATATCGATCTGGAAACCGAATAA

P. toyotomiensis SM2_ GCF_017167985.1_ CP070505.1

ATGGCTTACTCATACACTGAGAAAAAACGTATCCGCAAGGACTTTAGCAAGTTGCCGGATGTCATGGATGTGCCTTACCTCCTGGC
CATCCAGCTGGATTCGTACCGCGAATTCCTGCAGCAAGGGGTGAGCAAGGAACAGTTCCGCGACATCGGCCTGCATGCGGCCTTC
AAATCGGTATTCCCGATCATCAGCTACTCCGGCAACGCCGCCCTGGAGTACGTCGGCTATCGCCTGGGCGAGCCGGCGTTCGACG
TCAAGGAGTGCGTCCTGCGTGGCGTGACCTTCGCCGTGCCGCTGCGCGTGAAAGTGCGCCTGATCATTTTCGACAAAGAATCGTC
GAACAAAGCGATCAAGGACATCAAAGAGCAGGAAGTGTACATGGGCGAGATTCCGCTCATGACCGAGAACGGTACCTTCGTCAT
CAACGGTACCGAGCGTGTGATCGTGTCCCAGCTGCACCGTTCGCCGGGTGTGTTCTTCGACCACGACCGTGGCAAGACCCACAGC
TCGGGCAAGCTGCTGTACTCCGCTCGCATCATTCCTTACCGCGGTTCCTGGCTGGACTTCGAGTTCGATCCGAAGGACGCGGTATT
CGTGCGTATCGACCGTCGCCGCAAACTGCCGGCTTCCGTCCTGCTGCGCGCGCTGGGCTACAGCACTGAAGAAGTACTGGATGCC
TTCTATGACACCAACGTCTTCCATGTGAAGGGCGAGAGCCTGAGTCTGGAGCTGGTTCCGCAGCGCCTGCGCGGCGAAGTCGCCG
TTCTCGACATCAAGGACGGCAGCGGCAAGGTGATCGTGGAACAGGGCCGTCGTATCACGGCTCGTCACATCAACCAGCTGGACA
AGGCTGGTATCAAGGAGCTGGAAGTTCCGCTCGACTACGTCATTGGCCGTACCACTGCCAAGGCGATCGTGCACCCGGCTACCGG
CGAAATCATCGCCGAGTGCAACACCGAGCTGACCGCCGACCTGCTGGTCAAGATGGCCAAGGCGCAGGTGGTTCGTTTCGAGACC
CTGTACACCAACGACATCGACTGTGGTCCGTTCATCAGCGACACGCTGAAGATCGACAGCACCACCAATCAGTTGGAAGCGCTGG
TCGAGATCTACCGCATGATGCGTCCTGGCGAGCCGCCAACCAAGGATGCTGCCGAGACCCTGTTCAACAACCTGTTCTTCAGCGC
CGAGCGTTACGATCTGTCCGCCGTTGGCCGCATGAAGTTCAACCGTCGTATCGGTCGTACCGAGATCGAAGGTTCGGGCGTGCTG
AGCAAGGAAGACATCGTTGCCGTACTGAAGACCCTGGTCGACATCCGTAACGGCAAGGGCATCGTCGACGACATCGACCACCTG
GGTAACCGTCGCGTACGTTGCGTCGGCGAGATGGCCGAGAACCAGTTCCGCGTTGGCCTGGTGCGTGTAGAGCGCGCGGTCAAGG
AACGTCTGTCGATGGCCGAAAGCGAAGGCCTGATGCCGCAAGACCTGATCAACGCCAAGCCGGTTGCGGCAGCGGTGAAGGAGT
TCTTCGGTTCCAGCCAGCTCTCGCAGTTCATGGACCAGAACAACCCGCTGTCCGAGATCACCCACAAGCGCCGCGTTTCCGCACTC
GGCCCAGGTGGTCTGACTCGTGAGCGTGCAGGCTTCGAAGTCCGCGACGTACACCCGACTCACTATGGTCGCGTGTGCCCGATCG
AAACGCCGGAAGGTCCGAACATCGGTCTGATCAACTCGCTGGCTGCCTACGCCCGCACCAACCAGTACGGCTTCCTGGAAAGCCC
GTACCGCGTGGTCAAGGAAGGCAAGGTCACCGACGAGATCGTGTTCCTGTCCGCCATCGAAGAGGCCGATCACGTTATCGCCCAG
GCATCCGCGACCCTGAACGACAAGGGTCAGCTGATCGATGAGCTGGTAGCCGTACGTCACCTGAACGAATTCACCGTCAAGGCGC
CGGAAGACGTCACTCTGATGGACGTTTCGCCGAAGCAGGTCGTTTCCGTCGCTGCCTCGCTGATTCCGTTCCTCGAGCACGACGAC
GCCAACCGCGCCCTCATGGGTTCGAACATGCAGCGCCAGGCTGTGCCGACCCTGCGTGCCGACAAGCCGCTGGTAGGTACCGGCA
TGGAGCGCAACGTCGCCCGTGACTCCGGTGTCTGCGTCGTGGCCCGTCGTGGTGGTGTGATCGACTCCGTCGACGCCAGCCGTATC
GTGGTGCGTGTCAACGATGACGAAGTCGAAACTGGCGAAGCCGGTGTCGACATCTACAACCTGACCAAATACACCCGCTCCAACC
AGAACACCTGCATCAACCAGCGTCCGCTGGTGAGCAAAGGTGACAAGGTGGCGCGTAGCGACATCATGGCTGACGGCCCGTCCA
CCGACATGGGTGAACTGGCGCTGGGTCAGAACATGCGCGTTGCGTTCATGCCGTGGAACGGCTTCAACTTCGAAGACTCCATCTG
CCTGTCCGAGCGCGTGGTCCAGGAAGACCGCTTCACCACCATCCACATCCAGGAACTGACCTGTGTGGCGCGTGACACCAAGCTC
GGCCCAGAGGAAATCTCCTCTGACATCCCGAACGTGGGTGAAGCTGCTCTGAACAAGCTGGATGAAGCCGGTATCGTCTACGTCG
GCGCCGAAGTCGGCCCGGGCGACATCCTGGTCGGTAAGGTCACTCCGAAAGGCGAGACCCAGCTGACGCCGGAAGAGAAGCTGC
TGCGTGCGATCTTCGGTGAGAAAGCCTCCGACGTTAAGGACACTTCCCTGCGCGTGCCGACTGGCACCAAGGGTACCGTCATCGA
CGTGCAGGTCTTCACCCGTGATGGCGTGGAGCGCGACTCGCGCGCCCTGTCCATCGAGAAGATGCAGCTGGACGAGATCCGCAAG
GACCTCAACGAAGAGTTCCGCATCGTCGAAGGCGCGACCTTCGAGCGTCTGCGTTCCGCTCTGGTTGGCGCTATCGCCGAAGGCG
GCGCTGGTCTGAAGAAAGGCACTGCGATCACCGACGAGTTCCTCGACGGTCTTGAGCGTGGCCAGTGGTTCAAACTGCGCATGGC
CGATGATGCCCTGAACGAGCAGCTGGAGAAGGCTCAGGCCTACATCTCCGATCGCCGTCAGATGCTCGACGACAAGTTCGAAGAC
AAGAAGCGCAAGCTGCAGCAGGGCGATGACCTGGCGCCGGGCGTACTGAAGATCGTCAAGGTTTACCTGGCCATCCGCCGTCGC
ATCCAGCCGGGTGACAAGATGGCCGGTCGTCACGGTAACAAGGGTGTGGTGTCGGTGATCATGCCGGTCGAAGACATGCCGCAC
GACGCCAACGGTACTCCGGTCGACATCGTGTTGAACCCGCTGGGCGTTCCGTCGCGTATGAACGTCGGTCAGATTCTCGAAACCC
ACCTAGGCCTCGCAGCCAAGGGTCTGGGCGAGAAGATCAACCGCATGCTCGAAGAGCAGCGCAAGATCGCCGAACTGCGCAAGT
TCCTCGCTGAGATCTACAACGAGATCGGTGGCCGTCAGGAAAACCTCGACGAGTTCTCCGATACCGAGATCCTCGAGCTGGCGAA
GAACCTCAAAGGCGGTGTACCGATGGCGACTGCCGTGTTCGACGGCGCCAAGGAAACCGAGATCAAGGCCATGCTGAAGCTGGC
TGATCTGCCGGAGAGCGGCCAGATGCGCCTGTTCGACGGTCGTACCGGTAACCAGTTCGAGCGCCCGACCACCGTCGGCTACATG
TACATGCTCAAACTGAACCACCTGGTGGACGACAAGATGCACGCCCGTTCCACTGGTTCCTACAGCCTGGTTACCCAGCAGCCGC
TGGGTGGTAAGGCGCAGTTCGGTGGTCAGCGTTTCGGGGAGATGGAGGTCTGGGCACTGGAAGCCTATGGCGCCGCCTACACCCT
GCAGGAAATGCTGACCGTGAAGTCGGACGACGTGAACGGCCGTACCAAGATGTACAAGAACATCGTGGATGGCGATCACCGTAT
GGAGCCGGGCATGCCCGAGTCCTTCAACGTACTGATCAAAGAGATCCGTTCGCTCGGCATCGATATCGATCTGGAAACCGAATAA

P. umsongensis CY-1_ GCF_012647205.1_ CP051487.1

ATGGCTTACTCATATACTGAGAAAAAACGTATCCGCAAGGACTTTAGCAAGTTGCCGGACGTCATGGATGTGCCTTACCTCCTGGC
CATCCAGCTGGATTCGTATCGTGAATTCTTGCAAGCGGGAGCGACTAAAGATCAGTTCCGCGACGTGGGCCTGCATGCGGCCTTC
AAATCCGTTTTCCCGATCATCAGCTACTCCGGCAATGCTGCGCTGGAGTACGTCGGTTATCGCTTGGGCGAACCGGCATTTGATGT
CAAAGAATGCGTATTGCGCGGTGTAACTTACGCCGTACCTTTGCGGGTAAAAGTGCGCCTGATCATTTTCGACAAAGAATCGTCG
AACAAAGCGATCAAGGACATCAAAGAGCAAGAAGTCTACATGGGTGAAATCCCCCTGATGACTGAGAACGGTACCTTCGTAATC
AACGGTACCGAGCGTGTAATCGTTTCCCAGCTGCACCGTTCCCCTGGCGTGTTCTTCGACCACGACCGTGGCAAGACGCACAGCTC
CGGTAAACTGCTGTACTCCGCGCGCATCATTCCTTACCGCGGTTCGTGGCTGGACTTCGAGTTCGACCCGAAAGACTGCGTATTCG
TGCGTATCGACCGTCGTCGCAAGCTGCCTGCATCGGTACTGCTGCGCGCGCTCGGCTATACCACTGAAGAAGTGCTGGACGCGTT
CTACACCACCAACGTATTCCACGTTAAGGGCGAGACCCTGAGCCTGGAGCTGGTGCCTCAGCGCCTGCGCGGTGAAATCGCTGTC
CTCGATATCCAGGACGACAAAGGCAAGGTTATTGTCGAGCAGGGTCGTCGTATCACCGCTCGCCACATCAACCAGCTGGAAAAAG
CCGGGATCAAAGAGCTGGATGTGCCGCTGGACTACGTCCTGGGTCGCACAACCGCCAAGGTCATCGTGCACCCGGCAACCGGCG
AAATCCTGGCAGAGTGCAACACCGAGCTGAACACCGAGATCCTGGCGAAAATCGCCAAGGCCGGCGTTGTTCGCATCGAAACTCT

GTACACCAACGATATCGACTGCGGTCCGTTCGTCTCCGACACACTGAAGATCGACTCCACCAGCAACCAACTGGAAGCGCTGGTC
GAGATCTATCGCATGATGCGTCCTGGCGAGCCGCCAACCAAAGACGCTGCCGAGACCCTGTTCAACAACCTGTTCTTCAGCCCTG
AGCGCTACGACCTGTCTGCGGTCGGCCGGATGAAGTTCAACCGTCGTATCGGTCGTACCGAGATCGAAGGTTCGGGCGTGCTGTG
CAAGGAAGACATCGTCGCGGTACTGAAGACCCTGGTCGACATCCGTAACGGTAAAGGCATCGTCGATGACATCGACCACCTGGGT
AACCGTCGTGTTCGCTGCGTTGGCGAAATGGCCGAGAACCAGTTCCGCGTTGGCCTGGTACGTGTTGAGCGTGCGGTCAAAGAGC
GTCTGTCGATGGCTGAAAGCGAAGGCCTGATGCCGCAAGACCTGATCAACGCCAAGCCAGTGGCTGCGGCGGTGAAAGAGTTCTT
CGGTTCCAGCCAGTTGTCCCAGTTCATGGACCAGAACAACCCGCTGTCCGAGATCACCCACAAGCGTCGTGTCTCTGCACTCGGCC
CTGGTGGTCTGACTCGTGAGCGTGCCGGCTTTGAAGTTCGTGACGTACACCCGACGCACTACGGTCGTGTTTGCCCGATCGAAACG
CCGGAAGGTCCGAACATCGGTCTGATCAACTCCCTGGCTGCCTATGCGCGCACCAACCAGTACGGCTTCCTCGAGAGCCCGTACC
GCGTGGTGAAAGACGCCCTGGTCACCGACGAGATCGTGTTCCTGTCCGCCATCGAAGAAGCTGATCACGTGATCGCCCAGGCTTC
GGCCACGATGAACGACAAGAAAGTCCTGATCGACGAGCTGGTAGCTGTTCGTCACTTGAACGAATTCACCGTCAAGGCGCCGGA
AGACGTCACCTTGATGGACGTATCGCCGAAGCAGGTAGTTTCGGTTGCAGCGTCGCTGATTCCGTTCCTCGAGCACGACGACGCC
AACCGTGCGTTGATGGGTTCGAACATGCAGCGTCAAGCTGTACCAACCCTGCGTGCTGACAAGCCGCTGGTAGGTACCGGCATGG
AGCGTAACGTAGCCCGTGACTCCGGCGTTTGCGTCGTGGCTCGTCGTGGCGGCGTGATCGACTCCGTCGACGCCAGCCGTATCGT
GGTTCGCGTTGCCGATGACGAAGTAGAAACCGGTGAAGCCGGTGTCGACATCTACAACCTGACCAAGTACACCCGCTCCAACCAG
AACACCTGCATCAACCAGCGTCCGCTGGTGCGTAAAGGTGATCGGGTTCAGCGTAGCGACATCATGGCCGACGGTCCGTCCACCG
ACATGGGTGAACTGGCACTGGGTCAGAACATGCGCATCGCCTTCATGGCATGGAACGGCTTCAACTTCGAAGACTCCATCTGCCT
GTCGGAACGCGTTGTTCAAGAAGACCGTTTCACCACGATCCACATCCAGGAACTGACCTGTGTGGCCCGTGACACCAAGCTTGGC
CCAGAGGAAATCACTGCGGACATCCCGAACGTGGGTGAAGCTGCACTGAACAAGCTGGACGAAGCCGGTATCGTTTACGTAGGT
GCTGAAGTTGGCGCAGGCGACATCCTGGTGGGTAAGGTCACTCCGAAAGGCGAGACCCAACTGACTCCGGAAGAAAAACTGCTG
CGTGCGATCTTCGGTGAAAAAGCCAGCGACGTTAAAGACACCTCCCTGCGCGTACCTACCGGTACCAAAGGTACTGTCATTGACG
TACAAGTCTTCACCCGCGACGGCGTTGAGCGTGATGCGCGTGCCCTGTCGATCGAGAAGTCCCAGCTGGACGAGATCCGCAAGGA
CCTCAACGAAGAGTTCCGTATCGTCGAAGGTGCTACTTTCGAACGTCTGCGCTCCGCTCTGGTCGGCCACAAAGCCGAAGGCGGC
GCCGGTCTGAAGAAGGGCCAGGACATCACCGACGAAGTGCTCGACGGTCTTGAGCACGGCCAGTGGTTCAAACTGCGCATGGCT
GAAGATGCCCTGAACGAGCAGCTCGAGAAGGCCCAGGCCTACATCGTTGATCGTCGCCGTCTGCTGGACGACAAGTTCGAAGAC
AAGAAGCGCAAACTGCAGCAGGGCGATGACCTGGCTCCAGGCGTGCTGAAAATCGTCAAGGTTTACCTGGCAATCCGTCGTCGCA
TCCAGCCGGGCGACAAGATGGCCGGTCGTCACGGTAACAAGGGTGTGGTCTCCGTGATCATGCCGGTTGAAGACATGCCGCACGA
TGCCAATGGCACCCCGGTCGACGTCGTCCTCAACCCGTTGGGCGTACCTTCGCGTATGAACGTTGGTCAGATCCTTGAAACCCACC
TGGGCCTCGCGGCCAAAGGTCTGGGCGAGAAGATCAACCGCATGGTTGAAGAGCAGCGCAAAGTTGCCGAGCTGCGTAAGTTCC
TGCACGAGATCTACAACGAGATCGGCGGTCGTCAGGAAAGCCTCGATGACTTCTCGGACCAGGAAATCCTGGACCTGGCTCAAAA
CCTGCGTGGCGGCGTTCCAATGGCCACTCCAGTGTTCGACGGCGCCAAGGAAAGCGAAATCAAGGCCATGCTGAAACTGGCAGA
CCTGCCGGAAAGCGGCCAGATGCAGCTGACCGACGGCCGTACCGGCAACAAGTTCGAGCGCCCGGTTACCGTTGGCTACATGTAC
ATGCTGAAGCTGAACCACTTGGTAGACGACAAGATGCACGCGCGTTCTACCGGTTCGTACAGCCTGGTTACCCAGCAGCCGCTGG
GTGGTAAGGCGCAGTTCGGTGGTCAGCGTTTCGGGGAGATGGAGGTCTGGGCACTGGAAGCATACGGTGCCGCTTACACTCTGCA
AGAAATGCTCACAGTGAAGTCGGACGATGTGAACGGCCGTACCAAGATGTACAAGAACATCGTGGATGGCGATCACCGTATGGA
GCCGGGCATGCCCGAGTCCTTCAACGTGTTGATCAAGGAAATTCGTTCCCTCGGCATCGATATCGATCTGGAAACCGAATAA

P. versuta L10.10_ GCF_001294575.1_ CP012676.1

ATGGCTTACTCATATACTGAGAAAAAACGTATCCGCAAGGACTTTAGCAAGTTGCCGGACGTCATGGATGTGCCGTATCTCTTGGC
AATCCAGCTGGATTCGTATCGTGAATTCTTGCAGGCGGGAGCGACTAAAGATCAGTTCCGCGACGTGGGCCTGCATGCGGCCTTC
AAATCCGTTTTCCCGATCATCAGCTACTCCGGCAATGCTGCGCTGGAGTACGTCGGTTATCGCTTGGGCGAGCCGGCATTTGATGT
TAAAGAATGCGTGTTGCGCGGTGTAACTTACGCCGTACCTTTGCGGGTAAAAGTTCGTTTGATCATTTTCGACAAAGAATCGTCGA
ACAAAGCGATCAAGGACATCAAAGAGCAAGAAGTCTACATGGGTGAAATCCCCCTGATGACTGAGAACGGTACCTTTGTAATCA
ATGGCACCGAGCGTGTAATTGTTTCCCAGTTGCACCGTTCCCCGGGCGTGTTCTTTGACCACGACCGTGGCAAAACGCATAGCTCC
GGTAAACTGCTTTATTCCGCGCGTATCATTCCTTACCGTGGTTCGTGGTTGGACTTTGAGTTCGATCCGAAAGACTGCGTGTTCGTA
CGTATTGACCGTCGTCGCAAGCTGCCTGCATCGGTATTGCTGCGCGCGCTGGGTTATACGACTGAGCAGGTGCTGGACGCGTTCTA
CACCACCAACGTATTCCACGTTCAGGGCGAAAGTATTAGCCTGGAACTGGTACCTCATCGCCTGCGCGGTGAGATCGCGGCCATC
GATATTACCGATGACAAAGGCAAGGTGATTGTTGAGCAGGGTCGTCGTATCACTGCTCGTCATATCAACCAGCTGGAAAAAGCCG
GTATCAAAGAGCTCGTTATGCCTCTGGACTATGTCCTGGGTCGCACAACGGCCAAGGCTATCGTGCATCCGGCTACCGGCGAAAT
CATTGCTGAGTGCAACACCGAGCTGACCACCGAGATTCTGGCGAAAATCGCCAAGAGCCAGGTTGTCCGTATCGAAACGTTGTAC
ACCAACGATATCGACTGCGGTCCGTTCGTCTCCGACACTCTGAAGATCGACTCCACCAGCAACCAATTGGAAGCGCTGGTCGAGA
TCTATCGCATGATGCGTCCAGGCGAGCCGCCAACCAAAGACGCTGCCGAGACTCTGTTCAACAACCTGTTCTTCAGCCCTGAGCG
CTATGACCTGTCTGCGGTCGGCCGGATGAAGTTCAACCGTCGTATCGGTCGTACCGAGATCGAAGGTTCGGGCGTGTTGTGCAAA
GAAGACATCGTCGCGGTACTGAAGACTCTGGTCGACATCCGTAACGGTAAAGGCATCGTCGATGACATCGACCACCTGGGTAACC
GTCGTGTTCGCTGTGTTGGCGAAATGGCTGAAAACCAGTTCCGCGTTGGCCTGGTACGTGTTGAGCGTGCGGTCAAAGAGCGTCT
GTCGATGGCTGAAAGCGAAGGCCTGATGCCGCAAGACCTGATCAACGCCAAGCCAGTGGCTGCGGCGGTGAAGGAGTTCTTCGG
TTCCAGCCAGCTCTCGCAGTTCATGGACCAGAACAACCCTCTGTCCGAGATCACCCACAAGCGCCGTGTTTCTGCACTGGGCCCGG
GCGGTCTGACGCGTGAGCGTGCAGGCTTTGAAGTTCGTGACGTACACCCGACTCACTACGGTCGTGTATGCCCGATTGAAACGCC
GGAAGGTCCAAACATCGGTCTGATCAACTCCCTGGCCGCCTATGCGCGCACCAACCAGTACGGCTTCCTTGAGAGCCCGTATCGC
GTGGTGAAAGACGCTTTGGTCACCGACGAGATCGTGTTCCTGTCCGCCATCGAAGAAGCCGATCACGTGATCGCTCAGGCCTCGG
CCACGATGAACGACAAGAAAGTCCTGATCGACGAACTGGTAGCTGTTCGTCACTTGAACGAATTCACCGTCAAGGCGCCGGAAG
ACGTCACCCTGATGGACGTTTCGCCGAAGCAGGTTGTGTCGGTTGCAGCGTCGTTGATTCCGTTCCTCGAGCATGATGACGCCAAC
CGTGCGTTGATGGGTTCGAACATGCAGCGTCAAGCTGTACCTACCCTGCGCGCTGACAAGCCGCTGGTTGGTACCGGCATGGAGC
GTAACGTAGCCCGTGACTCCGGCGTTTGCGTCGTGGCTCGTCGTGGTGGCGTGATCGACTCCGTTGATGCAAGCCGTATCGTGGTA

CGTGTTGCTGATGACGAAGTAGAAACTGGCGAAGCCGGTGTCGACATCTACAACCTGACCAAATACACCCGCTCCAACCAGAACA
CCTGCATTAACCAGCGTCCGCTGGTTCGCAAGGGTGATCGCGTGCAGCGCAGCGACATCATGGCCGATGGTCCGTCCACCGATAT
GGGTGAGCTGGCTTTGGGTCAGAACATGCGCATCGCGTTCATGGCCTGGAACGGTTACAACTTCGAAGACTCCATCTGCTTGTCGG
AACGGGTTGTTCAAGAAGATCGCTTTACCACGATCCACATCCAGGAACTGACCTGTGTGGCACGTGACACCAAGCTTGGGCCTGA
AGAGATCACTGCAGACATCCCTAACGTGGGTGAAGCTGCACTGAACAAACTGGACGAAGCCGGTATCGTTTACGTTGGTGCTGAA
GTTGGCGCGGGCGACATTCTGGTAGGTAAGGTCACTCCGAAAGGCGAGACCCAGCTGACTCCGGAAGAGAAGCTGTTGCGTGCA
ATCTTCGGTGAAAAAGCCAGCGACGTTAAAGACACCTCCCTGCGCGTACCTACCGGTACCAAAGGTACTGTCATCGACGTGCAGG
TCTTCACCCGCGATGGCGTTGAGCGTGATGCTCGTGCCCTGTCGATCGAGAAGACTCAGCTGGACGAGATCCGCAAGGATCTGAA
CGAAGAGTTCCGTATCGTTGAAGGCGCCACTTTTGAACGTCTGCGCTCTGCACTGGTTGGCCGTATAGCCGAAGGTGGAGCCGGT
CTGAAGAAAGGTCAGGAAATCACCAATGAAATCCTGGACGGTCTTGAGCATGGTCAGTGGTTCAAACTGCGCATGGCTGAAGATG
CTCTGAACGAGCAGCTTGAAAAAGCCCAGGCTTACATCATCGATCGCCGTCGTCTGCTGGACGACAAGTTCGAAGACAAGAAGCG
CAAACTGCAGCAGGGCGATGACCTGGCTCCGGGCGTGCTGAAAATCGTCAAGGTTTACCTGGCAATCCGTCGTCGCATCCAGCCG
GGTGACAAGATGGCTGGTCGTCACGGTAACAAAGGTGTGGTCTCCGTGATCATGCCGGTTGAAGACATGCCGTACGATGCCAATG
GCACCCCGGTCGATGTGGTCCTCAACCCGTTGGGCGTACCTTCGCGTATGAACGTTGGTCAGATTCTCGAAACTCACCTGGGCCTC
GCAGCCAAAGGTTTGGGCGAGAAGATCAACCTCATGATTGAAGAGCAGCGCAAGGTTGCTGACCTGCGCAAGTTCCTGCATGAG
ATCTACAACGAGATTGGCGGTCGTCAAGAAAGCCTGGATGACTTCTCCGACCAGGAAATCCTGGATCTGGCGAAGAACCTTCGTG
GCGGTGTGCCGATGGCTACCCCGGTGTTCGACGGTGCCAAGGAAAGCGAAATCAAGGCAATGTTGCGCCTGGCAGATCTGCCGG
ACAGCGGCCAGATGACGCTGACTGACGGCCGTACCGGCAACCAGTTTGAACGTCCGGTTACTGTTGGCTACATGTACATGCTGAA
GCTGAACCACTTGGTAGACGACAAGATGCACGCTCGTTCTACCGGTTCTTACAGCTTGGTTACCCAGCAGCCGCTGGGTGGTAAG
GCACAGTTCGGTGGTCAGCGTTTCGGGGAGATGGAGGTTTGGGCGCTGGAAGCATACGGCGCGGCATACACTCTGCAAGAAATGC
TCACAGTGAAGTCGGATGATGTGAACGGTCGTACCAAGATGTACAAAAACATCGTGGACGGCGATCACCGTATGGAGCCGGGCA
TGCCCGAGTCCTTTAACGTGTTGATCAAAGAAATTCGTTCCCTCGGCATCGATATCGATCTGGAAACCGAATAA

P. viciae 11K1_ GCF_004786035.1_ CP035088.1

ATGGCTTACTCATATACTGAGAAAAAACGTATCCGCAAGGACTTTAGCAAGTTGCCGGACGTCATGGATGTGCCGTACCTCCTGG
CCATCCAGCTGGATTCGTATCGTGAATTCTTGCAAGCGGGAGCGACTAAAGATCAGTTCCGCGACGTGGGCCTGCATGCGGCCTT
CAAATCCGTTTTCCCGATCATCAGCTACTCCGGCAATGCTGCGCTGGAGTACGTCGGTTATCGCCTGGGCGAGCCGGCATTTGATG
TCAAAGAATGCGTATTGCGCGGTGTAACTTACGCCGTACCTTTGCGGGTAAAAGTGCGCCTGATCATTTTCGACAAAGAATCGTCG
AACAAAGCGATCAAGGACATCAAAGAGCAAGAAGTCTACATGGGTGAAATCCCCCTGATGACTGAGAACGGTACCTTCGTAATC
AACGGTACCGAGCGTGTAATCGTTTCCCAGCTGCACCGTTCTCCGGGCGTGTTCTTCGACCACGACCGTGGCAAAACGCACAGCT
CCGGCAAACTGCTTTACTCCGCGCGCATCATTCCTTACCGCGGTTCGTGGTTGGACTTCGAGTTCGATCCGAAAGACTGCGTATTC
GTGCGTATCGACCGTCGTCGCAAGCTGCCTGCATCGGTACTGCTGCGCGCGCTCGGTTATACCACTGAAGAAGTATTGGACGCGTT
CTACACCACCAACGTTTTCCACGTGCAGGGTGAAAACCTCAGCCTGGAGCTGGTGCCTCAGCGTCTGCGCGGTGAAATCGCTGTC
CTCGATATCCAGGATGACAAGGGCAAGGTTATTGTCGAGCAGGGTCGTCGTATTACCGCTCGCCACATCAACCAGCTGGAAAAAG
CCGGGATCAAAGAGCTGCAGGTGCCTCTGGACTACGTCCTGGGTCGCACCACCGCCAAGGTCATCGTGCATCCGGCCACCGGCGA
AATCCTGGCAGAGTGCAACACCGAGCTGAACACCGAGATCCTGGCAAAAATCGCCAAGGCCCAGGTTGTTCGCATCGAGACTCTG
TACACCAACGATATCGACTGCGGTCCGTTTATCTCCGACACGCTGAAGATCGACTCCACTGGCAACCAACTGGAAGCCTTGGTCG
AGATCTATCGCATGATGCGTCCTGGCGAGCCGCCAACCAAGGATGCAGCCGAGACTCTGTTCAACAACCTGTTCTTCAGCCCTGA
GCGCTACGACCTGTCTGCGGTCGGCCGGATGAAGTTCAACCGTCGTATCGGTCGTACCGAGATCGAAGGTTCGGGCGTGTTGAAT
AAAGACGACATCGTTGCGGTGCTCAAGACTCTGGTCGACATCCGTAACGGCAAAGGCATCGTCGATGACATCGACCACCTGGGTA
ACCGTCGTGTTCGCTGTGTAGGCGAAATGGCCGAGAACCAGTTCCGTGTTGGCCTGGTGCGTGTAGAGCGCGCGGTCAAGGAACG
TCTGTCGATGGCTGAAAGCGAAGGCCTGATGCCGCAAGACTTGATCAACGCCAAGCCAGTGGCTGCGGCGGTGAAGGAGTTCTTC
GGTTCGAGCCAGCTGTCTCAGTTCATGGACCAGAACAACCCGCTGTCCGAGATCACCCACAAGCGTCGTGTCTCTGCACTCGGCC
CGGGTGGTTTGACCCGTGAGCGCGCAGGCTTCGAAGTTCGTGACGTACACCCGACTCACTACGGTCGTGTATGCCCGATTGAAAC
GCCGGAAGGTCCTAACATCGGTCTGATCAACTCCTTGGCCGCCTATGCGCGCACCAACCAGTATGGCTTCCTCGAGAGCCCATAC
CGTGTGGTGAAAGACGCCCTGGTCACTGACGAGATCGTATTCCTGTCCGCCATCGAAGAAGCCGATCACGTGATCGCTCAGGCTT
CGGCCACGATGAACGACAAGAAAGTCCTGATCGACGAGCTGGTAGCTGTTCGTCACCTGAACGAGTTCACCGTCAAGGCGCCGG
AAGACGTCACCTTGATGGACGTGTCGCCGAAGCAGGTCGTTTCGGTTGCAGCGTCGCTGATCCCGTTCCTCGAGCATGACGACGC
CAACCGTGCGTTGATGGGTTCGAACATGCAGCGTCAAGCTGTACCAACCCTGCGCGCCGACAAGCCGCTGGTAGGTACCGGCATG
GAGCGAAACGTAGCCCGTGACTCCGGCGTTTGCGTCGTGGCTCGTCGTGGTGGCGTGATCGACTCCGTTGATGCCAGCCGTATCGT
GGTTCGTGTTGCCGATGACGAAGTTGAAACGGGCGAAGCCGGTGTCGACATCTACAACCTTACCAAATACACCCGCTCCAACCAG
AACACCTGCATCAACCAGCGTCCGCTGGTGAGCAAGGGTGATCGGGTTCAGCGTAGCGACATCATGGCCGATGGTCCGTCCACCG
ATATGGGTGAGCTGGCACTGGGTCAGAACATGCGCATCGCGTTCATGGCATGGAACGGCTTCAACTTCGAAGACTCCATCTGCCT
GTCCGAGCGTGTTGTTCAGGAAGATCGCTTCACCACGATCCACATCCAGGAACTGACCTGTGTGGCTCGTGACACCAAGCTTGGC
CCAGAGGAAATCACTGCAGACATCCCGAACGTGGGTGAGGCTGCACTGAACAAGCTGGACGAAGCCGGTATCGTTTACGTAGGT
GCTGAAGTAGGCGCAGGCGACATCCTGGTCGGTAAGGTCACTCCGAAAGGCGAGACCCAGCTGACTCCGGAAGAGAAACTGCTG
CGTGCCATCTTCGGTGAAAAAGCCAGCGACGTTAAAGACACCTCCCTGCGCGTGCCTACCGGCACCAAGGGTACCGTCATCGACG
TACAGGTCTTCACCCGTGACGGCGTTGAGCGTGATGCTCGTGCACTGTCCATCGAGAAGACTCAACTCGACGAGATCCGCAAGGA
CCTGAACGAAGAGTTCCGTATCGTTGAAGGCGCAACTTTCGAGCGTCTGCGCTCCGCTCTGGTCGGCCACAAAGCCGAAGGCGGC
GCCGGCCTGAAGAAAGGTCAGGAAATCACCGACGAAGTTCTCGACGGTCTTGAGCACGGCCAGTGGTTCAAACTGCGCATGGCT
GAAGATGCTCTGAACGAGCAGCTCGAGAAGGCCCAGGCCTATATCGTTGATCGCCGCCGTCTGCTGGACGACAAGTTCGAAGACA
AGAAGCGCAAGCTGCAGCAGGGCGATGACCTGGCTCCGGGCGTGCTGAAAATCGTCAAGGTTTACCTGGCAATCCGTCGTCGCAT

CCAGCCGGGCGACAAGATGGCCGGTCGTCACGGTAACAAGGGTGTGGTCTCCGTGATCATGCCGGTTGAAGACATGCCGCACGAT
GCCAATGGCACCCCGGTCGATGTGGTCCTCAACCCGTTGGGCGTACCTTCGCGTATGAACGTTGGTCAGATCCTTGAAACCCACCT
GGGCCTCGCGGCCAAAGGTCTGGGCGAGAAGATCAACCGTATGATCGAAGAGCAGCGCAAGGTCGCTGACCTGCGTAAGTTCCT
GCACGAGATCTACAACGAGATCGGCGGGCGCAACGAAGAGCTGGACACCTTCACCGACCAGGAAATCCTGGACTTGGCGAAGAA
CCTGCGCGGCGGCGTTCCAATGGCTACTCCGGTGTTCGACGGTGCCAAGGAAAGCGAAATCAAGGCCATGCTGAAACTGGCAGA
CCTGCCAGAAAGTGGCCAGATGCAGCTGTTCGACGGCCGTACCGGCAACAAGTTCGAGCGCCCGGTTACCGTTGGCTACATGTAC
ATGCTGAAGCTGAACCACTTGGTGGACGACAAGATGCACGCTCGTTCTACCGGTTCTTACAGCCTGGTTACCCAGCAGCCGCTGG
GTGGTAAGGCGCAGTTCGGTGGTCAGCGTTTCGGGGAGATGGAGGTCTGGGCACTGGAAGCGTACGGTGCTGCATACACTCTGCA
AGAAATGCTCACAGTGAAGTCGGACGATGTGAACGGCCGGACCAAGATGTACAAAAACATCGTGGACGGCGATCACCGTATGGA
GCCGGGCATGCCCGAGTCTTTCAACGTGTTGATCAAGGAAATTCGTTCCCTCGGCATCGATATCGATCTGGAAACCGAATAA
