# Supplementary material for: Genome Mining of Pseudomonas Species: Diversity and Evolution of Metabolic and Biosynthetic Potential
Source: Molecules. 2021 Dec 12;26(24):7524. doi: 10.3390/molecules26247524 (PMC8704066; doi:10.3390/molecules26247524)
Supplement: Supplementary file 1 [file molecules-26-07524-s001.zip › Supplementary file 2.pdf]

P. fluorescens UK4_GCF_000730425.1_CP008896.1

ATGGCTTACTCATATACTGAGAAAAAACGTATCCGCAAGGACTTTAGCAAGTTGCCGGACGTCATGGATGTCCCGTACCTTCTGGC
TATCCAGCTGGATTCGTATCGTGAATTCTTGCAGGCGGGAGCGACCAAAGATCAGTTCCGCGACGTGGGCCTGCATGCGGCCTTC
AAATCCGTTTTCCCGATCATCAGCTACTCCGGCAATGCTGCGCTGGAGTACGTCGGTTATCGCCTGGGCGAACCGGCATTTGATGT
CAAAGAATGCGTGTTGCGCGGTGTTACGTACGCCGTACCTTTGCGGGTAAAAGTCCGTCTGATCATTTTCGACAAAGAGTCGTCGA
ACAAAGCGATCAAGGACATCAAAGAGCAAGAAGTCTACATGGGCGAAATCCCGTTGATGACTGAGAACGGTACCTTCGTTATCA
ACGGTACCGAGCGTGTAATCGTTTCCCAGCTGCACCGTTCCCCGGGCGTGTTCTTTGACCACGACCGCGGTAAGACGCACAGCTCC
GGCAAGCTCCTGTATTCCGCGCGGATCATTCCGTACCGCGGTTCATGGTTGGACTTCGAGTTCGACCCGAAGGACTGCGTGTTCGT
GCGTATCGACCGTCGTCGCAAGCTGCCAGCCTCGGTACTGTTGCGCGCGCTCGGCTATACCACTGAGCAAGTGCTGGACGCGTTCT
ACACCACCAACGTATTTAGCCTGAAGGATGAAACCCTCAGCCTGGAGCTGATTGCTTCGCGTCTGCGTGGTGAAATTGCCGTCCTG
GACATTCAGGATGAAAAGGGCAAGGTCATTGTTGAGGCTGGCCGTCGTATTACTGCGCGCCACATCAACCAGATCGAAAAAGCCG
GTATCAAGTCGCTGGACGTGCCCCTGGACTACGTCCTGGGTCGCACTACCGCCAAGGTCATCGTACACCCAGCAACAGGCGAGAT
CCTGGCTGAGTGCAACACCGAGCTGAACACCGAGATCCTGGCGAAAATCGCCAAGGCCCAGGTTGTTCGCATCGAGACCTTGTAC
ACCAACGATATCGACTGCGGTCCGTTCGTCTCCGACACCCTGAAGATCGACTCCACCAGCAACCAATTGGAAGCGCTGGTCGAGA
TCTATCGCATGATGCGTCCTGGCGAGCCGCCAACCAAAGACGCTGCCGAGACCCTGTTCAACAACCTGTTCTTCAGCCCTGAGCG
CTATGACTTGTCTGCGGTCGGCCGGATGAAGTTCAACCGTCGTATCGGTCGTACCGAGATCGAAGGTTCGGGCGTGTTGTGCAAG
GAAGACATCGTTGCGGTCCTGAAGACTCTGGTCGACATCCGTAATGGCAAAGGCATCGTCGATGACATCGACCACCTGGGTAACC
GTCGTGTTCGCTGCGTAGGCGAAATGGCCGAGAACCAGTTCCGCGTTGGCCTGGTACGTGTTGAGCGTGCGGTCAAAGAGCGTCT
GTCGATGGCTGAAAGCGAAGGCCTGATGCCGCAAGACCTGATCAACGCCAAGCCAGTGGCTGCGGCGGTGAAAGAGTTCTTCGG
TTCCAGCCAGCTTTCCCAGTTCATGGACCAGAACAACCCGCTCTCCGAGATCACCCACAAGCGCCGTGTTTCTGCACTGGGCCCGG
GCGGTCTGACTCGTGAGCGTGCTGGCTTTGAAGTTCGTGACGTGCACCCGACGCACTACGGTCGTGTTTGCCCGATCGAAACACC
GGAAGGTCCGAACATCGGTCTGATCAACTCCCTGGCTGCCTATGCGCGCACCAACCAGTACGGCTTCCTCGAAAGCCCGTACCGT
GTGGTGAAAGACGCCTTGGTCACCGACGAGATCGTGTTCCTGTCCGCCATCGAAGAGGCTGATCACGTGATCGCCCAGGCTTCGG
CCACGATGAACGATAAGAAAGTCCTGATCGACGAGCTGGTAGCTGTTCGTCACTTGAACGAGTTCACTGTCAAGGCGCCAGAAGA
CGTCACCTTGATGGACGTCTCGCCCAAGCAGGTAGTTTCGGTTGCAGCGTCGCTGATCCCGTTCCTGGAGCACGATGACGCCAACC
GTGCGTTGATGGGTTCCAACATGCAGCGCCAAGCTGTACCGACCCTGCGCGCTGACAAGCCGCTGGTAGGTACCGGTATGGAGCG
CAACGTGGCCCGTGACTCCGGCGTTTGCGTCGTGGCTCGTCGTGGCGGCGTGATCGACTCCGTTGATGCCAGCCGTATCGTGGTTC
GTGTTGCCGATGATGAAGTTGAAACTGGCGAAGCCGGTGTCGACATCTACAACCTGACCAAATACACCCGTTCGAACCAGAACAC
CTGCATCAACCAGCGTCCGCTGGTGAGCAAGGGTGATCGCGTTCAGCGTAGCGACATCATGGCCGACGGCCCGTCCACCGATATG
GGTGAACTGGCTCTGGGGCAGAACATGCGTATCGCGTTCATGGCATGGAACGGCTTCAACTTCGAAGACTCCATCTGCCTGTCCG
AGCGTGTGGTTCAGGAAGACCGCTTCACCACGATCCACATTCAGGAACTGACCTGTGTGGCGCGTGACACCAAGCTTGGGCCTGA
GGAAATCACCGCAGACATCCCGAACGTGGGTGAAGCTGCTCTGAACAAGCTGGACGAAGCCGGTATCGTTTACGTAGGTGCTGAA
GTCGGCGCTGGCGACATTCTGGTTGGTAAGGTCACTCCGAAAGGCGAGACCCAACTGACTCCGGAAGAAAAACTGCTGCGTGCCA
TCTTCGGTGAAAAAGCCAGCGACGTTAAAGACACTTCCCTGCGCGTACCTACCGGTACCAAGGGTACTGTCATCGACGTGCAGGT
CTTCACCCGCGACGGTGTTGAGCGTGATGCTCGTGCGCTGTCCATCGAGAAGACCCAGCTCGACGAGATCCGCAAGGATCTGAAC
GAAGAGTTCCGTATCGTTGAAGGCGCGACCTTCGAGCGTCTGCGTTCCGCCCTGGTAGGCCGCAAGGCTGAAGGCGGTGCAGGTC
TGAAGAAAGGTCAGGACATCACCGACGAAATCCTCGACGGTCTTGAGCACGGCCAGTGGTTCAAACTGCGCATGGCTGAAGACG
CTCTGAACGAGCAGCTCGAGAAGGCCCAGGCCTACATCGTTGATCGCCGCCGTCTGCTGGACGACAAGTTTGAAGACAAGAAGC
GCAAACTGCAGCAGGGCGATGACCTGGCTCCGGGTGTGCTGAAGATCGTCAAGGTTTACCTGGCAATCCGTCGTCGCATCCAGCC
GGGCGACAAGATGGCCGGTCGTCACGGTAACAAGGGTGTGGTCTCCGTGATCATGCCGGTTGAAGACATGCCGCACGATGCCAAT
GGCACCCCGGTCGATGTCGTCCTCAACCCGTTGGGCGTACCTTCGCGTATGAACGTTGGTCAGATCCTTGAAACCCACCTGGGCCT
CGCGGCCAAAGGTCTGGGCGAGAAGATCAACCGTATGATCGAAGAGCAGCGCAAGGTCGCAGACCTGCGTAAGTTCCTGCACGA
GATCTACAACGAGATCGGCGGTCGCAACGAAGAGCTGGACACCTTCTCCGACCAGGAAGTCCTGGATCTGGCGAAGAACCTGCG
CGGCGGCGTTCCAATGGCTACCCCGGTGTTCGACGGTGCCAAGGAAAGCGAAATCAAGGCCATGCTGAAACTGGCAGACCTGCC
AGAAAGCGGCCAGATGCAGCTGTTCGACGGCCGTACCGGCAACAAGTTCGAGCGCCCGGTTACTGTTGGCTACATGTACATGCTG
AAGCTGAACCACTTGGTAGACGACAAGATGCACGCTCGTTCTACCGGTTCGTACAGCCTCGTTACCCAGCAGCCGCTGGGTGGTA
AGGCTCAGTTCGGTGGTCAGCGTTTCGGGGAGATGGAGGTCTGGGCACTGGAAGCATACGGTGCGGCATACACTCTGCAAGAAAT
GCTCACAGTGAAGTCGGACGATGTGAACGGTCGTACCAAGATGTACAAAAACATCGTGGACGGCGATCACCGTATGGAGCCGGG
CATGCCCGAGTCCTTCAACGTGTTGATCAAAGAAATTCGTTCCCTCGGCATCGATATCGATCTGGAAACCGAATAA

P. fluorescens FW300-N2E3_GCF_001307155.1_CP012830.1

ATGGCTTACTCATATACTGAGAAAAAACGTATCCGCAAGGACTTTAGCAAGTTGCCGGACGTCATGGATGTGCCGTACCTCCTGG
CCATCCAGCTGGATTCGTATCGTGAATTCTTGCAAGCGGGAGCGACTAAAGATCAGTTCCGCGACGTGGGCCTGCATGCGGCCTT
CAAATCCGTTTTCCCGATCATCAGCTACTCCGGCAATGCTGCGCTGGAGTACGTCGGTTATCGCCTGGGCGAACCGGCATTTGATG
TCAAAGAATGCGTATTGCGCGGTGTGACTTACGCCGTACCTTTGCGGGTAAAAGTGCGCCTGATCATTTTCGACAAAGAATCGTCG
AACAAAGCGATCAAGGACATTAAAGAGCAAGAAGTCTACATGGGTGAAATCCCCCTGATGACTGAGAACGGTACCTTCGTAATC
AACGGTACCGAGCGTGTAATCGTTTCCCAGCTGCACCGTTCCCCGGGCGTGTTCTTCGACCACGACCGTGGCAAGACGCACAGCT
CCGGCAAACTGCTGTACTCCGCGCGGATCATTCCTTACCGCGGTTCGTGGCTGGACTTCGAGTTCGACCCGAAAGACTGCGTATTC
GTGCGTATCGACCGTCGTCGCAAGCTGCCTGCATCGGTACTGCTGCGCGCGCTCGGCTATACCACTGAAGAAGTGCTGGACGCGT
TCTACACCACCAACGTCTTCCACGTGCAAGGTGAAAACCTCAGCCTGGAACTGGTGCCTCAGCGCCTGCGTGGTGAAATTGCTGT
CCTGGATATCCTGGATGACAAAGGCAAGGTTATTGTCGAGCAAGGTCGCCGTATCACCGCTCGCCACATCAACCAGCTGGAAAAG
GCCGGGATCAAAGAGCTGCAAGTGCCTCTGGACTACGTCCTGGGTCGCACTACCGCCAAGGTCATCGTGCATCCGGCAACCGGCG
AAATCCTGGCAGAGTGCAACACCGAGCTGAACACCGAGATCCTGGCGAAAATCGCCAAGGCTCAGGTCGTTCGCATCGAGACTCT

GTACACCAACGATATCGACTGCGGTCCGTTCGTCTCCGACACGCTGAAGATCGACTCCACCAGCAACCAATTGGAAGCGCTGGTC
GAGATCTATCGCATGATGCGTCCTGGCGAGCCGCCAACCAAAGACGCTGCCGAGACTCTGTTCAACAACCTGTTCTTCAGCCCTG
AGCGCTATGACCTGTCTGCGGTCGGCCGGATGAAGTTCAACCGTCGTATCGGTCGTACCGAGATCGAAGGTTCGGGCGTGTTGTG
CAAAGAAGACATCGTCGCGGTACTGAAGACTCTGGTCGACATCCGTAACGGTAAAGGCATCGTCGATGACATCGACCACCTGGGT
AACCGTCGTGTTCGCTGCGTAGGCGAAATGGCCGAGAACCAGTTCCGCGTTGGCCTGGTACGTGTTGAGCGTGCGGTCAAAGAGC
GTCTGTCGATGGCCGAAAGCGAAGGCCTGATGCCGCAAGACCTGATCAACGCCAAGCCAGTGGCTGCTGCGGTGAAAGAGTTCTT
CGGTTCGAGCCAGCTGTCCCAGTTCATGGACCAGAACAACCCGCTGTCCGAGATCACCCACAAGCGTCGTGTCTCTGCACTCGGC
CCTGGCGGTCTGACGCGTGAGCGTGCCGGCTTTGAAGTTCGTGACGTACACCCGACTCACTACGGTCGTGTATGCCCGATTGAAA
CACCGGAAGGTCCGAACATCGGTCTGATCAACTCCCTGGCCGCCTATGCGCGCACCAATCAGTACGGCTTCCTCGAGAGCCCGTA
CCGCGTGGTGAAAGACGCTCTGGTCACCGACGAGATCGTGTTCCTGTCCGCCATCGAAGAAGCTGATCACGTGATCGCTCAGGCT
TCGGCCACGATGAACGACAAAAAAGTCCTGATCGACGAGCTGGTAGCTGTTCGTCACTTGAACGAGTTCACCGTCAAGGCGCCGG
AAGACGTCACCTTGATGGACGTATCGCCGAAGCAGGTAGTTTCGGTTGCTGCGTCGCTGATCCCGTTCCTCGAGCACGATGACGC
CAACCGTGCGTTGATGGGTTCGAACATGCAGCGTCAAGCTGTACCAACCCTGCGCGCTGACAAGCCGCTGGTAGGTACCGGCATG
GAGCGTAACGTGGCTCGCGACTCCGGCGTTTGCGTCGTGGCTCGTCGTGGCGGCGTGATCGATTCCGTTGATGCCAGCCGTATCGT
GGTTCGTGTTGCTGATGATGAAGTTGAAACCGGCGAAGCTGGTGTCGATATCTACAACCTGACCAAATACACCCGCTCCAACCAG
AACACCTGCATCAACCAGCGTCCGCTGGTGAGCAAGGGTGATCGGGTTCAGCGTAGCGACATCATGGCTGACGGTCCGTCCACCG
ACATGGGTGAGCTGGCTCTGGGTCAGAACATGCGCATCGCGTTCATGGCATGGAACGGCTTCAACTTCGAAGACTCCATCTGCTT
GTCCGAGCGTGTGGTTCAGGAAGACCGTTTCACCACGATCCACATTCAGGAACTGACCTGTGTGGCGCGTGACACCAAGCTTGGG
CCAGAGGAAATCACTGCAGACATCCCGAACGTGGGTGAAGCTGCACTGAACAAGCTGGACGAAGCCGGTATCGTTTACGTAGGT
GCTGAAGTTGGCGCAGGCGACATTCTGGTTGGTAAGGTCACTCCGAAAGGCGAGACCCAACTGACTCCGGAAGAGAAGCTGTTGC
GTGCAATCTTCGGTGAAAAAGCCAGCGACGTTAAAGACACTTCCCTGCGTGTACCTACCGGTACCAAAGGTACTGTCATCGACGT
ACAGGTCTTCACCCGTGACGGCGTAGAGCGTGATGCTCGTGCTCTGTCGATCGAGAAGACTCAACTCGACGAGATCCGCAAGGAC
CTGAACGAAGAGTTCCGTATCGTTGAAGGCGCTACTTTCGAACGTCTGCGTTCCGCTCTGGTCGGCCATAAAGCCGAAGGCGGCG
CCGGTCTGAAGAAAGGTCAGGAAATCACCGACGAAGTTCTCGACGGTCTTGAGCACGGCCAGTGGTTCAAACTGCGCATGGCTGA
AGATGCTCTGAACGAGCAGCTCGAGAAGGCTCAGGCCTACATCGTTGATCGCCGCCGTCTGCTGGACGACAAGTTCGAAGACAAG
AAGCGCAAACTGCAGCAGGGCGATGACCTGGCTCCAGGCGTGCTGAAAATCGTCAAGGTTTACCTGGCAATCCGTCGCCGCATCC
AGCCGGGCGACAAGATGGCCGGTCGTCACGGTAACAAGGGTGTGGTCTCCGTGATCATGCCGGTTGAAGACATGCCGCACGATG
CCAATGGCACCCCGGTCGACGTTGTCCTCAACCCGTTGGGCGTACCTTCGCGTATGAACGTTGGTCAGATCCTTGAAACCCACCTG
GGCCTCGCGGCCAAAGGTCTGGGCGAGAAGATCAACCGTATGATCGAAGAGCAGCGCAAGGTTGCTGACCTTCGCAAGTTCCTGC
ACGAGATCTACAACGAGATCGGCGGCCGCAACGAAGAGCTGGACACCTTCTCCGACCAGGAAATCCTGGATCTGGCGAAGAACC
TGCGCGGTGGCGTACCGATGGCCACTCCGGTGTTCGACGGTGCCAAGGAAAGCGAAATCAAGGCCATGCTGAAACTGGCAGACC
TGCCGGAAAGCGGCCAGATGCAGCTGACTGACGGCCGTACCGGCAACAAGTTCGAGCGCCCGGTTACCGTTGGCTACATGTACAT
GCTGAAGCTGAACCACTTGGTAGACGACAAGATGCACGCTCGTTCTACCGGTTCGTACAGCCTGGTTACCCAGCAGCCGCTGGGT
GGTAAGGCGCAGTTCGGTGGTCAGCGTTTCGGGGAGATGGAGGTCTGGGCACTGGAAGCATACGGTGCTGCTTACACTCTGCAAG
AAATGCTCACAGTGAAGTCGGACGATGTGAACGGCCGGACCAAGATGTACAAAAACATCGTGGACGGCGATCACCGTATGGAGC
CGGGCATGCCCGAGTCCTTCAACGTGTTGATCAAGGAAATTCGTTCCCTCGGCATCGATATCGATCTGGAAACCGAATAA

P. fluorescens FW300-N2C3_GCF_001307275.1_CP012831.1

ATGGCTTACTCATATACTGAGAAAAAACGTATCCGCAAGGACTTTAGCAAGTTGCCGGACGTCATGGATGTGCCGTACCTCCTGG
CCATCCAGCTGGATTCGTATCGTGAATTCTTGCAAGCGGGAGCGACTAAAGATCAGTTCCGCGACGTGGGCCTGCATGCGGCCTT
CAAATCCGTTTTCCCGATCATCAGCTACTCCGGCAATGCTGCGCTGGAGTACGTCGGTTATCGCCTGGGCGAACCGGCATTTGATG
TCAAAGAATGCGTATTGCGCGGTGTTACTTACGCCGTACCTTTGCGGGTAAAAGTGCGCCTGATCATTTTCGACAAAGAATCGTCG
AACAAAGCGATCAAGGACATCAAAGAGCAAGAAGTCTACATGGGTGAAATCCCCCTGATGACTGAAAACGGTACCTTCGTAATC
AACGGTACCGAGCGTGTAATCGTTTCCCAGCTGCACCGTTCCCCGGGCGTGTTCTTCGACCACGACCGTGGCAAGACGCACAGCT
CCGGCAAACTGCTTTACTCCGCGCGCATCATTCCTTACCGCGGTTCGTGGCTGGACTTCGAGTTCGACCCGAAAGACTGCGTATTC
GTGCGTATCGACCGTCGTCGCAAGCTGCCTGCATCGGTACTGCTGCGCGCGCTCGGCTATACCACCGAAGAAGTGCTGGACGCGT
TCTACACCACCAACGTCTTCCACGTGCAAGGTGAAAACCTCAGTCTGGAACTGGTGCCTCAGCGCCTGCGCGGTGAAATCGCTGT
CCTCGATATCCAGGATGACAAAGGCAAGGTTATTGTCGAGCAGGGCCGTCGTATCACCGCTCGCCACATCAACCAGCTGGAAAAA
GCCGGGATCAAAGAGCTGCAGGTACCGATCGACTACGTCCTGGGTCGCACCACGGCCAAGGTCATCGTGCATCCGGCCACCGGCG
AAATCCTTGCAGAGTGCAATACCGAGCTGACCACCGAGATCCTGGCGAAAATCGCCAAGGCCCAGGTCGTTCGTATCGAAACCCT
GTACACCAACGACATCGACTGCGGTCCGTTCATCTCCGACACGCTGAAGATCGACTCCACCGGCAACCAGCTGGAAGCCTTGGTC
GAGATCTATCGCATGATGCGTCCTGGCGAGCCGCCAACCAAGGATGCAGCCGAGACCCTGTTCAACAACCTGTTCTTCAGTCCTG
AGCGCTATGACCTGTCTGCGGTCGGCCGGATGAAGTTCAACCGTCGTATCGGTCGTACCGAGATCGAAGGTTCGGGCGTGCTGAA
CAAGGATGACATCGTTGCGGTCCTCAAGACCCTGGTCGACATCCGTAACGGCAAAGGCATCGTCGATGACATCGACCACCTGGGT
AACCGTCGTGTTCGCTGTGTAGGCGAGATGGCCGAGAACCAGTTCCGTGTTGGCCTGGTGCGCGTAGAGCGCGCGGTCAAGGAAC
GTCTGTCGATGGCTGAAAGCGAAGGCCTGATGCCGCAAGACCTGATCAACGCCAAGCCTGTGGCTGCGGCGGTGAAGGAGTTCTT
CGGTTCGAGCCAGCTGTCCCAGTTCATGGACCAGAACAACCCGCTGTCCGAGATCACCCACAAGCGTCGTGTCTCCGCACTCGGC
CCAGGCGGTCTGACCCGTGAGCGTGCAGGCTTCGAAGTTCGAGACGTACACCCGACTCACTACGGTCGTGTCTGCCCGATCGAAA
CGCCGGAAGGTCCGAACATCGGTCTGATCAACTCCCTGGCGGCCTATGCGCGCACCAACCAGTACGGCTTCCTCGAGAGCCCGTA
CCGCGTGGTGAAAGACGCCCTGGTTACCGACGAGATCGTGTTCCTGTCCGCCATCGAAGAAGCCGATCACGTGATCGCCCAGGCT
TCGGCCACGATGAACGACAAGAAAGTCCTGGTCGACGAGCTGGTAGCTGTTCGTCACCTGAACGAGTTCACCGTCAAGGCGCCGG
AAGACGTCACCTTGATGGACGTATCGCCGAAGCAGGTAGTGTCGGTTGCAGCGTCGCTGATCCCGTTCCTCGAGCACGACGACGC
CAACCGTGCGTTGATGGGTTCGAACATGCAGCGTCAAGCTGTACCAACGCTGCGTGCCGACAAGCCGCTGGTCGGTACCGGCATG
GAGCGTAACGTTGCCCGTGACTCCGGCGTTTGCGTCGTGGCTCGTCGTGGTGGCGTGATCGATTCCGTCGATGCCAGCCGTATCGT

GGTTCGTGTTGCCGATGACGAAGTTGAAACTGGCGAAGCCGGTGTCGACATCTACAACCTGACCAAATACACCCGCTCCAACCAG
AACACCTGCATCAACCAGCGTCCGCTGGTAAGCAAGGGTGATCGGGTTCAGCGCAGCGACATCATGGCCGATGGTCCGTCCACCG
ATATGGGTGAACTGGCGTTGGGTCAGAACATGCGCATCGCGTTCATGGCATGGAACGGCTTCAACTTCGAAGACTCCATCTGCCT
GTCCGAGCGTGTGGTTCAGGAAGACCGCTTCACCACGATCCACATCCAGGAACTGACCTGTGTGGCCCGTGACACCAAGCTTGGC
CCAGAGGAAATCACTGCGGACATCCCGAACGTGGGTGAAGCCGCACTGAACAAACTGGACGAAGCCGGTATCGTTTATGTAGGT
GCCGAAGTCGGCGCAGGCGACATCCTGGTGGGCAAGGTCACTCCGAAAGGCGAGACCCAGCTGACTCCGGAAGAAAAACTGCTG
CGTGCCATCTTCGGTGAAAAAGCCAGCGACGTTAAAGACACCTCCCTGCGCGTGCCTACCGGCACCAAAGGTACCGTCATCGACG
TACAGGTCTTCACTCGCGACGGCGTGGAGCGTGATGCTCGTGCACTGTCCATCGAGAAGACTCAGCTCGACGAGATCCGCAAGGA
CCTGAACGAAGAGTTCCGTATCGTCGAAGGCGCAACTTTCGAGCGTCTGCGTTCCGCCCTGGTCGGCCACAAAGCCGAAGGCGGC
GCCGGCCTGAAGAAAGGTCAGGAAATCACCGACGAAGTCCTCGACGGTCTTGAGCATGGCCAGTGGTTCAAACTGCGCATGGCT
GAAGATGCTCTGAACGAGCAGCTCGAGAAGGCCCAGGCCTATATCGTTGATCGCCGCCGTCTGCTGGACGACAAGTTCGAAGACA
AGAAGCGCAAACTGCAGCAAGGCGATGACCTGGCTCCAGGCGTGCTGAAAATCGTCAAGGTTTACCTGGCAATCCGTCGTCGCAT
CCAGCCGGGCGACAAGATGGCCGGTCGTCACGGTAACAAGGGTGTGGTCTCTGTGATCATGCCGGTTGAAGACATGCCGCACGAT
GCCAATGGCACCCCGGTCGATGTGGTCCTCAACCCGTTGGGCGTACCTTCGCGTATGAACGTTGGTCAGATCCTTGAAACCCACCT
GGGCCTCGCGGCCAAGGGCTTGGGCGAGAAGATCAACCGGATGATCGAAGAGCAGCGCAAGGTCGCTGACCTGCGTAAGTTCCT
GCACGAGATCTACAACGAAATCGGCGGACGCAACGAAGAGCTGGACACCTTCTCCGACCAGGAAATCCTGGACCTGGCGAAGAA
CCTGCGCGGCGGCGTTCCAATGGCTACTCCGGTGTTCGACGGTGCCAAGGAAAGCGAAATCAAGGCCATGCTGAAACTGGCAGA
CCTGCCGGAAAGTGGCCAGATGCAGCTGTTCGACGGCCGTACCGGCAACAAGTTCGAGCGCCCGGTTACCGTTGGCTACATGTAC
ATGCTGAAGCTGAACCACTTGGTAGACGACAAGATGCACGCTCGTTCTACCGGTTCTTACAGCCTGGTTACCCAGCAGCCGCTGG
GTGGTAAGGCGCAGTTCGGTGGTCAGCGTTTCGGGGAGATGGAGGTCTGGGCACTGGAAGCGTACGGTGCTGCATACACTCTGCA
AGAAATGCTCACAGTGAAGTCGGACGATGTGAACGGCCGGACCAAGATGTACAAAAACATCGTGGACGGCGATCACCGTATGGA
GCCGGGCATGCCCGAGTCTTTCAACGTGTTGATCAAGGAAATTCGTTCCCTCGGCATCGATATCGATCTGGAAACCGAATAA

P. fluorescens 2P24_GCF_002865505.1_CP025542.1

ATGGCTTACTCATATACTGAGAAAAAACGTATCCGCAAGGACTTTAGCAAGTTGCCGGACGTCATGGATGTGCCGTACCTCCTGG
CCATCCAGCTGGATTCGTATCGTGAATTCTTGCAAGCGGGAGCGACTAAAGATCAGTTCCGCGACGTGGGCCTGCATGCGGCCTT
CAAATCCGTTTTCCCGATCATCAGCTACTCCGGCAATGCTGCGCTGGAGTACGTCGGTTATCGCCTGGGCGAACCGGCATTTGATG
TCAAAGAATGCGTATTGCGCGGTGTTACTTACGCCGTACCTTTGCGGGTAAAAGTGCGTCTGATCATTTTCGACAAAGAATCGTCG
AACAAAGCGATCAAGGACATCAAAGAGCAAGAAGTCTACATGGGTGAAATCCCCCTGATGACTGAGAACGGTACCTTCGTAATC
AACGGTACCGAGCGTGTAATCGTTTCCCAGCTGCACCGTTCGCCGGGCGTGTTCTTCGACCACGACCGTGGCAAGACGCACAGCT
CCGGCAAACTGCTGTACTCCGCGCGCATCATTCCTTACCGCGGTTCGTGGTTGGACTTCGAGTTCGACCCGAAAGACTGCGTATTC
GTGCGTATCGACCGTCGTCGCAAGCTGCCTGCATCGGTACTGCTGCGCGCGCTCGGCTATACCACCGAAGAAGTGCTTGACGCGT
TCTACACCACCAACGTCTTCCACGTGCAAGGTGAAAACCTCAGCCTGGAACTGGTGCCTCAGCGCCTGCGCGGTGAAATCGCTGT
CCTCGATATCCAGGATGACAAAGGCAAGGTTATTGTCGAGCAGGGTCGTCGTATCACCGCTCGCCACATCAACCAGCTGGAAAAA
GCCGGGATCAAAGAGCTGCAGGTGCCGATCGACTACGTCCTGGGTCGCACCACGGCCAAGGTCATCGTGCATCCGGCTACCGGCG
AGATCCTGGCAGAGTGCAATACCGAGCTGACCACCGAGATCCTGGCGAAAATCGCCAAGGCCCAGGTCGTTCGTATTGAAACGCT
GTATACCAACGACATCGACTGCGGTCCGTTCATCTCCGACACGCTGAAGATCGACTCCACTGGCAACCAACTGGAAGCCCTGGTC
GAAATCTATCGCATGATGCGTCCTGGCGAGCCGCCAACCAAGGATGCAGCCGAGACCCTGTTCAACAACCTGTTCTTCAGCCCTG
AGCGCTATGACCTGTCTGCGGTCGGCCGGATGAAGTTCAACCGTCGTATCGGCCGTACCGAGATCGAAGGTTCGGGTGTTCTGAA
CAAGGACGACATCGTTGCGGTCCTCAAGACCCTGGTCGACATCCGTAACGGCAAAGGCATCGTCGATGACATCGACCACCTGGGT
AACCGTCGTGTTCGCTGTGTAGGCGAAATGGCCGAGAACCAGTTCCGTGTTGGCCTGGTGCGCGTAGAGCGTGCGGTCAAGGAAC
GCCTGTCGATGGCTGAAAGCGAAGGCCTGATGCCGCAAGACCTGATCAACGCCAAGCCTGTGGCTGCGGCGGTGAAGGAGTTCTT
CGGTTCGAGCCAGCTGTCCCAGTTCATGGGCCAGAACAACCCGCTGTACGAGATCACCCACAAGCGTCGTGTTTCCGCACTGGGC
CCAGGTGGTCTGACCCGTGAGCGTGCAGGCTTCGAGGTTCGTGACGTACACCCGACTCACTACGGTCGTGTATGCCCGATCGAAA
CGCCGGAAGGTCCGAACATCGGTCTGATCAACTCCCTGGCTGCCTATGCGCGCACCAACCAGTACGGTTTCCTCGAGAGCCCGTA
CCGCGTAGTGAAAGACGCCCTGGTCACCGACGAGATCGTGTTCCTGTCCGCCATCGAAGAAGCCGATCACGTGATCGCCCAGGCT
TCGGCCACGATGAACGACAAGAAAGTCCTGACCGATGAATTGGTAGCTGTTCGTCACCTGAACGAGTTCACCGTCAAGGCGCCGG
AAGACGTCACCTTGATGGACGTATCGCCGAAGCAGGTAGTTTCGGTTGCAGCGTCGCTGATCCCGTTCCTCGAGCACGACGACGC
CAACCGTGCGTTGATGGGTTCGAACATGCAGCGTCAAGCTGTACCGACCCTGCGTGCCGACAAGCCGCTGGTAGGTACCGGCATG
GAGCGTAACGTTGCCCGTGACTCCGGCGTTTGCGTCGTGGCTCGTCGTGGTGGCGTGATCGACTCCGTCGACGCCAGCCGTATCGT
GGTTCGTGTTGCCGATGACGAAGTTGAAACTGGCGAAGCCGGTGTCGACATCTACAACCTGACCAAATACACCCGCTCCAACCAG
AACACCTGCATCAACCAGCGTCCGCTGGTAAGCAAGGGTGATCGGGTTCAGCGTAGCGACATCATGGCCGATGGTCCGTCCACCG
ATATGGGTGAACTGGCGTTGGGCCAGAACATGCGCATCGCGTTCATGGCATGGAACGGCTTCAACTTCGAAGACTCCATCTGCCT
GTCCGAGCGTGTGGTCCAGGAAGACCGTTTCACCACGATCCACATCCAGGAACTGACCTGTGTGGCCCGTGACACCAAGCTTGGC
CCAGAGGAAATCACTGCGGACATCCCGAACGTGGGTGAAGCAGCACTGAACAAACTGGACGAAGCCGGTATCGTTTACGTAGGT
GCTGAAGTTGGCGCAGGCGACATCCTGGTGGGCAAGGTCACTCCGAAAGGCGAGACCCAGCTGACTCCGGAAGAAAAACTGCTG
CGTGCCATCTTCGGTGAAAAAGCCAGCGACGTTAAAGACACCTCCCTGCGTGTGCCTACCGGCACCAAGGGTACCGTCATCGACG
TACAGGTCTTCACCCGTGACGGCGTCGAGCGTGATGCTCGTGCACTGTCCATCGAGAAGACTCAACTCGACGAGATCCGCAAGGA
CCTGAACGAAGAGTTCCGTATCGTCGAAGGCGCAACTTTCGAACGTTTGCGCTCCGCTTTGGTCGGCCACAAGGCCGAAGGCGGC
GCCGGCCTGAAGAAAGGTCAGGAAATCACCGACGAAGTACTCGACGGTCTTGAGCATGGCCAGTGGTTCAAACTGCGCATGGCT
GAAGATGCTCTGAACGAGCAGCTCGAGAAGGCCCAGGCCTATATCGTTGATCGCCGCCGTCTGCTGGACGACAAGTTCGAAGACA
AGAAGCGCAAGCTGCAGCAGGGCGATGACCTGGCTCCAGGCGTGCTGAAAATCGTCAAGGTTTACCTGGCAATCCGTCGTCGCAT
CCAGCCGGGCGACAAGATGGCCGGTCGTCACGGTAACAAGGGTGTGGTCTCCGTGATCATGCCGGTTGAAGACATGCCGCACGAT
GCCAATGGCACCCCGGTCGATGTGGTCCTCAACCCGTTGGGCGTACCTTCGCGTATGAACGTTGGCCAGATCCTTGAAACCCACCT

GGGCCTCGCAGCCAAGGGCTTGGGCGAGAAGATCAACCGCATGATCGAAGAGCAACGCAAGGTCGCTGACCTGCGTAAGTTCCT
GCACGAGATCTATAACGAGATCGGCGGCCGCAACGAAGAGCTGGACACCTTCTCCGACCAGGAAATCCTGGATCTGGCGAAGAA
CCTGCGCGGCGGCGTTCCAATGGCTACTCCGGTGTTCGACGGTGCCAAGGAAAGCGAAATCAAGGCCATGCTGAAACTGGCAGA
CCTGCCGGAAAGCGGCCAGATGCAGCTGTTCGACGGCCGTACCGGCAACAAGTTCGAGCGCCCGGTTACCGTTGGCTACATGTAC
ATGCTGAAGCTGAACCACTTGGTGGACGACAAGATGCACGCTCGTTCTACCGGTTCTTACAGCCTGGTTACCCAGCAGCCGCTGG
GTGGTAAGGCGCAGTTCGGTGGTCAGCGTTTCGGGGAGATGGAGGTCTGGGCACTGGAAGCGTACGGTGCTGCATACACTCTGCA
AGAAATGCTCACAGTGAAGTCGGACGATGTGAACGGCCGGACCAAGATGTACAAAAACATCGTGGACGGCGATCACCGTATGGA
GCCGGGCATGCCCGAGTCTTTCAACGTGTTGATCAAGGAAATTCGTTCCCTCGGCATCGATATCGATCTGGAAACCGAATAA

P. fluorescens MS82_GCF_003055645.1_CP028826.1

ATGGCTTACTCATATACTGAGAAAAAACGTATCCGCAAGGACTTTAGCAAGTTGCCGGACGTCATGGATGTGCCTTACCTCCTGGC
CATCCAGCTGGATTCGTATCGTGAATTCTTGCAAGCGGGAGCGACTAAAGATCAGTTCCGCGACGTGGGCCTGCATGCGGCCTTC
AAATCCGTTTTCCCGATCATCAGCTACTCCGGCAATGCTGCGCTGGAGTACGTCGGTTATCGCCTGGGCGAACCGGCATTTGATGT
CAAAGAATGCGTATTGCGCGGTGTAACTTTCGCCGTACCTTTGCGGGTAAAAGTGCGCCTGATCATTTTCGACAAAGAATCGTCGA
ACAAAGCGATCAAGGACATCAAAGAGCAAGAAGTCTACATGGGTGAAATCCCATTGATGACTGAGAACGGTACCTTCGTAATCA
ACGGTACCGAGCGTGTTATCGTTTCCCAGCTGCACCGTTCCCCGGGCGTGTTCTTCGACCACGACCGTGGCAAGACGCACAGCTCC
GGCAAACTGCTGTACTCCGCGCGCATCATTCCTTACCGCGGTTCGTGGCTGGACTTCGAGTTCGACCCGAAAGACTGCGTCTTCGT
GCGTATCGACCGTCGTCGCAAGCTGCCGGCCTCGGTTTTGCTGCGTGCACTCGGTTACACCACCGAAGAAGTGCTGGACGCGTTCT
ACACCACCAACGTATTCCACCTGAGCGGCGAAACCCTCAGCCTGGAACTGGTGCCATCGCGCCTGCGTGGTGAAGTTGCGGTTCT
GGACATCCAGGATGAGAAGGGCAAGGTCATCGTTGAGCAAGGCCGCCGTATTACCGCGCGCCACATCAACCAGATCGAAAAAGC
CGGAATCAAGACGCTGGATGTGCCGCTGGACTACGTCCTCGGTCGTACGACCGCGAAGGCCATCGTGCACCCGGCTACCGGTGAG
ATCCTGGCAGAGTGCAACACCGAGCTGTCGACCGAGATCCTGGCGAAAATCGCCAAGGCCGGCGTCGTACGCATCGAAACTCTGT
ACACCAACGACATCGACTGCGGTCCGTTCGTCTCCGACACGCTGAAGATCGACTCCACCAGCAACCAATTGGAAGCGCTGGTCGA
GATCTATCGCATGATGCGTCCAGGCGAGCCGCCAACCAAGGACGCTGCCGAGACCCTGTTCAACAACCTGTTCTTCAGCCCTGAG
CGTTACGATCTCTCTGCGGTCGGCCGGATGAAGTTCAACCGTCGTATCGGTCGTACCGAGATCGAAGGTTCGGGCGTGCTGTGCA
AGGAAGACATCGTCGCGGTACTGAAGACTCTGGTCGACATCCGTAACGGTAAAGGCATCGTCGATGACATCGACCACTTGGGTAA
CCGTCGTGTTCGCTGCGTAGGCGAAATGGCCGAGAACCAGTTCCGCGTTGGCCTGGTACGTGTTGAGCGTGCGGTCAAAGAGCGC
CTGTCGATGGCTGAAAGCGAAGGCCTGATGCCGCAAGATCTGATCAACGCCAAGCCAGTGGCTGCGGCGGTGAAAGAGTTCTTCG
GTTCCAGCCAGCTGTCGCAGTTCATGGACCAGAACAACCCGCTGTCCGAGATCACCCACAAGCGTCGTGTCTCTGCACTCGGCCCT
GGCGGTCTGACTCGTGAGCGCGCGGGCTTCGAAGTCCGTGACGTACACCCGACTCACTACGGTCGTGTCTGCCCGATTGAAACGC
CGGAAGGTCCGAACATCGGTCTGATCAACTCCCTGGCTGCTTACGCTCGCACCAACCAGTACGGCTTCCTCGAGAGCCCGTACCG
CGTGGTGAAAGAGGGTGTGGTCACCGACGACATCGTGTTCCTGTCCGCCATTGAAGAAGCCGATCACGTGATCGCGCAGGCTTCG
GCGACCATGAACGAGCAGAAAGTCCTGGTCGACGAACTGGTGGCCGTACGTCACCTGAACGAATTCACCGTCAAGGCGCCGGAA
GACGTCACCTTGATGGACGTTTCGCCGAAGCAGGTAGTTTCGGTTGCAGCGTCGCTGATTCCGTTCCTCGAGCACGACGACGCCA
ACCGTGCATTGATGGGTTCGAACATGCAGCGTCAAGCTGTACCAACCCTGCGCGCTGACAAGCCGCTGGTCGGTACCGGCATGGA
GCGTAACGTGGCTCGCGACTCCGGCGTTTGCGTCGTGGCTCGTCGCGGCGGCGTGATCGACTCCGTTGATGCCAGCCGTATCGTGG
TTCGCGTTGCTGATGACGAAGTTGAAACCGGCGAAGCGGGTGTCGACATCTACAACCTGACCAAATACACCCGCTCGAACCAGAA
CACCTGCATCAACCAGCGTCCGTTGGTGAGCAAGGGTGATCGTGTTCAGCGTGGCGACATCATGGCCGACGGTCCGTCCACCGAC
ATGGGTGAACTGGCTCTGGGTCAGAACATGCGCATCGCGTTCATGGCATGGAACGGCTTCAACTTCGAAGACTCCATCTGCCTGTC
CGAGCGTGTGGTTCAGGAAGACCGTTTCACCACGATCCACATTCAGGAACTGACCTGTGTGGCACGTGACACCAAGCTTGGCCCA
GAGGAAATCACTGCGGACATCCCTAACGTGGGTGAAGCTGCGCTGAACAAGCTGGACGAAGCCGGTATCGTTTACGTAGGTGCTG
AAGTTGGCGCAGGCGACATCCTGGTAGGCAAGGTCACTCCGAAAGGCGAGACCCAACTGACTCCGGAAGAAAAACTGCTGCGCG
CAATCTTCGGTGAAAAAGCCAGCGACGTTAAAGACACTTCCCTGCGCGTGCCAACGGGCACCAAAGGTACTGTCATCGACGTACA
GGTCTTCACGCGTGACGGCGTTGAGCGCGATGCTCGTGCGCTGTCGATCGAGAAGTCCCAGCTGGACGAGATCCGCAAGGATCTG
AACGAAGAGTTCCGCATCGTTGAAGGCGCGACCTTCGAGCGTCTGCGTTCCGCTCTGGTAGGCCACAAGGCTGAAGGCGGCGCGG
GCCTGAAGAAAGGTCAGGACATCACCGACGAAGTACTCGACGGTCTTGAGCATGGTCAGTGGTTCAAACTGCGCATGGCTGAAG
ATGCTCTGAACGAGCAGCTCGAGAAGGCTCAGGCCTACATCGTTGATCGTCGCCGTCTGCTGGACGACAAGTTCGAAGACAAGAA
GCGCAAACTGCAGCAGGGCGATGACCTGGCTCCAGGGGTGCTGAAGATCGTCAAGGTTTACCTGGCAATCCGTCGTCGCATCCAG
CCGGGCGACAAGATGGCCGGTCGTCACGGTAACAAAGGTGTGGTCTCCGTGATCATGCCGGTTGAAGACATGCCGCACGATGCCA
ATGGCACCCCGGTCGACGTGGTCCTCAACCCGCTGGGCGTACCTTCGCGTATGAACGTTGGTCAGATCCTTGAAACCCACCTGGG
CCTCGCGGCCAAAGGTCTGGGCGAGAAGATCAACCGGATGGTCGAAGAGCAGCGCAAAGTCGCTGAGCTGCGTACCTTCCTGGA
CGAGATCTACAACCAGATCGGCGGTCGTAACGAAGATCTGGACAGCTTCTCCGATCAGGAAATCCTGGATCTGGCGAAGAACCTG
CGTGGCGGTGTTCCAATGGCCACTCCAGTGTTCGACGGTGCCAAGGAAAGCGAAATCAAGGCCATGCTGAAACTGGCAGACCTGC
CAGAAAGCGGCCAGATGCAGCTGACCGACGGCCGTACCGGCAACAAGTTCGAACGTCCAGTTACCGTTGGCTACATGTACATGCT
GAAGCTGAACCACTTGGTAGACGACAAGATGCACGCGCGTTCTACCGGTTCGTACAGCCTGGTTACCCAGCAGCCGCTGGGTGGT
AAGGCGCAGTTCGGTGGTCAGCGTTTCGGGGAGATGGAGGTCTGGGCACTGGAAGCATACGGTGCTGCTTACACTCTGCAAGAAA
TGCTCACAGTGAAGTCGGACGATGTGAACGGCCGGACCAAGATGTACAAAAACATCGTGGACGGCGATCACCGTATGGAGCCGG
GCATGCCCGAGTCCTTCAACGTGTTGATCAAGGAAATTCGTTCCCTCGGCATCGATATCGATCTGGAAACCGAATAA

P. fluorescens Pf275_GCF_003410335.1_CP031648.1

ATGGCTTACTCATATACTGAGAAAAAACGTATCCGCAAGGACTTTAGCAAGTTGCCGGACGTCATGGATGTGCCGTACCTCCTGG
CCATCCAGCTGGATTCGTATCGTGAATTCTTGCAAGCGGGAGCGACTAAAGATCAGTTCCGCGACGTGGGCCTGCATGCGGCCTT
CAAATCCGTTTTCCCGATCATCAGCTACTCCGGCAATGCTGCGCTGGAGTACGTCGGTTATCGCCTGGGCGAACCGGCATTTGATG
TCAAAGAATGCGTATTGCGCGGTGTTACTTACGCCGTACCTTTGCGGGTAAAAGTGCGCCTGATCATTTTCGACAAAGAATCGTCG

AACAAAGCGATCAAGGACATCAAAGAGCAAGAAGTCTACATGGGTGAAATCCCCCTGATGACTGAGAACGGTACCTTCGTAATC
AACGGTACCGAGCGTGTAATCGTTTCCCAGCTGCACCGTTCTCCGGGCGTGTTCTTCGACCACGACCGTGGCAAGACGCACAGCT
CCGGCAAACTGCTGTACTCCGCGCGCATCATTCCTTACCGCGGTTCGTGGCTGGACTTCGAGTTCGACCCGAAAGACTGCGTATTC
GTGCGTATCGACCGTCGTCGCAAGCTGCCTGCATCGGTACTGCTGCGCGCGCTCGGCTATACCACCGAAGAAGTGCTGGACGCGT
TCTACACCACCAACGTCTTCCACGTGCAAGGTGAAAACCTCAGCCTGGAACTGGTGCCTCAGCGCCTGCGCGGTGAAATCGCTGT
CCTCGATATCCAGGATGACAAAGGCAAGGTTATTGTCGAGCAGGGTCGCCGTATCACCGCTCGCCACATCAACCAGCTGGAAAAA
GCCGGGATCAAAGAGCTGCAGGTGCCGATCGACTACGTCCTGGGTCGCACCACGGCCAAGGTCATCGTGCATCCGGCCACTGGCG
AAATCCTGGCAGAGTGCAATACCGAGCTGACCACCGAGATCCTGGCGAAAATCGCCAAGGCCCAGGTCGTTCGTATCGAAACGTT
GTACACCAACGACATCGACTGCGGTCCGTTCATCTCCGACACGCTGAAAATCGACTCCACCGGCAACCAGCTGGAAGCCCTGGTC
GAAATCTATCGCATGATGCGTCCTGGCGAGCCGCCAACCAAGGATGCAGCCGAGACCCTTTTCAACAACCTGTTCTTCAGCCCTG
AGCGCTATGACCTGTCTGCGGTCGGCCGGATGAAGTTCAACCGTCGTATCGGTCGTACCGAGATCGAAGGTTCGGGTGTTCTGAA
CAAGGACGACATCGTTGCGGTCCTCAAGACCCTGGTCGACATCCGTAACGGCAAAGGCATCGTCGATGACATCGATCACCTGGGT
AACCGTCGTGTTCGCTGTGTAGGCGAAATGGCCGAGAACCAGTTCCGTGTTGGCCTGGTGCGCGTAGAGCGTGCGGTCAAGGAGC
GCCTGTCGATGGCTGAAAGCGAAGGCCTGATGCCGCAAGACCTGATCAACGCCAAGCCTGTGGCTGCGGCGGTGAAAGAGTTCTT
CGGTTCGAGCCAGCTGTCCCAGTTCATGGACCAGAACAACCCGCTGTCCGAGATCACCCACAAGCGTCGTGTTTCCGCACTGGGC
CCAGGCGGTCTGACCCGTGAGCGTGCAGGCTTCGAAGTTCGTGACGTGCACCCGACGCACTACGGTCGTGTATGCCCGATCGAAA
CGCCGGAAGGTCCGAACATCGGTCTGATCAACTCCCTGGCTGCCTATGCGCGCACCAACCAGTACGGTTTCCTCGAGAGCCCGTA
TCGTGTGGTGAAAGACGCCCTGGTCACCGACGAGATCGTGTTCCTGTCCGCCATCGAAGAAGCCGATCACGTGATCGCCCAGGCT
TCGGCCACGATGAACGACAAGAAAGTCCTGACCGATGAGTTGGTAGCTGTTCGTCACCTGAACGAGTTCACCGTCAAGGCGCCGG
AAGACGTCACCTTGATGGACGTATCGCCGAAGCAGGTAGTTTCGGTTGCAGCGTCGCTGATCCCGTTCCTCGAGCACGACGACGC
CAACCGTGCGTTGATGGGTTCGAACATGCAGCGTCAAGCTGTACCGACCCTGCGTGCCGACAAGCCGCTGGTAGGTACCGGCATG
GAGCGTAACGTTGCCCGTGACTCCGGCGTTTGCGTCGTGGCTCGTCGTGGTGGCGTGATCGACTCCGTCGACGCCAGCCGTATCGT
GGTTCGTGTTGCCGATGACGAAGTTGAAACTGGCGAAGCCGGTGTCGACATCTACAACCTGACCAAATATACCCGCTCCAACCAG
AACACCTGCATCAACCAGCGTCCGCTGGTAAGCAAGGGTGATCGGGTGCAGCGTAGCGACATCATGGCCGACGGTCCGTCCACCG
ATATGGGTGAACTGGCGTTGGGTCAGAACATGCGCATCGCGTTCATGGCATGGAACGGCTTCAACTTCGAAGACTCCATCTGCCT
GTCCGAGCGTGTGGTCCAGGAAGACCGTTTCACCACGATCCACATCCAGGAACTGACCTGTGTGGCCCGTGACACCAAGCTTGGC
CCAGAGGAAATCACTGCGGACATCCCGAACGTGGGTGAAGCTGCACTGAACAAACTGGACGAAGCCGGTATCGTTTACGTAGGT
GCTGAAGTAGGCGCAGGCGACATCCTGGTGGGCAAGGTCACTCCGAAAGGCGAGACCCAGCTGACTCCGGAAGAAAAACTGCTG
CGTGCCATCTTCGGTGAAAAAGCCAGCGACGTTAAAGACACCTCCCTGCGCGTGCCTACTGGCACCAAGGGTACCGTCATCGACG
TACAGGTCTTCACCCGTGACGGCGTCGAGCGTGATGCTCGTGCACTGTCGATCGAGAAGACTCAACTCGACGAGATCCGCAAGGA
CCTGAACGAAGAGTTCCGTATCGTCGAAGGCGCAACTTTCGAGCGTCTGCGTTCCGCCCTGGTCGGCCACAAAGCCGAAGGCGGC
GCCGGCCTGAAGAAAGGACAGGAAATCACCGACGAAGTCCTCGACGGTCTTGAGCATGGCCAGTGGTTCAAACTGCGCATGGCT
GAAGATGCTCTGAACGAGCAGCTCGAGAAGGCCCAGGCCTATATCGTTGATCGCCGCCGTCTGCTGGACGACAAGTTCGAAGACA
AGAAGCGCAAACTGCAGCAGGGCGATGACCTGGCTCCAGGCGTGCTGAAAATCGTCAAGGTTTACCTGGCAATCCGTCGTCGCAT
CCAGCCGGGCGACAAGATGGCCGGTCGTCACGGTAACAAGGGTGTGGTCTCTGTGATCATGCCGGTTGAAGACATGCCGCACGAT
GCCAATGGCACCCCGGTCGACGTGGTCCTCAACCCATTGGGCGTACCTTCGCGTATGAACGTCGGTCAGATCCTTGAAACCCACCT
GGGCCTCGCGGCCAAGGGCTTGGGCGAGAAGATCAACCGTATGATCGAAGAGCAGCGCAAGGTTGCTGACCTGCGTAAGTTCCT
GCACGAGATCTACAACGAGATCGGCGGCCGCAACGAAGAGCTGGACACCTTCTCCGACCAGGAAATCCTGGATCTGGCGAAGAA
CCTGCGCGGTGGCGTTCCAATGGCTACTCCGGTGTTCGACGGTGCCAAGGAAAGCGAAATCAAGGCCATGCTGAAACTGGCAGAC
CTGCCGGAAAGCGGCCAGATGCAGCTGTTCGACGGCCGTACCGGCAACAAGTTCGAGCGCCCGGTTACCGTTGGCTACATGTACA
TGCTGAAGCTGAACCACTTGGTGGACGACAAGATGCACGCTCGTTCTACCGGTTCTTACAGCCTGGTTACCCAGCAACCGCTGGG
TGGTAAGGCGCAGTTCGGTGGTCAGCGTTTCGGGGAGATGGAGGTCTGGGCACTGGAAGCGTACGGTGCTGCATACACTCTGCAA
GAAATGCTCACAGTGAAGTCGGACGATGTGAACGGCCGGACCAAGATGTACAAAAACATCGTGGACGGCGATCACCGTATGGAG
CCGGGCATGCCCGAGTCTTTCAACGTGTTGATCAAGGAAATTCGTTCCCTCGGCATCGATATCGATCTGGAAACCGAATAA

P. fluorescens SIK_W1_GCF_003612935.1_CP031450.1

ATGGCTTACTCATATACTGAGAAAAAACGTATCCGCAAGGACTTTAGCAAGTTGCCGGACGTCATGGATGTCCCGTACCTTCTGGC
TATCCAGCTGGATTCGTATCGTGAATTCTTGCAGGCGGGAGCGACCAAAGATCAGTTCCGCGACGTGGGCCTGCATGCGGCCTTC
AAATCCGTTTTCCCGATCATCAGCTACTCCGGCAATGCTGCGCTGGAGTACGTCGGTTATCGCCTGGGCGAACCGGCATTTGATGT
CAAAGAATGCGTGTTGCGCGGTGTTACGTACGCCGTACCTTTGCGGGTAAAAGTCCGCCTGATCATTTTCGACAAAGAATCGTCG
AACAAAGCGATCAAGGACATCAAAGAGCAAGAAGTCTACATGGGCGAAATCCCATTGATGACTGAGAACGGTACCTTCGTTATC
AACGGTACCGAGCGTGTTATCGTTTCCCAGCTGCACCGTTCCCCGGGCGTGTTCTTTGACCACGACCGCGGCAAGACGCACAGCTC
CGGCAAGCTCCTGTACTCCGCGCGGATCATTCCGTACCGTGGTTCTTGGTTGGACTTCGAGTTCGACCCTAAAGACTGCGTGTTCG
TGCGTATCGACCGTCGTCGCAAGCTGCCGGCCTCGGTACTGCTGCGCGCGCTCGGCTATACCACTGAGCAAGTGCTGGACGCCTTC
TACACCACCAACGTATTCAGCCTGAAGGATGAAACCCTCAAGCTGGAGCTGATCGCTTCGCGTCTGCGTGGTGAAATTGCCGTCC
TGGACATCCAGGATGAAAAAGGCAAGGTCATTGTTGAAGCTGGCCGTCGTATCACTGCGCGCCACATCAACCAGATCGAAAAAG
CCGGTATCAAAGAGCTGGAAGTGCCTCTGGACTACGTCCTGGGTCGCACTACCGCCAAGGTCATCGTTCACCCGGCTACAGGCGA
AATCCTGGCTGAGTGCAACACCGAGCTGAACACCGAGATCCTGGCCAAAATCGCCAAGGCTCAGGTTGTTCGCATCGAGACCCTG
TACACCAACGACATCGACTGCGGTCCGTTCATCTCCGACACTCTGAAGATCGACTCCACCAGCAACCAATTGGAAGCGCTGGTCG
AGATCTATCGCATGATGCGTCCTGGCGAGCCACCGACCAAAGACGCTGCCGAGACCCTGTTCAACAACCTGTTCTTCAGCCCTGA
GCGCTATGACCTGTCTGCGGTCGGCCGGATGAAGTTCAACCGTCGTATCGGTCGTACCGAGATCGAAGGTTCGGGCGTGTTGTGC
AAGGAAGACATCGTCGCGGTACTGAAGACTCTGGTCGACATCCGTAACGGTAAAGGCATCGTCGATGACATCGACCACCTGGGTA
ACCGTCGTGTTCGCTGCGTAGGCGAAATGGCCGAGAACCAGTTCCGCGTTGGCCTGGTGCGTGTTGAGCGTGCGGTCAAAGAGCG
TCTGTCGATGGCTGAAAGCGAAGGCCTGATGCCACAAGACCTGATCAACGCCAAGCCAGTGGCTGCGGCGGTGAAAGAGTTCTTC

GGCTCCAGCCAGCTCTCGCAGTTCATGGACCAGAACAACCCGCTCTCCGAGATCACCCACAAGCGCCGTGTTTCGGCACTGGGCC
CAGGCGGTCTGACCCGTGAGCGTGCAGGCTTTGAAGTTCGTGACGTACACCCGACGCACTACGGTCGTGTTTGCCCGATCGAAAC
GCCGGAAGGTCCGAACATCGGTCTGATCAACTCCCTGGCTGCGTATGCGCGCACCAACCAGTACGGCTTCCTCGAGAGCCCGTAC
CGCGTGGTGAAAGACGCTCTGGTCACCGACGAGATCGTGTTCCTGTCCGCCATCGAAGAAGCGGATCACGTGATCGCCCAGGCCT
CGGCCACGATGAACGACAAGAAAGTCCTGGTCGACGAGCTGGTAGCGGTTCGTCACTTGAACGAGTTCACCGTCAAGGCGCCGG
AAGACGTCACCTTGATGGACGTTTCGCCGAAGCAGGTAGTTTCGGTTGCAGCGTCGCTGATCCCGTTCCTCGAGCACGACGACGC
CAACCGTGCGTTGATGGGTTCCAACATGCAGCGTCAAGCTGTACCGACCCTGCGCGCTGACAAGCCGCTGGTAGGTACCGGCATG
GAGCGTAACGTAGCCCGTGACTCCGGCGTTTGCGTCGTGGCTCGTCGTGGCGGCGTGATCGACTCCGTTGATGCCAGCCGTATCGT
GGTCCGTGTTGCCGATGACGAAGTTGAAACTGGCGAAGCCGGTGTCGACATCTACAACCTGACCAAATACACCCGCTCGAACCAG
AACACCTGCATCAACCAGCGTCCGCTGGTGAGCAAGGGTGATCGCGTTCAGCGTAGCGACATCATGGCCGACGGCCCGTCCACCG
ACATGGGTGAACTGGCACTGGGCCAGAACATGCGCATCGCGTTCATGGCATGGAACGGCTTCAACTTCGAAGACTCCATCTGCCT
GTCCGAGCGTGTTGTTCAAGAAGACCGTTTCACCACGATCCACATTCAGGAACTGACCTGTGTGGCACGTGACACCAAGCTTGGG
CCAGAGGAAATCACTGCAGACATCCCGAACGTGGGTGAAGCTGCACTGAACAAGCTGGACGAAGCCGGTATCGTTTATGTAGGT
GCTGAAGTTGGCGCAGGCGACATCCTGGTAGGTAAGGTCACTCCGAAAGGCGAGACCCAACTGACTCCAGAGGAAAAACTGCTG
CGTGCGATCTTCGGTGAAAAAGCCAGCGACGTTAAAGACACCTCCCTGCGCGTACCTACCGGTACCAAGGGTACTGTCATCGACG
TACAGGTCTTCACCCGTGACGGCGTTGAGCGTGATGCTCGTGCACTGTCCATCGAGAAGACCCAGCTCGACGAGATCCGCAAGGA
TCTGAACGAAGAGTTCCGTATCGTTGAAGGCGCGACCTTCGAACGTCTGCGTTCCGCCCTGGTAGGCCACAAGGCTGAAGGCGGC
GCAGGTCTGAAGAAAGGTCAGGACATCACCGACGAAGTTCTCGACGGTCTTGAGCACGGCCAGTGGTTCAAACTGCGCATGGCTG
AAGATGCTCTGAACGAGCAGCTCGAGAAGGCCCAGGCCTATATCGTTGATCGTCGCCGTCTGCTGGACGACAAGTTCGAAGACAA
GAAGCGCAAACTGCAGCAAGGCGATGACCTGGCTCCAGGCGTGCTGAAAATCGTCAAGGTTTACCTGGCAATCCGTCGTCGCATC
CAACCGGGCGACAAGATGGCCGGTCGTCACGGTAACAAAGGTGTGGTCTCCGTGATCATGCCGGTTGAAGACATGCCGCACGAT
GCCAATGGCACACCGGTCGACGTCGTCCTCAACCCGTTGGGCGTACCTTCGCGTATGAACGTTGGTCAGATCCTTGAAACCCACCT
GGGCCTCGCGGCCAAAGGTCTGGGCGAGAAGATCAACCGTATGATCGAAGAGCAGCGCAAGGTCGCAGACCTGCGTAAGTTCCT
GCACGAGATCTACAACGAGATCGGCGGTCGCAACGAAGAGCTGGATACCTTCTCCGACCAGGAAATCCTGGATCTGGCGAAGAA
CCTGCGCGGCGGCGTTCCAATGGCTACCCCGGTGTTCGACGGTGCCAAGGAAAGCGAAATCAAGGCCATGCTGAAACTGGCAGA
CCTGCCAGAAAGCGGCCAGATGCAGCTGTTCGACGGCCGTACCGGCAACAAGTTCGAGCGCCCGGTTACTGTTGGCTACATGTAC
ATGCTGAAGCTGAACCACTTGGTAGACGACAAGATGCACGCTCGTTCTACCGGTTCGTACAGCCTCGTTACCCAGCAGCCGCTGG
GTGGTAAGGCTCAGTTCGGTGGTCAGCGTTTCGGGGAGATGGAGGTCTGGGCACTGGAAGCATACGGTGCTGCTTACACTCTGCA
AGAAATGCTCACAGTGAAGTCGGACGATGTGAACGGTCGGACCAAGATGTACAAAAACATCGTGGACGGCGATCACCGTATGGA
GCCGGGCATGCCCGAGTCCTTCAACGTGTTGATCAAAGAAATTCGTTCCCTCGGCATCGATATCGATCTGGAAACCGAATAA

P. fluorescens PF08_GCF_003626995.1_CP032618.1

ATGGCTTACTCATATACTGAGAAAAAACGTATCCGCAAGGACTTTAGCAAGTTGCCGGACGTCATGGATGTCCCGTACCTTCTGGC
TATCCAGCTGGATTCGTATCGTGAATTCTTGCAGGCGGGAGCGACCAAAGATCAGTTCCGCGACGTGGGCCTGCATGCGGCCTTC
AAATCCGTTTTCCCGATCATCAGCTACTCCGGCAATGCTGCGCTGGAGTACGTCGGTTATCGCCTGGGCGAACCGGCATTTGATGT
CAAAGAATGCGTGTTGCGCGGTGTTACGTACGCCGTACCTTTGCGGGTAAAAGTTCGTCTGATCATTTTCGACAAAGAATCGTCGA
ACAAAGCGATCAAGGACATCAAAGAGCAAGAAGTCTACATGGGCGAAATCCCATTGATGACTGAGAACGGTACCTTCGTTATCA
ACGGTACCGAGCGTGTAATCGTTTCCCAGCTGCACCGTTCCCCGGGCGTGTTCTTCGACCACGACCGCGGCAAGACGCACAGCTC
CGGTAAGCTCCTGTACTCCGCGCGGATCATTCCGTACCGTGGTTCGTGGTTGGACTTCGAGTTCGACCCTAAAGACTGCGTGTTCG
TGCGTATCGACCGTCGTCGCAAGCTGCCGGCCTCGGTACTGCTGCGCGCGCTCGGCTATACCACTGAGCAAGTGCTGGACGCTTTC
TACACCACCAACGTATTCAGCCTGAAGGATGAAACCCTCAGCCTGGAACTGATTGCTTCGCGTCTGCGTGGTGAAATTGCCGTCCT
GGACATCCAGGATGAGAAGGGCAAGGTCATTGTTGAGGCTGGCCGTCGTATTACTGCGCGCCACATCAACCAGATCGAAAAAGC
CGGTATCAAGTCGCTGGACGTGCCTCTGGACTACGTCCTGGGCCGCACTACCGCCAAGGTTATCGTTCACCCGGCGACAGGCGAG
ATCCTGGCTGAGTGCAACACCGAGCTGAACACCGAGATCCTGGCAAAAATCGCCAAGGCCCAGGTTGTTCGCATCGAGACCCTGT
ACACCAACGATATCGACTGCGGTCCGTTCGTCTCCGACACCCTGAAGATCGACTCCACCAGCAACCAATTGGAAGCGCTGGTCGA
AATCTATCGCATGATGCGTCCAGGCGAGCCACCAACCAAAGACGCTGCCGAAACCCTGTTCAACAACCTGTTCTTCAGCCCTGAG
CGCTATGACCTGTCTGCGGTCGGCCGGATGAAGTTCAACCGTCGTATCGGTCGTACCGAGATCGAAGGTTCGGGCGTGCTGTGCA
AGGAAGACATCGTTGCGGTCCTGAAGACTCTGGTCGACATCCGTAACGGTAAAGGCATCGTCGATGACATCGACCACCTGGGTAA
CCGTCGTGTTCGCTGCGTAGGCGAAATGGCCGAGAACCAGTTCCGCGTTGGCCTGGTACGTGTTGAGCGTGCGGTCAAAGAGCGT
CTGTCGATGGCCGAAAGCGAAGGCCTGATGCCGCAAGACCTGATCAACGCCAAGCCAGTGGCTGCGGCGGTGAAAGAGTTCTTC
GGCTCCAGCCAGCTTTCGCAGTTCATGGACCAGAACAACCCGCTCTCCGAGATCACCCACAAGCGCCGTGTTTCTGCACTGGGCC
CGGGCGGTCTGACCCGTGAGCGTGCTGGCTTTGAAGTTCGTGACGTACACCCGACGCACTACGGTCGTGTTTGCCCGATCGAAAC
GCCGGAAGGTCCGAACATCGGTCTGATCAACTCCCTGGCTGCCTATGCGCGCACCAACCAGTACGGCTTCCTCGAGAGCCCGTAC
CGCGTGGTGAAAGACGCCCTGGTCACCGACGAGATCGTCTTCCTGTCCGCCATCGAAGAAGCTGATCACGTGATCGCTCAGGCTT
CGGCCACGATGAACGACAAGAAAGTCCTGATCGACGAGCTGGTAGCTGTTCGTCACTTGAACGAGTTCACCGTCAAGGCGCCGGA
AGACGTCACCTTGATGGACGTATCGCCCAAGCAGGTAGTTTCGGTTGCAGCATCGCTGATCCCGTTCCTGGAACACGATGACGCC
AACCGTGCGTTGATGGGTTCCAACATGCAGCGTCAAGCTGTACCGACCCTGCGCGCTGACAAGCCGCTGGTAGGTACCGGCATGG
AGCGTAACGTAGCCCGTGACTCCGGCGTTTGCGTCGTGGCTCGTCGTGGCGGCGTGATCGACTCTGTTGATGCCAGCCGTATCGTG
GTTCGTGTTGCCGATGACGAAGTTGAAACTGGCGAAGCCGGTGTCGACATCTACAACCTGACCAAATACACCCGCTCGAACCAGA
ACACCTGCATCAACCAGCGTCCGCTGGTGCGTAAGGGTGATCGCGTTCAGCGTAGCGACATCATGGCCGACGGTCCGTCCACCGA
TATGGGTGAGTTGGCTCTGGGTCAGAACATGCGCATCGCGTTCATGGCGTGGAACGGCTTCAACTTCGAAGACTCCATCTGCCTGT
CCGAGCGTGTTGTTCAAGAAGACCGTTTCACCACGATCCACATCCAGGAACTGACCTGTGTGGCACGTGACACCAAGCTTGGGCC
AGAGGAAATCACTGCAGACATCCCGAACGTGGGTGAAGCTGCGCTGAACAAGCTGGACGAAGCCGGTATCGTTTACGTAGGTGC
TGAAGTTGGCGCTGGCGACATCCTGGTCGGTAAGGTCACTCCGAAAGGCGAGACCCAACTGACTCCGGAAGAGAAGCTGCTGCG

TGCCATCTTCGGCGAAAAAGCCAGCGACGTTAAAGACACCTCCCTGCGCGTACCTACTGGTACCAAGGGTACTGTCATCGACGTA
CAGGTCTTCACCCGTGACGGCGTCGAGCGTGATGCTCGTGCACTGTCCATCGAGAAGACTCAACTCGACGAAATCCGCAAGGATT
TGAACGAAGAGTTCCGTATCGTTGAAGGCGCGACCTTCGAACGTCTGCGTTCTGCCCTGGTAGGCCACAAGGCTGAAGGCGGCGC
AGGTCTGAAGAAAGGTCAGGATATCACCGACGAAATCCTCGACGGTCTTGAGCACGGCCAGTGGTTCAAACTGCGCATGGCTGAA
GATGCTCTGAACGAGCAGCTCGAGAAGGCCCAGGCCTACATCGTTGATCGCCGCCGTCTGCTGGACGACAAGTTCGAAGACAAG
AAGCGCAAACTGCAGCAGGGCGATGACCTGGCTCCAGGCGTGCTGAAAATCGTCAAGGTTTACCTGGCAATCCGTCGTCGCATTC
AGCCGGGCGACAAGATGGCCGGTCGTCACGGTAACAAAGGTGTGGTCTCCGTGATCATGCCGGTTGAAGACATGCCGCACGATG
CCAATGGCACCCCGGTCGACGTCGTCCTCAACCCGTTGGGCGTACCTTCGCGTATGAACGTTGGTCAGATCCTTGAAACCCACCTG
GGCCTCGCGGCTAAAGGTCTGGGCGAGAAGATCAACCGTATGATCGAAGAGCAGCGCAAGGTCGCAGACCTGCGTAAGTTCCTG
CACGAGATCTACAACGAGATCGGCGGCCGCAAAGAAGAGCTGGACACTTTCTCCGACCAGGAAATCCTGGATCTGGCGAAAAAC
CTGCGCGGCGGCGTTCCAATGGCTACCCCGGTGTTCGACGGTGCCAAGGAAAGCGAAATCAAGGCCATGCTGAAACTGGCAGAC
CTGCCAGAAAGCGGCCAGATGCAGCTGTTCGACGGCCGTACCGGCAACAAGTTCGAGCGCCCGGTTACTGTTGGCTACATGTACA
TGCTGAAGCTGAACCACTTGGTAGACGACAAGATGCACGCTCGTTCTACCGGTTCGTACAGCCTGGTTACCCAGCAGCCGCTGGG
TGGTAAGGCTCAGTTCGGTGGTCAGCGTTTCGGGGAGATGGAGGTCTGGGCACTGGAAGCATACGGTGCTGCATACACTCTGCAA
GAAATGCTCACAGTGAAGTCGGACGATGTGAACGGTCGTACCAAGATGTACAAAAACATCGTGGACGGCGATCACCGTATGGAG
CCGGGCATGCCCGAGTCTTTCAACGTGTTGATCAAAGAAATTCGTTCCCTCGGCATCGATATCGATCTGGAAACCGAATAA

P. fluorescens LBUM677_GCF_004683905.1_CP038438.1

ATGGCTTACTCATATACTGAGAAAAAACGTATCCGCAAGGACTTTAGCAAGTTGCCGGACGTCATGGATGTGCCGTACCTCCTGG
CCATCCAGCTGGATTCGTATCGTGAATTCTTGCAAGCGGGAGCGACTAAAGATCAGTTCCGCGACGTGGGCCTGCATGCGGCCTT
CAAATCCGTTTTCCCGATCATCAGCTACTCCGGCAATGCTGCGCTGGAGTACGTCGGTTATCGCCTGGGCGAACCGGCATTTGATG
TCAAAGAATGCGTATTGCGCGGTGTAACTTTCGCCGTACCTTTGCGGGTAAAAGTGCGCCTGATCATTTTCGACAAAGAGTCGTCG
AACAAAGCGATCAAGGACATCAAAGAGCAAGAAGTCTACATGGGCGAAATCCCATTGATGACTGAGAACGGTACCTTCGTAATC
AACGGTACCGAGCGTGTTATCGTTTCCCAGCTGCACCGTTCCCCGGGCGTGTTCTTTGACCACGACCGCGGCAAGACGCACAGTTC
CGGTAAACTGCTGTACTCCGCGCGCATCATTCCTTACCGCGGTTCGTGGCTGGACTTCGAGTTCGACCCGAAAGACTGCGTGTTCG
TGCGTATCGACCGTCGTCGCAAGCTGCCGGCCTCGGTGCTGCTGCGCGCGCTCGGCTACACCACCGAAGAAGTGCTGGACGCTTT
CTACACCACCAACGTATTCCACGTTAAAGGCGAAGGCCTGAGCCTGGAACTGGTGCCTCAGCGTCTGCGCGGTGAAGTGGCTGTC
CTCGATATCCAGGACGATAAAGGCAAGGTTATTGTCGAGCAGGGCCGTCGTATTACTGCCCGCCACATCAACCAGTTGGAAAAGG
CCGGGATCAAAGAGCTGGACGTGCCTCTGGACTACGTCCTGGGTCGCACCACCGCCAAGGTCATCGTGCATCCGGCTACCGGCGA
AATCCTGGCAGAGTGCAACACCGAGCTGTCGACCGAAGTCCTGGCGAAAATCGCCAAGGCCGGCGTTGTTCGCATCGAAACTCTG
TACACCAACGACATCGACTGCGGTCCGTTCATCTCCGACACGCTGAAGATCGACTCCACCAGCAACCAATTGGAAGCGCTGGTCG
AGATCTATCGCATGATGCGTCCAGGCGAGCCGCCAACCAAAGACGCTGCCGAAACCCTGTTCAACAACCTGTTCTTCAGCCCTGA
GCGCTATGACCTGTCTGCGGTCGGCCGGATGAAGTTCAACCGTCGTATCGGTCGTACCGAGATCGAAGGTTCGGGCGTGCTGTGC
AAGGAAGACATCGTCGCGGTACTGAAGACTCTGGTCGACATCCGTAACGGTAAAGGCATCGTCGATGACATCGACCACCTGGGTA
ACCGTCGTGTTCGCTGCGTAGGCGAAATGGCCGAGAACCAGTTCCGCGTTGGCCTGGTACGTGTTGAGCGTGCGGTCAAAGAGCG
TCTGTCGATGGCTGAAAGCGAAGGCCTGATGCCGCAAGACCTGATCAACGCCAAGCCAGTGGCTGCGGCGGTGAAAGAGTTCTTC
GGTTCCAGCCAGCTCTCGCAGTTCATGGACCAGAACAACCCGCTGTCCGAGATCACCCACAAGCGTCGTGTCTCTGCACTCGGCC
CTGGCGGTCTGACTCGTGAGCGCGCAGGCTTTGAAGTTCGTGACGTACACCCGACTCACTACGGTCGTGTATGCCCGATTGAAAC
GCCGGAAGGTCCGAACATCGGTCTGATCAACTCCTTGGCTGCTTATGCCCGCACCAACCAGTACGGCTTCCTGGAAAGCCCGTAC
CGCGTGGTGAAAGAGGGTGTGGTCACTGACGAGATCGTGTTCCTGTCCGCCATTGAAGAAGCCGATCACGTGATCGCGCAGGCTT
CGGCGACCATGAACGACCAGAAAGTCCTGATCGACGAGCTGGTAGCCGTACGTCACCTGAACGAATTCACCGTCAAGGCGCCGG
AAGACGTCACCCTGATGGACGTTTCGCCGAAGCAGGTAGTTTCGGTTGCAGCGTCGCTGATTCCGTTCCTCGAGCACGACGACGC
CAACCGTGCGTTGATGGGTTCGAACATGCAGCGTCAAGCTGTACCAACCCTGCGTGCTGACAAGCCGCTGGTAGGTACCGGCATG
GAGCGTAACGTGGCTCGCGACTCCGGCGTTTGCGTCGTGGCTCGTCGTGGCGGCGTGATCGACTCTGTTGATGCCAGCCGTATCGT
GGTTCGTGTTGCCGATGACGAAGTTGAAACCGGTGAAGCTGGTGTCGACATCTACAACCTGACCAAATACACCCGCTCGAACCAG
AACACCTGCATCAACCAGCGTCCGCTGGTGAGAAAGGGTGATCGCGTTCAGCGTAGCGACATCATGGCCGACGGCCCGTCCACCG
ACATGGGTGAACTGGCACTGGGTCAGAACATGCGCATCGCGTTCATGGCATGGAACGGCTTCAACTTCGAAGACTCCATCTGCCT
GTCCGAGCGTGTGGTTCAGGAAGATCGCTTCACCACGATCCACATTCAGGAACTGACCTGTGTGGCCCGTGACACCAAGCTTGGC
CCAGAGGAAATCACTGCAGACATCCCGAACGTGGGTGAAGCTGCACTGAACAAGCTGGACGAAGCCGGTATCGTTTACGTAGGT
GCTGAAGTCGGCGCAGGCGACATCCTGGTGGGTAAGGTCACTCCGAAAGGCGAGACCCAACTGACTCCGGAAGAAAAACTGCTG
CGTGCGATCTTCGGTGAAAAAGCCAGCGACGTTAAAGACACCTCCCTGCGTGTACCTACAGGTACCAAAGGTACTGTCATCGACG
TACAGGTCTTCACCCGCGACGGCGTTGAGCGTGATGCTCGTGCACTGTCGATCGAGAAGTCCCAGCTGGACGAGATCCGCAAGGA
TCTGAACGAAGAGTTCCGTATCGTTGAAGGCGCGACCTTCGAACGTCTGCGCTCCGCTCTGGTAGGCCACAAGGCTGAAGGCGGC
GCCGGTCTGAAGAAAGGTCAGGACATCACCGACGAAGTTCTCGACGGTCTTGAGCATGGTCAGTGGTTCAAACTGCGCATGGCTG
AAGATGCTCTGAACGAGCAGCTCGAGAAGGCTCAGGCCTACATCGTTGATCGCCGCCGTCTGCTGGACGACAAGTTCGAAGACAA
GAAGCGCAAACTGCAGCAGGGCGATGACCTGGCTCCAGGCGTGCTGAAAATCGTCAAGGTTTACCTGGCAATCCGTCGTCGCATC
CAGCCGGGCGACAAGATGGCCGGTCGTCACGGTAACAAAGGTGTGGTCTCCGTGATCATGCCGGTTGAAGACATGCCGCACGAT
GCCAATGGCACCCCGGTCGACGTCGTCCTCAACCCGTTGGGCGTACCTTCGCGTATGAACGTTGGTCAGATCCTTGAAACCCACCT
GGGCCTCGCGGCCAAAGGTCTGGGCGAGAAGATCAACCGGATGGTCGAAGAGCAGCGTAAAGTCGCTGAACTGCGTACCTTCCT
CGACGAGATCTACAACCAGATCGGCGGTCGTAACGAAGATCTGGATAGCTTCTCCGATCAGGAAATCCTGGATCTGGCGAAGAAC
CTGCGTGGCGGCGTTCCAATGGCCACTCCAGTGTTCGACGGTGCCAAGGAAAGCGAAATCAAGGCCATGCTGAAACTGGCAGAC
CTGCCGGAAAGCGGCCAGATGCAGCTGACTGACGGCCGTACCGGCAACAAGTTCGAGCGTCCAGTTACCGTTGGCTACATGTACA
TGCTGAAGCTGAACCACTTGGTAGACGACAAGATGCACGCGCGTTCTACCGGTTCGTACAGCCTGGTTACCCAGCAGCCGCTGGG
TGGTAAGGCGCAGTTCGGTGGTCAGCGTTTCGGGGAGATGGAGGTCTGGGCACTGGAAGCATACGGTGCCGCTTACACTCTGCAA

GAAATGCTCACAGTGAAGTCGGACGATGTGAACGGCCGTACCAAGATGTACAAAAACATCGTGGATGGCGATCACCGTATGGAG
CCGGGCATGCCCGAGTCCTTCAACGTGTTGATCAAGGAAATTCGTTCCCTCGGCATCGATATCGATCTGGAAACCGAATAA

P. fluorescens DR133_GCF_010668965.1_CP048607.1

ATGGCTTACTCATATACTGAGAAAAAACGTATCCGCAAGGACTTTAGCAAGTTGCCGGACGTCATGGATGTGCCGTACCTCCTGG
CCATCCAGCTGGATTCGTATCGTGAATTCTTGCAAGCGGGAGCGACTAAAGATCAGTTCCGCGACGTGGGCCTGCATGCGGCCTT
CAAATCCGTTTTCCCGATCATCAGCTACTCCGGCAATGCTGCGCTGGAGTACGTCGGTTATCGCCTGGGCGAACCGGCATTTGATG
TCAAAGAATGCGTATTGCGCGGTGTTACTTACGCCGTACCTTTGCGGGTAAAAGTGCGCCTGATCATTTTCGACAAAGAATCGTCG
AACAAAGCGATCAAGGACATCAAAGAGCAAGAAGTCTACATGGGTGAAATCCCCCTGATGACTGAGAACGGTACCTTCGTAATC
AACGGTACCGAGCGTGTAATCGTTTCCCAGCTGCACCGTTCTCCGGGCGTGTTCTTCGACCACGACCGTGGCAAGACGCACAGCT
CCGGCAAACTGCTGTACTCCGCGCGCATCATTCCTTACCGCGGTTCGTGGCTGGACTTCGAGTTCGACCCGAAAGACTGCGTATTC
GTGCGTATCGACCGTCGTCGCAAGCTGCCTGCATCGGTACTGCTGCGCGCGCTCGGCTATACCACCGAAGAAGTGCTGGACGCGT
TCTACACCACCAACGTCTTCCACGTGCAAGGTGAAAACCTCAGCCTGGAACTGGTGCCTCAGCGCCTGCGCGGTGAAATCGCTGT
CCTCGATATCCAGGATGACAAAGGCAAGGTTATTGTCGAGCAGGGTCGCCGTATCACCGCTCGCCACATCAACCAGCTGGAAAAA
GCCGGGATCAAAGAGCTGCAGGTGCCGATCGACTACGTCTTGGGTCGCACCACGGCCAAGGTCATCGTGCATCCGGCCACTGGCG
AAATCCTGGCAGAGTGCAATACCGAGCTGACCACCGAGATCCTGGCGAAAATCGCCAAGGCCCAGGTCGTTCGTATCGAAACGTT
GTACACCAACGACATCGACTGCGGTCCGTTCATCTCCGACACGCTGAAGATCGACTCCACCGGCAACCAGCTGGAAGCCCTGGTC
GAAATCTATCGCATGATGCGTCCTGGCGAGCCGCCAACCAAGGATGCAGCCGAGACCCTTTTCAACAACCTGTTCTTCAGCCCTG
AGCGCTATGACCTGTCTGCGGTCGGCCGGATGAAGTTCAACCGTCGTATCGGTCGTACCGAGATCGAAGGTTCGGGTGTTCTGAA
CAAGGACGACATCGTTGCGGTCCTCAAGACCCTGGTCGACATCCGTAACGGCAAAGGCATCGTCGATGACATCGACCACTTGGGT
AACCGTCGTGTTCGCTGTGTAGGCGAAATGGCCGAGAACCAGTTCCGTGTTGGCCTGGTGCGCGTAGAGCGTGCGGTCAAGGAAC
GCCTGTCGATGGCTGAAAGCGAAGGCCTGATGCCGCAAGACCTGATCAACGCCAAGCCTGTGGCTGCGGCGGTGAAAGAGTTCTT
CGGTTCGAGCCAGCTGTCCCAGTTCATGGACCAGAACAACCCGCTGTCCGAGATCACCCACAAGCGTCGTGTTTCCGCACTGGGC
CCAGGCGGTCTGACCCGTGAGCGTGCAGGCTTCGAAGTTCGTGACGTGCACCCGACGCACTACGGTCGTGTATGCCCGATCGAAA
CGCCGGAAGGTCCGAACATCGGTCTGATCAACTCCCTGGCTGCCTATGCGCGCACCAACCAGTACGGTTTCCTCGAGAGCCCGTA
TCGTGTGGTGAAAGACGCCCTGGTCACCGACGAGATCGTGTTCCTGTCCGCCATCGAAGAAGCCGATCACGTGATCGCCCAGGCT
TCGGCCACGATGAACGACAAGAAAGTCCTGACCGATGAATTGGTAGCTGTTCGTCACCTGAACGAGTTCACCGTCAAGGCGCCGG
AAGACGTCACCTTGATGGACGTATCGCCGAAGCAGGTAGTTTCGGTTGCAGCGTCGCTGATCCCGTTCCTCGAGCACGACGACGC
CAACCGTGCGTTGATGGGTTCGAACATGCAGCGTCAAGCTGTACCGACCCTGCGTGCCGACAAGCCGCTGGTAGGTACCGGCATG
GAGCGTAACGTTGCCCGTGACTCCGGCGTTTGCGTCGTGGCTCGTCGTGGTGGCGTGATCGACTCCGTCGACGCCAGCCGTATCGT
GGTTCGTGTTGCCGATGACGAAGTTGAAACTGGCGAAGCCGGTGTCGACATCTACAACCTGACCAAATATACCCGCTCCAACCAG
AACACCTGCATCAACCAGCGTCCGCTGGTAAGCAAGGGTGATCGGGTGCAGCGTAGCGACATCATGGCCGACGGTCCGTCCACCG
ATATGGGTGAACTGGCGTTGGGTCAGAACATGCGCATCGCGTTCATGGCATGGAACGGCTTCAACTTCGAAGACTCCATCTGCCT
GTCCGAGCGTGTGGTCCAGGAAGACCGTTTCACCACGATCCACATCCAGGAACTGACCTGTGTGGCCCGTGACACCAAGCTTGGC
CCAGAGGAAATCACTGCGGACATCCCGAACGTGGGTGAAGCTGCACTGAACAAACTGGACGAAGCCGGTATCGTTTACGTAGGT
GCTGAAGTAGGCGCAGGCGACATCCTGGTGGGCAAGGTCACTCCGAAAGGCGAGACCCAGCTGACTCCGGAAGAAAAACTGCTG
CGTGCCATCTTCGGTGAAAAAGCCAGCGACGTTAAAGACACCTCCCTGCGCGTGCCTACTGGCACCAAGGGTACCGTCATCGACG
TACAGGTCTTCACCCGTGACGGCGTCGAGCGTGATGCTCGTGCGCTGTCGATCGAGAAGACTCAACTCGACGAGATCCGCAAGGA
CCTGAACGAAGAGTTCCGTATCGTCGAAGGCGCAACTTTCGAGCGTCTGCGTTCCGCCCTGGTCGGCCACAAAGCCGAAGGCGGC
GCCGGCCTGAAGAAAGGTCAGGAAATCACCGACGAAGTCCTCGACGGTCTTGAGCATGGCCAGTGGTTCAAACTGCGCATGGCT
GAAGATGCTCTGAACGAGCAGCTCGAGAAGGCCCAGGCCTATATCGTTGATCGCCGCCGTCTGCTGGACGACAAGTTCGAAGACA
AGAAGCGCAAACTGCAGCAGGGCGATGACCTGGCTCCAGGCGTGCTGAAAATCGTCAAGGTTTACCTGGCAATCCGTCGTCGCAT
CCAGCCGGGCGACAAGATGGCCGGTCGTCACGGTAACAAGGGTGTGGTCTCTGTGATCATGCCGGTTGAAGACATGCCGCACGAT
GCCAATGGCACCCCGGTCGACGTGGTCCTCAACCCATTGGGCGTACCTTCGCGTATGAACGTCGGTCAGATCCTTGAAACCCACCT
GGGCCTCGCGGCCAAGGGCTTGGGCGAGAAGATCAACCGTATGATCGAAGAGCAGCGCAAGGTTGCTGACCTGCGTAAGTTCCT
GCACGAGATCTACAACGAGATCGGCGGCCGCAACGAAGAGCTGGACACCTTCTCCGACCAGGAAATCCTGGATCTGGCGAAGAA
CCTGCGCGGTGGCGTTCCAATGGCTACTCCGGTGTTCGACGGTGCCAAGGAAAGCGAAATCAAGGCCATGCTGAAACTGGCAGAC
CTGCCGGAAAGCGGCCAGATGCAGCTGTTCGACGGCCGTACCGGCAACAAGTTCGAGCGCCCGGTTACCGTTGGCTACATGTACA
TGCTGAAGCTGAACCACTTGGTGGACGACAAGATGCACGCTCGTTCTACCGGTTCTTACAGCCTGGTTACCCAGCAACCGCTGGG
TGGTAAGGCGCAGTTCGGTGGTCAGCGTTTCGGGGAGATGGAGGTCTGGGCACTGGAAGCGTACGGTGCTGCATACACTCTGCAA
GAAATGCTCACAGTGAAGTCGGACGATGTGAACGGCCGGACCAAGATGTACAAAAACATCGTGGACGGCGATCACCGTATGGAG
CCGGGCATGCCCGAGTCTTTCAACGTGTTGATCAAGGAAATTCGTTCCCTCGGCATCGATATCGATCTGGAAACCGAATAA

P. fluorescens G7_GCF_012974785.1_CP027561.1

ATGGCTTACTCATATACTGAGAAAAAACGTATCCGCAAGGACTTTAGCAAGTTGCCGGACGTCATGGATGTGCCTTACCTCCTGGC
CATCCAGCTGGATTCGTATCGTGAATTCTTGCAAGCGGGAGCGACTAAAGATCAGTTCCGCGACGTGGGCCTGCATGCGGCCTTC
AAATCCGTTTTCCCGATCATCAGCTACTCCGGCAATGCTGCGCTGGAGTACGTCGGTTATCGCCTGGGCGAACCGGCATTTGATGT
CAAAGAATGCGTATTGCGCGGTGTAACTTTCGCCGTACCTTTGCGGGTAAAAGTGCGCCTGATCATTTTCGACAAAGAATCGTCGA
ACAAAGCGATCAAGGACATCAAAGAGCAAGAAGTCTACATGGGTGAAATCCCATTGATGACTGAGAACGGTACCTTCGTAATCA
ACGGTACCGAGCGTGTGATCGTTTCCCAGTTGCACCGCTCCCCGGGCGTGTTCTTCGACCACGACCGTGGCAAGACGCACAGCTC
CGGCAAACTGCTGTACTCCGCGCGCATCATTCCTTACCGCGGTTCGTGGCTGGACTTCGAATTCGACCCGAAAGACTGCGTATTCG
TGCGTATCGACCGTCGTCGCAAGCTGCCGGCCTCGGTACTGCTGCGCGCGCTCGGTTATACCACCGAGGAAGTGCTGGACGCGTT
CTACACCACCAACGTATTCCACGTGAAAGGCGAAGGCCTGAGCCTGGAGCTGGTGCCTCAGCGCCTGCGTGGTGAAGTTGCTGTT
CTCGACATCCAGGACGACAAAGGCAAGGTGATTGTCGAGCAGGGTCGTCGTATTACTGCCCGCCACATCAACCAGCTGGAAAAA

GCCGGGATCAAAGAGCTGGACGTGCCTCTGGATTACGTTCTGGGTCGTACCACCGCCAAGGCCATCGTGCACCCGGCTACCGGTG
AGATCCTGGCAGAGTGCAACACCGAACTGAGCACTGAAGTTCTGGCGAAGGTCGCCAAGGCCGGTGTCGTTCGTATTGAAACTCT
GTACACCAACGATATCGACTGCGGTCCGTTCATCTCCGACACACTGAAGATCGACTCCACCAGCAACCAATTGGAAGCGCTGGTC
GAAATCTATCGCATGATGCGTCCGGGCGAGCCGCCAACCAAGGATGCTGCCGAGACCCTGTTCAACAACCTGTTCTTCAGCCCTG
AGCGCTATGACCTGTCTGCGGTCGGTCGGATGAAGTTCAACCGTCGTATCGGTCGTACCGAGATCGAAGGTTCGGGCGTGCTGTG
CAAGGAAGACATCGTCGCGGTACTGAAGACTCTGGTCGACATCCGTAACGGTAAAGGCATCGTCGATGACATCGACCACCTGGGT
AACCGTCGTGTTCGCTGCGTAGGCGAAATGGCCGAGAACCAGTTCCGCGTTGGCCTGGTACGTGTTGAGCGTGCGGTCAAAGAGC
GTCTGTCGATGGCAGAAAGCGAAGGTCTGATGCCGCAAGACCTGATCAACGCCAAGCCAGTGGCTGCGGCGGTGAAAGAGTTCTT
CGGTTCCAGCCAGCTGTCGCAGTTCATGGACCAGAACAACCCGCTGTCCGAGATCACCCACAAGCGTCGTGTCTCCGCACTCGGC
CCTGGCGGTCTGACTCGTGAGCGCGCAGGCTTCGAAGTCCGTGACGTACACCCGACTCACTACGGTCGTGTCTGCCCGATTGAAA
CGCCGGAAGGTCCGAACATCGGTCTGATCAACTCCCTCGCCGCTTATGCGCGTACCAACCAGTACGGCTTCCTTGAGAGCCCGTA
CCGTGTGGTGAAAGAGGGTGTGGTCACCGACGATATCGTGTTCCTGTCCGCTATCGAAGAAGCCGATCACGTGATCGCGCAGGCT
TCGGCGACCATGAACGAGCAGAAAGTCCTGGTCGACGAACTGGTAGCCGTACGTCACCTGAACGAATTCACCGTCAAGGCGCCTG
AAGAAGTCACCTTGATGGACGTTTCGCCGAAGCAGGTAGTTTCGGTTGCAGCGTCGTTGATTCCGTTCCTCGAGCACGACGACGCC
AACCGTGCGTTGATGGGTTCGAACATGCAGCGTCAGGCTGTACCAACCCTGCGTGCCGACAAGCCGCTGGTAGGTACCGGCATGG
AGCGTAACGTGGCTCGCGACTCCGGCGTTTGCGTCGTGGCTCGTCGTGGCGGCGTGATCGACTCCGTTGATGCCAGCCGTATCGTG
GTTCGTGTTGCCGATGACGAAGTTGAAACCGGTGAAGCTGGTGTCGACATCTACAACCTGACCAAGTACACCCGCTCGAACCAGA
ACACCTGCATCAACCAGCGTCCGCTGGTGAGCAAAGGTGATCGCGTTCAGCGTAGTGACATCATGGCTGACGGCCCGTCCACCGA
CATGGGTGAGCTGGCTCTGGGTCAGAACATGCGCATCGCGTTCATGGCGTGGAACGGCTTCAACTTCGAAGACTCCATCTGCCTGT
CCGAGCGTGTGGTTCAGGAAGACCGCTTTACCACGATCCACATCCAGGAACTGACCTGTGTGGCTCGTGACACCAAGCTTGGCCC
AGAGGAAATCACTGCGGACATCCCGAACGTGGGTGAAGCTGCACTGAACAAGCTGGACGAAGCCGGTATCGTTTATGTAGGTGC
CGAAGTTGGCGCAGGCGACATCCTGGTAGGCAAGGTCACTCCGAAAGGCGAGACCCAACTGACCCCGGAAGAAAAACTGCTGCG
TGCGATCTTCGGTGAAAAAGCCAGCGACGTTAAAGACACTTCCCTGCGCGTACCTACCGGTACCAAAGGTACGGTCATCGACGTG
CAGGTCTTCACTCGCGACGGCGTTGAGCGTGATGCTCGTGCACTGTCGATCGAGAAGTCCCAGCTGGACGAGATCCGCAAGGATC
TGAACGAAGAGTTCCGCATCGTTGAAGGCGCGACCTTCGAACGTCTGCGTTCCGCTCTGGTAGGCCACAAGGCTGAAGGCGGCGC
AGGTCTGAAGAAAGGTCAGGACATCACCGACGAAGTACTCGACGGTCTTGAGCATGGTCAGTGGTTCAAACTGCGCATGGCTGAA
GATGCTCTGAACGAGCAGCTCGAGAAGGCTCAGGCCTACATCGTTGATCGTCGCCGTCTGCTGGACGACAAGTTCGAAGACAAGA
AGCGCAAACTGCAGCAGGGCGATGACCTGGCTCCAGGCGTGCTGAAGATCGTCAAGGTTTACCTGGCAATCCGTCGTCGCATCCA
GCCGGGCGACAAGATGGCCGGTCGTCACGGTAACAAGGGTGTGGTCTCTGTGATCATGCCGGTTGAAGACATGCCGCACGATGCC
AATGGCACCCCGGTCGACGTGGTCCTCAACCCGCTGGGCGTACCTTCGCGTATGAACGTTGGTCAGATCCTTGAAACCCACCTGG
GTCTCGCGGCCAAAGGTCTGGGCGAGAAGATCAACCGGATGGTCGAAGAGCAGCGCAAGGTCGCTGAACTGCGCACCTTCCTGG
ACGAGATCTACAACCAGATCGGCGGTCGTAACGAAGATCTGGACAGCTTCTCCGATCAGGAAATCCTCGATCTGGCGAAGAACCT
GCGTGGCGGCGTTCCAATGGCCACTCCAGTGTTCGACGGTGCCAAGGAAAGCGAAATCAAGGCCATGCTGAAACTGGCGGACCT
GCCAGAAAGCGGCCAGATGCAGCTGACCGACGGTCGTACCGGCAACAAGTTCGAGCGTCCAGTTACCGTTGGCTACATGTACATG
CTGAAGCTGAACCACTTGGTAGACGACAAGATGCACGCGCGTTCTACCGGTTCGTACAGCCTGGTTACCCAGCAGCCGCTGGGTG
GTAAGGCGCAGTTCGGTGGTCAGCGTTTCGGGGAGATGGAGGTCTGGGCACTGGAAGCATACGGTGCTGCTTACACTCTGCAAGA
AATGCTCACAGTGAAGTCGGACGATGTGAACGGCCGGACCAAGATGTACAAAAACATCGTGGACGGCGATCACCGTATGGAGCC
GGGCATGCCCGAGTCCTTCAACGTGTTGATCAAGGAAATTCGTTCCCTCGGCATCGATATCGATCTGGAAACCGAATAA

P. fluorescens KF1_GCF_015074865.1_CP063233.1

ATGGCTTACTCATATACTGAGAAAAAACGTATCCGCAAGGACTTTAGCAAGTTGCCGGACGTCATGGATGTCCCGTACCTTCTGGC
TATCCAGCTGGATTCGTATCGTGAATTCTTGCAGGCGGGAGCGACCAAAGATCAGTTCCGCGACGTGGGCCTGCATGCGGCCTTC
AAATCCGTTTTCCCGATCATCAGCTACTCCGGCAATGCTGCGCTGGAGTACGTGGGTTATCGCCTGGGCGAACCGGCATTTGATGT
CAAAGAATGCGTGTTGCGCGGTGTTACGTACGCCGTACCTTTGCGGGTAAAAGTCCGTCTGATCATTTTCGACAAAGAATCGTCGA
ACAAAGCGATCAAGGACATCAAAGAGCAAGAAGTCTACATGGGCGAAATCCCATTGATGACTGAAAACGGTACCTTCGTTATCA
ACGGTACCGAGCGTGTTATCGTTTCCCAGCTGCACCGTTCTCCGGGCGTGTTCTTCGACCACGACCGCGGCAAGACGCACAGCTCC
GGCAAACTCCTGTACTCCGCGCGGATCATTCCGTACCGTGGTTCGTGGTTGGACTTTGAGTTCGACCCGAAAGACTGCGTGTTCGT
GCGTATCGACCGTCGTCGCAAGCTGCCAGCCTCGGTATTGCTGCGCGCGCTTGGCTACACCACTGAGCAAGTGCTGGACGCTTTCT
ACACCACCAACGTATTCAGCCTGAAGGATGAAACCCTCAGCCTGGAGCTGATTGCTTCGCGTCTGCGTGGTGAAATTGCTGTTCTG
GACATCCAGGATGAAAAGGGCAAGGTCATTGTTGAGGCTGGCCGTCGTATTACTGCGCGCCACATCAACCAGATCGAAAAAGCC
GGTATCAAGTCGCTGGACGTGCCTTTGGACTACGTCCTGGGTCGCACGACCGCCAAGGTCATCGTTCACCCAGCCACAGGCGAGA
TCCTGGCTGAGTGCAACACTGAGCTGAACACCGAAATCCTGGCAAAAATCGCCAAGGCCCAGGTCGTTCGCATCGAGACTTTGTA
CACCAACGATATCGACTGCGGTCCGTTCGTTTCCGACACCCTGAAGATCGACTCCACCAGCAACCAATTGGAAGCGCTGGTCGAG
ATCTATCGCATGATGCGTCCTGGCGAGCCACCAACCAAAGACGCTGCCGAAACCCTGTTCAACAACCTGTTCTTCAGCCCTGAGC
GCTATGACCTGTCTGCGGTCGGCCGGATGAAGTTCAACCGTCGTATCGGTCGCACCGAAATCGAAGGTTCGGGCGTGCTGTGCAA
GGAAGACATCGTTGCGGTCCTGAAGACTCTGGTCGACATCCGCAACGGTAAGGGCATCGTTGATGACATCGACCACCTGGGTAAC
CGTCGTGTTCGCTGCGTAGGCGAAATGGCCGAGAACCAGTTCCGCGTTGGCCTGGTGCGTGTTGAGCGTGCGGTCAAAGAGCGTT
TGTCGATGGCTGAAAGCGAAGGCCTGATGCCGCAAGACCTGATCAACGCCAAGCCAGTGGCTGCGGCGGTGAAAGAGTTCTTCG
GTTCCAGCCAGCTCTCGCAGTTCATGGACCAGAACAACCCGCTCTCCGAGATCACCCACAAGCGCCGTGTTTCCGCACTGGGCCC
GGGCGGTCTGACCCGTGAGCGTGCAGGTTTTGAAGTTCGTGACGTACACCCGACTCACTATGGTCGTGTATGCCCGATCGAAACG
CCCGAAGGTCCGAACATCGGCCTGATCAACTCCCTGGCAGCCTATGCGCGCACCAACCAGTACGGTTTCCTTGAAAGCCCGTACC
GCGTGGTGAAAGACGCCCTGGTCACCGACGAGATCGTATTCCTGTCGGCCATCGAAGAAGCTGATCACGTGATCGCTCAGGCTTC
GGCCACGATGAACGACAAGAAAGTACTGATTGACGAGTTGGTAGCTGTTCGTCACTTGAACGAGTTCACCGTCAAGGCGCCGGAA
GACGTCACCTTGATGGACGTATCGCCGAAGCAGGTAGTCTCGGTTGCTGCGTCGCTGATCCCGTTCCTGGAGCACGATGACGCCA

ACCGTGCGTTGATGGGTTCTAACATGCAGCGTCAAGCTGTACCTACACTGCGTGCCGACAAGCCGCTGGTGGGTACCGGCATGGA
GCGTAACGTAGCTCGTGACTCCGGCGTTTGCGTCGTGGCTCGTCGTGGCGGCGTGATCGACTCCGTTGATGCCAGCCGTATCGTGG
TTCGTGTTGCTGATGACGAAGTTGAAACTGGCGAAGCCGGTGTCGACATCTACAACCTGACCAAATACACCCGCTCGAACCAGAA
CACCTGCATCAACCAGCGTCCGCTGGTGCGTAAGGGTGATCGCGTTCAGCGTAGCGACATCATGGCCGACGGCCCGTCCACCGAC
ATGGGTGAACTGGCGCTGGGCCAGAACATGCGCATTGCGTTCATGGCATGGAACGGCTTCAACTTCGAAGACTCCATCTGCCTGT
CCGAGCGTGTTGTTCAAGAAGACCGCTTCACCACGATCCACATCCAGGAACTGACCTGTGTGGCGCGTGATACCAAGCTTGGGCC
AGAGGAAATCACTGCAGACATCCCGAACGTGGGTGAAGCTGCACTGAACAAACTGGACGAAGCCGGTATCGTTTATGTAGGTGCT
GAAGTTGGCGCGGGCGACATCCTGGTTGGTAAGGTCACTCCGAAAGGCGAGACCCAACTGACTCCGGAAGAGAAGCTGTTGCGT
GCCATCTTCGGTGAAAAAGCCAGCGACGTTAAAGACACTTCCCTGCGCGTACCGACCGGTACCAAGGGTACTGTCATCGACGTAC
AGGTCTTCACCCGTGACGGCGTTGAGCGTGATGCTCGTGCACTGTCCATCGAGAAGACCCAGCTCGACGAGATCCGCAAGGACCT
GAACGAAGAGTTTCGTATCGTTGAAGGCGCAACCTTCGAGCGTCTGCGTTCCGCCCTGGTAGGTCACAAGGCTGAAGGCGGTGCG
GGCCTTAAGAAAGGTCAGGACATCACCGACGAAGTGCTCGACGGTCTTGAGCACGGCCAGTGGTTCAAACTGCGCATGGCTGAA
GATGCTCTGAACGAGCAGCTCGAGAAGGCCCAGGCCTATATCGTTGATCGCCGCCGTCTGCTGGACGACAAGTTCGAAGACAAGA
AGCGCAAACTGCAGCAGGGCGATGACCTGGCTCCAGGTGTGCTGAAAATCGTCAAGGTTTACCTGGCAATCCGTCGTCGCATCCA
GCCGGGCGACAAGATGGCCGGTCGTCACGGTAACAAGGGTGTGGTCTCCGTGATCATGCCGGTTGAAGACATGCCGCACGATGCC
AATGGCACCCCGGTCGACGTCGTCCTCAACCCGTTGGGCGTACCATCGCGTATGAACGTTGGTCAGATCCTCGAAACCCACCTGG
GCCTCGCGGCTAAAGGCCTGGGTGAGAAGATCAACCGTATGATCGAAGAGCAGCGCAAGGTTGCTGACCTGCGTAAGTTCCTGCA
CGAGATCTACAACGAGATCGGCGGTCGCAAAGAAGAGCTGGACACTTTCTCCGACCAGGAAATCCTGGATCTGGCGAAAAACCT
GCGCGGCGGCGTTCCAATGGCTACCCCGGTGTTCGATGGTGCCAAGGAAAGCGAAATCAAGGCCATGCTCAAACTGGCAGACCT
GCCAGAAAGCGGCCAGATGCAGCTGTTCGACGGCCGTACCGGCAACAAGTTTGAACGCCCGGTTACTGTTGGCTACATGTACATG
CTGAAGCTGAACCACTTGGTAGACGACAAGATGCACGCTCGTTCTACCGGTTCGTACAGCCTGGTTACCCAGCAGCCGCTGGGTG
GTAAGGCTCAGTTCGGTGGTCAGCGTTTCGGGGAGATGGAGGTCTGGGCACTGGAGGCATACGGTGCTGCTTACACTCTGCAAGA
AATGCTCACAGTGAAGTCGGACGATGTGAACGGTCGGACCAAGATGTACAAAAACATCGTGGACGGCGATCACCGTATGGAGCC
GGGCATGCCCGAGTCCTTCAACGTGTTGATCAAAGAAATTCGTTCCCTCGGCATCGATATCGATCTGGAAACCGAATAA

P. fluorescens FDAARGOS_1088_GCF_016728065.1_CP068151.1

ATGGCTTACTCATATACTGAGAAAAAACGTATCCGCAAGGACTTTAGCAAGTTGCCGGACGTCATGGATGTCCCGTACCTTCTGGC
TATCCAGCTGGATTCGTATCGTGAATTCTTGCAGGCGGGAGCGACCAAAGATCAGTTCCGCGACGTGGGCCTGCATGCGGCCTTC
AAATCCGTTTTCCCGATCATCAGCTACTCCGGCAATGCTGCGCTGGAGTACGTCGGTTATCGCCTGGGCGAACCGGCATTTGATGT
CAAAGAATGCGTGTTGCGTGGCGTTACGTACGCCGTACCTTTGCGGGTAAAAGTCCGTCTGATCATTTTCGACAAAGAATCGTCGA
ACAAAGCGATCAAGGACATCAAAGAGCAAGAAGTCTACATGGGCGAAATCCCACTGATGACTGAAAACGGTACCTTCGTAATCA
ACGGTACCGAGCGTGTAATCGTTTCCCAGCTGCACCGTTCCCCGGGCGTGTTCTTCGACCACGACCGCGGCAAGACGCACAGCTC
CGGCAAACTCCTGTACTCCGCGCGGATCATTCCGTACCGCGGTTCGTGGTTGGACTTCGAGTTCGACCCGAAAGACTGCGTGTTCG
TGCGTATCGACCGTCGTCGCAAGCTGCCGGCCTCGGTATTGCTGCGCGCGCTCGGTTACACCACTGAGCAAGTGCTGGATGCTTTC
TATACCACCAACGTATTCAGCCTGAAGGATGAAACCCTCAAGCTGGAGCTGATCGCTTCGCGTCTGCGTGGTGAAATTGCTGTACT
GGACATCCAGGACGAAAAAGGCAAGGTCATCGTTGAGGCTGGCCGTCGTATCACTGCGCGCCACATCAACCAGATCGAAAAAGC
CGGTATCAAAGAGCTGGAAGTGCCACTGGACTACGTCCTGGGTCGCACTACCGCCAAGGTCATCGTTCACCCGGCTACAGGCGAA
ATCCTGGCTGAGTGCAACACCGAGCTGAACACCGAGATCCTGGCCAAGATCGCCAAGGCCCAGGTTGTTCGCATCGAGACCCTGT
ACACCAACGATATCGACTGCGGTCCGTTCATCTCCGACACACTGAAGATCGACTCCACCAGCAACCAATTGGAAGCGCTGGTCGA
GATCTATCGCATGATGCGTCCTGGTGAGCCACCAACCAAAGACGCTGCCGAAACCCTGTTCAACAACCTGTTCTTCAGCCCTGAG
CGTTATGACCTGTCTGCGGTCGGCCGGATGAAGTTCAACCGTCGTATCGGTCGTACCGAGATCGAAGGTTCGGGCGTGCTGTGCA
AGGAAGACATCGTCGCGGTATTGAAGACCCTGGTTGACATCCGTAACGGCAAAGGCATCGTCGATGACATCGACCACCTGGGTAA
CCGTCGTGTTCGCTGCGTAGGCGAAATGGCCGAGAACCAGTTCCGTGTTGGCCTGGTGCGTGTTGAGCGTGCGGTCAAAGAACGT
CTGTCGATGGCTGAAAGCGAAGGCCTGATGCCGCAAGACCTGATCAACGCCAAGCCAGTGGCTGCGGCGGTGAAAGAGTTCTTC
GGTTCCAGCCAGCTTTCGCAGTTCATGGACCAGAACAACCCGCTCTCCGAGATCACCCACAAGCGCCGTGTTTCTGCACTGGGCCC
GGGCGGTCTGACCCGTGAGCGTGCAGGCTTTGAAGTTCGTGACGTACACCCGACGCACTACGGTCGTGTTTGCCCGATCGAAACG
CCGGAAGGTCCGAACATCGGTCTGATCAACTCCCTGGCCGCTTATGCGCGCACCAACCAGTACGGCTTCCTCGAAAGCCCGTACC
GTGTGGTGAAAGACGCTCTGGTCACCGACGAGATCGTGTTCCTGTCCGCCATCGAAGAAGCTGATCACGTGATCGCTCAGGCTTC
GGCCACGATGAACGACAAGAAAGTCCTGATCGACGAGCTGGTAGCTGTTCGTCACTTGAACGAGTTCACCGTCAAGGCGCCGGA
AGACGTCACCTTGATGGACGTTTCTCCGAAGCAGGTAGTGTCGGTTGCAGCGTCGCTGATCCCGTTCCTGGAACACGATGACGCC
AACCGTGCGTTGATGGGTTCCAACATGCAGCGTCAAGCTGTACCGACCCTGCGTGCTGACAAGCCGCTGGTAGGTACCGGCATGG
AGCGCAACGTAGCCCGTGACTCCGGCGTTTGCGTCGTGGCTCGTCGTGGCGGCGTGATCGACTCCGTTGATGCCAGCCGTATCGTG
GTTCGTGTTGCCGATGACGAAGTAGAAACTGGCGAAGCCGGTGTCGACATCTACAACCTGACCAAATACACCCGCTCGAACCAGA
ACACCTGCATCAACCAGCGTCCGCTGGTGAGCAAGGGTGATCGCGTTCAGCGTAGCGACATCATGGCCGACGGCCCGTCCACCGA
CATGGGTGAGCTGGCTCTGGGTCAGAACATGCGCATCGCGTTCATGGCATGGAACGGCTTCAACTTCGAAGACTCCATCTGCCTGT
CCGAGCGTGTGGTTCAAGAAGACCGCTTCACCACGATCCACATCCAGGAACTGACCTGTGTGGCACGTGATACCAAGCTTGGGCC
AGAGGAAATCACTGCAGACATCCCGAACGTGGGTGAAGCTGCACTGAACAAGCTGGACGAAGCCGGTATCGTTTACGTAGGTGC
TGAAGTTGGCGCTGGCGACATCCTGGTCGGTAAGGTCACTCCGAAAGGCGAGACCCAACTGACGCCGGAAGAGAAGCTGCTGCG
TGCCATCTTCGGTGAAAAAGCCAGCGACGTTAAAGACACTTCCCTGCGTGTACCTACCGGTACCAAGGGTACTGTCATCGACGTA
CAGGTCTTCACCCGTGACGGCGTTGAGCGTGATGCTCGTGCACTGTCCATTGAGAAGACCCAGCTCGACGAGATCCGCAAGGACC
TGAACGAAGAGTTCCGTATCGTTGAAGGCGCGACCTTCGAACGTCTGCGTTCCGCTCTGGTAGGCCACAAGGCTGAAGGCGGCGC
AGGTCTGAAGAAAGGTCAGGACATCACCGACGAAATCCTCGACGGTCTTGAGCACGGCCAGTGGTTCAAACTGCGCATGGCTGA
AGACGCTCTGAACGAGCAGCTTGAGAAGGCCCAGGCCTATATCGTTGATCGCCGCCGTCTGCTGGACGACAAGTTCGAAGACAAG
AAGCGCAAACTGCAGCAGGGCGATGACCTGGCTCCAGGCGTGCTGAAAATCGTCAAGGTTTACCTGGCAATCCGTCGCCGCATCC

AACCGGGCGACAAGATGGCCGGTCGTCACGGTAACAAAGGTGTGGTCTCCGTGATCATGCCGGTTGAAGACATGCCGCACGATG
CCAATGGCACCCCGGTCGACGTCGTCCTTAACCCGTTGGGCGTACCTTCGCGTATGAACGTTGGTCAGATCCTTGAAACCCACCTG
GGCCTCGCGGCCAAAGGTCTGGGCGAGAAGATCAACCGTATGATCGAAGAGCAGCGCAAGGTCGCAGACCTGCGCAAGTTCCTG
CACGAGATCTACAACGAGATCGGCGGTCGCAACGAAGAGCTGGACACCTTCTCCGACCAGGAAATCCTGGATCTGGCGAAGAAC
CTGCGCGGCGGCGTTCCAATGGCTACCCCGGTGTTCGACGGTGCCAAGGAAAGCGAAATCAAGGCCATGCTGAAACTGGCAGAC
CTGCCGGAAAGCGGCCAGATGCAGCTGTTCGACGGCCGTACCGGCAACAAGTTTGAGCGCCCGGTTACTGTTGGCTACATGTACA
TGCTGAAGCTGAACCACTTGGTAGACGACAAGATGCACGCTCGTTCTACCGGTTCGTACAGCCTGGTTACCCAGCAGCCGCTGGG
TGGTAAGGCTCAGTTCGGTGGTCAGCGTTTCGGGGAGATGGAGGTCTGGGCACTGGAAGCATACGGTGCTGCATACACTCTGCAA
GAAATGCTCACAGTGAAGTCGGACGATGTGAACGGTCGTACCAAGATGTACAAAAACATCGTGGACGGCGATCACCGTATGGAG
CCGGGCATGCCCGAGTCCTTCAACGTGTTGATCAAAGAAATTCGTTCCCTCGGCATCGATATCGATCTGGAAACCGAATAA

P. fluorescens YK-310_GCF_017498465.1_CP071797.1

ATGGCTTACTCATATACTGAGAAAAAACGTATCCGCAAGGACTTTAGCAAGTTGCCGGACGTCATGGATGTGCCTTACCTCCTGGC
CATCCAGCTGGATTCGTATCGTGAATTCTTGCAAGCGGGAGCGACTAAAGATCAGTTCCGCGACGTGGGCCTGCATGCGGCCTTC
AAATCCGTTTTCCCGATCATCAGCTACTCCGGCAATGCTGCGCTGGAGTACGTCGGTTATCGCCTGGGCGAACCGGCATTTGATGT
CAAAGAATGCGTATTGCGCGGTGTAACTTTCGCCGTACCTTTGCGGGTAAAAGTGCGCCTGATCATTTTCGACAAAGAATCGTCGA
ACAAAGCGATCAAGGACATCAAAGAGCAAGAAGTCTACATGGGTGAAATCCCATTGATGACTGAGAACGGTACCTTCGTAATCA
ACGGTACCGAGCGTGTTATCGTTTCCCAGCTGCACCGTTCCCCGGGCGTGTTCTTCGACCACGACCGTGGCAAGACGCACAGCTCC
GGCAAACTGCTGTACTCCGCGCGCATCATTCCTTACCGCGGTTCGTGGCTGGACTTTGAGTTCGACCCGAAAGACTGCGTATTCGT
GCGTATCGACCGTCGTCGTAAGCTGCCGGCCTCGGTACTGCTGCGCGCGCTCGGTTATACCACCGAGGAAGTGCTGGACGCGTTC
TACACCACCAACGTATTCCACGTGAAAGGCGAAGGCCTGAGCCTGGAGCTGGTGCCTCAGCGCCTGCGTGGTGAAGTTGCTGTTC
TCGACATCCAGGACGACAAAGGCAAGGTGATCGTTGAGCAGGGCCGTCGTATTACTGCCCGCCACATCAACCAGCTGGAAAAAG
CCGGGATCAAAGAGCTGGACGTGCCTCTGGATTACGTTCTGGGTCGTACCACCGCCAAGGCCATCGTGCACCCGGCTACCGGTGA
GATCCTGGCAGAGTGCAACACCGAGCTGTCGACCGAGATCCTGGCGAAAATCGCCAAGGCCGGCGTCGTACGCATCGAAACTCT
GTACACCAACGACATCGACTGCGGTCCGTTCGTCTCCGACACGCTGAAGATCGACTCCACCAGCAACCAATTGGAAGCGCTGGTC
GAGATCTATCGCATGATGCGTCCAGGCGAGCCGCCAACCAAGGACGCTGCCGAGACCCTGTTCAACAACCTGTTCTTCAGCCCTG
AGCGCTACGACCTGTCTGCGGTCGGCCGGATGAAGTTCAACCGTCGTATCGGTCGTACCGAGATCGAAGGTTCGGGCGTGCTATG
CAAGGAAGACATCGTCGCGGTACTGAAGACTCTGGTCGACATCCGTAACGGTAAAGGCATCGTCGACGACATCGACCACCTGGGT
AACCGTCGTGTTCGCTGCGTAGGCGAAATGGCCGAGAACCAGTTCCGTGTTGGCCTGGTACGTGTTGAGCGTGCGGTCAAAGAGC
GTCTGTCGATGGCTGAAAGCGAAGGCCTGATGCCGCAAGATCTGATCAACGCCAAGCCAGTGGCTGCGGCGGTGAAAGAGTTCTT
CGGTTCCAGCCAGCTGTCGCAGTTCATGGACCAGAACAACCCGCTGTCCGAGATCACCCACAAGCGTCGTGTCTCTGCACTCGGC
CCTGGCGGTCTGACTCGTGAGCGCGCGGGCTTCGAAGTCCGTGACGTACACCCGACTCACTACGGTCGTGTCTGCCCGATTGAAA
CGCCGGAAGGTCCGAACATCGGCCTGATCAACTCCCTGGCTGCTTACGCTCGCACCAACCAGTACGGCTTCCTCGAGAGCCCGTA
CCGTGTGGTGAAAGAGGGTGTGGTCACCGACGACATCGTGTTCCTGTCCGCCATTGAAGAAGCCGATCACGTGATCGCGCAGGCT
TCGGCGACCATGAACGAGCAGAAAGTCCTGATCGACGAACTGGTAGCTGTACGTCACCTGAACGAATTCACCGTCAAGGCGCCG
GAAGACGTCACCTTGATGGACGTATCGCCGAAGCAGGTAGTTTCGGTTGCAGCGTCGCTGATTCCGTTCCTCGAGCACGACGACG
CCAACCGTGCGTTGATGGGTTCGAACATGCAGCGTCAAGCTGTACCAACCCTGCGCGCTGACAAGCCGCTGGTCGGTACCGGCAT
GGAGCGTAACGTGGCTCGCGACTCCGGCGTTTGCGTCGTGGCTCGTCGTGGCGGCGTGATCGACTCCGTTGATGCCAGCCGTATC
GTGGTTCGTGTTGCTGATGACGAAGTTGAAACCGGCGAAGCTGGTGTCGACATCTACAACCTGACCAAATACACCCGTTCGAACC
AGAACACCTGCATTAACCAGCGTCCGCTGGTGAGCAAAGGTGATCGCGTTCAGCGTAGCGACATCATGGCCGACGGCCCGTCCAC
CGACATGGGTGAACTGGCTCTGGGTCAGAACATGCGCATCGCGTTCATGGCATGGAACGGCTTCAACTTCGAAGACTCCATCTGC
CTGTCCGAGCGTGTGGTTCAGGAAGACCGTTTCACCACGATCCACATTCAGGAACTGACCTGTGTGGCCCGTGACACCAAGCTTG
GCCCAGAGGAAATCACTGCGGACATCCCGAACGTGGGTGAAGCCGCTCTGAACAAACTGGACGAAGCCGGTATCGTTTACGTAG
GTGCTGAAGTCGGCGCAGGCGACATCCTGGTAGGTAAGGTCACTCCAAAAGGCGAGACCCAACTGACTCCGGAAGAAAAACTGC
TGCGTGCGATCTTCGGTGAAAAAGCCAGCGACGTTAAAGACACCTCCCTGCGCGTGCCTACCGGCACCAAAGGTACTGTCATCGA
CGTACAGGTCTTCACCCGTGACGGCGTTGAGCGTGATGCTCGTGCACTGTCGATCGAGAAGTCCCAGCTGGACGAGATCCGCAAG
GATCTGAACGAAGAGTTCCGCATCGTTGAAGGCGCGACCTTCGAGCGTCTGCGTTCCGCTCTGGTAGGCCACAAGGCTGAAGGCG
GCGCGGGCCTGAAGAAAGGTCAGGACATCACCGACGAAGTACTCGACGGTCTTGAGCATGGTCAGTGGTTCAAACTGCGCATGG
CTGAAGATGCTCTGAACGAGCAGCTCGAGAAGGCCCAGGCCTACATCGTTGATCGTCGCCGTCTGCTGGACGACAAGTTCGAAGA
CAAGAAGCGCAAACTGCAGCAGGGCGATGACCTGGCTCCAGGCGTGCTGAAGATCGTCAAGGTTTACCTGGCAATCCGTCGTCGC
ATCCAGCCGGGCGACAAGATGGCCGGTCGTCACGGTAACAAAGGTGTGGTCTCCGTGATCATGCCGGTTGAAGACATGCCGCACG
ATGCCAATGGCACCCCGGTCGACGTGGTCCTCAACCCGCTGGGCGTACCTTCGCGTATGAACGTTGGTCAGATCCTTGAAACCCA
CCTGGGCCTCGCGGCCAAAGGTCTGGGCGAGAAGATCAACCGGATGGTCGAAGAACAGCGCAAAGTCGCTGAGCTGCGTACCTT
CCTGGACGAGATCTACAACCAGATCGGCGGTCGTAACGAAGATCTGGACAGCTTCTCCGATCAGGAAATCCTGGATCTGGCGAAG
AACCTGCGTGGCGGTGTTCCAATGGCCACTCCAGTGTTCGACGGTGCCAAGGAAAGCGAAATCAAGGCCATGCTGAAACTGGCA
GACCTGCCAGAAAGCGGCCAGATGCAGCTGACCGACGGCCGTACCGGCAACAAGTTCGAGCGTCCAGTTACCGTTGGCTACATGT
ACATGCTGAAGCTGAACCACTTGGTAGACGACAAGATGCACGCGCGTTCTACCGGTTCGTACAGCCTGGTTACCCAGCAGCCGCT
GGGTGGTAAGGCGCAGTTCGGTGGTCAGCGTTTCGGGGAGATGGAGGTCTGGGCACTGGAAGCATACGGTGCTGCTTACACTCTG
CAAGAAATGCTCACAGTGAAGTCGGACGATGTGAACGGCCGGACCAAGATGTACAAAAACATCGTGGACGGCGATCACCGTATG
GAGCCGGGCATGCCCGAGTCCTTCAACGTGTTGATCAAGGAAATTCGTTCCCTCGGCATCGATATCGATCTGGAAACCGAATAA

P. fluorescens JNU01_GCF_017948385.1_CP072873.1

ATGGCTTACTCATATACTGAGAAAAAACGTATCCGCAAGGACTTTAGCAAGTTGCCGGACGTCATGGATGTCCCGTACCTTCTGGC
TATCCAGCTGGATTCGTATCGTGAATTCTTGCAGGCGGGAGCGACCAAAGATCAGTTCCGCGACGTGGGCCTGCATGCGGCCTTC

AAATCCGTTTTCCCGATCATCAGCTACTCCGGCAATGCTGCGCTGGAGTACGTCGGTTATCGCCTGGGCGAACCGGCATTTGATGT
CAAAGAATGCGTGTTGCGCGGTGTTACGTACGCCGTACCTTTGCGGGTAAAAGTCCGCCTGATCATTTTCGACAAAGAATCGTCG
AACAAAGCGATCAAGGACATCAAAGAGCAAGAAGTCTACATGGGCGAAATCCCATTGATGACTGAGAACGGTACCTTCGTTATC
AACGGTACCGAGCGTGTTATCGTTTCCCAGCTGCACCGTTCCCCGGGCGTGTTCTTTGACCACGACCGCGGCAAGACGCACAGCTC
CGGCAAGCTCCTGTACTCCGCGCGGATCATTCCGTACCGTGGTTCTTGGTTGGACTTCGAGTTCGACCCTAAAGACTGCGTGTTCG
TGCGTATCGACCGTCGTCGCAAGCTGCCGGCCTCGGTACTGCTGCGCGCGCTCGGCTATACCACTGAGCAAGTGCTGGACGCCTTC
TACACCACCAACGTATTCAGCCTGAAGGATGAAACCCTCAAGCTGGAGCTGATCGCTTCGCGTCTGCGTGGTGAAATTGCCGTCC
TGGACATCCAGGATGAAAAAGGCAAGGTCATTGTTGAAGCTGGCCGTCGTATCACTGCGCGCCACATCAACCAGATCGAAAAAG
CCGGTATCAAAGAGCTGGAAGTGCCTCTGGACTACGTCCTGGGTCGCACTACCGCCAAGGTCATCGTTCACCCGGCTACAGGCGA
AATCCTGGCTGAGTGCAACACCGAGCTGAACACCGAGATCCTGGCCAAAATCGCCAAGGCTCAGGTTGTTCGCATCGAGACCCTG
TACACCAACGACATCGACTGCGGTCCGTTCATCTCCGACACTCTGAAGATCGACTCCACCAGCAACCAATTGGAAGCGCTGGTCG
AGATCTATCGCATGATGCGTCCTGGCGAGCCACCGACCAAAGACGCTGCCGAGACCCTGTTCAACAACCTGTTCTTCAGCCCTGA
GCGCTATGACCTGTCTGCGGTCGGCCGGATGAAGTTCAACCGTCGTATCGGTCGTACCGAGATCGAAGGTTCGGGCGTGTTGTGC
AAGGAAGACATCGTCGCGGTACTGAAGACTCTGGTCGACATCCGTAACGGTAAAGGCATCGTCGATGACATCGACCACCTGGGTA
ACCGTCGTGTTCGCTGCGTAGGCGAAATGGCCGAGAACCAGTTCCGCGTTGGCCTGGTGCGTGTTGAGCGTGCGGTCAAAGAGCG
TCTGTCGATGGCTGAAAGCGAAGGCCTGATGCCACAAGACCTGATCAACGCCAAGCCAGTGGCTGCGGCGGTGAAAGAGTTCTTC
GGCTCCAGCCAGCTCTCGCAGTTCATGGACCAGAACAACCCGCTCTCCGAGATCACCCACAAGCGCCGTGTTTCGGCACTGGGCC
CAGGCGGTCTGACCCGTGAGCGTGCAGGCTTTGAAGTTCGTGACGTACACCCGACGCACTACGGTCGTGTTTGCCCGATCGAAAC
GCCGGAAGGTCCGAACATCGGTCTGATCAACTCCCTGGCTGCGTATGCGCGCACCAACCAGTACGGCTTCCTCGAGAGCCCGTAC
CGCGTGGTGAAAGACGCTCTGGTCACCGACGAGATCGTGTTCCTGTCCGCCATCGAAGAAGCGGATCACGTGATCGCCCAGGCCT
CGGCCACGATGAACGACAAGAAAGTCCTGGTCGACGAGCTGGTAGCGGTTCGTCACTTGAACGAGTTCACCGTCAAGGCGCCGG
AAGACGTCACCTTGATGGACGTTTCGCCGAAGCAGGTAGTTTCGGTTGCAGCGTCGCTGATCCCGTTCCTCGAGCACGACGACGC
CAACCGTGCGTTGATGGGTTCCAACATGCAGCGTCAAGCTGTACCGACCCTGCGCGCTGACAAGCCGCTGGTAGGTACCGGCATG
GAGCGTAACGTAGCCCGTGACTCCGGCGTTTGCGTCGTGGCTCGTCGTGGCGGCGTGATCGACTCCGTTGATGCCAGCCGTATCGT
GGTCCGTGTTGCCGATGACGAAGTTGAAACTGGCGAAGCCGGTGTCGACATCTACAACCTGACCAAATACACCCGCTCGAACCAG
AACACCTGCATCAACCAGCGTCCGCTGGTGAGCAAGGGTGATCGCGTTCAGCGTAGCGACATCATGGCCGACGGCCCGTCCACCG
ACATGGGTGAACTGGCACTGGGCCAGAACATGCGCATCGCGTTCATGGCATGGAACGGCTTCAACTTCGAAGACTCCATCTGCCT
GTCCGAGCGTGTTGTTCAAGAAGACCGTTTCACCACGATCCACATTCAGGAACTGACCTGTGTGGCACGTGACACCAAGCTTGGG
CCAGAGGAAATCACTGCAGACATCCCGAACGTGGGTGAAGCTGCACTGAACAAGCTGGACGAAGCCGGTATCGTTTATGTAGGT
GCTGAAGTTGGCGCAGGCGACATCCTGGTAGGTAAGGTCACTCCGAAAGGCGAGACCCAACTGACTCCAGAGGAAAAACTGCTG
CGTGCGATCTTCGGTGAAAAAGCCAGCGACGTTAAAGACACCTCCCTGCGCGTACCTACCGGTACCAAGGGTACTGTCATCGACG
TACAGGTCTTCACCCGTGACGGCGTTGAGCGTGATGCTCGTGCACTGTCCATCGAGAAGACCCAGCTCGACGAGATCCGCAAGGA
TCTGAACGAAGAGTTCCGTATCGTTGAAGGCGCGACCTTCGAACGTCTGCGTTCCGCCCTGGTAGGCCACAAGGCTGAAGGCGGC
GCAGGTCTGAAGAAAGGTCAGGACATCACCGACGAAGTTCTCGACGGTCTTGAGCACGGCCAGTGGTTCAAACTGCGCATGGCTG
AAGATGCTCTGAACGAGCAGCTCGAGAAGGCCCAGGCCTATATCGTTGATCGTCGCCGTCTGCTGGACGACAAGTTCGAAGACAA
GAAGCGCAAACTGCAGCAAGGCGATGACCTGGCTCCAGGCGTGCTGAAAATCGTCAAGGTTTACCTGGCAATCCGTCGTCGCATC
CAACCGGGCGACAAGATGGCCGGTCGTCACGGTAACAAAGGTGTGGTCTCCGTGATCATGCCGGTTGAAGACATGCCGCACGAT
GCCAATGGCACACCGGTCGACGTCGTCCTCAACCCGTTGGGCGTACCTTCGCGTATGAACGTTGGTCAGATCCTTGAAACCCACCT
GGGCCTCGCGGCCAAAGGTCTGGGCGAGAAGATCAACCGTATGATCGAAGAGCAGCGCAAGGTCGCAGACCTGCGTAAGTTCCT
GCACGAGATCTACAACGAGATCGGCGGTCGCAACGAAGAGCTGGATACCTTCTCCGACCAGGAAATCCTGGATCTGGCGAAGAA
CCTGCGCGGCGGCGTTCCAATGGCTACCCCGGTGTTCGACGGTGCCAAGGAAAGCGAAATCAAGGCCATGCTGAAACTGGCAGA
CCTGCCAGAAAGCGGCCAGATGCAGCTGTTCGACGGCCGTACCGGCAACAAGTTCGAGCGCCCGGTTACTGTTGGCTACATGTAC
ATGCTGAAGCTGAACCACTTGGTAGACGACAAGATGCACGCTCGTTCTACCGGTTCGTACAGCCTCGTTACCCAGCAGCCGCTGG
GTGGTAAGGCTCAGTTCGGTGGTCAGCGTTTCGGGGAGATGGAGGTCTGGGCACTGGAAGCATACGGTGCTGCTTACACTCTGCA
AGAAATGCTCACAGTGAAGTCGGACGATGTGAACGGTCGGACCAAGATGTACAAAAACATCGTGGACGGCGATCACCGTATGGA
GCCGGGCATGCCCGAGTCCTTCAACGTGTTGATCAAAGAAATTCGTTCCCTCGGCATCGATATCGATCTGGAAACCGAATAA

P. fluorescens G20-18_GCF_018502625.1_CP075566.1

ATGGCTTACTCATATACTGAGAAAAAACGTATCCGCAAGGACTTTAGCAAGTTGCCGGACGTCATGGATGTGCCGTACCTCCTGG
CCATCCAGCTGGATTCGTATCGTGAATTCTTGCAAGCGGGAGCGACTAAAGATCAGTTCCGCGACGTGGGCCTGCATGCGGCCTT
CAAATCCGTTTTCCCGATCATCAGCTACTCCGGCAATGCTGCGCTGGAGTACGTCGGTTATCGCCTGGGCGAACCGGCATTTGATG
TCAAAGAATGCGTATTGCGCGGTGTAACTTACGCCGTACCTTTGCGGGTAAAAGTGCGCCTGATCATTTTCGACAAAGAATCGTCG
AACAAAGCGATCAAGGACATCAAAGAGCAAGAAGTCTACATGGGTGAAATCCCCCTGATGACTGAGAACGGTACCTTCGTAATC
AACGGTACCGAGCGTGTAATCGTTTCCCAGCTGCACCGTTCGCCGGGCGTGTTCTTCGACCACGACCGTGGCAAGACGCACAGCT
CCGGCAAACTGCTGTACTCCGCGCGAATCATTCCTTACCGCGGTTCGTGGCTGGACTTCGAGTTCGACCCGAAAGACTGCGTGTTC
GTGCGTATCGACCGTCGTCGCAAGCTGCCTGCATCGGTACTGCTGCGCGCGCTCGGCTATACCACTGAAGAAGTGCTCGACGCGT
TCTACACCACCAACGTCTTCCACGTGCAAGGTGAAAACCTCAGCCTGGAACTGGTGCCTCAGCGCCTGCGTGGTGAAATTGCTGT
CCTGGATATCCTGGATGACAAAGGCAAGGTTATTGTCGAGCAAGGTCGTCGTATCACTGCTCGCCACATCAACCAGCTGGAAAAA
GCAGGGATCAAAGAGCTGCAAGTGCCTCTGGACTACGTCCTGGGTCGCACTACCGCCAAGGTCATCGTGCATCCGGCAACCGGCG
AAATCCTGGCAGAGTGCAACACCGAGCTGAACACCGAGATCCTGGCGAAAATCGCCAAGGCCGGCGTTGTTCGCATCGAAACTCT
GTACACCAACGATATCGACTGCGGTCCGTTCGTCTCCGACACGCTGAAGATCGACTCCACCAGCAACCAATTGGAAGCGCTGGTC
GAGATCTATCGCATGATGCGTCCAGGCGAGCCGCCAACCAAAGACGCTGCCGAGACCCTGTTCAACAACCTGTTCTTCAGCCCTG
AGCGCTATGATCTGTCTGCGGTCGGCCGGATGAAGTTCAACCGTCGTATCGGTCGTACCGAGATCGAAGGTTCGGGCGTGTTGTG
CAAGGAAGACATCGTCGCGGTACTGAAGACTCTTGTCGACATCCGTAACGGTAAAGGCATCGTCGATGACATCGACCACCTGGGT

AACCGTCGTGTTCGCTGTGTAGGCGAAATGGCCGAGAACCAGTTCCGCGTTGGCCTGGTACGTGTTGAGCGTGCGGTCAAAGAGC
GTCTGTCGATGGCTGAAAGCGAAGGCCTGATGCCGCAAGACCTGATCAACGCCAAGCCAGTGGCTGCGGCGGTGAAAGAGTTCTT
CGGTTCCAGCCAGCTGTCCCAGTTCATGGGCCAGAACAACCCGCTGTCCGAGATCACCCACAAGCGTCGTGTGTCTGCACTCGGC
CCTGGCGGTCTGACTCGTGAGCGTGCCGGCTTTGAAGTTCGTGACGTACACCCGACTCACTACGGTCGTGTATGCCCGATTGAAAC
GCCGGAAGGTCCAAACATCGGTCTGATCAACTCCCTGGCCGCCTATGCGCGCACCAATCAGTACGGCTTCCTCGAAAGCCCGTAC
CGCGTGGTGAAAGACGCTCTGGTCACCGACGAGATCGTGTTCCTGTCCGCCATCGAAGAAGCTGATCACGTGATCGCTCAGGCCT
CGGCCACGATGAACGACAAGAAAGTCCTGATCGACGAACTGGTAGCTGTTCGTCACTTGAACGAGTTCACCGTCAAGGCGCCGGA
AGACGTCACCTTGATGGACGTATCGCCAAAACAGGTAGTTTCGGTCGCAGCGTCGCTGATCCCGTTCCTCGAGCACGACGACGCC
AACCGTGCGTTGATGGGTTCGAACATGCAGCGTCAAGCTGTACCAACCCTGCGCGCTGACAAGCCGCTGGTCGGTACCGGCATGG
AGCGTAACGTAGCCCGTGACTCCGGCGTTTGCGTCGTGGCTCGTCGTGGTGGCGTTATCGATTCCGTCGACGCCAGCCGTATCGTG
GTTCGTGTTGCTGATGACGAAGTTGAAACCGGCGAAGCTGGTGTCGACATCTACAACCTGACCAAGTACACCCGCTCCAACCAGA
ACACCTGCATCAACCAGCGTCCGCTGGTGCGTAAAGGTGATCGGGTTCAGCGCAGCGACATCATGGCCGACGGTCCGTCCACCGA
CATGGGTGAGCTGGCTCTGGGTCAGAACATGCGCATCGCGTTCATGGCATGGAACGGCTTCAACTTCGAAGACTCCATCTGCCTGT
CCGAGCGTGTGGTTCAGGAAGATCGCTTCACCACGATCCACATCCAGGAACTGACCTGTGTGGCACGTGACACCAAGCTTGGGCC
AGAGGAAATCACTGCAGACATCCCGAACGTGGGTGAAGCTGCACTGAACAAACTGGACGAAGCCGGTATCGTTTACGTAGGTGC
TGAAGTAGGCGCAGGCGACATCCTGGTTGGTAAGGTCACTCCGAAAGGCGAGACCCAACTGACGCCGGAAGAAAAACTGCTGCG
TGCCATCTTCGGTGAAAAAGCCAGCGACGTTAAAGACACCTCCCTGCGTGTGCCTACCGGCACCAAAGGTACTGTCATCGACGTA
CAGGTCTTCACCCGCGACGGCGTTGAGCGTGATGCTCGTGCACTGTCGATCGAGAAGACTCAACTCGACGAGATCCGCAAGGACC
TGAACGAAGAGTTCCGTATCGTTGAAGGCGCCACTTTCGAACGTCTGCGTTCCGCTCTGGTCGGCCACAAAGCCGAAGGCGGCGC
CGGTCTGAAGAAAGGTCAGGAAATCACCGACGAAGTTCTCGACGGTCTTGAGCATGGTCAGTGGTTCAAACTGCGCATGGCTGAA
GATGCTCTGAACGAGCAACTCGAGAAGGCTCAGGCCTACATCGTTGATCGCCGCCGTCTGCTGGACGACAAGTTCGAAGACAAGA
AGCGCAAACTGCAGCAGGGCGATGACCTGGCTCCAGGCGTGCTGAAAATCGTCAAGGTTTACCTGGCAATCCGTCGCCGCATCCA
GCCGGGCGACAAGATGGCCGGTCGTCACGGTAACAAAGGTGTGGTCTCCGTGATCATGCCGGTTGAAGACATGCCGCACGATGCC
AATGGCACCCCGGTCGACGTCGTCCTCAACCCGTTGGGCGTACCTTCGCGTATGAACGTTGGTCAGATCCTTGAAACCCACCTCGG
CCTCGCGGCTAAAGGTCTGGGTGAGAAGATCAACCGGATGATCGAAGAGCAGCGCAAAGTCGCCGAGCTTCGCAAGTTCCTGCA
CGAGATCTACAACGAGATCGGCGGTCGTAACGAAGATCTGGATAGCTTCTCCGACCAGGAAATCCTGGATCTGGCGAAGAACCTG
CGTGGCGGCGTTCCAATGGCCACTCCAGTGTTCGACGGTGCCAAGGAAAGCGAAATCAAGGCCATGCTGAAACTGGCAGACCTG
CCAGAAAGCGGCCAGATGCAGCTGACCGACGGCCGTACCGGCAACAAGTTCGAGCGCCCGGTTACTGTTGGCTACATGTACATGC
TGAAGCTGAACCACTTGGTAGACGACAAGATGCACGCTCGTTCTACCGGTTCGTACAGCCTGGTTACCCAGCAGCCGCTGGGTGG
TAAGGCGCAGTTCGGTGGTCAGCGTTTCGGGGAGATGGAGGTCTGGGCACTGGAAGCATACGGTGCTGCTTACACTCTGCAAGAA
ATGCTCACAGTGAAGTCGGACGATGTGAACGGCCGTACCAAGATGTACAAAAACATCGTGGATGGCGATCACCGTATGGAGCCG
GGCATGCCCGAGTCCTTCAACGTGTTGATCAAGGAAATTCGTTCCCTCGGCATCGATATCGATCTGGAAACCGAATAA

P. fluorescens NCTC10038_GCF_900475215.1_LS483372.1

ATGGCTTACTCATATACTGAGAAAAAACGTATCCGCAAGGACTTTAGCAAGTTGCCGGACGTCATGGATGTCCCGTACCTTCTGGC
TATCCAGCTGGATTCGTATCGTGAATTCTTGCAAGCGGGAGCGACTAAAGATCAGTTCCGCGACGTGGGCCTGCATGCGGCCTTC
AAATCCGTTTTCCCGATCATCAGCTACTCCGGCAATGCTGCGCTGGAGTACGTGGGTTATCGCCTGGGCGAACCGGCATTTGATGT
CAAAGAATGCGTGTTGCGCGGTGTTACGTACGCCGTACCTTTGCGGGTAAAAGTCCGTCTGATCATTTTCGACAAAGAATCGTCGA
ACAAAGCGATCAAGGACATCAAAGAGCAAGAAGTCTACATGGGCGAAATCCCATTGATGACTGAAAACGGTACCTTCGTTATCA
ACGGTACCGAGCGCGTTATCGTTTCCCAGCTGCACCGTTCCCCGGGCGTGTTCTTCGACCACGACCGCGGCAAGACGCACAGCTC
CGGTAAGCTCCTGTACTCCGCGCGGATCATTCCGTACCGCGGCTCGTGGTTGGACTTCGAGTTCGACCCGAAAGACTGCGTGTTCG
TGCGTATCGACCGTCGTCGTAAGCTGCCGGCCTCGGTACTGCTGCGCGCGCTCGGCTATACCACTGAGCAAGTGCTTGATGCTTTC
TACACCACCAACGTATTCAGCCTGAAGGATGAAACCCTCAGCCTGGAACTGATTGCTTCGCGTCTGCGTGGTGAAATTGCCGTCCT
GGATATCCAGGATGAAAACGGCAAGGTCATCGTTGAAGCTGGCCGCCGTATTACCGCGCGCCACATCAACCAGATCGAAAAAGC
CGGTATCAAGTCGCTGGATGTGCCGCTGGACTACGTCCTGGGTCGCACCACTGCCAAGGTCATCGTTCACCCGGCTACAGGCGAA
ATCCTGGCTGAGTGCAACACCGAGCTGAACACCGAGATCCTGGCAAAAATCGCCAAGGCCCAGGTTGTTCGCATCGAGACCCTGT
ACACCAACGACATCGACTGCGGTCCGTTCATCTCCGACACGCTGAAGATCGACTCCACCAGCAACCAATTGGAAGCGCTGGTTGA
GATCTATCGCATGATGCGTCCTGGTGAGCCACCGACCAAAGACGCTGCCGAGACCCTGTTCAACAACCTGTTCTTCAGCCCTGAG
CGCTATGACCTGTCTGCGGTCGGCCGGATGAAGTTCAACCGTCGTATCGGTCGTACCGAGATCGAAGGTTCGGGCGTGCTGTGCA
AGGAAGACATCGTCGCGGTACTGAAGACCTTGGTCGACATCCGTAACGGTAAAGGCATCGTCGATGACATCGACCACTTGGGTAA
CCGTCGTGTTCGCTGCGTAGGCGAAATGGCCGAGAACCAGTTCCGCGTTGGCCTGGTACGTGTTGAGCGTGCGGTCAAAGAGCGT
CTGTCGATGGCTGAAAGCGAAGGCCTGATGCCGCAAGATCTGATCAACGCCAAGCCAGTGGCTGCGGCGGTGAAAGAGTTCTTCG
GTTCCAGCCAGCTCTCGCAGTTCATGGACCAGAACAACCCGCTCTCCGAGATCACCCACAAGCGCCGTGTTTCCGCACTGGGCCC
GGGCGGTCTGACCCGTGAGCGTGCAGGCTTTGAAGTTCGTGACGTACACCCAACGCACTACGGTCGTGTTTGCCCGATCGAAACG
CCGGAAGGTCCGAACATCGGTCTGATCAACTCCCTGGCCGCTTATGCACGCACTAACCAGTACGGCTTCCTCGAGAGCCCGTACC
GTGTAGTGAAAGATGCACTGGTCACCGACGAGATCGTGTTCCTGTCCGCCATCGAAGAAGCCGATCACGTGATCGCTCAGGCTTC
GGCCACGATGAACGACAAGAAAGTCCTGATCGACGAGCTGGTAGCTGTTCGTCACTTGAACGAGTTCACCGTTAAGGCGCCGGAA
GACGTCACCTTGATGGACGTTTCGCCGAAGCAGGTAGTTTCGGTTGCAGCGTCGCTGATCCCGTTCCTGGAGCACGATGACGCCA
ACCGTGCGTTGATGGGTTCCAACATGCAGCGTCAAGCTGTACCCACCCTGCGTGCCGACAAGCCGCTGGTAGGTACCGGCATGGA
GCGTAACGTAGCCCGTGACTCCGGCGTTTGCGTCGTGGCTCGTCGTGGCGGCGTGATCGACTCTGTTGATGCCAGCCGTATCGTGG
TTCGTGTTGCCGATGACGAAGTTGAGACTGGCGAAGCCGGTGTCGACATCTACAACCTGACCAAATACACCCGCTCGAACCAGAA
CACCTGCATCAACCAGCGCCCGCTGGTGAGCAAGGGTGATCGCGTTCAGCGTAGCGACATCATGGCCGACGGCCCGTCCACCGAT
ATGGGTGAGCTGGCACTGGGTCAGAACATGCGCATCGCGTTCATGGCATGGAACGGCTTCAACTTCGAAGACTCCATCTGCCTGT
CCGAGCGTGTTGTTCAAGAAGACCGCTTCACCACGATCCACATTCAGGAACTGACCTGTGTGGCGCGTGACACCAAGCTTGGGCC

AGAGGAAATCACTGCAGACATCCCGAACGTGGGTGAAGCTGCACTGAACAAACTGGACGAAGCCGGTATCGTTTACGTAGGTGC
TGAAGTTGGCGCAGGCGACATCCTGGTTGGTAAGGTCACTCCGAAAGGCGAGACCCAACTGACTCCGGAAGAGAAGCTGTTGCGT
GCCATCTTCGGTGAGAAAGCCAGCGACGTTAAAGACACTTCCCTGCGCGTACCTACCGGTACCAAGGGTACTGTCATCGACGTAC
AGGTCTTCACCCGTGACGGCGTTGAGCGTGATGCTCGTGCACTGTCCATCGAGAAGACTCAACTCGACGAGATCCGCAAGGACCT
GAACGAAGAGTTCCGTATCGTTGAAGGCGCGACCTTCGAACGTCTGCGTTCCGCTCTGGTAGGCCACAAGGCTGAAGGCGGCGCA
GGTCTGAAGAAAGGTCAGGACATCACCGACGAAATCCTCGACGGTCTTGAGCACGGCCAGTGGTTCAAACTGCGCATGGCTGAA
GACGCTCTGAACGAGCAGCTCGAGAAGGCCCAGGCCTATATCGTTGATCGCCGCCGTCTGCTGGACGACAAGTTCGAAGACAAG
AAGCGCAAACTGCAGCAGGGCGATGACCTGGCTCCAGGCGTGCTGAAAATCGTCAAGGTTTACCTGGCAATCCGTCGCCGCATTC
AGCCGGGCGACAAGATGGCCGGTCGTCACGGTAACAAGGGTGTGGTCTCCGTGATCATGCCGGTTGAAGACATGCCGCACGATG
CCAATGGCACCCCGGTCGACGTCGTCCTCAACCCGTTGGGCGTACCTTCGCGTATGAACGTTGGTCAGATCCTTGAAACCCACCTG
GGCCTCGCGGCCAAAGGTCTGGGCGAGAAGATCAACCGTATGATCGAAGAGCAGCGCAAGGTCGCAGACCTGCGTAAGTTCCTG
CACGAGATCTACAACGAGATCGGCGGTCGCAACGAAGAGTTGGACACTTTCTCCGACCAGGAAATCCTGGATCTGGCGAAGAAC
CTGCGCGGCGGCGTTCCAATGGCTACCCCGGTATTCGACGGTGCCAAGGAAAGCGAAATCAAGGCCATGCTGAAACTGGCAGAC
CTGCCGGAAAGTGGCCAGATGCAGCTGTTCGACGGCCGTACCGGCAACAAGTTTGAGCGCCCGGTTACTGTTGGCTACATGTACA
TGCTGAAGCTGAACCACTTGGTAGACGACAAGATGCACGCTCGTTCTACCGGTTCGTACAGCCTGGTTACCCAGCAGCCGCTGGG
TGGTAAGGCTCAGTTCGGTGGTCAGCGTTTCGGGGAGATGGAGGTCTGGGCACTGGAAGCATACGGTGCTGCTTACACTCTGCAA
GAAATGCTCACAGTGAAGTCGGACGATGTGAACGGTCGGACCAAGATGTACAAAAACATCGTGGACGGCGATCACCGTATGGAG
CCGGGCATGCCCGAGTCCTTCAACGTGTTGATCAAAGAAATTCGTTCCCTCGGCATCGATATCGATCTGGAAACCGAATAA

P. fluorescens NCTC10783_GCF_900636635.1_LR134300.1

ATGGCTTACTCATACACTGAGAAAAAACGTATCCGCAAGGACTTTAGCAAGTTGCCGGACGTCATGGATGTGCCGTATTTGCTGG
CCATCCAGCTGGATTCCTATCGCGAATTCCTGCAGGCTGGCGCAACCAAGGAGCAGTTCCGCGATATCGGTCTGCACGCGGCCTT
CAAGTCCGTTTTCCCGATTATCAGCTATTCCGGCAATGCTGCCCTGGAATACGTCGGCTACCGTCTGGGTGAGCCGGCATTCGATG
TCAAGGAGTGCGTGCTGCGCGGCGTGACCTTCGCCGTACCGCTGCGCGTGAAAGTTCGCCTGATCATCTTCGACCGCGAGTCGTC
GAACAAGGCGATCAAGGACATCAAGGAACAAGAAGTCTACATGGGGGAAATCCCCCTGATGACCGAGAACGGTACCTTCATCAT
CAACGGTACCGAACGTGTCATCGTCTCCCAGCTGCACCGTTCCCCGGGCGTGTTCTTCGACCACGACCGTGGCAAGACCCACAGC
TCCGGCAAGCTGCTGTACTCCGCGCGGATCATTCCTTACCGCGGTTCCTGGCTGGACTTCGAGTTCGATCCGAAGGACTGCGTGTT
CGTCCGTATCGACCGTCGCCGCAAGCTGCCGGCCTCGGTACTGCTGCGCGCGCTCGGCTACAGCACCGAAGAGATCCTCAACGCC
TTCTACGCGACCAACGTCTTCCACATCAAGGGCGAGACCCTGAACCTGGAACTGGTCCCGCAGCGCCTGCGCGGTGAAGTCGCGA
GCATCGACATCAAGGATGGCAGCGGCAAGGTGATCGTGGAGCAGGGGCGTCGTATCACTGCCCGCCACATCAACCAGCTGGAAA
AGGCTGGCGTGAGTCAGCTGGAAGTGCCGTTCGACTACCTGATCGGTCGTACCATCGCCAAGGCGATCGTGCATCCGGCTACCGG
CGAGATCATCGCCGAGTGCAACACCGAGCTGACCCTCGACCTCCTGGCCAAGGTGGCCAAGGCCCAGGTCGTGCGCATCGAGACC
CTGTACACCAACGACATCGACTGCGGTCCGTTCATCTCCGACACCCTGAAGATCGACAACACCAGCAACCAGCTGGAAGCCCTGG
TCGAGATCTACCGGATGATGCGTCCGGGCGAGCCGCCGACCAAGGAAGCTGCCGAGACCCTGTTCGGCAACCTGTTCTTCAGCGC
CGAGCGTTACGACCTGTCGGCCGTAGGCCGGATGAAGTTCAACCGCCGTATCGGTCGTACCGAGATCGAAGGTCCGGGCGTCCTG
AGCAAGGAAGACATCATCGATGTGCTCAAGACCCTCGTCGACATCCGTAACGGCAAGGGCATCGTCGATGACATCGACCACCTGG
GCAACCGTCGCGTCCGTTGCGTCGGCGAAATGGCCGAGAACCAGTTCCGCGTGGGCCTGGTGCGTGTCGAGCGCGCGGTCAAGGA
ACGCCTGTCCATGGCCGAAAGCGAAGGCCTGATGCCGCAGGACCTGATCAACGCCAAGCCGGTGGCTGCCGCGATCAAGGAGTT
CTTCGGTTCGAGCCAGCTGTCGCAGTTCATGGACCAGAACAACCCGCTTTCCGAGATCACCCACAAGCGCCGCGTCTCCGCGCTC
GGCCCGGGCGGTCTGACCCGTGAGCGTGCGGGCTTCGAGGTTCGTGACGTACACCCGACCCACTACGGCCGCGTGTGCCCGATCG
AAACCCCTGAAGGTCCGAACATCGGTCTGATCAACTCCCTGGCGACCTACGCCCGCACCAACAAGTACGGCTTCCTCGAGAGCCC
GTACCGCGTGGTCAAGGACAGCCTGGTAACCGACGAGATCGTGTTCCTGTCGGCGATCGAAGAAGCCGACCACGTCATCGCCCAG
GCTTCGGCGACCCTCAACGAGAAGGGTCAACTGGTGGACGAGCTGGTGGCCGTGCGTCACCTGAACGAATTCACCGTGAAGGCG
CCGGAAGACGTGACCCTGATGGACGTGTCGCCGAAGCAGGTCGTTTCCGTCGCTGCCTCGCTGATTCCGTTCCTCGAGCACGATG
ACGCCAACCGCGCACTCATGGGCTCGAACATGCAGCGTCAGGCCGTGCCGACCCTGCGTGCCGACAAGCCGCTGGTGGGTACCGG
CATGGAGCGCAACGTGGCGCGCGACTCCGGCGTCTGCGTCGTGGCTCGCCGTGGCGGTGTGATCGACTCGGTCGATGCCAGCCGT
GTCGTGGTTCGCGTGGCGGATGACGAAGTCGAGACCGGCGAAGCGGGTGTCGACATCTACAACCTGACCAAGTACACCCGTTCCA
ACCAGAACACCTGCATCAACCAGCGTCCGCTGGTGAGCAAGGGTGACGTGGTCGCGCGCGGCGACATCCTGGCCGACGGTCCGTC
CACCGACATGGGCGAACTGGCCCTGGGCCAGAACATGCGCGTAGCGTTCATGCCCTGGAACGGCTTCAACTTCGAAGACTCCATC
TGCCTGTCCGAGCGCGTGGTCCAGGAAGATCGTTTCACCACGATCCACATCCAGGAACTGACCTGCGTCGCTCGTGACACCAAGC
TTGGCCCAGAGGAAATCACCGCGGACATCCCGAACGTGGGCGAGGCCGCGCTGAACAAGCTGGACGAAGCCGGTATCGTCTACG
TCGGCGCCGAAGTGCAGGCCGGCGACATCCTGGTCGGCAAGGTCACTCCGAAAGGCGAGACCCAGTTGACTCCGGAAGAGAAGC
TGCTGCGCGCGATCTTCGGTGAGAAGGCGTCCGACGTGAAGGACACCTCCCTGCGTGTGCCGACCGGCACCAAGGGTACCGTCAT
CGACGTTCAGGTCTTCACCCGCGACGGCGTCGAGCGCGACTCCCGCGCGCTGTCCATCGAGAAGATGCAACTCGACCAGATCCGC
AAGGACCTGAACGAAGAGTTCCGCATCGTCGAAGGCGCGACCTTCGAGCGTCTGCGTGCCGCCCTGGTCGGTGCCAAGGCTGAAG
GTGGCCCGGCGCTGAAGAAGGGTACGGAGATCACCGACGACTACCTCGACGGTCTCGATCGCGGCCAGTGGTTCAAGCTGCGCAT
GGCCGACGACGCCCTGAACGAACAGCTGGAGAAGGCCCAGGCCTACATCAGCGATCGTCGCCAGCTCCTGGACGACAAGTTCGA
GGACAAGAAGCGCAAGCTGCAGCAGGGCGACGACCTGGCTCCGGGCGTGCTGAAGATCGTCAAGGTCTACCTGGCGATCAAGCG
TCGCATCCAGCCGGGCGACAAGATGGCCGGCCGTCACGGTAACAAGGGTGTGGTCTCGGTGATCATGCCGGTGGAAGACATGCC
GCACGATGCCAACGGCACCCCGGTCGACATCGTCCTCAACCCGCTGGGCGTACCGTCGCGTATGAACGTCGGTCAGATCCTCGAA
ACCCACCTGGGCCTCGCGGCCAAGGGGCTGGGCGAGAAGATCAACCGCATGCTCGAAGAGCAGCGCAAGGTCGCCGAACTGCGT
AAGTTCCTGCACGAGATCTACAACGAGATCGGCGGTCGCGAGGAAAACCTCGACGAGCTGGGCGACAACGAGATCCTCGCGCTG
GCCAAGAACCTGCGCGGTGGCGTACCGATGGCGACCCCGGTGTTCGATGGCGCCAAGGAACGCGAGATCAAGGCCATGCTGAAG
CTGGCCGACCTGCCGGAAAGCGGCCAGATGCGTCTGTTCGACGGCCGTACCGGCAACCAGTTCGAGCGTCCGACCACCGTCGGCT

ACATGTACATGCTCAAGCTGAACCACCTGGTGGACGACAAGATGCACGCCCGTTCCACCGGCTCGTACAGCCTGGTTACCCAGCA
GCCGCTGGGTGGTAAGGCACAGTTCGGTGGTCAGCGCTTCGGTGAGATGGAGGTGTGGGCGCTGGAAGCCTATGGCGCGGCGTAC
ACCCTGCAGGAAATGCTGACGGTCAAGTCGGACGACGTGAACGGCCGGACCAAGATGTACAAGAACATCGTGGACGGCGATCAC
CGCATGGAGGCCGGCATGCCCGAGTCCTTCAACGTTCTGATCAAAGAGATCCGTTCGCTCGGCATCGACATCGAACTGGAAACCG
AATAA

P. fluorescens NCTC9428_GCF_900636825.1_LR134318.1

ATGGCTTACTCATATACTGAGAAAAAACGTATCCGCAAGGACTTTAGCAAGTTGCCGGACGTCATGGATGTGCCGTACCTCCTGG
CCATCCAGCTGGATTCGTATCGTGAATTCTTGCAAGCGGGAGCGACTAAAGATCAGTTCCGCGACGTGGGCCTGCATGCGGCCTT
CAAATCCGTTTTCCCGATCATCAGCTACTCCGGCAATGCTGCGCTGGAGTACGTCGGTTATCGCCTGGGCGAACCGGCATTTGATG
TCAAAGAATGCGTATTGCGCGGTGTAACTTTCGCCGTACCTTTGCGGGTAAAAGTGCGCCTGATCATTTTCGACAAAGAATCGTCG
AACAAAGCGATCAAGGACATCAAAGAGCAAGAAGTCTACATGGGTGAAATTCCCCTGATGACTGAGAACGGTACCTTTGTAATC
AACGGTACCGAGCGTGTAATCGTTTCCCAGCTGCACCGTTCCCCGGGCGTGTTCTTCGATCACGACCGCGGCAAGACGCATAGCT
CCGGCAAACTGTTGTACTCCGCGCGGATCATTCCTTACCGCGGTTCGTGGTTGGATTTCGAGTTCGACCCGAAAGACTGCGTATTT
GTACGTATCGACCGTCGTCGCAAGCTGCCGGCATCGGTACTGCTGCGCGCGCTCGGCTACACCACTGAAGAAGTGCTGGACGCTT
TCTACACCACCAACGTTTTCCACCTGAGCGGCGAAACCCTCAGCCTGGAACTGATTGCTTCGCGTCTGCGTGGTGAAATTGCGGTT
CTGGATATTCAGGACGAGAAGGGCAAGGTCATCGTTGAAGCGGGCCGCCGTATTACCGCGCGCCACATCAACCAGATCGAAAAA
GCCGGTATCAAGACTCTGGACGTGCCACTGGATTACGTCCTCGGCCGCACTACCGCCAAGGTCATCGTGCACCCGGCTACTGGCG
AGATCCTGGCAGAGTGCAACACCGAGCTGAACACTGAAGTCCTGGCCAAAATTGCCAAGTCGGGCGTTGTTCGCATCGAAACTCT
GTACACCAACGACATCGACTGCGGTCCGTTTATCTCCGACACGCTGAAGATCGACTCCACCAGCAACCAATTGGAAGCGCTGGTC
GAGATCTATCGCATGATGCGTCCAGGCGAGCCGCCAACCAAAGACGCTGCCGAAACCCTGTTCAACAACCTGTTCTTCAGCCCTG
AGCGTTATGACCTGTCTGCGGTCGGCCGGATGAAGTTCAACCGTCGTATCGGTCGTACCGAGATCGAAGGTTCGGGCGTGCTGTG
CAAGGAAGATATCGTCGCGGTACTGAAGACTCTGGTCGACATCCGTAACGGTAAAGGCATCGTCGATGACATCGACCACCTGGGT
AACCGTCGTGTTCGCTGCGTAGGCGAAATGGCCGAGAACCAGTTCCGCGTTGGCCTGGTGCGTGTTGAGCGTGCGGTCAAAGAGC
GTCTGTCGATGGCAGAAAGCGAAGGCCTGATGCCGCAAGACCTGATCAACGCCAAGCCAGTGGCTGCGGCGGTGAAAGAGTTCT
TCGGTTCCAGCCAGCTCTCGCAGTTCATGGACCAGAACAACCCGCTGTCCGAGATCACTCACAAGCGTCGTGTTTCTGCACTCGGC
CCTGGCGGTCTGACTCGTGAGCGCGCAGGCTTTGAAGTCCGTGACGTACACCCGACTCACTACGGTCGTGTATGCCCGATCGAAA
CGCCGGAAGGTCCGAACATCGGTCTGATCAACTCCCTGGCTGCTTACGCTCGCACCAACCAGTACGGCTTCCTGGAAAGCCCGTA
CCGCGTGGTGAAAGAGGGTGTGGTCACCGATGAGATCGTTTTCCTGTCCGCTATTGAAGAAGCCGATCATGTGATCGCGCAGGCT
TCGGCGACCATGAACGAACAGAAAGTCCTGATCGACGAGCTGGTAGCCGTACGTCACCTGAACGAGTTCACCGTCAAGGCGCCTG
AAGAAGTCACCCTGATGGACGTTTCGCCGAAGCAGGTAGTTTCGGTTGCGGCGTCGCTGATTCCGTTCCTCGAGCACGACGACGC
CAACCGTGCGTTGATGGGTTCGAACATGCAGCGTCAAGCTGTACCAACCCTGCGCGCTGACAAGCCGCTGGTAGGTACCGGCATG
GAGCGTAACGTAGCCCGTGACTCCGGCGTTTGCGTCGTGGCTCGTCGTGGCGGCGTGATCGACTCCGTCGACGCCAGCCGTATCG
TGGTTCGCGTTGCTGATGATGAAGTTGAAACCGGTGAAGCCGGTGTCGACATCTATAACCTGACCAAGTACACCCGTTCGAACCA
GAACACCTGCATCAACCAGCGTCCGCTGGTGCGTAAAGGCGATCGCGTTCAGCGTAGCGACATCATGGCCGACGGCCCGTCCACC
GACATGGGTGAACTGGCACTGGGTCAGAACATGCGCATCGCGTTCATGGCATGGAACGGCTTCAACTTCGAAGACTCCATCTGCC
TGTCCGAGCGTGTAGTTCAGGAAGACCGCTTCACCACGATCCACATTCAGGAACTGACCTGTGTGGCCCGTGACACCAAGCTTGG
CCCAGAGGAAATCACTGCGGACATCCCGAACGTGGGTGAAGCCGCACTGAACAAGCTGGACGAAGCCGGTATCGTTTACGTAGG
TGCTGAAGTAGGCGCAGGCGACATCCTGGTCGGCAAGGTCACTCCGAAAGGCGAGACCCAACTGACTCCGGAAGAAAAACTGCT
GCGTGCAATCTTCGGTGAGAAAGCCAGCGACGTTAAAGACACTTCCCTGCGCGTGCCAACCGGCACCAAAGGTACTGTCATTGAC
GTGCAGGTCTTCACTCGTGACGGCGTTGAGCGTGACGCTCGTGCTCTCTCGATCGAGAAGTCCCAGCTGGACGAGATCCGCAAGG
ATCTGAACGAAGAGTTCCGTATCGTTGAAGGCGCTACTTTCGAACGTCTGCGTTCCGCTCTGGTAGGCCACAAGGCCGAAGGCGG
CGCCGGTCTGAAGAAAGGTCAGGACATCACCGACGAAGTACTCGACGGTCTCGAGCACGGCCAGTGGTTCAAACTGCGCATGGC
TGAAGACGCTCTGAACGAGCAGCTCGAGAAGGCTCAGGCCTACATCGTTGATCGTCGCCGTCTGCTGGACGACAAGTTCGAAGAC
AAGAAGCGCAAACTGCAGCAGGGCGATGACCTGGCTCCAGGCGTGCTGAAAATCGTCAAGGTTTACCTGGCAATCCGTCGCCGC
ATCCAGCCGGGCGACAAGATGGCCGGTCGTCACGGTAACAAAGGTGTGGTCTCGGTGATCATGCCGGTTGAAGACATGCCGCAC
GACGCCAATGGCACCCCGGTCGACGTGGTCCTCAACCCGCTGGGCGTACCTTCGCGTATGAACGTTGGTCAGATCCTCGAAACTC
ACCTGGGCCTCGCGGCTAAAGGTCTGGGCGAGAAGATCAACCGGATGGTCGAAGAGCAGCGTAAAGTCGCTGAACTGCGTACTTT
CCTGGACGAGATCTACAACCAGATCGGTGGTCGCAACGAAGATCTGGATAGCTTCTCCGATCAGGAAATCCTGGATCTGGCGAAT
AACCTGCGTGGCGGCGTTCCAATGGCCACTCCAGTGTTCGACGGCGCCAAGGAAAGCGAAATCAAGGCCATGCTGAAACTGGCA
GACCTGCCGGAAAGTGGCCAGATGCAGCTGACCGACGGCCGTACCGGCAACAAGTTCGAGCGTCCAGTTACCGTTGGCTACATGT
ACATGCTGAAGCTGAACCACTTGGTAGACGACAAGATGCACGCGCGTTCTACCGGTTCTTACAGCCTGGTTACCCAGCAGCCGCT
GGGTGGTAAGGCGCAGTTCGGTGGTCAGCGTTTCGGGGAGATGGAGGTCTGGGCACTGGAAGCATACGGTGCTGCTTACACTCTG
CAAGAAATGCTCACAGTGAAGTCGGACGATGTGAACGGCCGTACCAAGATGTACAAAAACATCGTGGATGGCGATCACCGTATG
GAGCCGGGCATGCCCGAGTCCTTCAACGTGTTGATCAAGGAAATTCGTTCCCTCGGCATCGATATCGATCTGGAAACCGAATAA

P. fluorescens A506_GCF_000262325.2_CP003041.1

ATGGCTTACTCATATACTGAGAAAAAACGTATCCGCAAGGACTTTAGCAAGTTGCCGGACGTCATGGATGTCCCGTACCTTCTGGC
TATCCAGCTGGATTCGTATCGTGAATTCTTGCAGGCGGGAGCGACCAAAGATCAGTTCCGCGACGTGGGCCTGCATGCGGCCTTC
AAATCCGTTTTCCCGATCATCAGCTACTCCGGCAATGCTGCGCTGGAGTACGTCGGTTATCGCCTGGGCGAACCGGCATTTGATGT
CAAAGAATGCGTGTTGCGTGGCGTTACGTACGCCGTACCTTTGCGGGTAAAAGTCCGTCTGATCATTTTCGACAAAGAATCGTCGA
ACAAAGCGATCAAGGACATCAAAGAGCAAGAAGTCTACATGGGCGAAATCCCACTGATGACTGAAAACGGTACCTTCGTAATCA
ACGGTACCGAGCGTGTAATCGTTTCCCAGCTGCACCGTTCCCCGGGCGTGTTCTTCGACCACGACCGCGGCAAGACGCACAGCTC
CGGCAAACTCCTGTACTCCGCGCGGATCATTCCGTACCGCGGTTCGTGGTTGGACTTCGAGTTCGACCCGAAAGACTGCGTGTTCG

TGCGTATCGACCGTCGTCGCAAGCTGCCGGCCTCGGTATTGCTGCGCGCGCTCGGTTACACCACTGAGCAAGTGCTGGATGCTTTC
TATACCACCAACGTATTCAGCCTGAAGGATGAAACCCTCAAGCTGGAGCTGATCGCTTCGCGTCTGCGTGGTGAAATTGCTGTCCT
GGACATCCAGGACGAAAAAGGCAAGGTCATCGTTGAGGCTGGCCGTCGTATCACTGCGCGCCACATCAACCAGATCGAAAAAGC
CGGTATCAAAGAGCTGGAAGTGCCACTGGACTACGTCCTGGGTCGCACTACCGCCAAGGTCATCGTTCACCCGGCTACAGGCGAA
ATCCTGGCTGAGTGCAACACCGAGCTGAACACCGAGATCCTGGCCAAGATCGCCAAGGCCCAGGTTGTTCGCATCGAGACCCTGT
ACACCAACGATATCGACTGCGGTCCGTTCATCTCCGACACACTGAAGATCGACTCCACCAGCAACCAATTGGAAGCACTGGTCGA
GATCTATCGCATGATGCGTCCTGGTGAGCCACCAACCAAAGACGCTGCCGAAACCCTGTTCAACAACCTGTTCTTCAGCCCTGAG
CGCTATGACCTGTCTGCGGTCGGCCGGATGAAGTTCAACCGTCGTATCGGTCGTACCGAGATCGAAGGTTCGGGCGTGCTGTGCA
AGGAAGACATCGTCGCGGTATTGAAGACCCTGGTCGACATCCGTAACGGCAAAGGCATCGTCGATGACATCGACCACCTGGGTA
ACCGTCGTGTTCGCTGCGTAGGTGAAATGGCCGAGAACCAGTTCCGCGTTGGCCTGGTACGTGTTGAGCGTGCGGTCAAAGAGCG
TCTGTCGATGGCTGAAAGCGAAGGCCTGATGCCGCAAGACCTGATCAACGCCAAGCCAGTGGCTGCGGCGGTGAAAGAGTTCTTC
GGTTCCAGCCAGCTTTCGCAGTTCATGGGCCAGAACAACCCGCTCTCCGAGATCACCCACAAGCGCCGTGTTTCTGCACTGGGCCC
GGGCGGTCTGACCCGTGAGCGCGCTGGCTTTGAAGTCCGTGACGTACACCCGACGCACTACGGTCGTGTTTGCCCGATCGAAACG
CCGGAAGGTCCGAACATCGGTCTGATCAACTCCCTGGCTGCCTATGCGCGCACCAACCAGTACGGCTTCCTCGAAAGCCCGTACC
GTGTGGTGAAAGACGCCCTGGTCACTGACGAGATCGTATTCCTGTCCGCCATCGAAGAAGCTGATCACGTGATCGCTCAGGCTTC
GGCCACGATGAACGACAAGAAAGTCCTGATCGACGAGCTGGTAGCTGTTCGTCACTTGAACGAGTTCACCGTCAAGGCGCCGGA
AGACGTCACCTTGATGGACGTTTCTCCGAAGCAGGTAGTGTCGGTTGCAGCGTCGCTGATCCCGTTCCTGGAACACGATGACGCC
AACCGTGCGTTGATGGGTTCCAACATGCAGCGTCAAGCTGTACCGACCCTGCGTGCTGACAAGCCGCTGGTAGGTACCGGCATGG
AGCGCAACGTAGCCCGTGACTCCGGCGTTTGCGTCGTGGCTCGTCGTGGCGGCGTGATCGACTCCGTTGATGCCAGCCGTATCGTG
GTTCGTGTTGCCGATGACGAAGTAGAAACTGGCGAAGCCGGTGTCGACATCTACAACCTGACCAAATACACCCGCTCGAACCAGA
ACACCTGCATCAACCAGCGTCCGCTGGTGAGCAAGGGTGATCGCGTTCAGCGTAGCGACATCATGGCCGACGGCCCGTCCACCGA
CATGGGTGAGCTGGCTCTGGGTCAGAACATGCGCATCGCGTTCATGGCATGGAACGGCTTCAACTTCGAAGACTCCATCTGCCTGT
CCGAGCGTGTGGTTCAAGAAGACCGCTTCACCACGATCCACATCCAGGAACTGACCTGTGTGGCACGTGATACCAAGCTTGGGCC
AGAGGAAATCACTGCAGACATCCCGAACGTGGGTGAAGCTGCACTGAACAAGCTGGACGAAGCCGGTATCGTTTACGTAGGTGC
TGAAGTTGGCGCTGGCGACATCCTGGTCGGTAAGGTCACTCCGAAAGGCGAGACCCAACTGACGCCGGAAGAGAAGCTGCTGCG
TGCCATCTTCGGTGAAAAAGCCAGCGACGTTAAAGACACTTCCCTGCGTGTACCTACCGGTACCAAGGGTACTGTCATCGACGTA
CAGGTCTTCACCCGTGACGGCGTTGAGCGTGATGCTCGTGCACTGTCCATCGAGAAGACCCAGCTCGACGAGATCCGCAAGGACC
TGAACGAAGAGTTCCGTATCGTTGAAGGCGCGACCTTCGAACGTCTGCGTTCCGCTCTGGTAGGCCACAAGGCTGAAGGCGGCGC
AGGTCTGAAGAAAGGTCAGGACATCACCGACGAAATTCTCGACGGTCTTGAGCACGGCCAGTGGTTCAAACTGCGCATGGCTGAA
GACGCTCTGAACGAGCAGCTCGAGAAGGCCCAGGCCTATATCGTTGATCGCCGCCGTCTGCTGGACGACAAGTTCGAAGACAAG
AAGCGCAAACTGCAGCAGGGCGATGACCTGGCTCCAGGCGTGCTGAAAATCGTCAAGGTTTACCTGGCAATCCGTCGCCGCATCC
AACCGGGCGACAAGATGGCCGGTCGTCACGGTAACAAAGGTGTGGTCTCCGTGATCATGCCGGTTGAAGACATGCCGCACGATG
CCAATGGCACCCCGGTCGACGTCGTCCTTAACCCGTTGGGCGTACCTTCGCGTATGAACGTTGGTCAGATCCTTGAAACCCACCTG
GGCCTCGCGGCCAAAGGTCTGGGCGAGAAGATCAACCGTATGATCGAAGAGCAGCGCAAGGTCGCAGACCTGCGCAAGTTCCTG
CACGAGATCTACAACGAGATCGGCGGTCGCAACGAAGAGCTGGACACCTTCTCCGACCAGGAAATCCTGGATCTGGCGAAGAAC
CTGCGCGGCGGCGTTCCAATGGCTACCCCGGTGTTCGACGGTGCCAAGGAAAGCGAAATCAAGGCCATGCTGAAACTGGCAGAC
CTGCCGGAAAGCGGCCAGATGCAGCTGTTCGACGGCCGTACCGGCAACAAGTTTGAGCGCCCGGTTACTGTTGGCTACATGTACA
TGCTGAAGCTGAACCACTTGGTAGACGACAAGATGCACGCTCGTTCTACCGGTTCGTACAGCCTGGTTACCCAGCAGCCGCTGGG
TGGTAAGGCTCAGTTCGGTGGTCAGCGTTTCGGGGAGATGGAGGTCTGGGCACTGGAAGCATACGGTGCTGCATACACTCTGCAA
GAAATGCTCACAGTGAAGTCGGACGATGTGAACGGTCGTACCAAGATGTACAAAAACATCGTGGACGGCGATCACCGTATGGAG
CCGGGCATGCCCGAGTCCTTCAACGTGTTGATCAAAGAAATTCGTTCCCTCGGCATCGATATCGATCTGGAAACCGAATAA

P. fluorescens F113_GCF_000237065.1_CP003150.1

ATGGCTTACTCATATACTGAGAAAAAACGTATCCGCAAGGACTTTAGCAAGTTGCCGGACGTCATGGATGTGCCGTACCTCCTGG
CCATCCAGCTGGATTCGTATCGTGAATTCTTGCAAGCGGGAGCGACTAAAGATCAGTTCCGCGACGTGGGCCTGCATGCGGCCTT
CAAATCCGTTTTCCCGATCATCAGCTACTCCGGCAATGCTGCGCTGGAGTACGTCGGTTATCGCCTGGGCGAGCCGGCATTTGATG
TCAAAGAATGCGTATTGCGCGGTGTTACTTACGCCGTACCTTTGCGGGTAAAAGTGCGCCTGATCATTTTCGACAAAGAATCGTCG
AACAAAGCGATCAAGGACATCAAAGAGCAAGAAGTCTACATGGGTGAAATCCCCCTGATGACTGAGAACGGTACCTTCGTAATC
AACGGTACCGAGCGTGTAATCGTTTCCCAGCTGCACCGTTCTCCGGGCGTGTTCTTCGACCACGACCGTGGCAAGACGCACAGCT
CCGGCAAACTGCTTTACTCCGCGCGCATCATTCCTTACCGCGGTTCGTGGTTGGACTTCGAGTTCGACCCGAAAGACTGCGTATTC
GTGCGTATCGACCGTCGTCGCAAGCTGCCTGCATCGGTACTGCTGCGCGCGCTCGGCTATACCACCGAAGAAGTGCTGGACGCGT
TCTACACCACCAACGTTTTCCATGTGCAAGGTGAAAACCTCAGCCTGGAACTGGTGCCACAGCGCCTGCGCGGTGAAATCGCTGT
CCTCGATATCCAGGATGACAAAGGCAAGGTTATTGTCGAGCAGGGTCGTCGTATCACCGCTCGCCACATCAACCAGCTGGAAAAA
GCCGGGATCAAAGAGCTGCAGGTGCCTCTGGACTACGTCCTGGGTCGCACCACGGCCAAGGTCATCGTGCATCCGGCCACCGGCG
AAATCCTGGCAGAGTGCAACACCGAGCTGAACACCGAGATCCTGGCGAAAATCGCCAAGGCCCAGGTCGTTCGCATCGAGACGC
TGTACACCAACGACATCGACTGCGGTCCGTTCATCTCCGACACGCTGAAGATCGACTCCACCGGCAACCAACTGGAAGCCCTGGT
CGAGATCTATCGCATGATGCGTCCTGGCGAGCCGCCAACCAAGGATGCCGCCGAGACCCTGTTCAACAACCTGTTCTTCAGCCCT
GAACGCTATGACCTGTCTGCGGTCGGCCGGATGAAGTTCAACCGTCGTATCGGTCGTACCGAGATCGAAGGTTCGGGTGTACTGA
ACAAGGACGACATCGTTGCGGTCCTCAAGACCCTGGTCGACATCCGTAACGGCAAGGGCATCGTCGATGACATCGACCACCTGGG
TAACCGTCGTGTTCGCTGTGTAGGCGAGATGGCCGAGAACCAGTTCCGTGTTGGCCTGGTGCGCGTAGAGCGTGCGGTCAAGGAA
CGTCTGTCGATGGCTGAAAGCGAAGGCCTGATGCCGCAAGACCTGATCAACGCCAAGCCTGTGGCTGCGGCGGTGAAGGAGTTCT
TCGGTTCGAGCCAGCTGTCCCAGTTCATGGACCAGAACAACCCGCTGTCCGAGATCACCAACAAGCGTCGTGTCTCCGCACTCGG
CCCGGGCGGTCTGACCCGTGAGCGTGCAGGCTTCGAAGTTCGTGACGTACACCCGACGCACTACGGTCGTGTATGCCCGATCGAA
ACGCCGGAAGGTCCGAACATCGGTCTGATCAACTCCCTGGCTGCCTATGCGCGCACCAACCAGTACGGCTTCCTCGAGAGCCCGT

ACCGTGTGGTGAAAGACGCCCTGGTGACCGACGAGATCGTGTTCCTGTCCGCCATCGAAGAAGCTGATCACGTGATCGCCCAGGC
TTCGGCCACGATGAACGACAAGAAGGTCCTGGTCGACGAGCTGGTAGCTGTTCGTCACTTGAACGAGTTCACCGTCAAGGCGCCT
GAAGACGTCACCTTGATGGACGTGTCGCCGAAGCAGGTAGTTTCGGTTGCAGCGTCGTTGATCCCGTTCCTCGAGCACGACGACG
CCAACCGTGCGTTGATGGGTTCGAACATGCAGCGTCAAGCTGTACCCACCCTGCGTGCCGACAAGCCGCTGGTAGGTACCGGCAT
GGAGCGTAACGTTGCCCGTGACTCCGGCGTTTGCGTCGTGGCTCGTCGTGGTGGCGTGATCGACTCCGTCGACGCCAGCCGTATCG
TGGTTCGTGTTGCCGATGACGAAGTTGAAACTGGCGAAGCCGGTGTCGACATCTACAACCTGACCAAATACACCCGCTCCAACCA
GAACACCTGCATCAACCAGCGTCCGCTGGTGAGCAAGGGTGATCGGGTTCAGCGCAGCGACATCATGGCCGACGGTCCGTCCACC
GATATGGGTGAGCTGGCGCTGGGTCAGAACATGCGCATCGCGTTCATGGCATGGAACGGCTTCAACTTCGAAGACTCCATCTGCC
TGTCCGAGCGTGTGGTTCAGGAAGACCGCTTCACCACGATCCACATCCAGGAACTGACCTGTGTGGCTCGTGACACCAAGCTTGG
CCCAGAGGAAATCACTGCGGACATCCCGAACGTGGGTGAAGCTGCACTGAACAAGCTGGACGAAGCCGGTATCGTTTACGTAGG
TGCTGAAGTAGGCGCAGGCGACATCCTGGTGGGCAAGGTCACTCCGAAAGGCGAGACCCAGCTGACTCCGGAAGAAAAACTGCT
GCGTGCCATCTTCGGTGAAAAAGCCAGCGACGTTAAAGACACCTCCCTGCGCGTGCCTACCGGCACCAAGGGTACCGTCATCGAC
GTACAAGTCTTCACTCGCGACGGTGTTGAGCGTGATGCTCGTGCACTGTCCATCGAGAAGACTCAACTCGACGAGATCCGCAAGG
ACCTGAACGAAGAGTTCCGTATCGTCGAAGGCGCAACTTTCGAGCGTCTGCGCTCCGCTCTGGTCGGCCACAAAGCCGAAGGCGG
CGCCGGCCTGAAGAAAGGTCAGGAAATCACCGACGAAGTTCTCGACGGTCTTGAGCATGGTCAGTGGTTCAAACTGCGCATGGCT
GAAGATGCTCTGAACGAGCAGCTCGAGAAGGCCCAGGCCTATATCGTTGATCGCCGCCGTCTGCTGGACGACAAGTTCGAAGACA
AGAAGCGCAAACTGCAGCAGGGCGATGACCTGGCTCCGGGCGTGCTGAAAATCGTCAAGGTTTACCTGGCAATCCGTCGCCGCAT
CCAGCCGGGCGACAAGATGGCCGGTCGTCACGGTAACAAAGGTGTGGTCTCCGTGATCATGCCGGTTGAAGACATGCCGCACGAT
GCCAATGGCACCCCGGTCGATGTGGTCCTCAACCCGTTGGGCGTACCTTCGCGTATGAACGTTGGTCAGATCCTTGAAACCCACCT
GGGCCTCGCGGCCAAGGGCTTGGGCGAGAAGATCAACCGTATGATCGAAGAGCAGCGCAAGGTCGCGGACCTGCGTAAGTTCCT
GCACGAGATCTACAACGAGATCGGCGGTCGCAACGAAGAGCTGGACACCTTCTCCGACCAGGAAATCCTGGACCTGGCGAAGAA
CCTGCGCGGCGGCGTTCCAATGGCTACTCCGGTGTTCGACGGTGCCAAGGAAAGCGAAATCAAGGCCATGCTGAAACTGGCAGA
CCTGCCAGAAAGCGGCCAGATGCAGCTGTTCGACGGCCGTACCGGCAACAAGTTCGAGCGCCCGGTTACCGTTGGCTACATGTAC
ATGCTGAAGCTGAACCACTTGGTAGACGACAAGATGCACGCTCGTTCTACCGGTTCTTACAGCCTGGTTACCCAGCAGCCGCTGG
GTGGTAAGGCGCAGTTCGGTGGTCAGCGTTTCGGGGAGATGGAGGTCTGGGCACTGGAAGCGTACGGTGCTGCATACACTCTGCA
AGAAATGCTCACAGTGAAGTCGGACGATGTGAACGGCCGGACCAAGATGTACAAAAACATCGTGGACGGCGATCACCGTATGGA
GCCGGGCATGCCCGAGTCTTTCAACGTGTTGATCAAGGAAATTCGTTCCCTCGGCATCGATATCGATCTGGAAACCGAATAA

P. fluorescens NCIMB 11764_GCF_000293885.2_CP010945.1

ATGGCTTACTCATATACTGAGAAAAAACGTATCCGCAAGGACTTTAGCAAGTTGCCGGACGTCATGGATGTGCCGTATCTCCTGG
CAATCCAGCTGGATTCGTATCGTGAATTCTTGCAGGCGGGAGCGACTAAAGATCAGTTCCGCGACGTGGGCCTGCATGCGGCCTT
CAAATCCGTTTTCCCGATCATCAGCTACTCCGGCAATGCTGCGCTGGAGTACGTCGGTTATCGCCTGGGCGAACCGGCATTTGATG
TCAAAGAATGCGTATTGCGCGGTGTAACTTACGCCGTACCTTTGCGGGTAAAAGTGCGCCTGATCATTTTCGACAAAGAATCGTCG
AACAAAGCGATCAAGGACATCAAAGAGCAAGAAGTCTACATGGGTGAAATCCCCCTGATGACTGAGAACGGTACCTTCGTAATC
AACGGTACCGAGCGTGTAATCGTTTCCCAGCTGCACCGTTCCCCGGGCGTGTTCTTCGACCACGACCGTGGCAAGACGCACAGCT
CCGGCAAACTGCTGTACTCCGCGCGCATCATTCCTTACCGCGGTTCGTGGCTGGACTTCGAGTTCGACCCGAAAGACTGCGTGTTC
GTGCGTATCGACCGTCGTCGCAAGCTGCCTGCATCGGTACTGCTGCGCGCGCTCGGCTATACCACTGAAGAAGTGCTCGACGCGT
TCTACACCACCAACGTTTTCCACCTGAGCGGCGAAACCCTCAGTCTGGAACTGATTGCTTCGCGTCTGCGTGGTGAAATCGCTGTT
CTTGATATTCAGGACGAGAAGGGCAAGGTCATCGTTGAGGCTGGTCGCCGTATTACTGCGCGCCACATCAACCAGATCGAAAAAG
CCGGTCTCAAGACCCTTGACGTGCCTCTGGAGTACGTTCTGGGTCGCACTACCGCCAAGGCCATCGTGCATCCGGCAACCGGCGA
AATCCTGGCAGAGTGCAACACCGAGCTGAACACCGAGATCCTGGCAAAAATCGCCAAGGCCCAGGTTGTTCGCATCGAGACTCTG
TACACCAACGATATCGACTGCGGTCCGTTCGTCTCCGACACCCTGAAGATCGACTCCACCAGCAACCAATTGGAAGCGCTGGTCG
AGATCTATCGCATGATGCGTCCAGGCGAGCCGCCAACCAAAGACGCTGCCGAGACCCTGTTCAACAACCTGTTCTTCAGCCCTGA
GCGCTATGACCTGTCTGCGGTCGGCCGGATGAAGTTCAACCGTCGTATCGGTCGTACCGAGATCGAAGGTTCGGGCGTGTTGTGC
AAGGAAGACATCGTCGCGGTACTGAAGACTCTGGTCGACATCCGTAACGGTAAAGGCATCGTCGATGACATCGACCACCTGGGTA
ACCGTCGTGTTCGCTGCGTAGGCGAAATGGCCGAGAACCAGTTCCGCGTTGGCCTGGTACGTGTTGAGCGTGCGGTCAAAGAGCG
TCTGTCGATGGCTGAAAGCGAAGGCCTGATGCCGCAAGACCTGATCAACGCCAAGCCAGTGGCTGCGGCGGTGAAAGAGTTCTTC
GGTTCCAGCCAGCTGTCCCAGTTCATGGACCAGAACAACCCGCTGTCCGAGATCACCCACAAGCGTCGTGTGTCTGCACTCGGCC
CTGGCGGTTTGACCCGTGAGCGTGCTGGCTTTGAAGTGCGTGACGTACACCCGACTCACTACGGTCGTGTATGCCCGATTGAAACG
CCGGAAGGTCCGAACATCGGTCTGATCAACTCCCTGGCCGCTTATGCGCGCACCAACCAGTACGGCTTCCTCGAGAGCCCGTACC
GCGTGGTGAAAGACGCTCTGGTCACCGACGAGATCGTGTTCCTGTCTGCCATCGAAGAAGCTGATCATGTGATCGCTCAGGCTTC
GGCCACGATGAACGACAAGAAAGTCCTGATCGACGAGCTGGTAGCTGTTCGTCACTTGAACGAGTTCACCGTCAAGGCGCCGGA
AGACGTCACCTTGATGGACGTATCGCCGAAGCAGGTAGTTTCGGTTGCAGCGTCGCTGATCCCGTTCCTCGAGCACGACGACGCC
AACCGTGCGTTGATGGGTTCGAACATGCAGCGTCAAGCTGTACCAACCCTGCGCGCTGACAAGCCGCTGGTAGGTACTGGCATGG
AGCGTAACGTAGCTCGTGACTCCGGCGTTTGCGTCGTGGCTCGTCGTGGCGGCGTTATCGATTCCGTCGATGCCAGCCGTATCGTG
GTTCGTGTTGCTGATGACGAAGTTGAAACCGGCGAAGCTGGTGTCGACATCTACAACCTGACCAAGTACACCCGCTCCAACCAGA
ACACCTGCATCAACCAGCGTCCGCTGGTGCGTAAGGGTGATCGGGTTCAGCGTAGCGACATCATGGCCGACGGTCCGTCCACCGA
TATGGGTGAACTGGCGCTGGGTCAGAACATGCGCATCGCGTTCATGGCATGGAACGGCTTCAACTTCGAAGACTCCATCTGCCTG
TCCGAGCGTGTGGTTCAGGAAGATCGCTTCACCACGATCCACATTCAGGAACTGACCTGTGTGGCACGTGACACCAAGCTTGGGC
CAGAGGAAATCACTGCAGACATCCCGAACGTGGGTGAAGCTGCACTGAACAAGCTGGACGAAGCCGGTATCGTTTACGTAGGTG
CTGAAGTTGGCGCAGGCGACATCCTGGTTGGTAAGGTCACTCCGAAAGGCGAGACCCAGCTGACTCCGGAAGAAAAACTGCTGC
GTGCCATCTTCGGTGAAAAAGCCAGCGACGTTAAAGACACCTCCCTGCGCGTGCCTACCGGCACCAAGGGCACTGTCATCGACGT
ACAGGTCTTCACCCGCGACGGCGTTGAGCGTGATGCTCGTGCACTGTCGATCGAGAAGACTCAACTCGACGAGATCCGCAAGGAT
CTGAACGAAGAGTTCCGTATCGTTGAAGGCGCCACTTTCGAACGTCTGCGTTCCGCTCTGGTCGGCCACAAAGCCGAAGGCGGCG

CCGGTCTGAAGAAAGGTCAGGACATCACCGACGAAGTCCTCGACGGTCTTGAGCATGGTCAGTGGTTCAAACTGCGCATGGCTGA
AGATGCTCTGAACGAGCAGCTCGAGAAGGCTCAGGCCTATATCGTTGATCGCCGCCGTCTGCTGGACGACAAGTTCGAAGACAAG
AAGCGCAAACTGCAGCAGGGCGATGACCTGGCTCCAGGCGTGCTGAAAATCGTTAAGGTTTACCTGGCAATCCGTCGTCGCATCC
AGCCGGGCGACAAGATGGCCGGTCGTCACGGTAACAAAGGTGTGGTCTCCGTGATCATGCCGGTTGAAGACATGCCGCACGATG
CCAATGGCACCCCGGTCGACGTCGTCCTCAACCCGTTGGGCGTACCTTCGCGTATGAACGTTGGTCAGATCCTTGAAACCCACCTC
GGCCTCGCGGCCAAAGGTCTGGGCGAGAAGATCAACCGGATGATCGAAGAGCAGCGCAAAGTCGCCGAGCTTCGTAAATTCCTC
GACGAGATCTACAACCAGATCGGCGGCCGTAACGAAGATCTGGATAGCTTCTCCGACCAGGAAATCCTGGATCTGGCGAAGAAC
CTGCGTGGCGGCGTTCCAATGGCCACTCCAGTGTTCGACGGTGCCAAGGAAAGCGAAATCAAGGCCATGCTGAAACTGGCAGAC
CTGCCAGAAAGCGGCCAGATGCAGCTGACCGACGGCCGTACCGGCAACAAGTTCGAGCGCCCGGTTACTGTTGGCTATATGTACA
TGCTGAAGCTGAACCACTTGGTAGACGACAAGATGCACGCTCGTTCTACCGGTTCGTACAGCCTGGTTACCCAGCAGCCGCTGGG
TGGTAAGGCGCAGTTCGGTGGTCAGCGTTTCGGGGAGATGGAGGTCTGGGCACTGGAAGCATACGGTGCTGCTTACACTCTGCAA
GAAATGCTCACAGTGAAGTCGGACGATGTGAACGGCCGTACCAAGATGTACAAAAACATCGTGGATGGCGATCACCGTATGGAG
CCGGGCATGCCCGAGTCCTTCAACGTGTTGATCAAGGAAATTCGTTCCCTCGGCATCGATATCGATCTGGAAACCGAATAA

P. fluorescens Pf0-1_GCF_000012445.1_CP000094.2

ATGGCTTACTCATATACTGAGAAAAAACGTATCCGCAAGGACTTTAGCAAGTTGCCGGACGTCATGGATGTGCCTTACCTCCTGGC
CATCCAGCTGGATTCGTATCGTGAATTCTTGCAAGCGGGAGCGACTAAAGATCAGTTCCGCGACGTGGGCCTGCATGCGGCCTTC
AAATCCGTTTTCCCGATCATCAGCTACTCCGGCAATGCTGCGCTGGAGTACGTCGGTTATCGCCTGGGCGAACCGGCATTTGATGT
CAAAGAATGCGTATTGCGCGGTGTAACTTTCGCCGTACCTTTGCGGGTAAAAGTGCGCCTGATCATTTTCGACAAAGAATCGTCGA
ACAAAGCGATCAAGGACATCAAAGAGCAAGAAGTCTACATGGGTGAAATCCCATTGATGACTGAGAACGGTACCTTCGTAATCA
ACGGTACCGAGCGTGTTATCGTTTCCCAGCTGCACCGTTCCCCGGGCGTGTTCTTCGACCACGACCGTGGCAAGACGCACAGCTCC
GGCAAGCTGCTGTACTCCGCGCGCATCATTCCTTACCGCGGTTCGTGGCTGGACTTCGAGTTCGACCCGAAAGACTGCGTATTCGT
GCGTATCGACCGTCGTCGCAAGCTGCCGGCCTCGGTACTGCTGCGCGCGCTCGGTTATACCACCGAGGAAGTGCTGGACGCGTTC
TACACTACCAACGTATTCCACGTGAAAGGCGAAGGCCTGAGCCTGGAGCTGGTACCTCAGCGTCTGCGTGGTGAAGTTGCGGTTC
TCGATATCCAGGACGACAAAGGCAAGGTGATCGTTGAGCAGGGCCGTCGTATTACTGCCCGCCACATCAACCAGCTGGAAAAAG
CCGGGATCAAAGAGCTGGACGTGCCTCTGGACTACGTTCTGGGTCGTACCACCGCCAAGGCCATCGTGCACCCGGCTACCGGTGA
GATCCTGGCAGAGTGCAACACCGAGCTTTCGACCGAGATCCTGGCGAAAATCGCCAAGGCCGGCGTCGTACGCATCGAAACTCTG
TACACCAACGACATCGACTGCGGTCCGTTCGTCTCCGACACGCTGAAGATCGACTCCACCAGTAACCAATTGGAAGCGCTGGTCG
AGATCTATCGCATGATGCGTCCGGGTGAACCGCCAACCAAAGACGCTGCCGAGACCCTGTTCAACAACCTGTTCTTCAGTCCTGA
GCGCTATGACCTGTCTGCGGTCGGCCGGATGAAGTTCAACCGTCGTATCGGTCGTACCGAGATCGAAGGTTCGGGCGTGCTGTGC
AAGGAAGACATCGTCGCGGTACTGAAGACTCTGGTCGACATCCGTAACGGTAAAGGCATCGTCGACGACATCGACCACCTGGGT
AACCGTCGTGTTCGCTGCGTAGGCGAAATGGCCGAGAACCAGTTCCGCGTTGGCCTGGTACGTGTTGAGCGTGCGGTCAAAGAGC
GTCTGTCGATGGCTGAAAGCGAAGGCCTGATGCCGCAAGATCTGATCAACGCCAAGCCAGTGGCTGCGGCGGTGAAAGAGTTCTT
CGGTTCCAGCCAGCTGTCGCAGTTCATGGACCAGAACAACCCGCTGTCCGAGATCACCCACAAGCGTCGTGTCTCTGCACTCGGC
CCTGGCGGTCTGACTCGTGAGCGCGCGGGCTTCGAAGTCCGTGACGTACACCCGACTCACTATGGTCGTGTCTGCCCGATTGAAA
CGCCGGAAGGTCCGAACATCGGGCTGATCAACTCCCTGGCTGCTTACGCTCGCACCAACCAGTACGGCTTCCTCGAGAGCCCGTA
CCGCGTGGTGAAAGAGGGTGTGGTCACCGACGACATCGTGTTCCTGTCCGCCATTGAAGAAGCTGATCACGTGATCGCGCAGGCT
TCGGCGACCATGAACGAGCAGAAAGTCCTGATCGATGAACTGGTGGCCGTGCGTCACCTGAACGAATTCACCGTCAAGGCGCCG
GAAGACGTCACCTTGATGGACGTTTCGCCGAAGCAGGTAGTTTCTGTTGCAGCGTCGCTGATTCCGTTCCTCGAGCATGACGACGC
CAACCGTGCGTTGATGGGTTCGAACATGCAGCGTCAGGCTGTACCAACCCTGCGTGCTGACAAGCCGCTGGTCGGTACCGGCATG
GAGCGTAACGTGGCTCGCGACTCCGGCGTTTGCGTCGTGGCTCGTCGTGGCGGCGTGATCGACTCCGTTGATGCCAGCCGTATCGT
GGTTCGTGTTGCTGATGACGAAGTTGAAACCGGCGAAGCTGGTGTCGACATCTACAACCTGACCAAATACACCCGCTCGAACCAG
AACACCTGCATCAACCAGCGTCCGCTGGTGAGCAAAGGTGATCGCGTTCAGCGTAGCGACATCATGGCCGACGGCCCGTCCACCG
ACATGGGTGAGCTGGCTCTGGGTCAGAACATGCGCATCGCGTTCATGGCATGGAACGGCTTCAACTTCGAAGACTCCATCTGCCTT
TCCGAGCGTGTGGTGCAGGAAGACCGCTTCACCACGATCCACATTCAGGAACTGACCTGTGTGGCCCGTGACACCAAGCTTGGCC
CAGAGGAAATCACTGCGGACATCCCGAACGTGGGTGAAGCTGCGCTGAACAAGCTGGACGAAGCCGGTATCGTTTACGTAGGTG
CTGAAGTTGGCGCAGGCGACATCCTGGTAGGCAAGGTCACTCCGAAAGGCGAGACCCAACTGACTCCGGAAGAAAAACTGCTGC
GCGCAATCTTCGGTGAAAAAGCCAGCGACGTTAAAGACACTTCCCTGCGCGTGCCAACGGGCACCAAAGGCACTGTCATCGACGT
ACAGGTCTTCACGCGTGACGGCGTTGAGCGTGATGCTCGTGCACTGTCGATCGAGAAGTCCCAGCTGGACGAGATCCGCAAGGAT
CTGAACGAAGAGTTCCGCATCGTTGAAGGCGCGACCTTCGAGCGTCTGCGTTCCGCTCTGGTAGGCCACAAGGCTGAAGGCGGCG
CCGGCCTGAAGAAAGGTCAGGACATCACCGACGAAGTACTCGACGGTCTTGAGCATGGTCAGTGGTTCAAACTGCGCATGGCTGA
AGATGCTCTGAACGAGCAGCTCGAGAAGGCTCAGGCCTACATCGTTGATCGTCGCCGTCTGCTGGACGACAAGTTCGAAGACAAG
AAGCGCAAACTGCAGCAGGGCGATGACCTGGCTCCAGGCGTGCTGAAGATCGTCAAGGTTTACCTGGCAATCCGTCGTCGCATCC
AGCCGGGCGACAAGATGGCCGGTCGTCACGGTAACAAAGGTGTGGTCTCCGTGATCATGCCGGTTGAAGACATGCCGCACGATG
CCAATGGCACCCCGGTCGACGTGGTCCTCAACCCGCTGGGCGTACCTTCGCGTATGAACGTTGGTCAGATCCTTGAAACCCACCTG
GGCCTCGCGGCCAAAGGTCTGGGCGAGAAGATCAACCGGATGGTCGAAGAGCAGCGCAAAGTCGCTGAACTGCGTACCTTCCTG
GACGAGATCTACAACCAGATCGGCGGTCGTAACGAAGATCTGGACAGCTTCTCCGATCAGGAAATCCTGGATCTGGCGAAGAAC
CTGCGTGGCGGTGTTCCAATGGCCACTCCAGTGTTCGACGGTGCCAAGGAAAGCGAAATCAAGGCCATGCTGAAACTGGCAGACC
TGCCAGAAAGCGGCCAGATGCAGCTGACCGACGGCCGTACCGGCAACAAGTTCGAGCGTCCAGTTACCGTTGGCTACATGTACAT
GCTGAAGCTGAACCACTTGGTAGACGACAAGATGCACGCGCGTTCTACCGGTTCGTACAGCCTGGTTACCCAGCAGCCGCTGGGT
GGTAAGGCGCAGTTCGGTGGTCAGCGTTTCGGGGAGATGGAGGTCTGGGCACTGGAAGCATACGGTGCTGCTTACACTCTGCAAG
AAATGCTCACAGTGAAGTCGGACGATGTGAACGGCCGGACCAAGATGTACAAAAACATCGTGGACGGCGATCACCGTATGGAGC
CGGGCATGCCCGAGTCCTTCAACGTGTTGATCAAGGAAATTCGTTCCCTCGGCATCGATATCGATCTGGAAACCGAATAA

P. fluorescens SBW25_ GCF_000009225.2_AM181176.4

ATGGCTTACTCATATACTGAGAAAAAACGTATCCGCAAGGACTTTAGCAAGTTGCCGGACGTCATGGATGTCCCGTACCTTCTGGC
TATCCAGCTGGATTCGTATCGTGAATTCTTGCAGGCGGGAGCGACCAAAGATCAGTTCCGCGACGTGGGCCTGCATGCGGCCTTC
AAATCCGTTTTCCCGATCATCAGCTACTCCGGCAATGCTGCGCTGGAGTACGTCGGTTATCGCCTGGGCGAACCGGCATTTGATGT
CAAAGAATGCGTGTTGCGCGGTGTCACGTACGCCGTACCTTTGCGGGTAAAAGTCCGCCTGATCATTTTCGACAAAGAATCGTCG
AACAAAGCGATCAAGGACATCAAAGAGCAAGAAGTCTACATGGGCGAAATCCCATTGATGACTGAGAACGGTACCTTCGTTATC
AACGGTACCGAGCGTGTTATCGTTTCCCAGCTGCACCGTTCCCCGGGCGTGTTCTTCGACCACGACCGCGGCAAGACGCACAGCT
CCGGCAAACTCCTGTACTCCGCGCGGATCATTCCGTACCGCGGTTCGTGGTTGGACTTCGAGTTCGACCCGAAAGACTGCGTGTTC
GTGCGTATCGACCGTCGTCGCAAGCTGCCGGCCTCGGTACTGCTGCGTGCGCTCGGCTATACCACTGAGCAAGTGCTGGACGCTTT
CTACACTACCAACGTATTCAGCCTGAAGGATGAAACCCTCAGCCTGGAGCTGATTGCTTCGCGTCTGCGTGGTGAAATTGCCGTAC
TGGACATCCAGGATGAAAAGGGCAAGGTCATTGTTGAAGCTGGCCGTCGTATTACTGCGCGCCACATCAACCAGATCGAAAAAGC
CGGTATCAAGTCGTTGGACGTGCCTCTGGACTACGTCCTGGGTCGCACCACCGCTAAGGTTATTGTTCACCCGGCCACAGGCGAA
ATCCTGGCTGAGTGCAACACCGAGCTGAACACCGAGATCCTGGCAAAAATCGCCAAGGCTCAGGTCGTTCGCATCGAAACCCTGT
ATACCAACGATATCGACTGCGGTCCGTTCATCTCCGACACATTGAAGATCGACTCCACCAGCAACCAATTGGAAGCGCTGGTCGA
GATCTATCGCATGATGCGTCCTGGCGAGCCACCAACCAAAGACGCTGCCGAAACCCTGTTCAACAACCTGTTCTTCAGCCCTGAG
CGTTATGACCTGTCTGCGGTCGGCCGGATGAAGTTCAACCGTCGTATCGGTCGTACCGAGATCGAAGGTTCAGGCGTGCTGTGCA
AGGAAGACATCGTTGCGGTCCTGAAGACTCTGGTCGACATCCGTAACGGCAAAGGCATTGTCGATGACATCGACCACCTGGGTAA
CCGTCGTGTTCGCTGCGTAGGCGAGATGGCCGAGAACCAGTTCCGCGTTGGCCTGGTACGTGTTGAGCGTGCGGTCAAAGAGCGT
CTGTCGATGGCTGAAAGCGAAGGCCTGATGCCGCAAGACCTGATCAACGCCAAGCCAGTGGCTGCGGCGGTGAAAGAGTTCTTC
GGTTCCAGCCAGCTCTCGCAGTTCATGGACCAGAACAACCCGCTCTCCGAGATCACCCACAAGCGCCGTGTTTCCGCACTGGGCC
CGGGCGGTCTGACGCGTGAACGTGCAGGCTTTGAAGTTCGTGACGTACACCCGACGCACTATGGTCGTGTATGCCCGATCGAAAC
GCCGGAAGGTCCGAACATCGGTCTGATCAACTCCCTGGCCGCTTATGCGCGCACCAACCAGTATGGCTTCCTCGAGAGCCCGTAC
CGTGTGGTGAAAGACGCTCTGGTCACCGACGAGATCGTATTCCTGTCCGCCATCGAAGAAGCTGATCACGTGATCGCTCAGGCTT
CGGCCACGATGAACGACAAGAAAGTCCTGATCGACGAGCTGGTAGCTGTTCGTCACTTGAACGAGTTCACCGTCAAGGCGCCGGA
AGACGTCACCTTGATGGACGTATCGCCGAAGCAGGTAGTTTCGGTTGCTGCGTCGCTGATCCCGTTCCTGGAGCACGATGACGCC
AACCGTGCGTTGATGGGTTCCAACATGCAGCGTCAAGCTGTACCCACCCTGCGTGCTGACAAGCCGCTGGTAGGTACCGGCATGG
AGCGTAACGTAGCCCGTGACTCCGGCGTTTGCGTCGTGGCTCGTCGTGGCGGCGTGATCGACTCCGTTGATGCCAGCCGTATCGTG
GTTCGTGTTGCCGATGACGAAGTTGAAACTGGCGAAGCCGGTGTCGACATCTACAACCTGACCAAATACACCCGCTCGAACCAGA
ACACCTGCATCAACCAGCGTCCGCTGGTACGTAAGGGTGATCGCGTTCAGCGCAGCGACATCATGGCTGACGGCCCGTCCACCGA
CATGGGTGAATTGGCACTGGGTCAGAACATGCGCATCGCGTTCATGGCATGGAACGGCTTCAACTTCGAAGACTCCATCTGCCTG
TCCGAGCGTGTTGTTCAAGAAGACCGTTTCACCACGATCCACATTCAGGAACTGACCTGTGTGGCACGTGACACCAAGCTTGGGC
CAGAAGAAATCACTGCAGACATCCCGAACGTGGGTGAAGCTGCACTGAACAAGCTGGACGAAGCCGGTATCGTTTACGTAGGTG
CTGAAGTAGGCGCAGGCGACATCCTGGTTGGTAAGGTCACTCCGAAAGGCGAGACCCAACTGACGCCGGAAGAAAAACTGCTGC
GTGCCATCTTCGGTGAAAAAGCCAGCGACGTTAAAGACACCTCCCTGCGCGTACCTACCGGTACCAAGGGCACTGTCATCGACGT
ACAGGTCTTCACTCGTGATGGTGTTGAGCGTGATGCTCGTGCGCTGTCCATCGAGAAGACTCAACTCGACGAGATCCGCAAGGAC
CTGAACGAAGAGTTCCGTATCGTTGAAGGCGCGACCTTCGAACGTCTGCGTTCCGCGCTGGTAGGCCACAAGGCCGAAGGCGGCG
CAGGCCTGAAAAAAGGTCAGGACATCACCGACGAAATCCTCGACGGTCTTGAGCACGGCCAGTGGTTCAAACTGCGCATGGCTG
AAGATGCTCTGAACGAGCAGCTCGAGAAGGCCCAGGCCTACATCGTTGATCGCCGCCGTCTGCTGGACGACAAGTTCGAAGACA
AGAAGCGCAAACTGCAGCAGGGCGATGACCTGGCTCCAGGCGTGCTGAAAATCGTCAAGGTTTACCTGGCAATCCGTCGCCGCAT
CCAGCCGGGCGACAAGATGGCCGGTCGTCACGGTAACAAGGGTGTGGTCTCCGTGATCATGCCGGTTGAAGACATGCCGCACGAT
GCCAATGGCACCCCGGTCGACGTCGTCCTCAACCCATTGGGCGTACCTTCGCGTATGAACGTTGGTCAGATCCTTGAAACCCACCT
GGGCCTCGCGGCTAAAGGTTTGGGCGAGAAGATCAACCGTATGATCGAAGAGCAGCGCAAGGTTGCTGACCTGCGCAAGTTCCTG
CACGAGATCTACAACGAGATCGGCGGCCGCAAAGAAGAGCTGGATACCTTCTCCGACCAGGAAATCCTGGATCTGGCGAAGAAC
CTGCGCGGCGGCGTTCCAATGGCTACCCCGGTGTTCGACGGTGCCAAGGAAAGCGAAATCAAGGCCATGCTGAAACTGGCAGAT
CTGCCAGAAAGCGGCCAGATGCAGCTGTTCGACGGCCGTACCGGCAACAAGTTTGAGCGCCCGGTTACTGTTGGCTACATGTACA
TGCTGAAGCTGAACCACTTGGTAGACGACAAGATGCACGCTCGTTCTACCGGTTCGTACAGCCTGGTTACCCAGCAGCCGCTGGG
TGGTAAGGCTCAGTTCGGTGGTCAGCGTTTCGGGGAGATGGAGGTCTGGGCACTGGAAGCATACGGTGCTGCTTACACTCTGCAA
GAAATGCTCACAGTGAAGTCGGACGATGTGAACGGTCGGACCAAGATGTACAAAAACATCGTGGACGGCGATCACCGTATGGAG
CCGGGCATGCCCGAGTCTTTCAACGTGTTGATCAAAGAAATTCGTTCCCTCGGCATCGATATCGATCTGGAAACCGAATAA
